# Recent Strategies in the Nucleophilic Dearomatization of Pyridines, Quinolines, and Isoquinolines

**DOI:** 10.1021/acs.chemrev.3c00625

**Published:** 2024-01-02

**Authors:** Marcos Escolano, Daniel Gaviña, Gloria Alzuet-Piña, Santiago Díaz-Oltra, María Sánchez-Roselló, Carlos del Pozo

**Affiliations:** † Department of Organic Chemistry, Faculty of Pharmacy, University of Valencia, 46100 Burjassot, Valencia, Spain; ‡ Department of Inorganic Chemistry, Faculty of Pharmacy, University of Valencia, 46100 Burjassot, Valencia, Spain

## Abstract

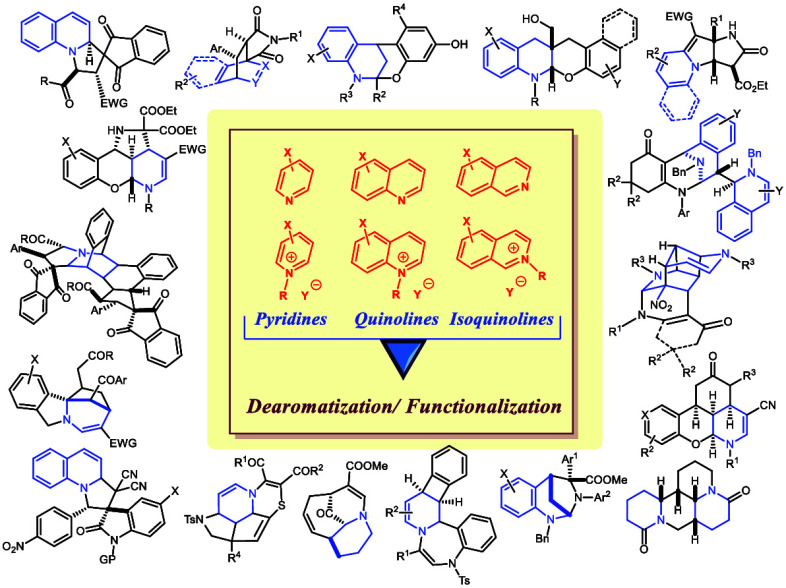

Dearomatization reactions have become fundamental chemical transformations in organic synthesis since they allow for the generation of three-dimensional complexity from two-dimensional precursors, bridging arene feedstocks with alicyclic structures. When those processes are applied to pyridines, quinolines, and isoquinolines, partially or fully saturated nitrogen heterocycles are formed, which are among the most significant structural components of pharmaceuticals and natural products. The inherent challenge of those transformations lies in the low reactivity of heteroaromatic substrates, which makes the dearomatization process thermodynamically unfavorable. Usually, connecting the dearomatization event to the irreversible formation of a strong C–C, C–H, or C–heteroatom bond compensates the energy required to disrupt the aromaticity. This aromaticity breakup normally results in a 1,2- or 1,4-functionalization of the heterocycle. Moreover, the combination of these dearomatization processes with subsequent transformations in tandem or stepwise protocols allows for multiple heterocycle functionalizations, giving access to complex molecular skeletons. The aim of this review, which covers the period from 2016 to 2022, is to update the state of the art of nucleophilic dearomatizations of pyridines, quinolines, and isoquinolines, showing the extraordinary ability of the dearomative methodology in organic synthesis and indicating their limitations and future trends.

## 1 Introduction

Readily available nitrogen-containing aromatic compounds constitute fundamental chemical feedstocks.^1^ Among them, pyridines, quinolines, and isoquinolines occupy a prominent place as these scaffolds are prevalent in a wide variety of pharmacologically active synthetic and natural compounds. These heterocycles are common fragments of a vast majority of marketed drugs and they can serve as useful tools to manipulate the lipophilicity, polarity, and hydrogen-bonding capacity of molecules, which may lead to improved pharmacological and pharmacokinetic profiles.

However, a recent structural analysis of drug candidates has discovered that successful drugs have a higher percentage of *sp*^3^-carbons and are more likely to contain stereogenic centers.^2,3^ Improved solubility^4^ and a less promiscuous binding behavior^5^ are the reasons that explain the better pharmacological profiles of *sp*^3^-rich and chiral molecules. In contrast to this apparent need, the most commonly used reactions in medicinal chemistry comprise amide bond formation, the Suzuki–Miyaura reaction, and nucleophilic aromatic substitution, methodologies that are focused on the creation of *sp*^2^-carbons.^6^

In this context, it was recently stated by researchers from Merck and Janssen companies the paramount importance of synthetic organic chemistry in the pharmaceutical industry.^7^ They recognized that selective saturation and functionalization of heteroaromatics was an unsolved problem in synthetic chemistry. In fact, the high frequency of *sp*^3^-carbons and chiral centers in successful drugs has been termed in several articles as “escape from flatland”.^8^ The development of more efficient tools to construct nonplanar heterocyclic structures is a pressing need in the pharmaceutical industry.

The most direct way to achieve this goal is the dearomatization of heteroarenes to access partially or fully saturated heterocycles. When performed on electron deficient heteroarenes such as pyridines, quinolines, or isoquinolines, different types of nonaromatic nitrogen-containing derivatives will be formed. These *N*-heterocycles are relevant structural components of pharmaceuticals and natural products, as it was revealed by a recent analysis of FDA approved drugs, indicating that 59% of small molecule drugs contain at least one *N*-heterocycle, with the piperidine ring being the most prevalent one.^9,10^

Clearly, a dearomatization reaction entails an inherent difficulty resulting from the large energy input necessary for the disruption of the aromaticity. In the case of pyridines, quinolines, and isoquinolines, the lone pair of electrons on the nitrogen atom is not involved in the aromaticity and it reacts with electrophiles, rendering the corresponding heteroarenium salts, thereby reducing the electron density and making the nucleophilic dearomatization more facile. On the other hand, the direct dearomatization reaction on the heterocycle is less favorable and electron-withdrawing groups or strong nucleophiles are needed to achieve that goal. Despite those inconveniences, several methods have been devised in the past decade in order to overcome the aromaticity issue while controlling regio-, diastereo-, and enantioselectivity, converting this strategy in one of the best ways to access saturated nitrogen heterocycles and opening new ways to explore chemical spaces previously unexploited.^11,12^

### 1.1 Scope and Organization of the Review

In the present review, we will highlight the novel synthetic strategies employed for the nucleophilic dearomatization and/or functionalization of pyridines, quinolines, and isoquinolines covering the 2016–2022 period. Several general reviews on dearomatization reactions of electronically deficient heteroarenes have been published by Charette, You, Fan, Takemoto, Todd, and Sathiyanarayanan, especially until 2016.^13−18^ After this period, Bertuzzi, Bernardi, and Fochi published in 2018 a review regarding nucleophilic dearomatizations of activated pyridines exclusively.^19^ During the process of elaboration of this manuscript, Hu and Xia revised in early 2022 the metal-catalyzed nucleophilic dearomatization of electron-deficient heteroarenes.^20^ On the other hand, although hydrogenation reactions are common strategies to perform dearomatization of pyridines, quinolines, and isoquinolines, recent reviews from Glorius,^21^ Xiao,^22^ and Zhou^23^ contain these types of transformations and will be omitted in this review. Nevertheless, the research activity in this area is overwhelming, and a general revision of this topic from 2016 will be of interest for the organic and medicinal chemistry community.

The purpose of this review is to demonstrate the transformative capacity of dearomatization reactions of electronically deficient heteroarenes (pyridines, quinolines, and isoquinolines) to generate three-dimensional complexity from two-dimensional precursors with concomitant introduction of functionality. Since a wide variety of methodologies have been devised for the dearomatization of those heteroarenes, the organization of the present review intends to rationalize these strategies for a better understanding of the topic.

Section 2 deals with hydroboration and hydrosilylation reactions. These transformations are useful alternatives to hydrogenation, avoiding one of the main drawbacks, which is the overreduction of the ring. Instead, hydroboration and hydrosilylation reactions usually proceed under mild conditions and it is possible to achieve partial reductions with excellent regioselectivity. Several transition metal-catalyzed reactions have been established for the selective 1,2- and 1,4-hydrosilylation and hydroboration of heteroarenes.^24^ Good levels of selectivity have been reached depending on the nature of the metal. Additionally, some metal-free and organocatalytic variants have been devised lately.

Cycloaddition reactions involving pyridines, quinolines, and isoquinolines will be described in Section 3. The nucleophilicity of the nitrogen atom of these heterocycles has been used to generate a wide variety of dipoles such as pyridinium-, quinolinium-, and isoquinolinium ylides and imides, Huisgen 1,4-dipoles, or *N*-aromatic zwitterions, among others, which participate in dipolar cycloadditions with concomitant dearomatization of the heterocyclic skeleton. Additionally, these heterocycles can act themselves as good electron-deficient dienes in various inverse electron demand Diels–Alder reactions and also perform as good dienophiles and dipolarophiles with the appropriate dipoles. Advances in this field will be covered in this section.

Intramolecular cyclizations have also been employed to perform dearomative transformations of heteroarenes and several examples will be discussed in Section 4. These processes can be assumed in two general types. The first one takes advantage of the generation of highly electrophilic species, such as π-allyl complexes, from substituents attached to the starting heterocycle, by means of a metal-catalyzed process. These complexes undergo intramolecular nucleophilic addition of the heterocyclic nitrogen, usually leading to dearomatized products. On the other hand, the generation of nucleophilic species, such as enolates, from substituents attached to the heterocycle, usually evolve through intramolecular addition over the heteroaromatic ring, ending up with a final dearomatization event.

Birch-type reductions represent the method of choice to perform selective dearomatization reactions. However, they are nonconvenient processes from an operational point of view since they require liquid ammonia as solvent and alkali metals. This scenario dramatically changed with the advent of photoredox catalysis, which offers the possibility of generating radicals in a catalytic manner under mild reaction conditions. Regarding the application of this methodology to dearomatization reactions, several methodologies have been recently devised. The examples that exemplify this strategy will be commented in Section 5.

In Section 6, other dearomatizations of pyridines, quinolines, and isoquinolines via intermolecular reactions will be covered. The addition of carbon- and heteroatom-centered nucleophiles to the heteroarene, such as carbon nucleophiles, silyl enol ethers, cuprates, Grignard reagents, indoles, alkynes, sulfoxonium ylides, or Breslow intermediates, among others, will be evaluated. Additionally, dearomatizations where the heteroarene is initially acting as nucleophile, such as transfer hydrogenations coupled with addition of electrophiles, will also be included herein.

Finally, some examples of the application of dearomatization reactions of pyridines, quinolines, and isoquinolines to the synthesis of natural products will be covered in Section 7.

**Figure 1:**
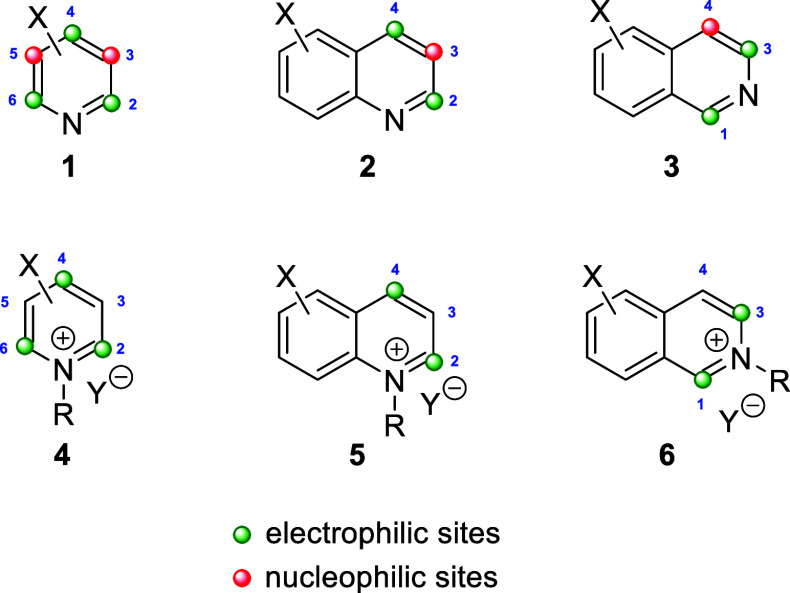
Reactive sites of pyridines, quinolines, isoquinolines, and their salts.

Theoretically, pyridines (**1**) and their salts (**4**) possess three electrophilic sites, namely the C2-, C4-, and C6-positions. Moreover, pyridines (**1**) hold two nucleophilic sites, at the C3- and C5-positions, besides the N atom. By the same token, quinolines (**2**) and isoquinolines (**3**), as well as their salts (**5** and **6**, respectively) hold two electrophilic sites (Figure 1).

Most examples of dearomative nucleophilic additions to these heterocycles deal with regioselective reactions through one of the electrophilic sites of the azaarenes (1,2- versus 1,4-dichotomy) and also with the functionalization of two reactive sites by addition of two nucleophiles or by means of cycloaddition reactions. However, all sites of the heterocycles are potentially reactive and the combination of those processes with subsequent transformations in a tandem fashion, involving multiple reaction sites, allows for the multifunctionalization of those heterocycles, thus increasing the molecular complexity from readily available starting materials in a very simple manner. Examples of the successful application of this principle have been devised lately.^25^

## 2 HYDROBORATION, HYDROSILYLATION, AND REDUCTION REACTIONS

Hydrogenation is the most straightforward method to obtain saturated nitrogen-containing heterocycles from heteroarenes. However, high pressures and/or elevated temperatures are usually required, which makes the chemoselective reduction a quite difficult task. In this context, hydroboration and hydrosilylation reactions have been extensively employed as convenient alternatives to hydrogenations. These transformations offer several advantages; namely, special equipment is not needed, reactions can be performed under milder conditions, variations of the electronic and steric properties of reagents can be tuned up by the substituents, and a silicon or boron moiety, suitable for further transformations, is introduced in the final products. Therefore, a handful of efficient and selective protocols, both metal-mediated and metal-free catalyzed, have been developed, providing access to a broad range of 1,2- and/or 1,4-dihydrogenated products. Additionally, when coupled with other reductive processes, a complete functionalization of the heteroarene can be achieved.

On the other hand, the use of borohydride derivatives is also a good alternative for the dearomatization of heteroarenes.^26,27^ New reducing methodologies, in combination with metal-catalyzed or metal-free processes, will also be revised in this section, which has been precisely divided in metal-catalyzed and metal-free processes.

### 2.1 Transition Metal-Catalyzed Hydroborations and Hydrosilylations

#### 2.1.1 Copper-Catalyzed Protocols

Direct dearomative reactions of *N*-heteroarenes, particularly by means of 1,2- or 1,4-nucleophilic additions, is not easily achieved under mild conditions due to the resonance stabilization of the *N*-aromatic core. In this context, transition metal-catalyzed processes have succeeded in the past decade in order to accomplish the dearomatization of pyridines, quinolines, and isoquinolines. Among the different metals used, Cu catalysts have received considerable attention due to their low cost, high earth abundance of copper, and environmentally benign nature.

**Scheme 1:**
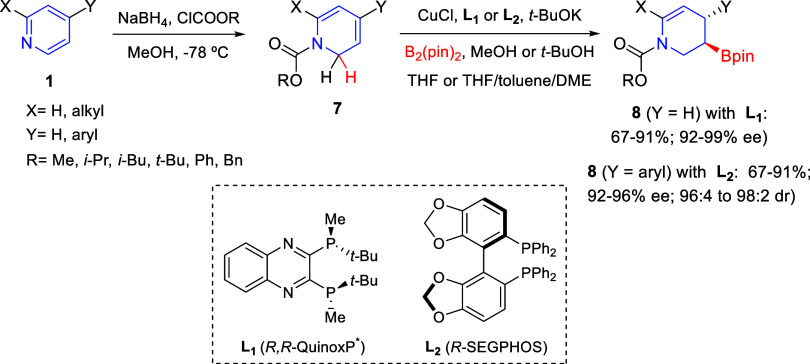
Stepwise Dearomatization/Enantioselective Cu(I)-Catalyzed Borylation of Pyridines

Ito and co-workers reported in 2016 an enantioselective method for the conversion of pyridines into chiral boryl-piperidines (Scheme 1).^28^ Initial attempts were performed on *N*-acylpyridinium salts under Cu(I) catalysis, although the 1,2-borylation products were unstable during purification. Instead, the authors developed a stepwise strategy involving a dearomative reduction followed by the Cu(I)-catalyzed enantioselective borylation. Thus, *N*-acyl 1,2-dihydropyridines **7** were generated through partial reduction of pyridinium salts, in turn prepared by reaction of substituted pyridines **1** with chloroformates, and were subjected to the Cu(I)-catalyzed protoborylation to render 3-boryl-tetrahydropyridines **8** in a regio-, diastereo-, and enantioselective manner. The reaction with monosubstituted 1,2-dihydropyridines **7** (Y = H) with bis(pinacolato)diboron in the presence of CuCl and the chiral phosphine **L**_**1**_ (*R*,*R*-QuinoxP*) led to products **8** bearing various carbamate-type protecting groups in good yields with excellent enantioselectivities. With 4-aryl-1,2-dihydropyridines **7** (X = H), the corresponding borylated products **8** bearing consecutive stereogenic centers were obtained with high diastereo- and enantioselectivities employing the phosphine ligand **L**_**2**_ (*R*-SegPhos) (Scheme 1). The authors also carried out several derivatizations of the boryl group and the enamine moiety in products **8**, providing access to chiral piperidines bearing a C-3 stereocenter, which are important scaffolds in various pharmaceutical drugs.

**Scheme 2:**
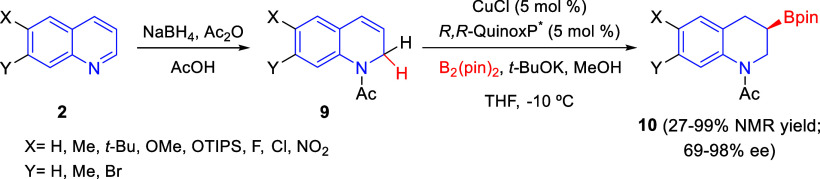
Copper(I)-Catalyzed Regio- and Enantioselective Protoborylation of 1,2-Dihydroquinolines

This dearomatization/enantioselective borylation sequence was further extended to quinoline derivatives **2**, which were transformed into 1,2-dihydroquinolines **9** to finally render enantiomerically enriched chiral 3-boryl-tetrahydroquinolines **10** (Scheme 2).^29^ The best results were obtained with the chiral phosphine ligand **L**_**1**_ (*R*,*R*-QuinoxP*). The borylation products were isolated in good yields as the corresponding silyl ethers arising from the sequential oxidation/silylation process. Regarding the scope of the process, it was compatible with quinolines with different electronic properties, although substrates bearing electron-withdrawing groups at the 6-position (X) provided moderate enantioselectivities. In addition, the borylation products were employed as versatile building blocks for the synthesis of several optically active tetrahydroquinolines bearing a C-3 stereocenter.

**Scheme 3:**
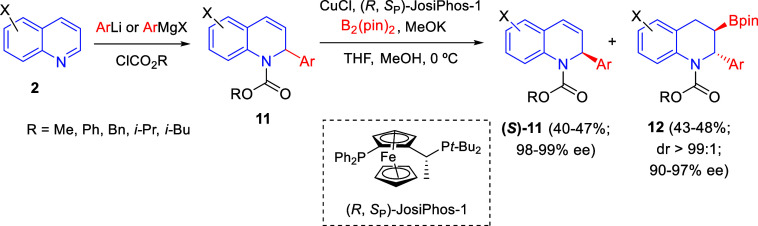
Kinetic Resolution of 2-Substituted 1,2-Dihydroquinolines by Asymmetric Cu-Catalyzed Borylation

A highly efficient asymmetric kinetic resolution of racemic 2-substituted 1,2-dihydroquinolines through an enantioselective Cu(I)-catalyzed borylation was reported by Hou and co-workers in 2017 (Scheme 3).^30^ The addition of aryl lithium or aryl Grignard reagents to quinolines **2** in the presence of chloroformates allowed the preparation of the dihydroquinoline substrates **11**, which were subjected to the optimized borylation conditions. This reaction took place with bis(pinacolato)diboron and the complex formed from CuCl and (*R*,*S*_p_)-Josiphos-1, in the presence of MeOK and MeOH as additives, affording simultaneously 2,3-disubstituted boryl-tetrahydroquinolines **12** as single diasteroisomers and the enantiomerically enriched recovered 1,2-dihydroquinolines (*S*)-**11**, both with excellent ee values. The process was highly efficient irrespective of the electronic properties and the carbamate moieties of the starting quinolines, with kinetic selectivity factors (*s*) up to 569.

**Scheme 4:**
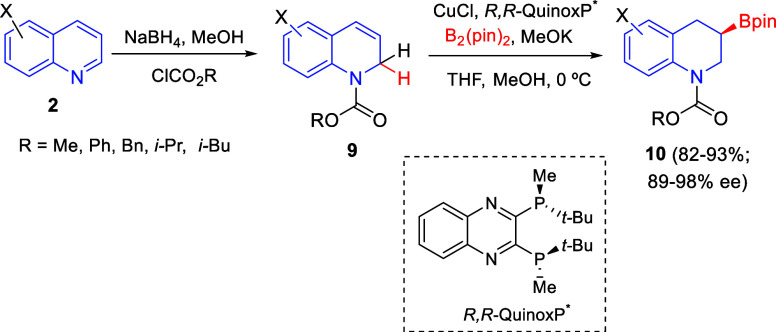
Cu-Catalyzed Regio- and Enantioselective Hydroboration of 1,2-Dihydroquinolines

An analogous Cu-catalyzed regio- and enantioselective hydroboration of 1,2-dihydroquinolines was also reported by Zhang and Hou in 2018 (Scheme 4).^31^ In this case, the Fowler reductive 1,2-dearomatization of quinolines **2** gave *N*-CO_2_R-protected 1,2-dihydroquinolines **9**, which were reacted with B_2_(pin)_2_ and CuCl in the presence of the chiral ligand *R*,*R*-QuinoxP* to render 3-boryl tetrahydropyridines **10** in good yields with up to 98% ee. 1,2-Dihydroquinolines **9** bearing various *N*-protecting groups were suitable substrates for the hydroboration reaction. Moreover, substituents on the phenyl ring had no relevant effect on the yield and enantioselectivity of the reaction, tolerating electron-donating and electron-withdrawing groups at the 6- or 7-positions of the quinoline ring. However, this catalytic system did not work for quinolines bearing a substituent at the 4-position.

**Scheme 5:**
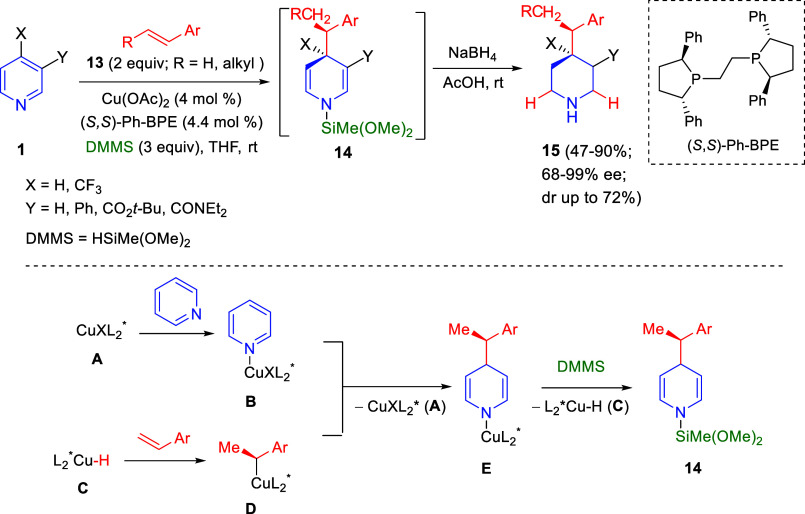
Asymmetric Cu(I)-Catalyzed Direct 1,4-Dearomatization of Pyridines

In 2018, Buchwald and co-workers reported a highly enantioselective nucleophilic 1,4-dearomatization of heteroarenes (Scheme 5). They demonstrated that a chiral copper hydride complex catalyzes the C–C bond-forming dearomatization of pyridines under mild conditions, without preactivation of the heterocycle nor preformation of the nucleophile.^32^ The reaction of pyridines **1** and aromatic olefins **13** with catalytic Cu(OAc)_2_ and the chiral ligand (*S*,*S*)-Ph-BPE, in the presence of dimethoxymethyl silane (DMMS) as a hydride source, gave rise to enantiomerically enriched 1,4-dihydropyridines **14** that were *in situ* reduced to piperidines **15** in good yields. This asymmetric dearomatization/reduction protocol was applied to a range of C3-substituted pyridines **1**, while substitution at the C4-position was only possible in special cases (X = CF_3_). Regarding the aryl alkene substituents in olefins **13**, the reaction was compatible with substituents at the C2- and C3-positions, while C4-substituents completely suppressed the dearomatization event.

A plausible mechanism for this Cu-catalyzed dearomatization reaction was proposed, according to which activation of the pyridine would occur through the formation of complex **B** with the copper salt **A**. At the same time, catalytically active species Cu-H (**C**), formed by reaction of the copper precatalyst with the silane, would generate an organocopper nucleophile **D** upon addition to the aryl alkene. Then, the activated pyridine complex **B** would undergo the nucleophilic dearomatization with the benzyl copper intermediate **D** and the resulting *N*-cuprated dihydropyridine **E** would deliver the *N*-silyl dihydropyridine **14** and regenerate the catalyst via σ-bond metathesis with the silane (Scheme 5).

The mechanism and the origin of the selectivity of this asymmetric 1,4-dearomatization reaction of pyridines with styrenes and dimethoxy(methyl)silane catalyzed by copper hydride complexes was theoretically studied by Buchwald^33^ and Sheong and Lin,^34^ independently.

**Scheme 6:**
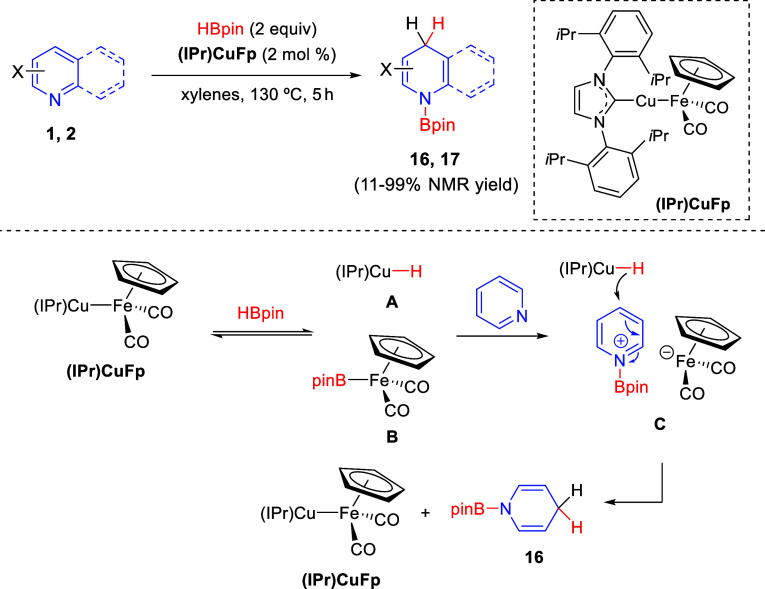
Regioselective Heterobinuclear Cu/Fe-Catalyzed 1,4-Hydroboration of Pyridines

In 2020, the group of Mankad described a regioselective 1,4-hydroboration of nonactivated pyridines **1** and quinolines **2** with pinacolborane by means of Cu/Fe heterobimetallic catalysis (Scheme 6).^35^ The process was efficiently catalyzed by the heterobinuclear complex (IPr)CuFp and provided access to valuable 1,4-dihydropyridine derivatives **16** and **17** in moderate to good yields. A variety of electronically different substituents were tolerated on the *meta*-position of the pyridine ring, albeit electron-donating ones gave poor 1,4- vs 1,2-regioselectivity. On the other hand, *ortho*- and *para*-substituted pyridines gave modest yields of the final products.

The authors investigated the enhanced catalytic activity and 1,4-regioselectivity observed with the heterobimetallic catalyst (IPr)CuFp compared to the mononuclear (IPr)CuO*t*Bu catalyst. The reaction of (IPr)CuFp with pinacolborane would generate the active catalyst (IPr)Cu-H (**A**) and FpBpin species **B** (Scheme 6). Then, pyridine would be activated through interaction with complex **B** and the resulting electrophilic pyridyl cation **C** would undergo nucleophilic 1,4-addition by (IPr)Cu-H in the presence of the reactive Fe site, rendering final products **16** and regenerating the (IPr)CuFp catalyst. In this manner, the bulky Bpin group at the nitrogen of the activated pyridine **C** would block the 2-position from the nucleophilic attack, favoring the reaction at the 4-position.

**Scheme 7:**
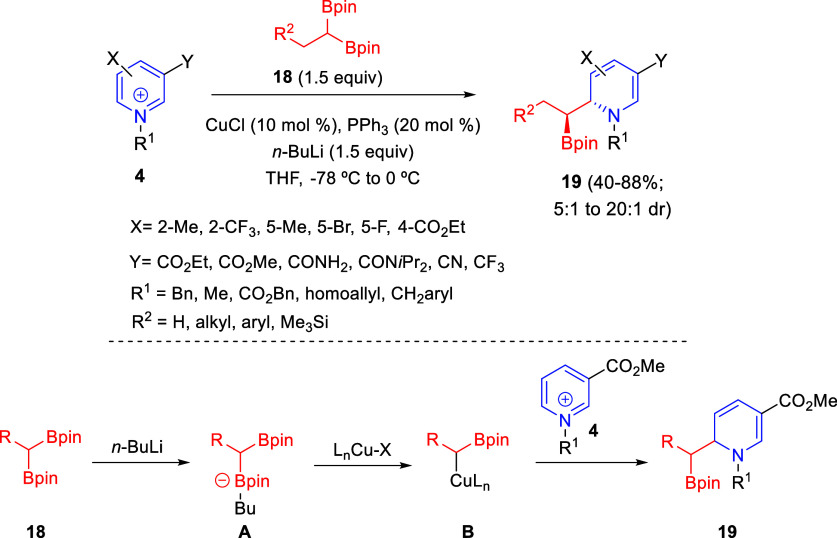
Copper-Catalyzed Regio- and Diastereoselective Reaction of Pyridinium Salts with Diborylalkyl Reagents

In 2021, Karimov and co-workers reported the 1,2-nucleophilic addition of diborylalkyl reagents to *N*-alkyl and *N*-acylpyridinium derivatives for the synthesis of nonaromatic *N*-heterocycles containing contiguous sterocenters (Scheme 7).^36^ Pyridinium salts **4** reacted with 1,1-diborylalkanes **18** in the presence of CuCl, Ph_3_P, and *n*-butyl lithium to deliver 1,2-dihydropyridines **19** in good isolated yields and diasterocontrol. A variety of benzyl- and heterobenzyl-substituted diborylmethane derivatives were tolerated in the process. Regarding the substitution at the pyridine ring, a range of electron-withdrawing groups were allowed at the C3-position, as well as electronically different groups at the C2-, C4-, and C5-positions. The process was also successfully extended to quinoline and isoquinoline salts. The authors also demonstrated that the dihydropyridine products could be transformed into tetrahydropyridine and piperidine derivatives.

This copper-catalyzed reaction would start with the generation of the boronate complex **A** by treatment of the diborylalkyl reagent **18** with *n*-BuLi. This boronate would undergo transmetalation with the Cu(I) complex to produce the nucleophilic species **B**, which would attack the pyridinium salt **4** to deliver product **19** and regenerate the Cu catalyst (Scheme 7).

**Scheme 8:**
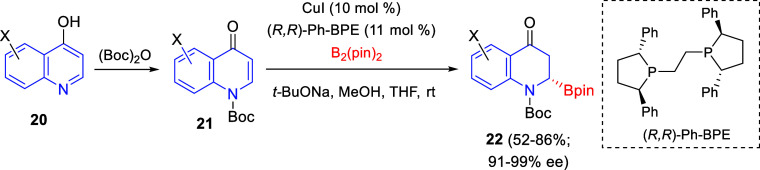
Copper-Catalyzed Enantioselective Dearomative Borylation of 4-Quinolinols

Very recently, Li and co-workers reported a regio- and enantioselective copper-catalyzed dearomative borylation of 4-quinolinols **21**,^37^ obtained through *N*-selective Boc-protection of 4-hydroxiquinolines **20** (Scheme 8). Treatment of a variety of *N*-Boc protected 4-quinolones **21**, with bis(pinacolato)diboron in the presence of CuCl as the catalyst, (*R*,*R*)-Ph-BPE as a chiral ligand, *t*-BuONa as a base, and MeOH as the proton source, led to cyclic α-aminoboronates **22** in good yields with excellent enantioselectivities.

#### 2.1.2 Nickel-Catalyzed Protocols

The field of nickel-catalysis has experienced a great advance in the past decade. This metal lies just above palladium in the group 10 of the periodic table. A relevant difference between these two transition metals is that nickel is a nonprecious metal and this is one of the reasons for the recent attention garnered to this metal. Moreover, several properties of nickel, such as its facile oxidative addition and ready access to multiple oxidation states, accounts for the numerous and diverse nickel-catalyzed reactions reported in the literature in recent years.^38^ Homogeneous nickel catalysis has also reached the field of heteroaromatic dearomatization.

**Scheme 9:**
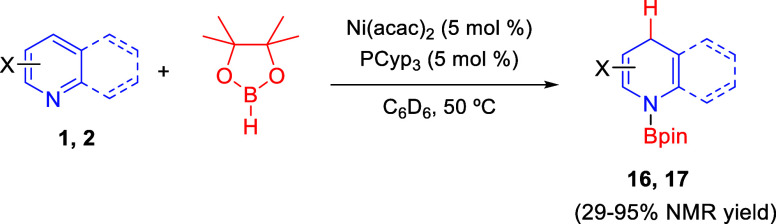
Nickel-Catalyzed Regioselective 1,4-Hydroboration of Pyridines

In 2018, Findlater and co-workers found that Ni(acac)_2_, in combination with tricyclopentyl phosphine (PCyp_3_), catalyzes the regioselective 1,4-hydroboration of pyridines **1** with pinacol borane, affording *N*-borylated 1,4-dihydropyridines **16** in generally good ^1^H NMR yields and regioselectivities (Scheme 9).^39^ The best results were obtained with pyridines substituted with electronically different functional groups at the C4-position. Substituents at the C3-position were tolerated, albeit with a clear drop in regioselectivity, while substituents at the C2-position produced very low yields of the final products. The process also showed good catalytic activity in the 1,4-hydroboration of quinolines **2**. The authors performed studies to determine the origin of the regioselectivity. Although the mechanism is not clear, it seems that two pyridine rings are incorporated at the nickel coordination sphere, activating the pyridine ring and, at the same time, blocking the 2-position.

**Scheme 10:**
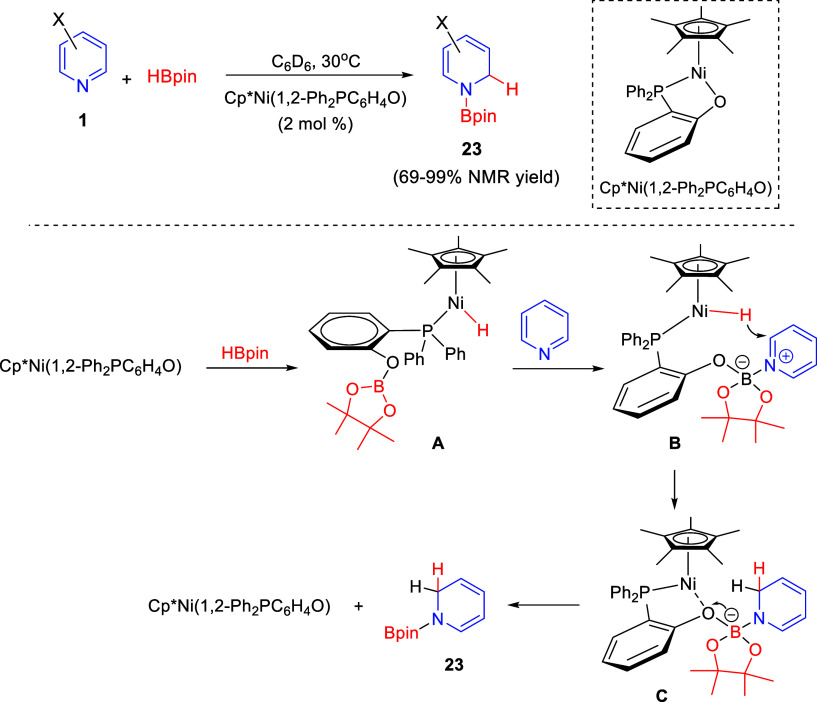
1,2-Hydroboration of Pyridines Catalyzed by a Cooperative Ni–O Catalyst

The groups of Liao and Wang designed and synthesized an air-stable half-sandwich nickel(II) complex bearing a phosphinophenolato ligand for the catalytic 1,2-hydroboration of pyridines **1** to form 1,2-dihydropyridines **23** (Scheme 10).^40^ DFT calculations allowed the authors to propose that activation of HBpin by the catalyst would generate a nickel hydride complex **A**. After addition of pyridine, a borate complex **B** would be formed, with subsequent hydride transfer from the nickel center to the *ortho*-position of the pyridine ring, to render intermediate **C**. Final cleavage of the O–B bond would regenerate the starting nickel species and release the 1,2-dihydropyridine **23** (Scheme 10). Several substituents at the 4-position of the pyridine ring were tolerated, independently of their electronic nature, while substitution at the 3-position resulted in an important loss of regioselectivity. The process was further extended to quinolines and isoquinolines.

#### 2.1.3 Ruthenium-Catalyzed Protocols

**Scheme 11:**
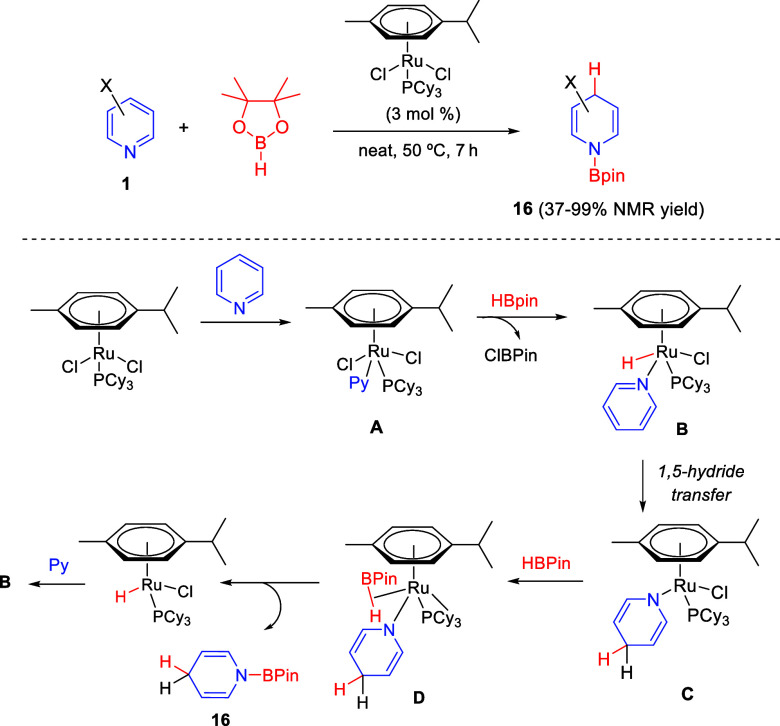
Ruthenium-Catalyzed Regioselective 1,4-Hydroboration of Pyridines

Ruthenium complexes have also been employed as effective catalysts for the regioselective 1,4-dearomatization of pyridines with pinacol boranes.^41^ In this context, [Ru(*p*-cymene)P(Cy)_3_Cl_2_] was found to be a suitable catalyst for the synthesis of *N*-boryl-1,4-dihydropyridines **16** (Scheme 11). A wide range of substituents at the 3-position of the pyridine ring were tolerated in this reaction, while 2-substituted pyridines did not undergo hydroboration, probably due to steric hindrance.

The authors also proposed a plausible catalytic cycle for this reaction on the basis of several control experiments. Ru complex [Ru(*p*-cymene)P(Cy)_3_Cl_2_] would react with pyridine to afford intermediate **A** that, upon reaction with HBpin, would generate the Ru-H complex **B**, responsible for catalysis. Then, the 1,5-hydride transfer, which would prevail over the 1,3-transfer (probably due to steric requirements), would give rise to intermediate **C**. Finally, coordination with HBpin and transmetalation would release the 1,4-dihydropyridine **16**, rendering Ru complex **E** that, after subsequent coordination of pyridine, would regenerate the catalytic species **B**, closing the catalytic cycle (Scheme 11).

**Scheme 12:**
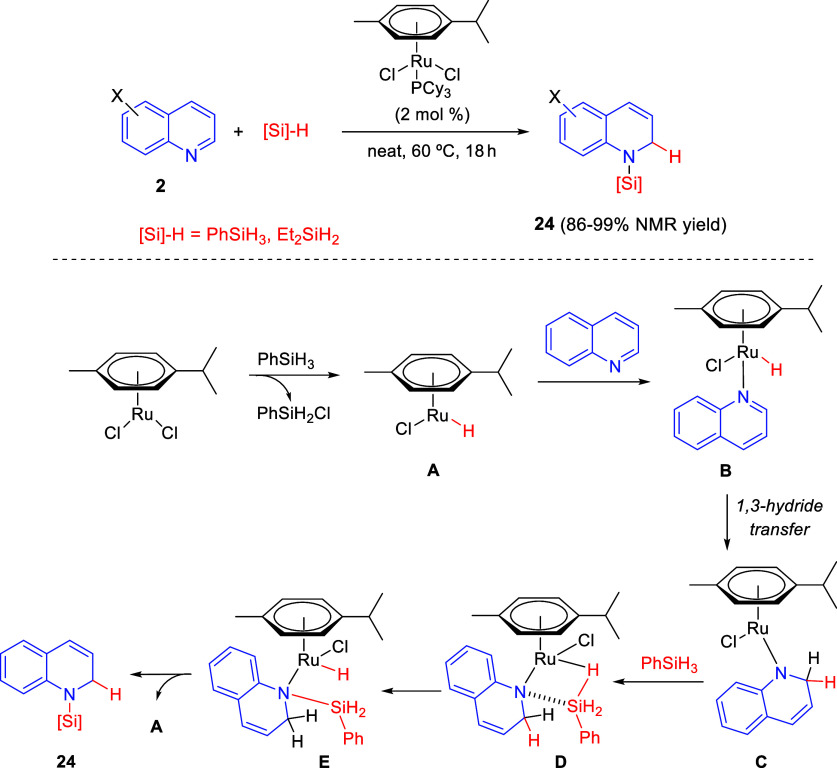
Ruthenium-Catalyzed Regioselective 1,2-Hydrosilylation of Quinolines

The same ruthenium precatalyst, [Ru(*p*-cymene)P(Cy)_3_Cl_2_], was effective in the regioselective 1,2-hydrosilylation of quinolines **2** with phenylsilane and diethylsilane, to afford *N*-silyl 1,2-dihydroquinolines **24** in good to excellent yields (Scheme 12).^42^ Interestingly, this reaction proceeded with reverse regioselectivity compared to the use of HBpin as the dearomatizing nucleophile. Both electron-donating and electron-withdrawing groups at several positions of the starting quinolines were tolerated in this protocol, which also was extended to pyridines, isoquinolines, and other *N*-heteroarenes.

Control experiments and DFT calculations were performed in order to investigate the reaction mechanism. Accordingly, the process would be initiated by dissociation of PCy_3_ from the Ru precatalyst and further reaction with the silane to generate the catalytically active ruthenium hydride **A** by dissociation of chlorophenylsilane. After coordination with the *N*-heteroarene, the resulting intermediate **B** would undergo intramolecular 1,3-hydride transfer to the *ortho*-position of the quinoline ring to render Ru-amide intermediate **C**. Then, the reaction with the silane via square intermediate **D** and subsequent σ-metathesis would result in the transfer of the hydride to ruthenium. Finally, dissociation of the product from intermediate **E** would simultaneously regenerate the catalytically active species **A** (Scheme 12). The authors concluded that, in the previously reported ruthenium-catalyzed hydroboration of pyridines (see Scheme 11), the steric effects exerted by methyl groups on pinacolborane and the *p*-cymene ligand would be responsible for the 1,4-selectivity observed in the hydroboration reaction.

#### 2.1.4 Zinc-Catalyzed Protocols

**Scheme 13:**
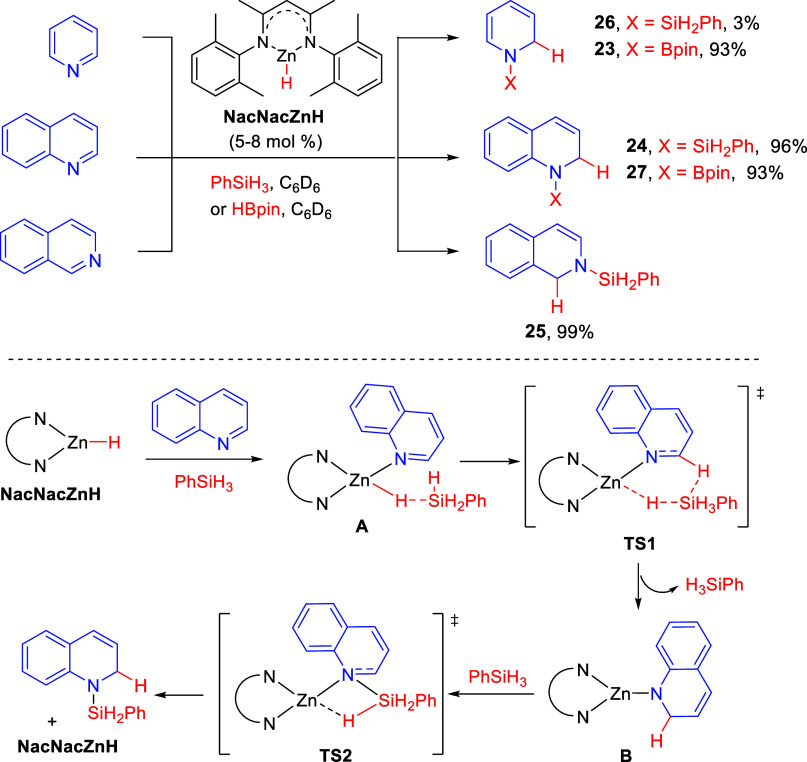
Zinc-Catalyzed 1,2-Hydrosilylation and Hydroboration of *N*-Heterocycles

Nikonov and co-workers found that zinc hydride NacNacZnH (NacNac = [Ar′NC(Me)CHC(Me)NAr′]–, Ar′ = 2,6-Me_2_C_6_H_3_) catalyzes regioselective 1,2-hydrosilylation and 1,2-hydroboration reactions of several heterocycles, including pyridine, quinoline and isoquinoline (Scheme 13).^43^ In this manner, the usual transition-metal catalysts were replaced by more abundant and less toxic zinc derivatives. Hydrosilylation took place with unsubstituted quinoline and isoquinoline to afford the corresponding silyl derivatives **24** and **25**, respectively, in excellent yields; however, pyridine was almost unreactive under these conditions (**26**: 3% NMR yield). On the other hand, hydroboration reactions with pyridine and unsubstituted quinoline gave the corresponding dihydro-derivatives **23** and **27**, respectively, in excellent ^1^H NMR yields (Scheme 13). Methyl substitution at the 2-position of quinolines resulted in a clear drop of the chemical yield.

DFT studies led the authors to propose that the reaction would preferably proceed by initial complexation of the Zn catalyst with quinoline and silane, forming complex **A**. A concerted six-membered transition state **TS1** stemming from silane attack would produce the 1,2-addition on the quinoline ring. Then, complex **B** would react with another equivalent of silane to render, through **TS2**, the final dihydroquinoline product (Scheme 13).

**Scheme 14:**
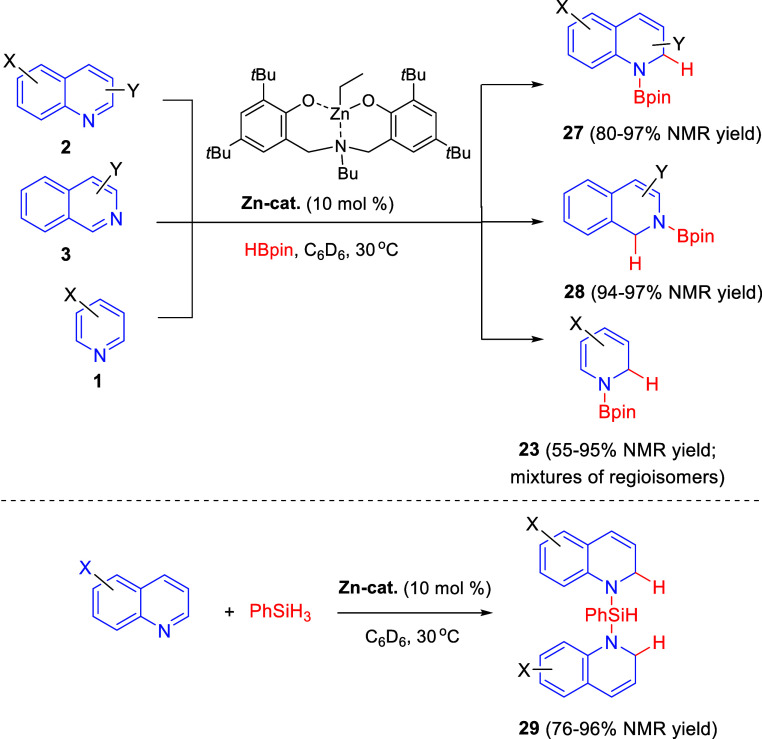
Regioselective Hydroboration and Hydrosilylation of *N*-Heteroarenes Catalyzed by a Zinc Alkyl Complex

Yao, Yuan, and co-workers reported in 2020 the use of readily available zinc alkyl complexes as catalysts for the regioselective hydroboration of *N*-heteroarenes (Scheme 14).^44^ The combination of ZnEt_2_ with multidentate phenolate ligands allowed the authors to identify a suitable catalyst that converted quinolines **2** bearing either electron-donating or electron-withdrawing groups at C3–C7-positions into 1,2-dihydroquinolines **27** in very good yields. Isoquinolines **3** also reacted under the same conditions to afford the corresponding 1,2-hydroboration products **28**. Regarding pyridines, mixtures of regioisomers were obtained, with 1,2-dihydropyridines **23** as the main products. This catalytic system was also extended to the addition of phenyl silane to quinolines, which gave rise to the bis-hydrosilylation products **29** in good yields (Scheme 14).

#### 2.1.5 Cobalt-Catalyzed Protocols

**Scheme 15:**
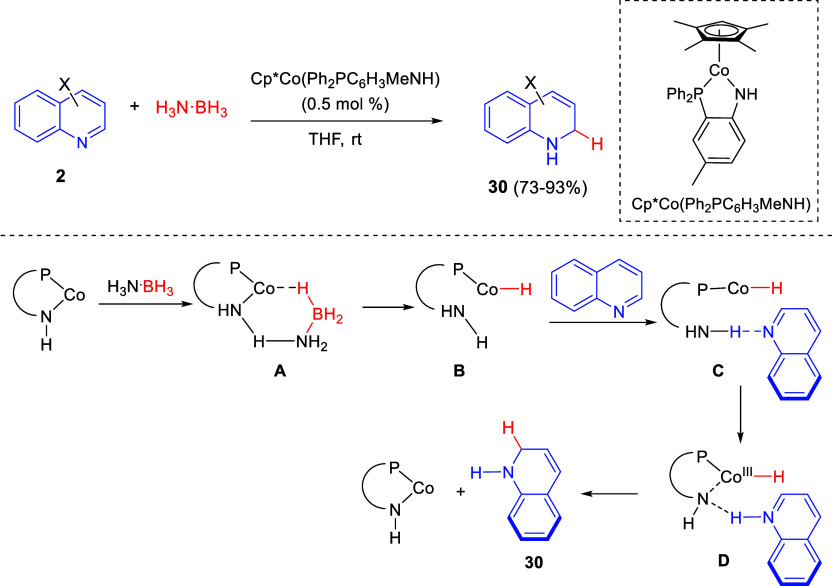
Partial Transfer Hydrogenation of Quinolines through Cobalt-Amido Cooperation with Ammonia Borane

In 2020 Wang, Liao, and co-workers disclosed a cobalt-amido cooperative catalyst for the efficient 1,2-hydroboration of quinolines (Scheme 15).^45^ Thus, the cobalt(II) complex Cp*Co(Ph_2_PC_6_H_3_MeNH) catalyzed a highly regioselective addition of ammonia borane (H_3_N·BH_3_) to quinolines **2** at room temperature to render 1,2-dihydroquinoline derivatives **30** in good isolated yields. The functional group tolerance of this catalytic system was exceedingly broad and a wide variety of substituents, including ester, amide, and alkenyl groups, among others, were allowed at the C3–C7-positions of the quinoline ring, regardless their electronic nature.

DFT calculations led the authors to propose a mechanism for this catalytic partial transfer hydrogenation of quinolines. Initially, the cobalt-amido complex would activate H_3_N·BH_3_ in a concerted proton transfer/hydride transfer reaction through intermediate **A** to render cobalt(II)-hydride species **B**. Subsequent complexation of quinoline would result in complex **C**, which would undergo proton transfer from the amino group of the ligand to the N atom of quinoline with electron transfer from the Co(II) center to the quinoline moiety. Finally, the amido site, through H-bonding interaction with the N atom in intermediate **D**, would assist the hydrogen transfer from Co(III)-H to the 2-position of the quinoline ring, rendering product **34** and releasing the cobalt catalyst (Scheme 15).

**Scheme 16:**
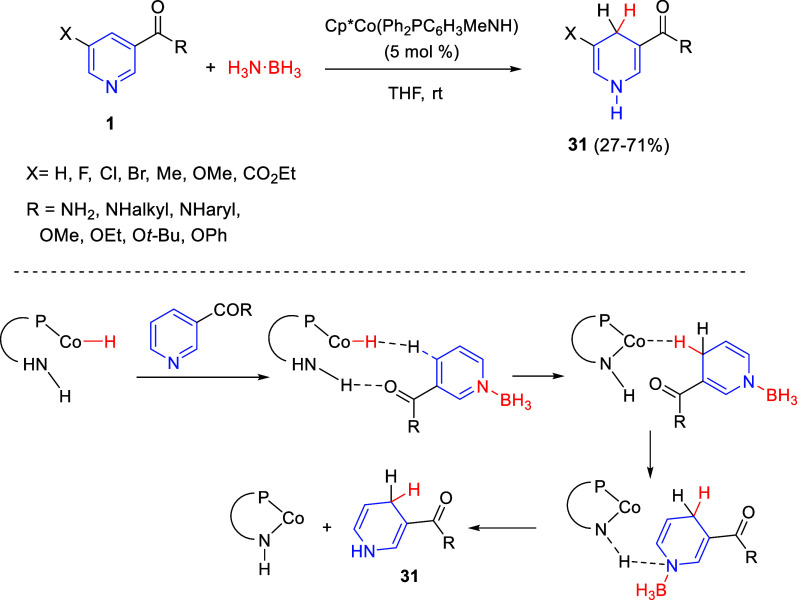
Cobalt-Catalyzed Selective 1,4-Dearomatization of Pyridines

Two years later, the same authors applied the cobalt-amido cooperative catalysis to the 1,4-dearomatization of pyridines (Scheme 16).^46^ Thus, the selective transfer hydrogenation of nicotinic acid derivatives **1** with ammonia borane as the dihydrogen source was achieved with the cobalt complex Cp*Co(Ph_2_PC_6_H_3_MeNH), furnishing 1,4-dihydropyridine derivatives **31** in moderate to good isolated yields. This reaction was compatible with differently substituted nicotinamides and nicotinates; however, substitution at C2 and C6 was not tolerated.

In order to explain the change of regioselectivity observed in the dearomatization of pyridines to 1,4-dihydropyridines, when compared to the previously developed reaction with quinolines (see Scheme 15), DFT calculations showed that, in this case, a hydrogen bond between the carbonyl group in the substrate and the amino site of the Co-H complex would direct the hydride transfer from the cobalt center to the 4-position of the pyridine ring (Scheme 16).

#### 2.1.6 Protocols Catalyzed by Other Metals

**Scheme 17:**
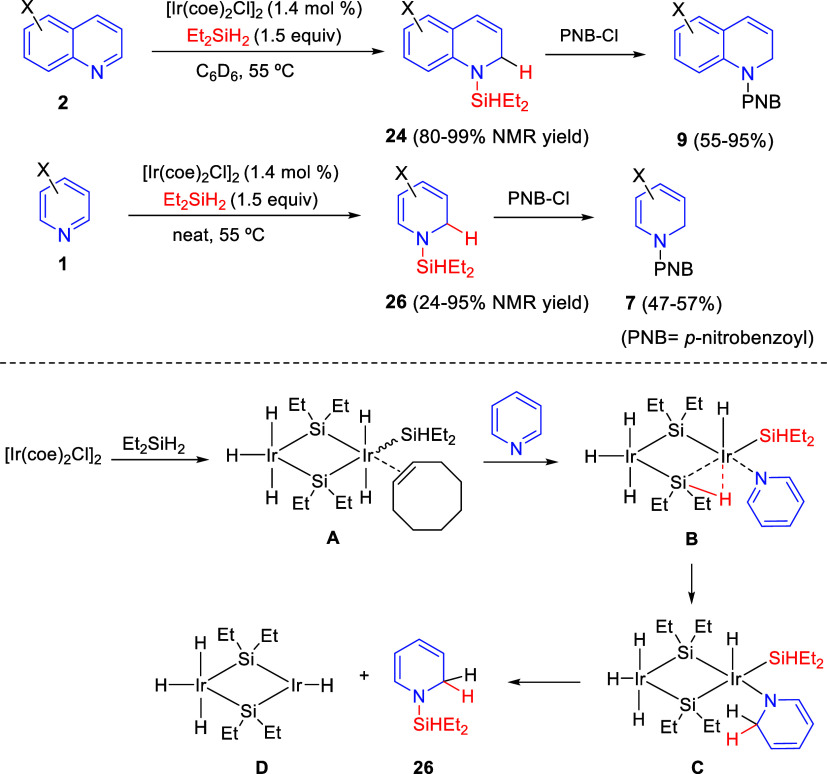
Ir-Catalyzed Dearomative 1,2-Hydrosilylation of Quinolines and Pyridines

Chan, Park, and co-workers reported in 2016 the hydrosilylation reaction of quinolines and pyridines catalyzed by a silylene-bridged Ir dimer, generated *in situ* from chlorobis(cyclooctene)iridium dimer, [Ir(coe)_2_Cl]_2_, and diethylsilane (Scheme 17).^47^ The hydrosilylative dearomatization took place without the need of other additives and rendered 1,2-dihydroquinolines **24** and 1,2-dihydropyridines **26** in good yields with high regioselectivity. Since several *N*-silylated products were unstable, they were subjected to *N*-benzoylation, furnishing the corresponding protected dearomatized products in good isolated yields over two steps.

On the basis of mechanistic studies in combination with the literature precedent, the authors proposed that, initially, [Ir(coe)_2_Cl]_2_ would react with the silane to generate a bis-iridium adduct **A**. Then, the ligand exchange with the heteroarene substrate would form species **B**, which would undergo the key hydride insertion to render intermediate **C**. Reductive elimination would release the final 1,2-hydrosilylated product and regenerate the Ir-H complex **D** (Scheme 17).

**Scheme 18:**
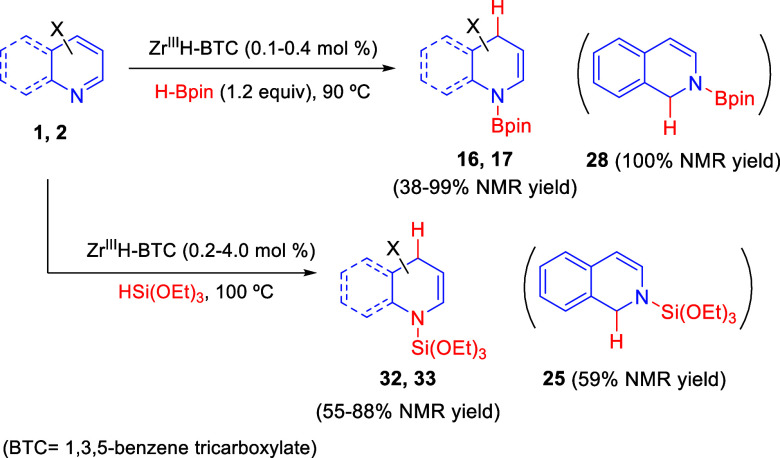
Zr^III^H-BTC-Catalyzed 1,4-Dearomative Hydroboration and Hydrosilylation of Pyridines and Quinolines

Lin and co-workers found that Zr^III^H-BTC (BTC = 1,3,5-benxene tricarboxylate) metal organic frameworks (MOFs) catalyze the regioselective 1,4-hydroboration and 1,4-hydrosilylation of pyridines and quinolones (Scheme 18).^48^ This MOF was generated from Zr^IV^Cl_2_-BTC by simple reduction with NaBEt_3_H and contained the zirconium hydride necessary to effect the reaction. Heating a mixture of pyridines **1** or quinolines **2** and HBpin or SiH(OEt)_2_ in the presence of very low catalyst loadings of Zr^III^H-BTC afforded the desired 1,4-addition products **16/17** and **32/33** in very good ^1^H NMR yields. Pyridines bearing substituents at C3 and C5 were tested in the hydroboration reaction, as well as quinolines substituted at C8, either with electron-donating or electron-withdrawing substituents. On the other hand, the hydroboration and hydrosilylation of isoquinoline gave exclusively *N*-substituted 1,2-dihydroisoquinolines **28** and **25** (Scheme 18).

**Scheme 19:**
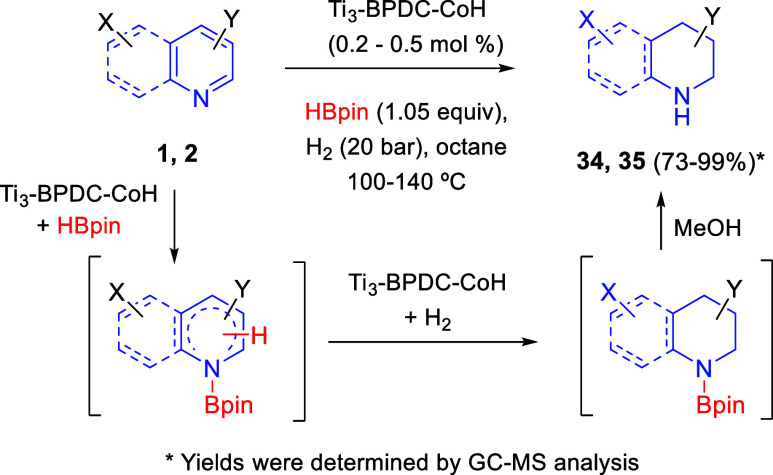
Ti_3_-BPDC-CoH-Catalyzed Cascade Reduction of Pyridines and Quinolines

Also in the field of heterogeneous catalysis, in 2019 Lin and co-workers reported a porous Ti-carboxylate MOF made of biphenyl-4,4′-dicarboxylate (BPDC) linkers and Ti_3_(OH)_2_ secondary building units (SBUs) (Scheme 19).^49^ Each pair of Ti^IV^-OH groups of neighboring SBUs deprotonated and acted as bidentate ligands to support Co^II^-hydride species, able to promote a cascade reduction of *N*-heterocyclic rings such as pyridines **1** and quinolines **2** via sequential dearomative hydroboration and hydrogenation. In this manner, piperidines **34** and tetrahydroquinolines **35** were obtained in high to quantitative yields, good functional group tolerance and outstanding chemoselectivity. Ti_3_-BPDC-CoH also demonstrated good recovery and reusability: at least 6-fold without a significant decrease in yields. The cascade reduction pathway would start with a dearomative hydroboration with pinacolborane (HBpin) followed by hydrogenation of the remaining double bonds, both steps being catalyzed by the Co-H centers (Scheme 19).

**Scheme 20:**
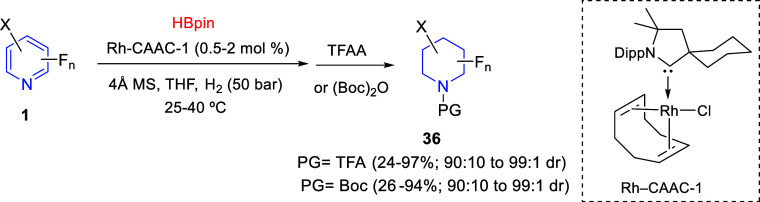
One-Pot Rhodium-Catalyzed Dearomative Hydroboration/Hydrogenation of Fluorinated Pyridines

The group of Glorius developed in 2019 a one-pot rhodium-catalyzed dearomative hydroboration/hydrogenation of fluorinated pyridines, which provided access to *cis*-fluorinated piperidines (Scheme 20).^50^ The reaction of mono-, di-, and trisubstituted fluoropyridines **1** with pinacol borane (HBpin) and rhodium cyclic aminoalkyl carbene Rh-CAAC-1 in the presence of hydrogen, followed by treatment with trifluoroacetic anhydride (TFAA) or (Boc)_2_O, delivered all-*cis*-(multi)fluorinated piperidines **36** in generally good yields and excellent diastereomeric ratios. A wide variety of substituents at the pyridine ring were compatible with the process, although fluorine atoms were always accompanied by electron-donating substituents. The process was also efficiently extended to quinolines and isoquinolines, with complete hydrogenation of the heterocyclic side.

**Scheme 21:**
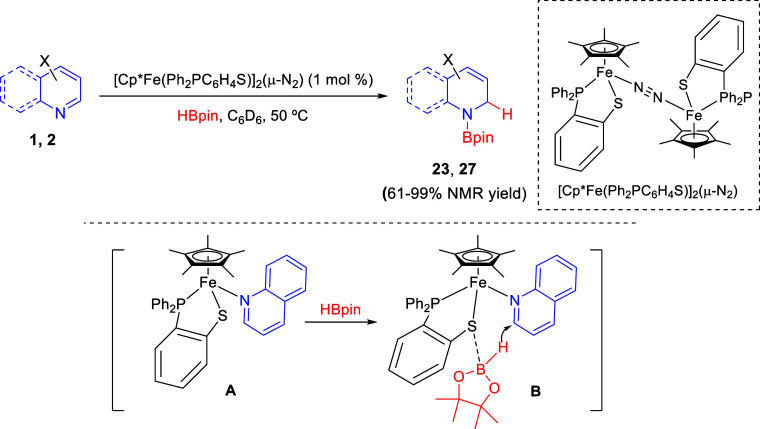
Iron-Catalyzed 1,2-Selective Hydroboration of *N*-Heteroarenes

Wang and co-workers employed the N_2_-bridged diiron complex [Cp*Fe(Ph_2_PC_6_H_4_S)]_2_(μ-N_2_) to catalyze the 1,2-hydroboration of pyridines and quinolines with pinacolborane (Scheme 21).^51^ The process took place in benzene and afforded the corresponding *N*-borylated dihydropyridines **23** and dihydroquinolines **27** in generally good yields. Pyridines with both electron-donating and electron-withdrawing groups underwent the hydroboration efficiently, and the process was also extended to isoquinolines.

This catalytic reaction would involve coordination of the heteroarene to the iron center, replacing the N_2_ ligand, to generate mononuclear iron(II) complex **A**. Unlike other metals, the process did not start with the activation of the B–H bond. In addition, it was not possible to detect Fe(II)-H species by NMR studies. However, the sulfur atom of the iron complex would play a crucial role through coordination to the boron atom to directly deliver the hydrogen to the heterocycle, as indicated in intermediate **B** (Scheme 21).

**Scheme 22:**
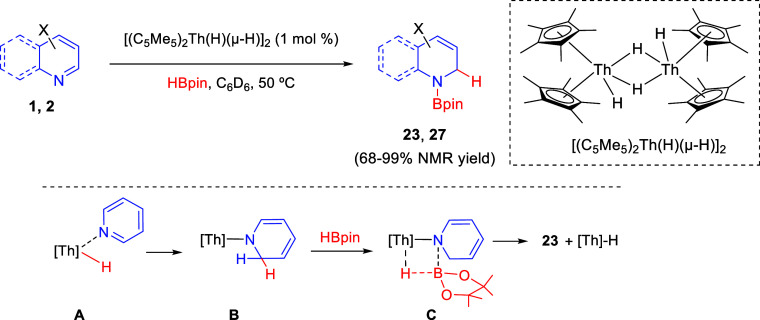
Thorium-Catalyzed 1,2-Selective Hydroboration of *N*-Heteroarenes

One year later, the same transformation was described by Eisen and co-workers, employing the thorium hydride complex [(C_5_Me_5_)_2_Th(H)(μ-H)]_2_, which catalyzed the reaction of pyridines and quinolines with pinacolborane to afford the corresponding 1,2-dearomatized products **23** and **27** in moderate to good yields (Scheme 22).^52^ A wide range of pyridines with different groups and electronic properties were tolerated, although strong electron-donating groups such as NMe_2_, as well as the presence of substituents at the C2-position, were not compatible with the reaction. Quinolines were also good partners for this protocol, even those substituted at the C8-position.

The reaction would involve the insertion of the Th-H moiety into the C=N bond of the coordinated pyridine to generate the thorium dihydropyridine **B**. In the presence of HBpin, a subsequent Th-N/H-B σ-bond metathesis through transition state **C** would produce the product and simultaneously regenerate the catalytically active species (Scheme 22).

**Scheme 23:**
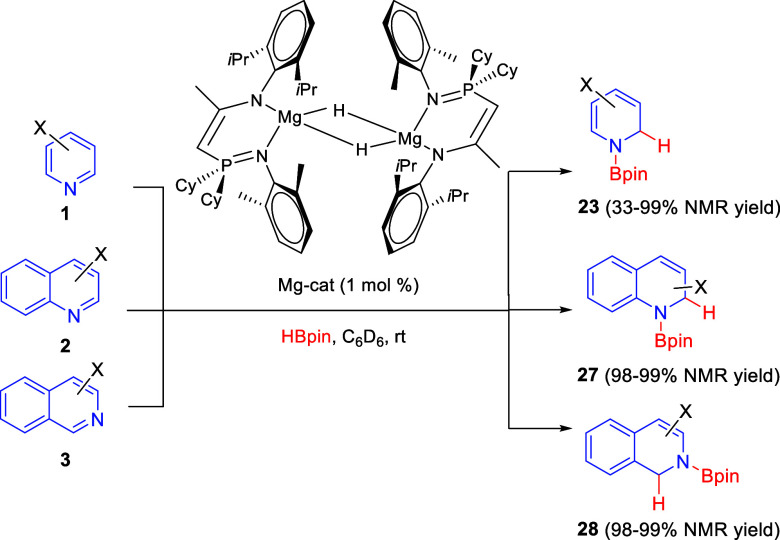
Magnesium-Catalyzed Regioselective 1,2-Hydroboration of *N*-Heteroarenes

Finally, in 2020 Cui and co-workers developed a regioselective 1,2-hydroboration reaction of pyridine derivatives with HBpin as the boron source catalyzed by magnesium complexes ligated by phosphinimino amides (Scheme 23).^53^ Pyridines **1** bearing electronically different substituents at the C3- and C4-positions were compatible with the process, albeit electron-withdrawing groups provided better yields. The process was initially developed for pyridines and extended to quinolines **2** and isoquinolines **3** with comparable efficiency. DFT calculations were performed in order to rationalize the regioselectivity observed, concluding that the transition state for the 1,4-addition is 4 kcal/mol higher in energy than that for the 1,2-addition, which is in accordance with experimental results.

### 2.2 Metal-Free Hydroborations, Hydrosilylations, and Reductive Protocols

#### 2.2.1 Hydride Addition to Pyridinium, Quinolinium, and Isoquinolinium Salts

**Scheme 24:**
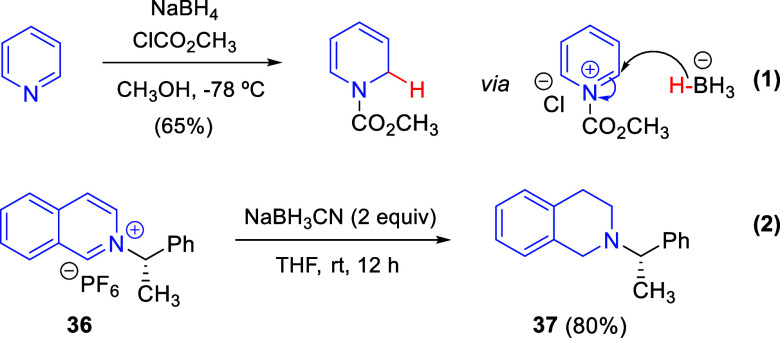
Reductive Dearomatization of Pyridines and Isoquinolinium Salts

One of the oldest metal-free strategies to dearomatize pyridines and quinolines to their corresponding 1,2-dihydro-derivatives was developed by Fowler in 1972.^54^ In that work, *N*-carbomethoxy-1,2-dihydropyridine was produced by treating pyridine with sodium borohydride and methyl chloroformate in methanol at −78 °C (Scheme 24, eq 1). This reaction proceeded through the initial formation of the *N*-carbomethoxypyridinium salt followed by hydride reduction of the electron-deficient pyridinium ring at C2.

Hurvois and co-workers employed a similar strategy to reduce the chiral hexafluorophosphate isoquinolinium salt **36** to the tetrahydroisoquinoline **37** in the presence of 2 equiv of NaBH_3_CN in THF at room temperature (Scheme 24, eq 2).^55^ This tetrahydro-derivative was employed as a building block for the synthesis of chiral *N*-Boc-1-alkyl-tetrahydroquinolines.

**Scheme 25:**
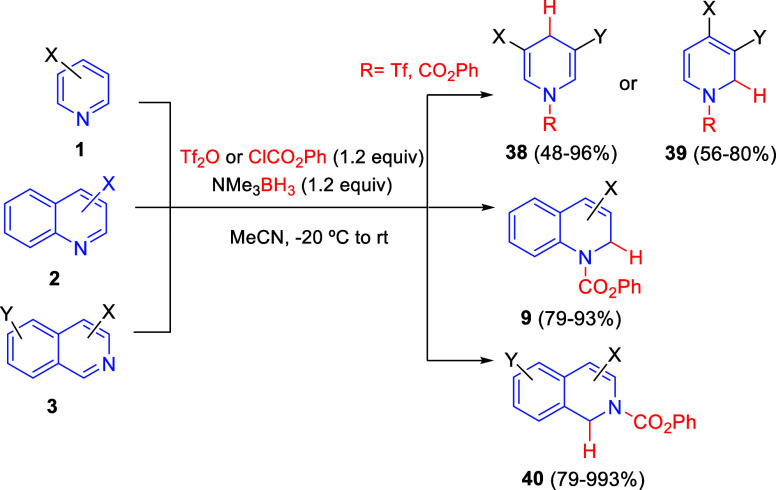
Dearomatization of Pyridines, Quinolines, and Isoquinolines with Trimethylamine Borane as Mild Reducing Agent

In 2021, Glorius and co-workers designed a modification of Fowler’s 1,2-reductive dearomatization of pyridines by replacing sodium borohydride with trimethylamine borane as the hydride donor, and using phenyl chloroformate or triflic anhydride as the activating reagent in acetonitrile (Scheme 25).^56^ A broad range of 3- and 3,5-substituted pyridines **1** were converted into the corresponding 1,4-dihydropyridines **38** in high yields and very good selectivities, meaning a reversal of the regioselectivity obtained with the Fowler reduction. However, it was found that the presence of substituents at the 4-position inverted the selectivity to yield 1,2-dihydropyridines **39**. Likewise, the reaction with quinolines **2** and isoquinolines **3** also afforded the 1,2-dearomatized products **9** and **40**, respectively (Scheme 28). This methodology did not require anhydrous solvents or inert atmosphere and it was compatible with a wide variety of substituents, including easily reducible groups such as boronic esters, aldehydes, nitro, or nitrile groups.

**Scheme 26:**
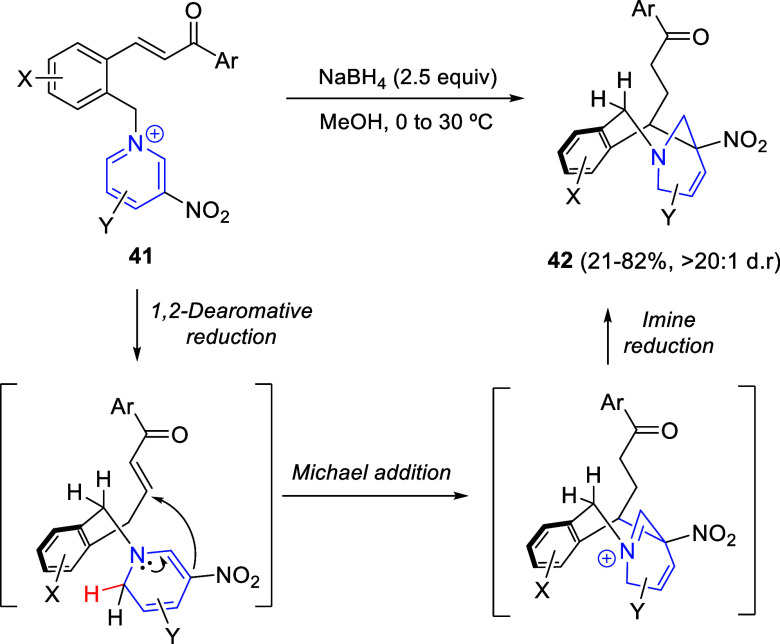
Interrupted Dearomative Reduction Strategy of Chalcone-Derived Pyridinium Salts

Dihydropyridines and their derivatives are useful building blocks; however, they are usually not very stable, which makes their purification and isolation difficult. For this reason, they are often used immediately after their synthesis. A solution to this problem is to couple Fowler’s 1,2-reductive dearomatization to other reactions in cascade sequences, as reported very recently by Xu, Wang, and co-workers (Scheme 26). These authors described the transformation of planar chalcone-derived pyridinium salts **41** into bridged piperidine derivatives **42**, with complete regio- and diastereocontrol, by using NaBH_4_ in methanol under mild conditions.^57^ This interrupted dearomative reduction strategy consisted of three sequential reactions; namely, 1,2-dearomative reduction/Michael addition/imine reduction, which was supported by control experiments employing NaBD_4_ as the reductant. A variety of chalcone-based pyridinium salts bearing substituents at various positions with different electronic properties were tolerated in this transformation. It is worth mentioning that the nitro group at the C3-position of the pyridinium ring was crucial to ensure the subsequent Michael addition. This was confirmed by replacing the nitro group with methyl or ester substituents, or by using a chalcone-based quinolinium or isoquinolinium salt instead.

**Scheme 27:**
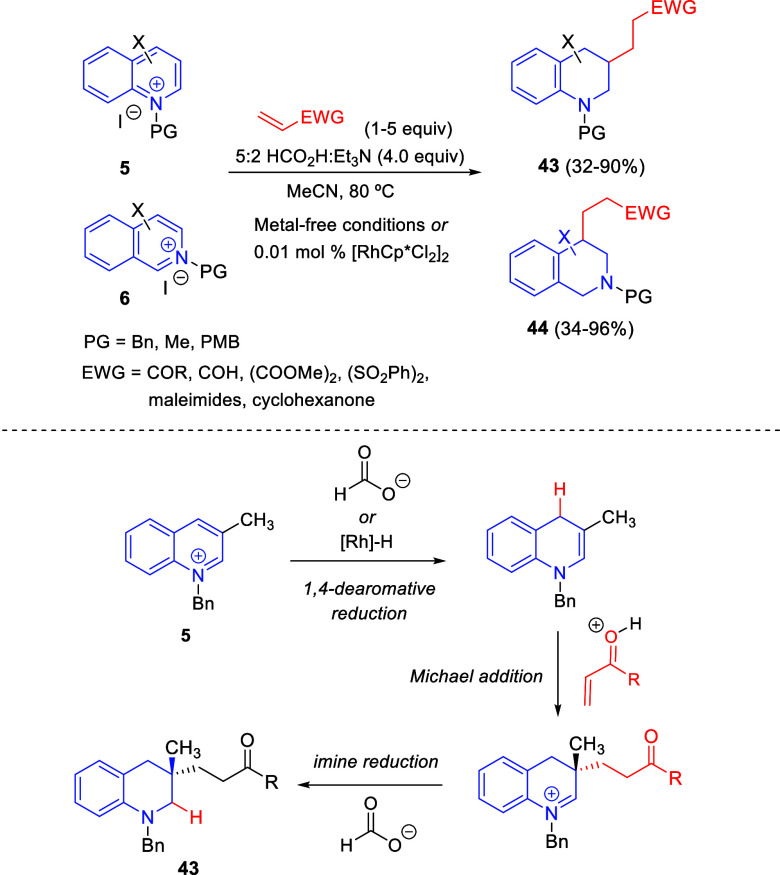
Reductive Functionalization of Quinolinium and Isoquinolinium Salts with Michael Acceptors

The 1,2-dearomative reduction/conjugate addition/imine reduction cascade sequence was also reported by Donohoe and co-workers for the reductive functionalization of quinolinium (**5**) and isoquinolinium (**6**) salts with different unsaturated electrophiles (Scheme 27).^58^ Inexpensive formic acid was used as the terminal reductant, although its acidity had to be attenuated by using Et_3_N in order to minimize undesired reaction pathways. The reaction took place under metal-free conditions in most examples, although sometimes it was necessary to add very low catalyst loadings (0.01 mol %) of [RhCp*Cl_2_]_2_ to increase the chemical yields. A wide variety of Michael-acceptors, including enones, maleimides, unsaturated esters and sulfones, malonates, and acrolein, were successfully incorporated at the C3- and C4-positions of quinolines and isoquinolines, respectively, affording numerous substituted tetrahydro-(iso)quinolines **43** and **44** in acceptable to excellent yields. In addition, different substitution patterns with diverse substituents on the (iso)quinolinium substrates were well tolerated.

Kinetic studies and deuterium labeling experiments were carried out in order to investigate the reaction mechanism, which would involve an initial 1,4-dearomative hydride addition of formate ion (or Rh-H) to the quinolinium salt, followed by enamine addition to the electrophile and second hydride addition (Scheme 27).

**Scheme 28:**
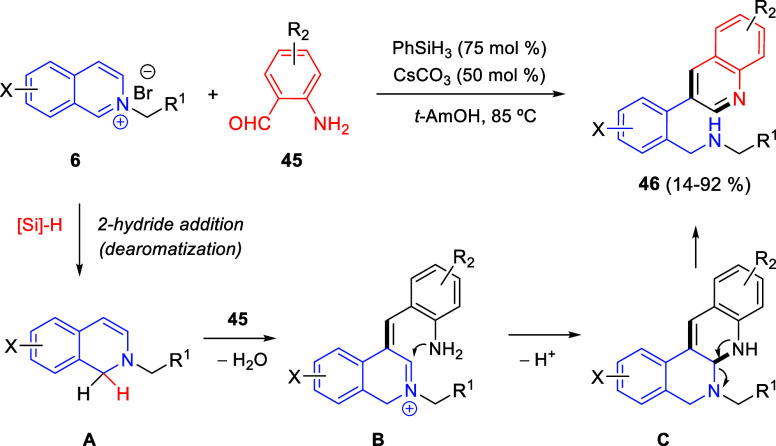
Hydride Transfer-Initiated Synthesis of 3-Functionalized Quinolines from *N*-Isoquinolinium Salts and 2-Aminobenzaldehydes

Zhang and co-workers reported in 2022 a cascade reaction starting with a 1,2-reductive dearomatization of isoquinolinium salts, employing hydrosilanes as hydride donors (Scheme 28).^59^ The reaction produced, under mild conditions, several 3-(2-aminomethyl)aryl quinolines **46** from *N*-isoquinolinium salts **6** and 2-aminobenzaldehydes **45** in generally good yields.

The mechanism of this transformation, which was supported by control experiments, would begin with the dearomative hydride transfer of PhSiH_3_ to the C1 of the isoquinolinium salt to form enamine intermediate **A** (Scheme 28). Intermolecular β-nucleophilic addition of this enamine to the aldehyde group of 2-aminobenzaldehyde **45**, followed by base-promoted dehydration would generate β-alkenyl iminium compounds **B**. Next, intramolecular cyclization by nucleophilic addition of the amine to the iminium carbon would produce aminals **C**. Finally, aromatization-induced cleavage of the C–N bond would provide the final products **46**. When the scope and limitations of the methodology were studied, very satisfactory functional group compatibility was found. However, strong electron-withdrawing groups in both the 2-aminobenzaldehyde **45** and the isoquinolinium salt **6** resulted in a significant decrease in yield, probably because they render difficult both the intramolecular nucleophilic addition in intermediate **B** and the β-nucleophilic addition of the enamine intermediate **A** to the aldehyde, respectively (Scheme 28).

#### 2.2.2 B(C_6_F_5_)_3_/Hydrogen Donor Combination

**Scheme 29:**
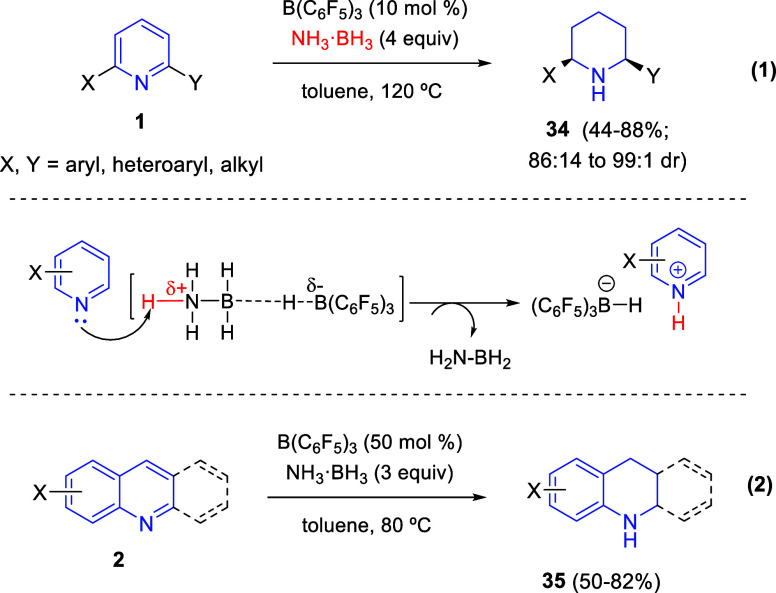
B(C_6_F_5_)_3_-Catalyzed Hydrogenation of *N*-Heterocycles with Ammonia Borane

A metal-free alternative method to the use of stoichiometric amounts of borohydrides for the hydrogenation of pyridines, quinolines, and isoquinolines is the *in situ* formation of borohydride by using catalytic amounts of an electron-deficient borane, such as B(C_6_F_5_)_3_, and stoichiometric quantities of hydrogen-donor species. In this context, Yang, Du, and co-workers developed in 2016 the B(C_6_F_5_)_3_-catalyzed transfer hydrogenation of pyridines employing ammonia borane as the hydrogen source (Scheme 29).^60^ In this manner, 2,6-disubstituted pyridines **1** were converted into the corresponding piperidines **34** in good yields with moderate to excellent *cis*-selectivities (Scheme 29, eq 1). 2,3-Disubstituted pyridines were also subjected to the B(C_6_F_5_)_3_-catalyzed transfer hydrogenation, albeit a drop of yield and *cis*-selectivity was observed. In this reaction, the lone pair of the pyridine nitrogen together with the B(C_6_F_5_)_3_ can split the N–H and B–H bonds of ammonia borane to form the borohydride salt of the activated pyridinium ring, which results in the subsequent reduction to finally produce piperidines (Scheme 29).

This B(C_6_F_5_)_3_-catalyzed transfer hydrogenation with ammonia borane was extended by Shi and co-workers to a variety of *N*-heterocycles with a six-membered ring, such as quinoline derivatives **2**, obtaining a broad range of hydrogenated *N*-heterocycles **35** in moderate to good yields (Scheme 29, eq 2).^61^ The enantioselective version of this methodology was also examined, although just 29% ee was achieved in the presence of a chiral phosphoric acid.

**Scheme 30:**
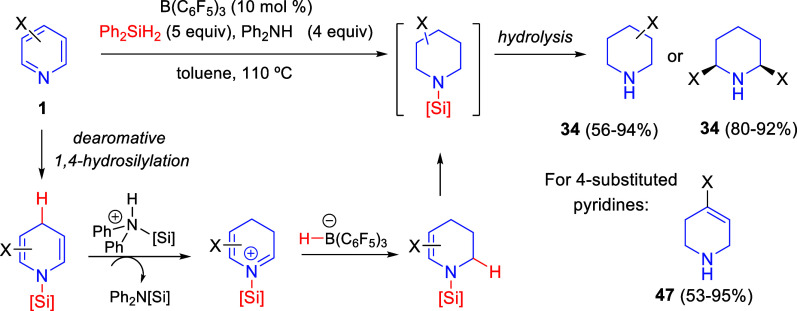
B(C_6_F_5_)_3_-Catalyzed Cascade Reduction of Pyridines

Despite the advances of the borane-catalyzed transfer hydrogenation methodology, it had some limitations (bulky *ortho* substituents were generally needed and unsaturated easily reducible functional groups were rarely compatible). In order to overcome them, the group of Wang designed an alternative methodology consisting of a B(C_6_F_5_)_3_-catalyzed cascade process involving a dearomative 1,4-hydrosilylation (or hydroboration), followed by transfer hydrogenation of the enamine double bonds with hydrosilanes (or hydroboranes) as hydride donors and primary or secondary amines as proton donors (Scheme 30).^62^ Under the optimized conditions, employing Ph_2_SiH_2_ and Ph_2_NH as the reducing system, a wide variety of *ortho*- and *meta*-substituted pyridines **1** were reduced to the corresponding piperidines **34** in moderate to excellent yields. Starting from 2,6-disubstituted pyridines, *cis*-piperidines **34** were selectively obtained in very good yields. However, when differently *para*-substituted pyridines **1** were subjected to the optimal cascade reaction conditions, 1,2,3,6-tetrahydropyridine derivatives **47** were obtained instead, achieving higher yields with pyridines bearing electron-withdrawing groups. The reaction was further extended to quinolines and isoquinolines with similar efficiency although, in the case of quinoline derivatives, a less hindered PhNH_2_ had to be used as the proton donor.

Mechanistic studies revealed that the cascade reduction would start with a dearomative hydrosilylation and the *N*-silyl-1,4-dihydropyridine intermediate would undergo rapid transfer hydrogenation of the remaining two double bonds to give the final product (Scheme 30). However, for *para*-substituted pyridines, the first step of the cascade reaction would be a 1,2-hydrosilylation and the subsequent transfer hydrogenation of the enamine double bond would yield the tetrahydropyridine product **47**.

**Scheme 31:**
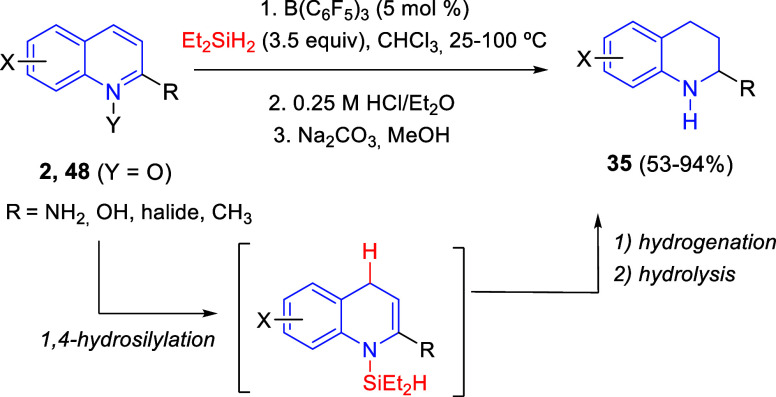
B(C_6_F_5_)_3_-Catalyzed Hydrogenative Reduction of *N*-Aromatic Rings

In 2017, Chang, Park, and co-workers extended the B(C_6_F_5_)_3_-catalyzed hydrogenative reduction methodology to other substituted *N*-aromatic rings by employing hydrosilanes as the reducing agent without a proton-donating additive (Scheme 31).^63^ The catalyst system was shown to work satisfactorily with substituted quinolines **2** and quinoline *N*-oxides **48**, including amino- and hydroxyquinolines, and afforded, after hydrolysis by treatment with an ethereal solution of HCl followed by neutralization with Na_2_CO_3_, the corresponding NH tetrahydroquinolines **34** in good to excellent yields. Mechanistic studies revealed that the cascade hydrogenation would involve a 1,4-hydrosilylation followed by reduction of the enamine intermediate (Scheme 31). The second reduction would probably involve the H_2_ generated *in situ* upon competitive dehydrogenative silylation of the azacyclic substrates, intermediates, and products.^64^

**Scheme 32:**
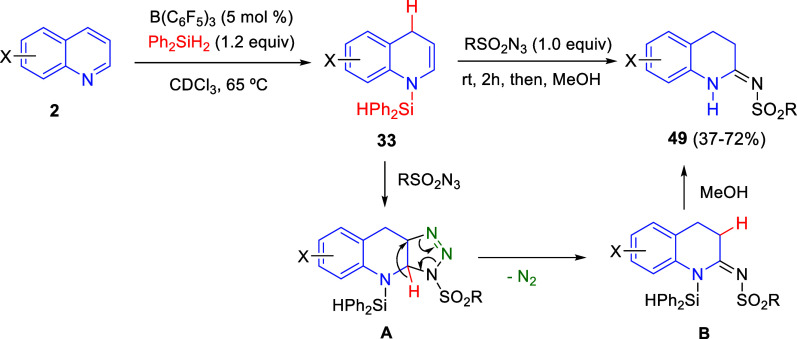
One-Pot Dearomative Hydrosilylation of Quinolines and Enamine-Azide (3 + 2) Cycloaddition

Later on, Joung and co-workers coupled the borane-catalyzed 1,4-hydrosilylation of quinolines with a regioselective (3 + 2) cycloaddition of the dearomatized enamine intermediates with sulfonyl azides (Scheme 32).^65^ The resulting triazoline intermediates **A** were immediately rearranged, via hydride shift and release of nitrogen gas to finally produce cyclic amidines (3,4-dihydroquinolinimines) **49** after the addition of methanol. A variety of sulfonyl azides, including sterically bulky ones and quinolines with substituents at positions 5–7, reacted in moderate to good yields, although electron-donating substituents seem to hinder the conversion of the first dearomatization step. Isoquinoline and 3-chloropyridine were also suitable substrates for this reaction.

**Scheme 33:**
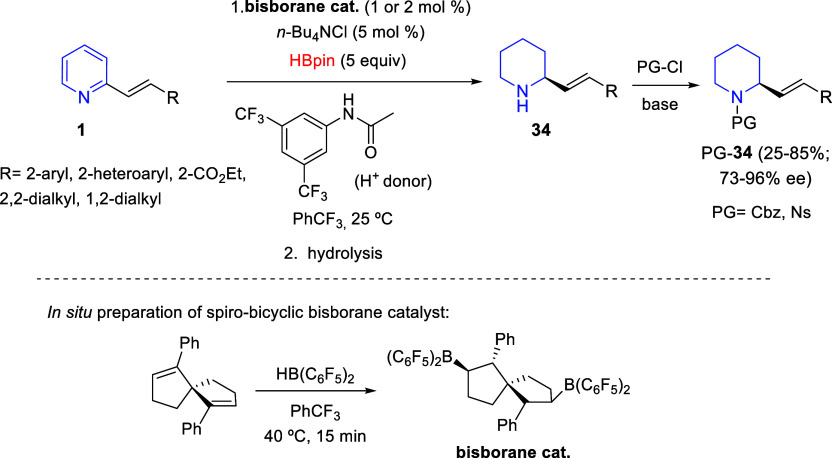
Borane-Catalyzed Chemoselective and Enantioselective Reduction of 2-Vinyl-Substituted Pyridines

Continuing their work on the B(C_6_F_5_)_3_-catalyzed hydrosilylation or hydroboration/transfer hydrogenation cascade process for the reduction of pyridines, Wang and co-workers reported in 2020 an enantioselective reduction of 2-vinyl-substituted pyridines **1** catalyzed by a chiral spiro-bicyclic bisborane, with HBpin and *N*-[3,5-bis(trifluoromethyl)phenyl]acetamide as reducing reagents (Scheme 33).^66^ The cascade sequence involved 1,4-hydroboration followed by transfer hydrogenation of a dihydropyridine intermediate. It proved to be highly chemoselective and exhibited excellent functional group tolerance so a wide variety of 2-substituted piperidines **34** were obtained in acceptable to good yields and with generally high enantioselectivities. Those piperidines were subjected to *N*-protection with a carboxybenzyl (Cbz) group or a *p*-nitrobenzenesulfonyl (Ns) group for their optical purity determination by HPLC (Scheme 33). To further demonstrate the synthetic utility of the methodology, the authors carried out two reactions on a gram scale, being able to lower the catalytic charge to 0.5 mol % with no loss in yield and enantioselectivity. They also synthesized an intermediate in a reported synthesis of caulophyllumine B.

**Scheme 34:**
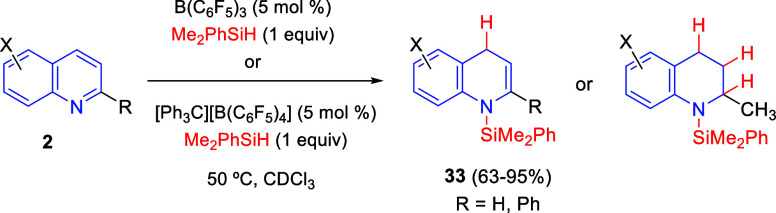
Hydrosilylation of Quinolines Catalyzed by B(C_6_F_5_)_3_ or by a Silylinium Ion

Nikonov and co-workers compared the hydrosilylation of quinolines catalyzed by B(C_6_F_5_)_3_ to the same reaction catalyzed directly by the silylinium ion R_3_Si^+^ (Scheme 34).^67^ This silylinium ion was generated by hydride abstraction with catalytic amounts of Ph_3_C^+^B(C_6_F_5_)_4_^–^. In most cases, 1,4-regioselective hydrosilylation of quinolines **2** was observed with both catalysts, rendering silylated dihydroquinolines **33** in higher yields with B(C_6_F_5_)_3_. When quinaldine was employed (R = Me), complete reduction to the corresponding tetrahydroquinaldine occurred (Scheme 34).

**Scheme 35:**
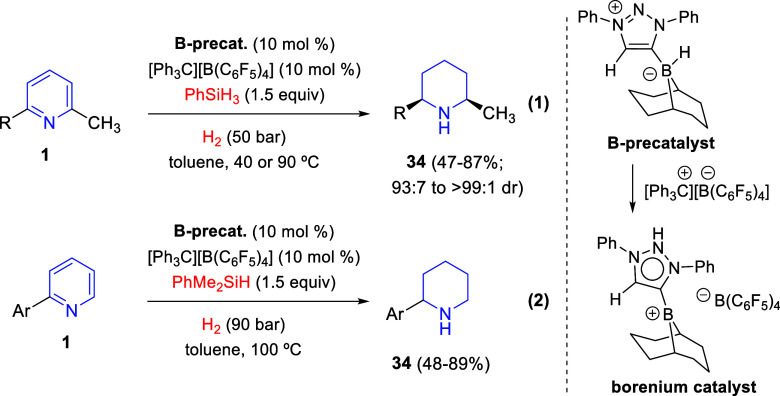
Borenium-Catalyzed Reduction of Pyridines with Hydrogen and Hydrosilane

An alternative strategy for the hydrogenation of pyridines, which also makes use of an electron-deficient boron catalyst, was developed in 2021 by Crudden and co-workers (Scheme 35). The authors demonstrated that mesoionic carbene-stabilized borenium ions, in the presence of hydrogen atmosphere and hydrosilanes, promoted a tandem hydrogenation reaction of di- and monosubstituted pyridines under mild conditions.^68^ The carbene-stabilized borenium catalyst was generated *in situ* by hydride abstraction with Ph_3_C^+^B(C_6_F_5_)_4_^–^ from the corresponding precatalyst. Therefore, the reductive combination of H_2_ (50 bar) and PhSiH_3_, under the catalysis of the borenium ion, allowed the authors to obtain 2,6-disubstituted pyridines **34** in moderate to good yields with high diastereoselectivities (Scheme 35, eq 1). The methodology was also valid for monosubstituted 2-arylpyridines **1**, although their lower steric demand meant that a higher temperature and a higher hydrogen pressure (90 bar) were required, as well as a bulkier hydrosilane, PhMe_2_SiH (Scheme 35, eq 2).

**Scheme 36:**
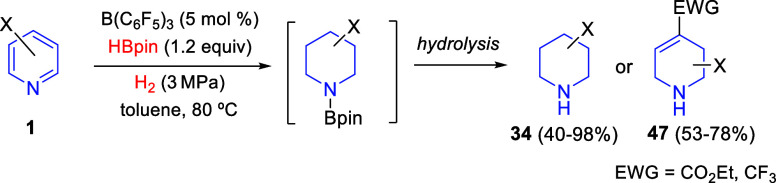
Borane-Catalyzed Reduction of Pyridines via Hydroboration/Hydrogenation Cascade

A similar strategy involving the combination of hydrogen atmosphere with HBpin as the hydride donor was reported by Wang and co-workers for the B(C_6_F_5_)_3_-catalyzed hydroboration/hydrogenation cascade reduction of pyridines (Scheme 36).^69^ This method was particularly effective for 2,3-disubstituted pyridines and demonstrated broad functional group tolerance, leading to the piperidines **34** in high yields and generally complete *cis* selectivity. It also proved to be suitable for obtaining 2,6-di- and 2-monosubstituted piperidines. However, disubstituted pyridines with an electron-withdrawing group at C4 provided tetrahydropyridines **47** instead. Mechanistic studies, including DFT calculations, indicated that the pyridine substrates and the piperidine products sequentially acted as bases in cooperation with B(C_6_F_5_)_3_ to split H_2_.

#### 2.2.3 Hantzsch Esters (HEH) Reductions

**Scheme 37:**
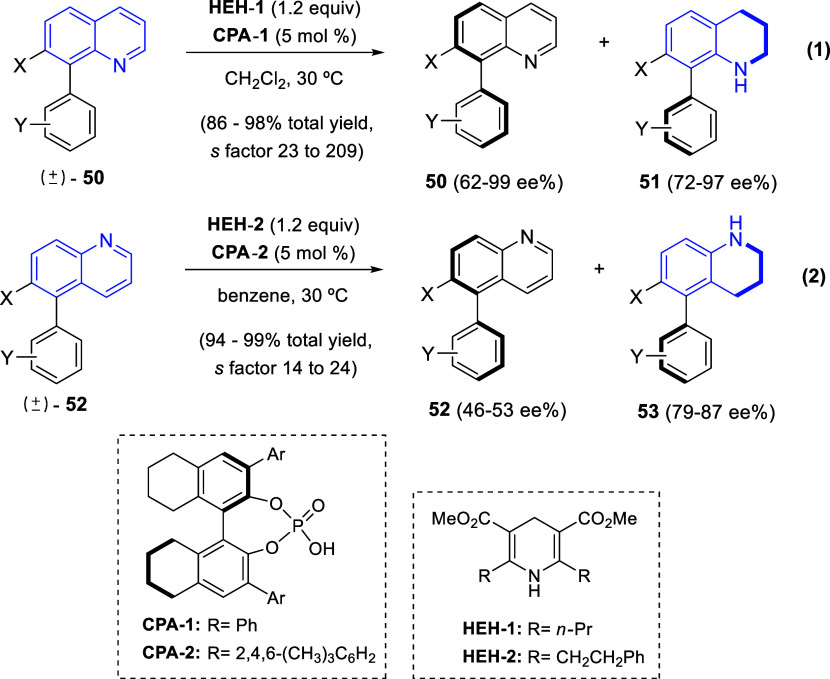
Kinetic Resolution of Axially Chiral 5- or 8-Substituted Quinolines via Asymmetric Transfer Hydrogenation

In 2016, Zhou and co-workers applied their previously developed methodology for the chiral phosphoric acid-catalyzed asymmetric transfer hydrogenation of 2-aryl-substituted quinolin-3-amines^70^ to the to the kinetic resolution of axially chiral biaryls (Scheme 37).^71^ It involved the use of chiral phosphoric acids (CPAs) as catalysts and Hantzsch esters (HEHs) as the hydrogen source. In this manner, the kinetic resolution of a variety of (±)-8-substituted quinoline-derived biaryls **50** was successfully accomplished with an excellent selectivity factor (up to 209) with **HEH-1** and **CPA-1**, rendering enantiomerically enriched compounds **50** and tetrahydroquinolines **51** (Scheme 37, eq 1). This strategy also worked satisfactorily with 5-substituted quinoline derived biaryls (±)-**52**, bearing the nitrogen atom further away from the non-C2 symmetry axis. In this case, the **HEH-2** and **CPA-2** system was necessary to achive enantiomerically enriched biaryls **52** and tetrahydroquinolines **53** (Scheme 37, eq 2). To increase the value of this methodology, it was demonstrated that the two different kinds of axially chiral skeletons were easily interconverted by hydrogenation of the recovered substrates **50** and **52** with Pd/C or reoxidation of the corresponding hydrogenation products **51** and **53** by using DDQ.

**Scheme 38:**
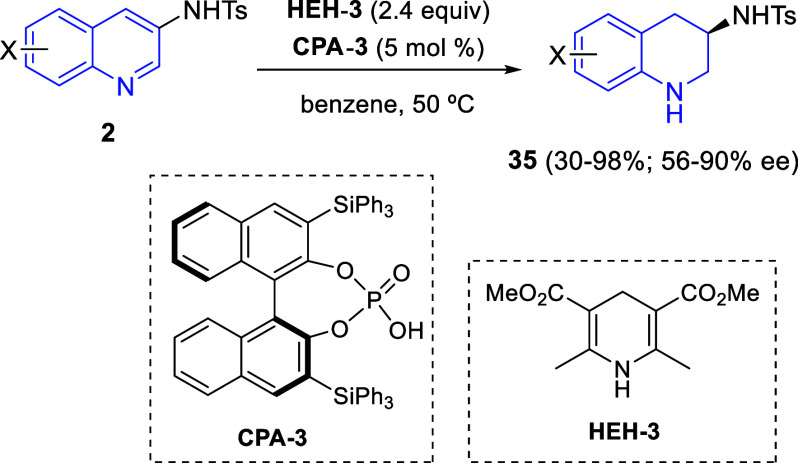
Enantioselective Transfer Hydrogenation of Quinolin-3-tosylamines

Bousquet and Pélinski applied Zhou’s asymmetric transfer hydrogenation methodology^70^ to quinolin-3-tosylamines **2** without substitution at the C2-position (Scheme 38).^72^ This reaction was accomplished with Hantzsch dihydropyridine **HEH-3** and the organocatalyst **CPA-3** and furnished 3-aminotetrahydroquinolines **35** with moderate to good yields and enantioselectivities (Scheme 38).

**Scheme 39:**
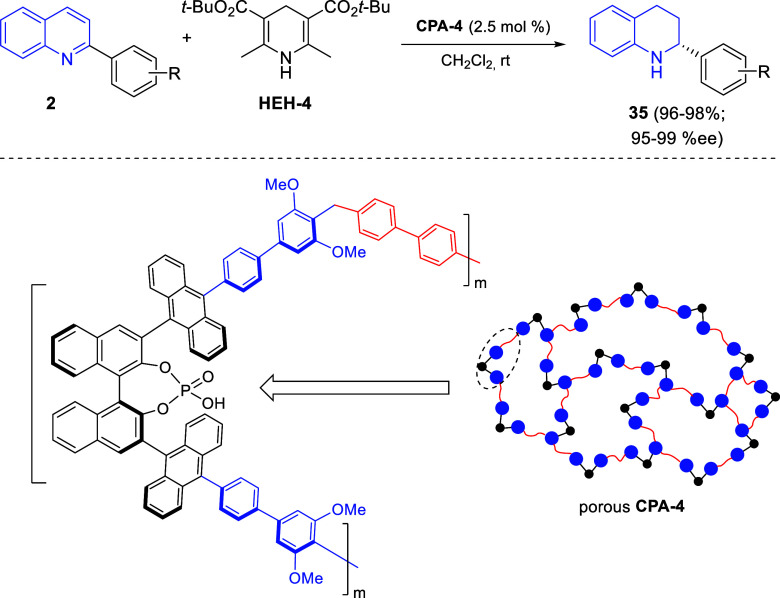
Enantioselective Dearomatization of 2-Substituted Quinolines Catalyzed by a Porous Chiral Phosphoric Acid

Very recently, Gu, Tu, You, and co-workers reported the synthesis and application of porous chiral phosphoric acids, such as the BINOL-derived compound **CPA-4**, for the enantioselective transfer hydrogenation of a variety of 2-phenylquinolines **2** in the presence of Hantzsch ester **HEH-4** (Scheme 39).^73^ The porous heterogeneous catalyst **CPA-4** could be recovered via centrifugation and reused up to 10 times without significant loss in terms of yield and enantioselectivity, which greatly enhances the synthetic utility of this methodology.

#### 2.2.4 Metal-Free Hydroborations

**Scheme 40:**
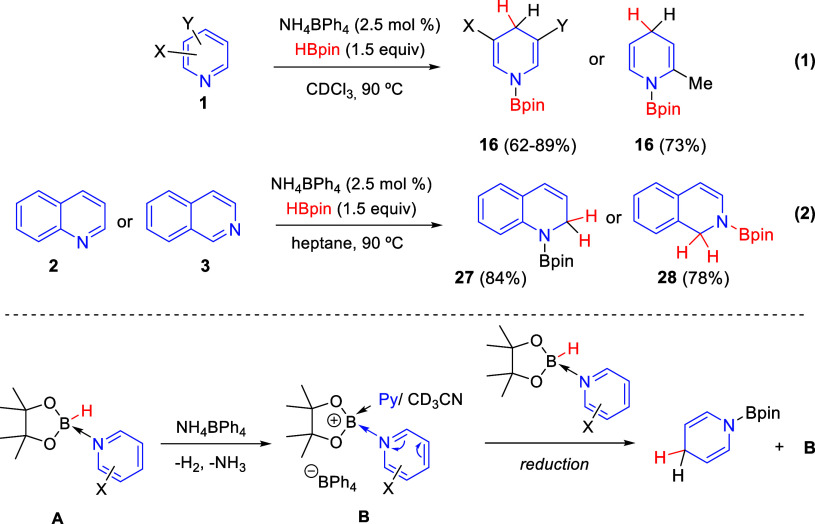
Regioselective 1,4-Hydroboration of Pyridines Catalyzed by an Acid-Initiated Boronium Cation

One of the most widely employed strategies for the synthesis of dihydropyridines from pyridines, avoiding the overreduction to piperidines, has been the catalyzed 1,4- and 1,2-hydroboration reactions. Besides the metal-catalyzed processes previously commented (see Section 2.1), Wright and co-workers developed in 2017 the boronium cation-catalyzed 1,4-hydroboration of 3-substituted pyridines **1** employing only pinacolborane, as the hydride source, and catalytic amounts of an ammonium salt initiator (NH_4_BPh_4_, which is inexpensive and commercially available) (Scheme 40).^74^ Different substituents at the C3-position were allowed, obtaining the 1,4-hydroboration products **16** in good yields and high 1,4-regioselectivity, including the sterically demanding 3,5-lutidine. However, the hydroboration of 2-substituted pyridines was found to be less successful and only 2-methylpyridine was successfully converted into the corresponding borylated dihydropyridine (Scheme 40, eq 1). Remarkably, the solvent played a significant role in the regioselectivity of the reaction, as polar solvents favored the selective 1,4-hydroboration while nonpolar solvents resulted in greater ratios of the 1,2-regioisomer. On the other hand, quinoline and isoquinoline were not affected by solvent polarity and afforded, both in heptane and acetonitrile, the 1,2-hydroboration products **27** and **28** in good yields and regioselectivity (Scheme 40, eq 2).

The proposed mechanism for this boronium-catalyzed hydroboration of pyridine would start with the reaction of the pyridine-coordinated HBpin complex (**A**) with NH_4_BPh_4_, resulting in the pyridine-stabilized boronium-BPh_4_ salt **B**, which would act as the catalyst during the reaction, and releasing H_2_ and NH_3_. Both H_2_ and the boronium cation were observed by ^1^H and ^11^B NMR, respectively. Furthermore, the structure of the proposed boronium species **B** (pinBPy_2_BPh_4_) was confirmed by single crystal X-ray diffraction. This boronium cation **B** would activate the coordinated pyridine toward reduction by a second pyridine-HBpin adduct (**A**), thus regenerating the catalytic boronium species **B** (Scheme 40).

**Scheme 41:**
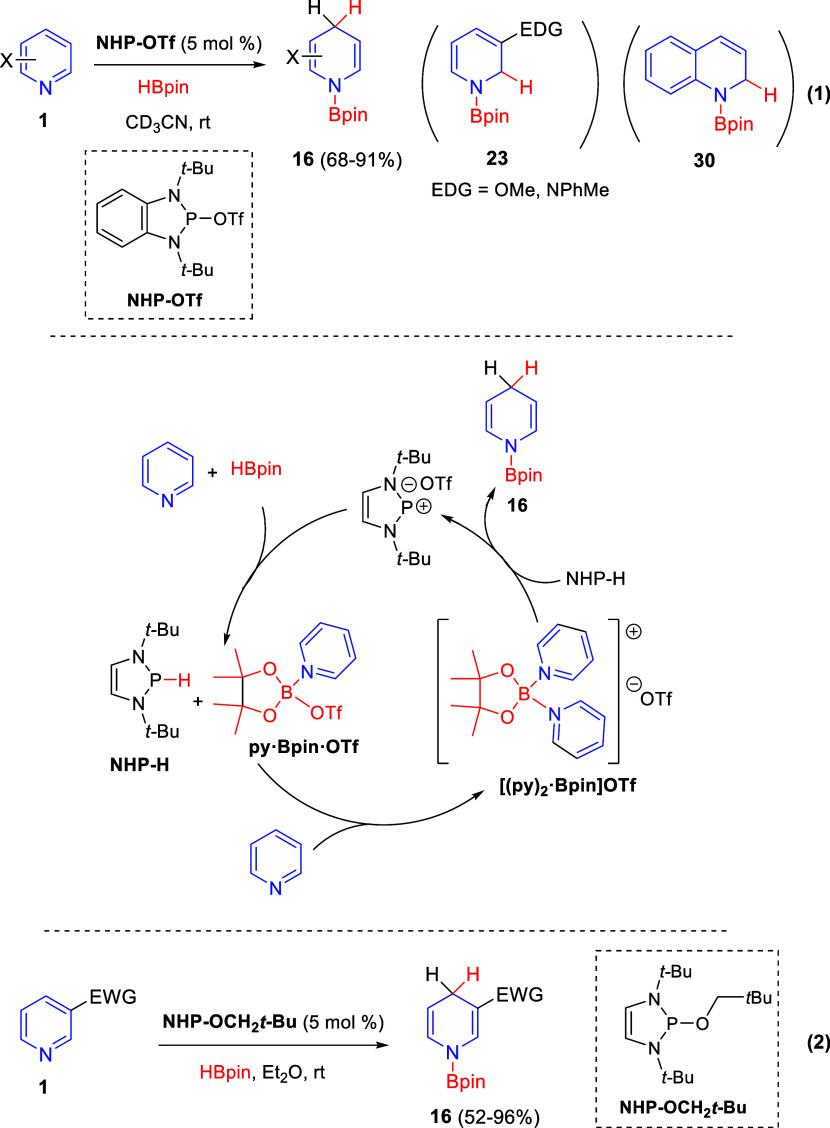
Regioselective Hydroboration of Pyridines Mediated by Phosphorus-Based Catalysts

In 2018, Kinjo and co-workers demonstrated that *N*-heterocyclic phosphenium triflates (NHP-OTf) efficiently catalyze the regio- and chemoselective hydroboration of pyridines **1** with good functional group tolerance (Scheme 41).^75^ In addition, different substituents at the C2-, C3-, and C5-positions were tolerated, giving rise to the corresponding hydroboration products **16** in good to excellent yields and with complete or very good 1,4-regioselectivity. However, 3-substituted pyridines with strong electron-donating groups and quinoline gave mostly the 1,2-hydroboration products (Scheme 41, eq 1).

Control reactions experiments and DFT calculations supported the proposed reaction mechanism, in which the phosphenium species would have a crucial role during the catalytic cycle by acting as hydrogen transfer reagent. First, both NHP-H and py·Bpin·OTf would be generated via hydride abstraction from HBpin by the phosphenium catalyst. Then, complexation of py·Bpin·OTf with a second pyridine molecule would afford the boronium [(py)_2_·Bpin]·OTf. One of the two activated pyridines of this boronium salt would be reduced by NHP-H, affording the 1,4-hydroboration product selectively and releasing the phosphenium catalyst and the dearomatized pyridine **16** (Scheme 41).

Very shortly after Kinjo’s publication, Speed and co-workers also reported the regioselective 1,4-hydroboration of pyridines **1** mediated by a *N*-heterocyclic phosphorene catalyst (Scheme 41, eq 2).^76^ The neutral catalytic system, which is believed to function mechanistically similar to that proposed by Kinjo, was limited to 3-substituted pyridines with strong electron-withdrawing groups.

**Scheme 42:**
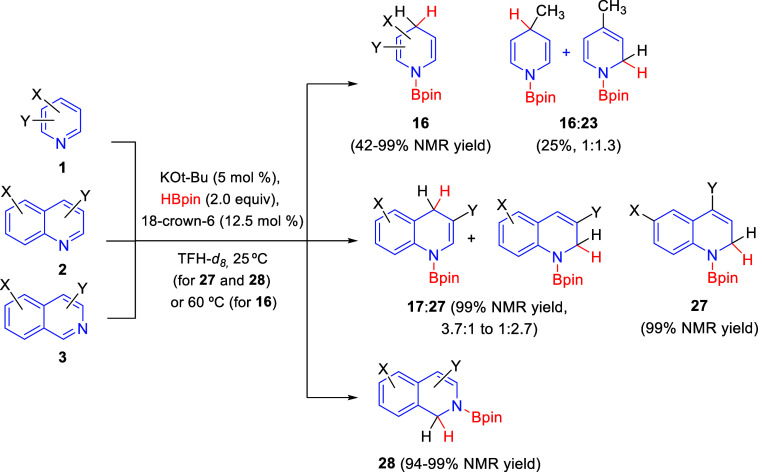
Potassium *tert*-Butoxide-Promoted Regioselective Hydroboration of *N*-Heteroarenes

Potassium *tert*-butoxide and hydride bases have also been shown to successfully catalyze the regioselective hydroboration of pyridine, quinoline, and isoquinoline rings. The first to develop this mode of catalysis were Park, Chang, and co-workers in 2019, who employed catalytic amounts of *t*-BuOK in the presence of substoichiometric quantities of 18-crown-6 for the hydroboration of pyridines **1**, quinolines **2**, and isoquinolines **3** with HBpin (Scheme 42).^77^ In this manner, 3,5-disubstituted and 3-substituted pyridines, as well as, 2-methylpyridine, were regioselectively hydroborated with generally good 1,4-selectivity, giving 1,4-dihydropyridines **16**. However, mixtures of 1,2- and 1,4-regioisomers **16** and **23** were obtained with 4-methylpyridine. Quinolines **2** reacted with inferior regioselectivity, affording mixtures of the 1,4- and 1,2-hydroboration products **17** and **27**, respectively. Only C4-substituted quinolines reacted with high or complete 1,2-selectivity, probably due to steric factors. In addition, 1,2-hydroborated isoquinolines **28** were obtained in high yields and complete regioselectivity.

**Scheme 43:**
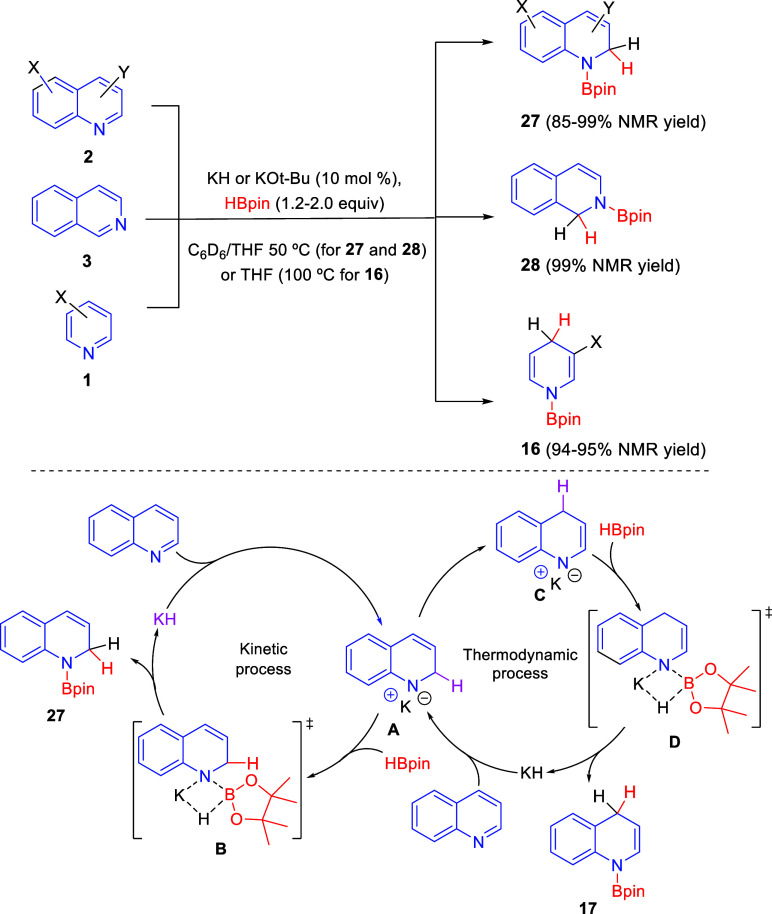
Regioselective Hydroboration of *N*-Heteroarenes with a Potassium-Based Catalyst

Very shortly thereafter, Zhang, He, and co-workers also published the regioselective 1,2-hydroboration of *N*-heteroarenes with pinacolborane using a potassium-based catalyst (Scheme 43).^78^ In the presence of 10 mol % of *t*-BuOK or KH, a variety of quinolines **2** and isoquinoline underwent 1,2-hydroboration to give products **27** and **28** in excellent yields and very high regioselectivity. On the other hand, the hydroboration of pyridine and several 3-alkylpyridines **1** was also carried out with very good yields and excellent 1,4-regioselectivity.

Mechanistic and kinetic studies allowed the authors to propose a reaction mechanism for this potassium-catalyzed hydroboration of quinolines in which KH would be the actual active catalyst, which would be *in situ* generated from the reaction of *t*-BuOK and HBpin. Initially, quinoline would undergo 1,2-addition of KH to generate the kinetic intermediate **A**. This would react with HBpin through transition state **B** to afford the 1,2-hydroboration product **27** and release KH. However, intermediate **A** can also isomerize in a thermodynamic process to intermediate **C**, which would react in the same way with HBpin, releasing KH to produce the 1,4-hydroboration product **17**. The authors disclosed that the reaction of kinetic intermediate **A** with HBpin was faster than the isomerization to intermediate **C**, thus explaining the 1,2-regioselective hydroboration observed. In the case of the pyridine derivatives, the thermodynamic process would be preferable upon heating at 100 °C, thus achieving the 1,4-hydroboration products **16** (Scheme 43).

**Scheme 44:**
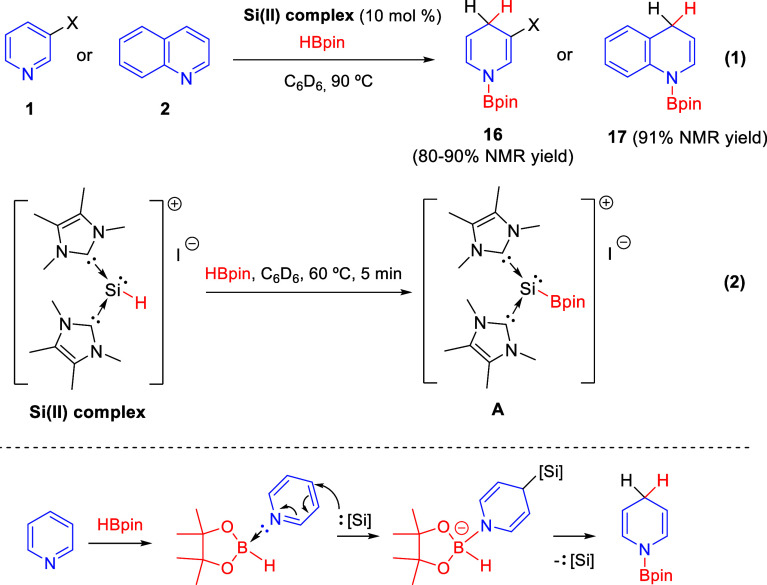
NHC-Parent Silyliumylidene Cation for the 1,4-Regioselective Hydroboration of *N*-Heteroarenes

The groups of Siu, Su, and So showed that a NHC-based silyliumylidene cation complex efficiently catalyzes the regioselective 1,4-hydroboration of unsubstituted and 3-substituted pyridines **1** and quinoline **2** with HBpin in excellent yield and complete regioselectivity (Scheme 44, eq 1).^79^ The described silicon(II) complex was transformed, under the reaction conditions, into the NHC-borylsilyliumylidene complex, which was also capable to act as a catalyst during the hydroboration reaction (Scheme 44, eq 2).

The mechanism proposed by the authors would start with the activation of the pyridine ring **1** through the coordination with HBpin, followed by the nucleophilic addition of the silicon complex at the *para*-position in order to minimize the steric congestion. The hydride substitution from the borane moiety would produce the *N*-boryl-1,4-dihydropyridine product together with the regeneration of the catalysts (Scheme 44).

**Scheme 45:**
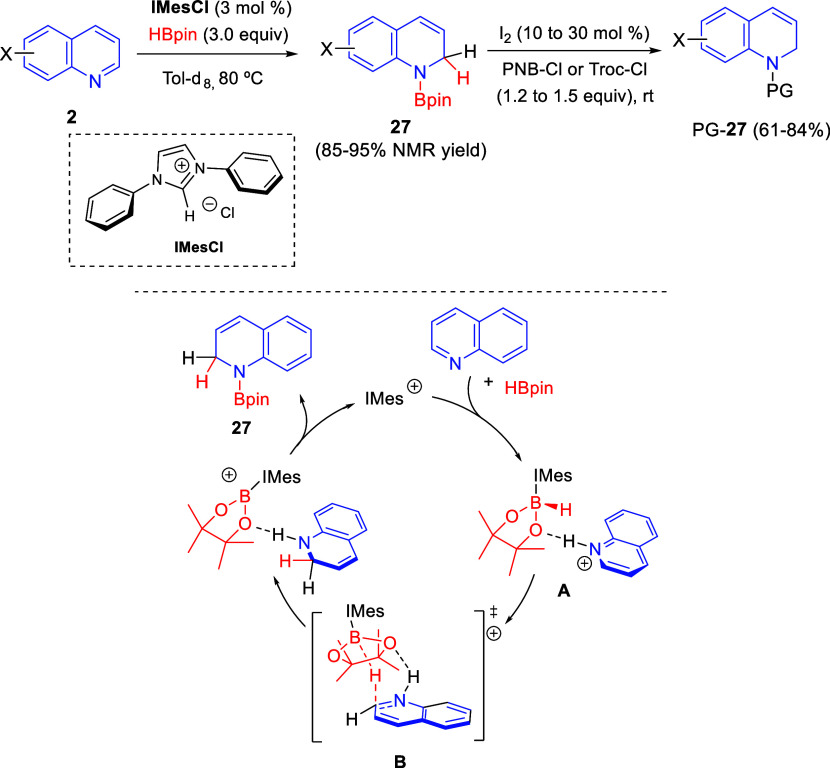
NHC-Catalyzed 1,2-Regioselective Hydroboration of Quinolines

Chang and co-workers, after their *t*-BuOK-promoted hydroboration of *N*-heteroarenes, described in 2020 the first example of NHC-catalyzed 1,2-hydroboration of quinolines (Scheme 45).^80^ By using the imidazolium salt IMesCl as the NHC precursor, in the absence of external bases, and HBpin as a reducing reagent in toluene, a variety of quinolines **2** were efficiently reduced to the corresponding *N*-boryl-1,2-dihydroquinolines **27** in very good yields (by ^1^H NMR analysis) and displaying high functional group tolerance. Labile dihydroquinoline products could be isolated in high yields through *in situ N*-protection with 4-nitrobenzoyl chloride (PNB-Cl) or 2,2,2-trichloroethoxycarbonyl chloride (Troc-Cl), employing molecular iodine as a catalyst.

Combined experimental and theoretical studies allowed the authors to propose a mechanism in which, first, a IMes-HBpin adduct **A** would be formed from the interaction of the IMesCl salt with HBpin by quinoline protonation. Protonated quinolinium would be presumably attached to the IMes-HBpin adduct via hydrogen bonding of the quinolinium N and the borate O. Then, the hydride from the activated borohydride would be added to the C2-position of the quinolinium ring via a key 6-membered transition state **B** to finally afford the *N*-boryl-1,2-dihydroquinoline product **27** and regenerate the IMesCl salt (Scheme 45).

**Scheme 46:**
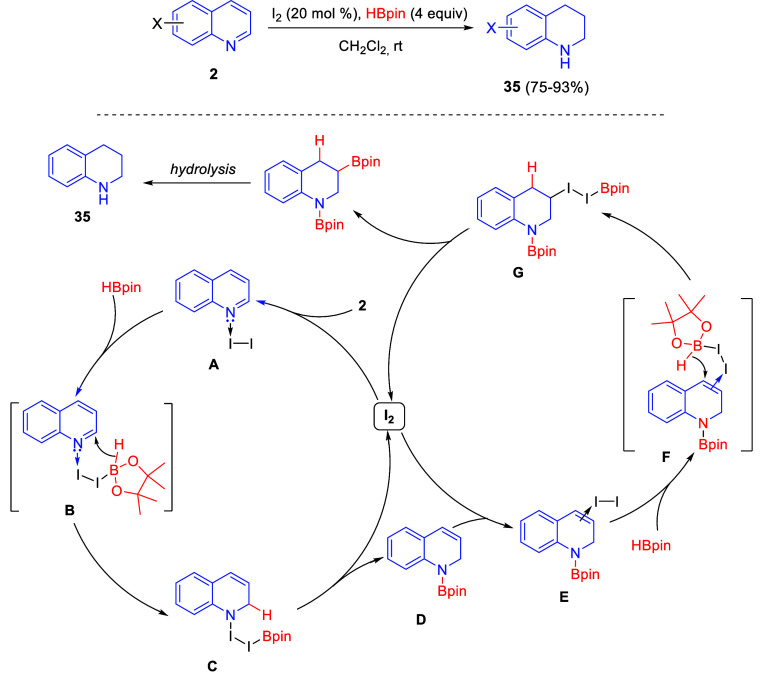
Iodine-Catalyzed Reduction of Quinolines with HBpin

In 2018, Chang, Wang, and co-workers described an alternative methodology to the borane-catalyzed hydrogenative reduction of quinolines to tetrahydroquinolines by replacing the catalyst B(C_6_F_5_)_3_ (see Scheme 31) with molecular iodine (20 mol %) and using HBpin as the hydrogen donor (Scheme 46).^81^ Under the optimized mild conditions, the reduction of a series of quinolines **2** was carried out, obtaining the corresponding 1,2,3,4-tetrahydroquinolines **35** with very good yields. No clear influence of the electronic properties or the position of the substituents at the starting quinoline ring was observed in terms of chemical yield. To demonstrate the practical utility of the developed reaction, a gram-scale reaction was successfully carried out and several products with biological activity were efficiently obtained.

Based on preliminary mechanistic NMR and deuterium labeling studies, together with previously reported results, the authors proposed a catalytic cycle for each bond reduction starting with the formation of a quinoline-I_2_ complex **A**. This complex would undergo hydride addition from HBpin, facilitated by I-B interaction (**B**). The new 1,2-dihydroquinoline **C** would capture the borenium ion to give intermediate **D** and regenerate the molecular iodine catalyst. The 1,2-dihydroquinoline intermediate **D** would undergo a cycle similar to that mentioned for quinoline giving rise to 1,3-diborylated tetrahydroquinoline which, after hydrolysis, would yield the final tetrahydroquinoline **35** (Scheme 46).

## 3 CYCLOADDITION REACTIONS AND ANNULATIONS

Cycloaddition reactions involving the use of heterocycles such as pyridines, quinolines, and isoquinolines are especially relevant, since they allow for creating complex heterocyclic scaffolds. Furthermore, if those reactions end up with the dearomatization of the heterocycle, multiple stereocenters can be created in a very simple manner.

The most common way in which nitrogen heterocycles participate in cycloaddition-type reactions is through quaternization of the nitrogen. The resulting salts are precursors of a wide variety of dipoles such as pyridinium- and (iso)quinolinium ylides and imides, *N*-heterocyclic zwitterions, or Huisgen 1,4-dipoles, which participate in various dipolar cycloadditions to generate new families of heterocyclic skeletons. The heterocyclic moiety, either in neutral form or as a salt, also can act as diene, dienophile or dipolarophile partner in several cycloaddition reactions.

The last advances in dearomatizations of heteroarenes by means of cycloaddition reactions are compiled herein, classified by the type of dipole.

### 3.1 Pyridinium Ylides

Pyridinium ylides, as a special type of azomethine ylides, are versatile synthetic intermediates in organic synthesis, which can act as 1,3-dipoles. Classified as nitrogen ylides with a pyridinium moiety, they exhibit high stability and constitute suitable building blocks for the synthesis of a wide variety of *N*-heterocycles.^82−85^ Among the many reactions that pyridinium ylides can undergo, we will focus on those involving the dearomatization of the pyridine ring.

**Scheme 47:**
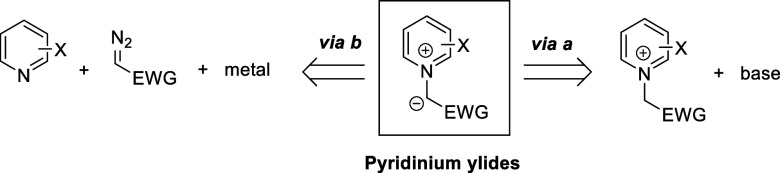
Generation of Pyridinium Ylides

There are two general ways in order to access pyridinium ylides. The most common approach involves deprotonation of pyridinium salts containing an electron withdrawing group (Scheme 47, ***via a***). The second one is the reaction of metal carbenes, in turn prepared from diazo compounds and metals, with pyridine as a Lewis base (Scheme 47, ***via b***). This last pathway tends to be more convenient as it takes place under aprotic conditions and offers the opportunity for further transformations in tandem or multicomponent protocols.

**Scheme 48:**
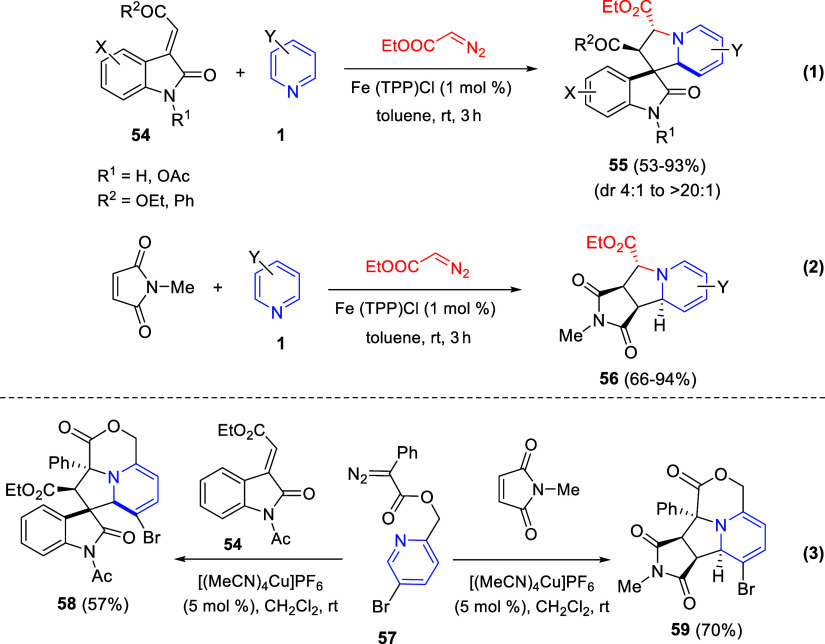
Synthesis of Tetrahydroindolizines through the Formation of Pyridinium Ylides from Diazo Compounds

In 2016, Dowden and co-workers reported a multicomponent synthesis of highly functionalized tetrahydroindolizidines by means of the 1,3-cycloaddition reaction of pyridinium ylides, *in situ* generated from metallocarbenes, with electrophilic alkenes; namely, 3-alkenyloxindoles **54** and *N*-methylmaleimide (Scheme 48).^86^ Reactions between these alkenes, substituted pyridines **1** and ethyl diazoacetate, in the presence of the Fe(TPP)Cl (TPP = tetraphenylporphyrin) complex as the catalyst, afforded tetrahydroindolizidines **55** and **56** (Scheme 48, eqs 1 and 2, respectively) in good yields and moderate to complete diastereoselectivity. Substitution on the pyridine ring with both electron-withdrawing and electron-donating groups was tolerated in the process.

On the other hand, when pyridine **57**, derived from diazophenylacetic acid was tested, the Fe(III) catalyst failed to provide the final products; however, the use of [(MeCN)_4_Cu]PF_6_ led to the desired cycloadducts **58** and **59** as single diastereoisomers in good yields, with 3-alkenyloxindole and *N*-methylmaleimide, respectively (Scheme 48, eq 3).

**Scheme 49:**
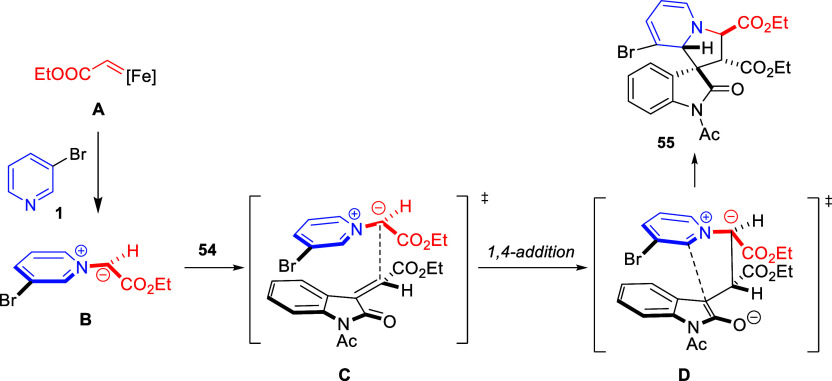
Proposed Mechanism to Rationalize the Synthesis of Spiroindolizidine Oxindoles through Cycloaddition of Pyridinium Ylides

The authors performed control experiments and concluded that nucleophilic pyridines **1** would add to the *in situ* generated metallocarbene **A** to catalytically form the key pyridinium ylide **B**. Then, the cycloaddition with the electrophilic alkene such as oxindole **54** would take place in a stepwise manner involving the 1,4-addition of the pyridinium ylide to the alkenyloxindole through transition state **C**, followed by a Mannich-type addition to the pyridine ring to afford the dearomatized spirocyclic product **55** (Scheme 49).

**Scheme 50:**
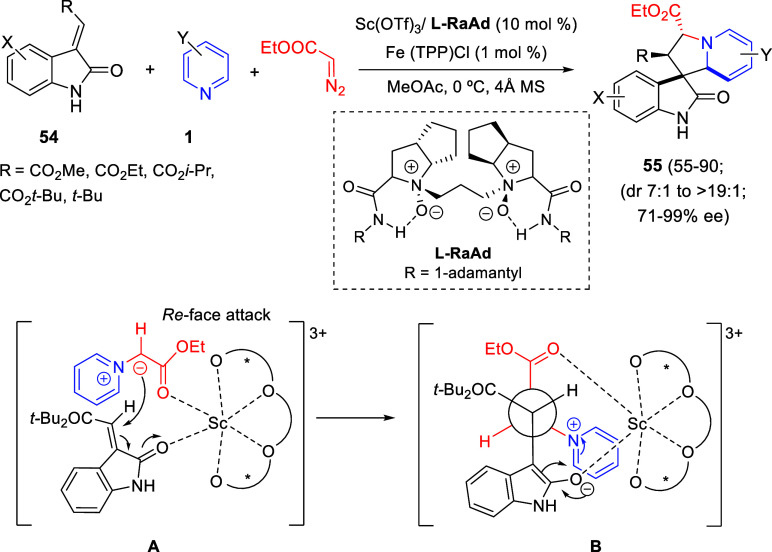
Asymmetric Synthesis of Tetrahydroindolizines by Bimetallic Relay Catalyzed Cycloaddition of Pyridinium Ylides with Oxindoles

The enantioselective version of this 1,3-dipolar cycloaddition reaction of pyridinium ylides with 3-alkenyloxindoles was developed by Feng and co-workers taking advantage of a bimetallic relay catalytic system involving an achiral iron(III) catalyst and a chiral *N*,*N*′-dioxide-scandium(III)-complex (Scheme 50).^87^ The authors envisioned that, once the pyridinium ylide was formed, the chiral Lewis acid complex could promote the subsequent cycloaddition reaction in an enantioselective manner. They found that the combination of the iron salt Fe(TPP)Cl with scandium triflate and the chiral *N*,*N*′-dioxide ligand **L-RaAd** constitutes an appropriate catalytic system for the synthesis of tetrahydroindolizidines **55**, in good yields and excellent diastereo- and enantioselectivities, by means of the cycloaddition reaction of diazoacetate, substituted pyridines **1** and alkenyloxindoles **54**. Electron-donating groups (X) on the phenyl ring of oxindoles **54** provided good results in terms of yield, diastereoselectivity, and ee values, while electron-withdrawing substituents yielded the final products with lower ee values, probably due to the increase in background reactivity. Moreover, the substituent (R) at alkene moiety played an important role in the enantiocontrol of the cycloaddition reaction, providing better results with bulky substituents. On the other hand, substituents (Y) on the pyridine ring did not affect the efficiency of the process.

Regarding a plausible explanation about the stereoselectivity, the authors obtained a crystal structure of the **L-RaAd**/Sc(OTf)_3_ complex, showing that the scandium cation formed a six-coordinated octahedral geometry, with two coordination sites occupied by the ester group of the pyridinium ylide and the amide group of the oxindole moiety (Scheme 50, intermediate **A**). At this point, the *Si* face of the oxindole would be shielded by the adamantyl group of the catalyst, delivering the nucleophilic conjugate addition to the *Re* face to render intermediate **B**. Subsequent Mannich-type addition, again controlled by the scandium octahedral complex, would form the spirocyclic adduct with excellent stereochemical control.

**Scheme 51:**
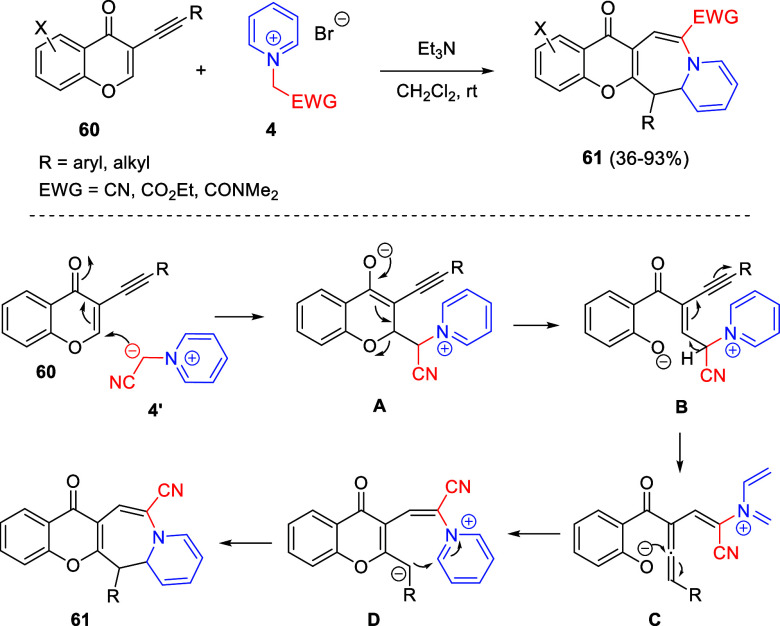
Base-Promoted Cascade Reaction of 3-(1-Alkynyl)chromones with Pyridinium Ylides

Although not a strictly dearomative cycloaddition process, Hu and co-workers described a base promoted tandem reaction of 3-(1-alkynyl)chromones **60** with pyridinium ylides (formed *in situ* from pyridinium salts **4**) leading to chromeno[2,3-*d*]azepine derivatives **61** in moderate to good yields (Scheme 51).^88^ The electronic nature of the substituents (X) on the phenyl ring of chromones **60** did not affect the efficiency of the process. However, substitution at the alkynyl group (R) had an important effect, since aliphatic groups slowed down the reaction, which turned into a clear decrease in yield. On the other hand, nitriles, amides or esters at the pyridinium salts **4** were compatible with the synthesis of the corresponding cycloadducts.

The overall transformation involves several reactions. Upon formation of the pyridinium ylide **4′** by deprotonation of the starting pyridinium salt, a Michael-type addition to chromone derivative **60** would take place to form intermediate **A**, which would evolve with ring opening of the chromone. Then, deprotonation followed by alkyne-allene isomerization and cyclization would regenerate the chromone ring. Final intramolecular 1,2-addition would occur at the C=N bond of the pyridinium intermediate **D** to yield the target products **61** (Scheme 51).

**Scheme 52:**
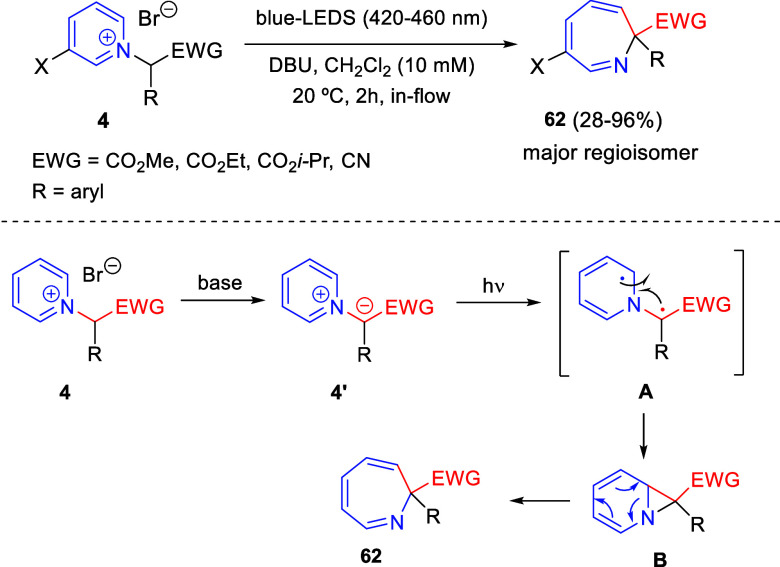
Synthesis of Azepines by Visible Light-Mediated Dearomative Ring Expansion of Pyridinium Ylides

Beeler and co-workers found that pyridinium ylides undergo dearomative ring expansion promoted by visible-light irradiation (Scheme 52).^89^ Thus, when pyridinium bromide salts **4** were irradiated with blue light in the presence of 1,8-diazabicyclo[5.4.0]undec-7-ene (DBU), an aza-Buchner ring expansion occurred, leading to azepine derivatives **62** in moderate to very good yields. The reaction was conducted in a flow photoreactor to minimize the formation of byproducts by aerobic oxidation. Ester or nitrile substituents were appropriate electron-withdrawing groups on the pyridinium salt, as well as aromatic substituents at the α-position (R). The regioselectivity of the ring expansion improved with sterically hindered substituents at the C3-position of the pyridinium salts.

Additionally, the authors performed experiments to propose a plausible mechanism for this transformation. After the base-promoted generation of the pyridinium ylides **4′**, these would be photochemically excited to singlet diradicals **A**. Then, radical recombination would give aza-norcaradiene intermediates **B**, which would undergo 6π electrocyclic ring opening to afford final azepine products **62** (Scheme 52).

**Scheme 53:**
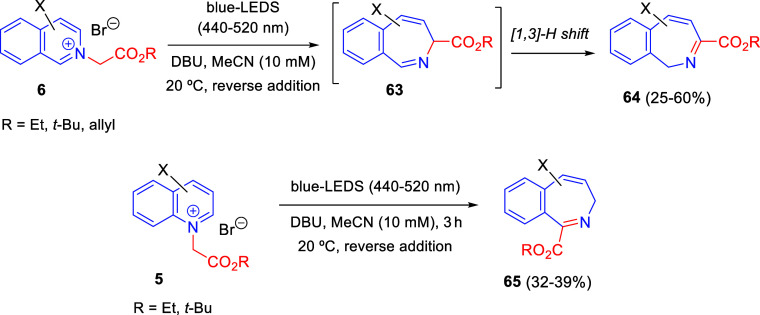
Synthesis of Policyclic Azepines by Visible-Light-Mediated Dearomative Ring Expansion of Isoquinolinium and Quinolinium Ylides

This reaction was further extended to quinoline and isoquinoline derivatives to provide polycyclic azepines (Scheme 53). Isoquinolinium salts **6** underwent the photochemical ring expansion in the presence of DBU to render α-imino esters **64**, after isomerization via 1,3-hydride shift on intermediates **63**). Likewise, quinolinium salts **5** gave 3*H*-benzo[*c*]azepines **65** in moderate yields.

**Scheme 54:**
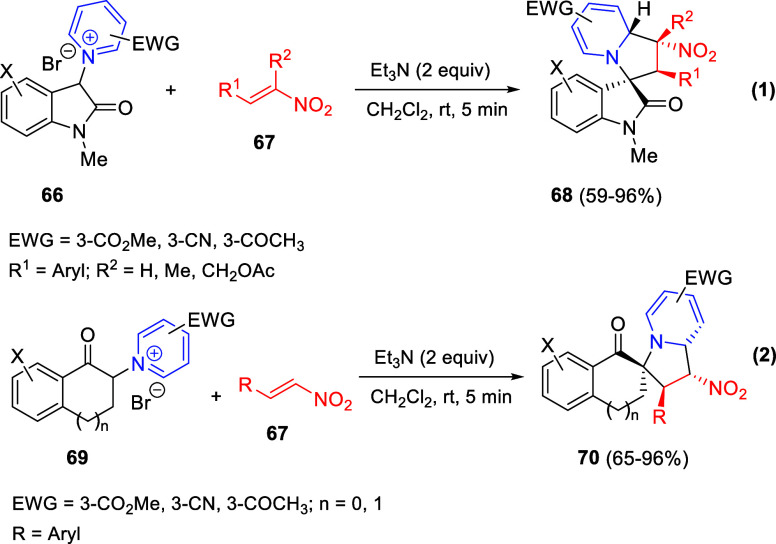
Diastereoselective Dearomative (3 + 2) Cycloaddition with *In Situ* Generated Pyridinium Ylides

In 2021, Yang and Wang developed a highly stereoselective dearomative (3 + 2) cycloaddition of cyclic pyridinium ylides with nitroolefins, leading to spiro-indolizidine scaffolds (Scheme 54).^90^ The authors designed disubstituted pyridinium salts bearing an electron-withdrawing group on the pyridine moiety in order to promote the cycloaddition reaction by increasing their electrophilicity and avoiding the elimination of the pyridine ring as a leaving group. Under the optimized conditions, oxindole pyridinium salts **66** reacted with nitroolefins **67**, providing, in 5 min, the target spiro-indolizidine derivatives **68** in good to excellent yields and outstanding diastereocontrol (>19:1 dr values) (Scheme 54, eq 1). It is worth mentioning that the reaction did not take place in the absence of the electron-withdrawing group on the pyridine ring, and this group should be at the 3-position in order to be effective.

Other electron-deficient pyridinium salts such as naphthanenone and indanone derivatives **69** were also good pyridinium ylide precursors for the (3 + 2) cycloaddition reaction with nitroolefins **67**, producing the corresponding spiro-indolizidine skeletons **70** in very good yields with excellent diastereoselectivity (Scheme 54, eq 2).

**Scheme 55:**
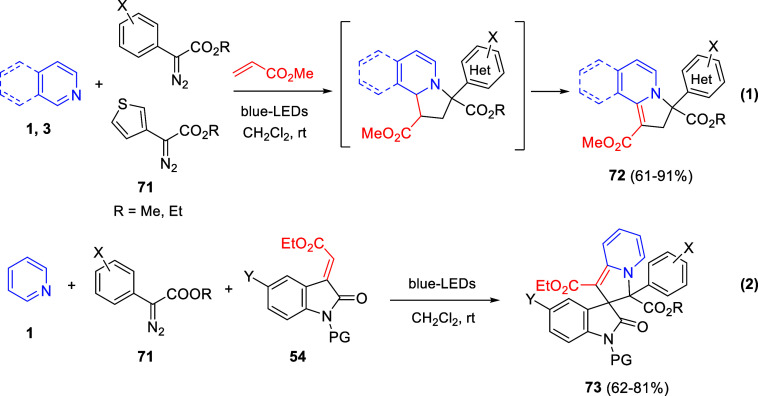
Blue LED-Mediated (3 + 2) Cycloaddition with Pyridinium and Isoquinolinium Ylides from Aryl Diazoesters

Sen and co-workers reported the reaction of pyridinium ylides with conjugated esters under visible-light irradiation (Scheme 55).^91^ Taking advantage of the photolytic generation of nitrogen ylides from *N*-heteroarenes and aryl diazoesters, the authors developed a three-component (3 + 2) cycloaddition reaction among pyridine or isoquinoline, aryl diazoesters **71**, and methyl acrylate, which rendered dihydroindolizine scaffolds **72** after oxidation of the corresponding tetrahydroindolizines (Scheme 55, eq 1). Both electron-donating and electron-withdrawing groups on the aryl diazoester counterpart were compatible with the process.

This cycloaddition reaction was further extended to 3-alkenyl oxindoles **54** as dipolarophiles, giving rise to spirocyclic dihydroindolizidines **73** in good yields (Scheme 55, eq 2). The use of other dipolarophiles such as phenyl acetylene of 2-butyne did not provide the desired adducts.

**Scheme 56:**
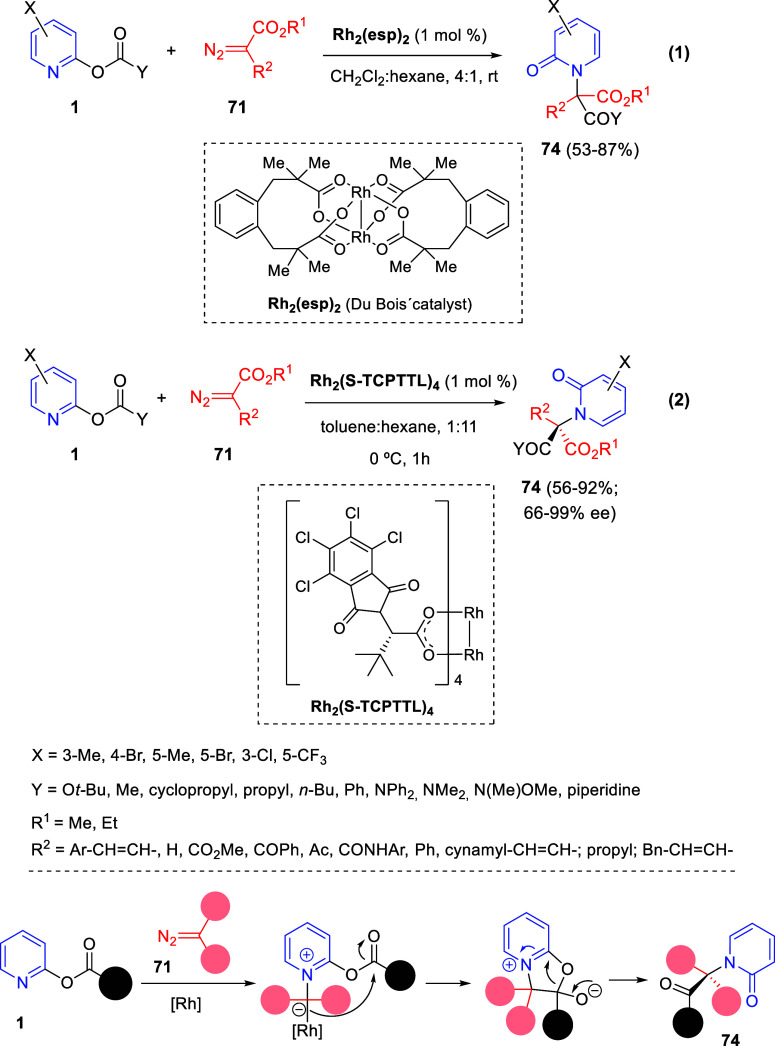
Rhodium-Catalyzed Dearomatization of *O*-Substituted Pyridines with Diazocompounds for the Synthesis of *N*-Substituted 2-Pyridones

In 2019, the groups of Sun and Zhang developed an efficient synthesis of *N*-substituted 2-pyridones **74** by means of a rhodium-catalyzed dearomatization of 2-*O*-substituted pyridines **1**, employing diazocompounds **71** as the alkylation reagents (Scheme 56, eq 1).^92^ The reaction took place in good yields with Rh_2_(esp)_2_ as the catalyst, and it was amenable to a great variety of carbene precursors such as vinyl diazoacetates, alkyl, aryl, and other types of diazocompounds. In addition, several *O*-carboxyl and *O*-carbonyl groups on the pyridine counterpart, as well as *O*-amide pyridines were tolerated. The enantioselective version of this catalytic asymmetric dearomatization was achieved with a chiral rhodium catalyst, Rh_2_(S-TCPTTL)_4_, rendering enantiomerically enriched 2-pyridones **74** in good yields and excellent ee values, in general (Scheme 56, eq 2).

DFT calculations suggested that the rhodium-catalyzed reaction of the starting pyridine with the diazocompound would allow the formation of a pyridinium ylide, which would undergo 1,4-acyl migration to render final *N*-substituted 2-pyridones (Scheme 56).

### 3.2 Quinolinium Ylides

Quinolinium ylides are another type of heteroaromatic *N*-ylides, which, like pyridinium ylides, can be engaged in 1,3-dipolar cycloadditions with various dipolarophiles in order to access new polyheterocyclic frameworks.

**Scheme 57:**
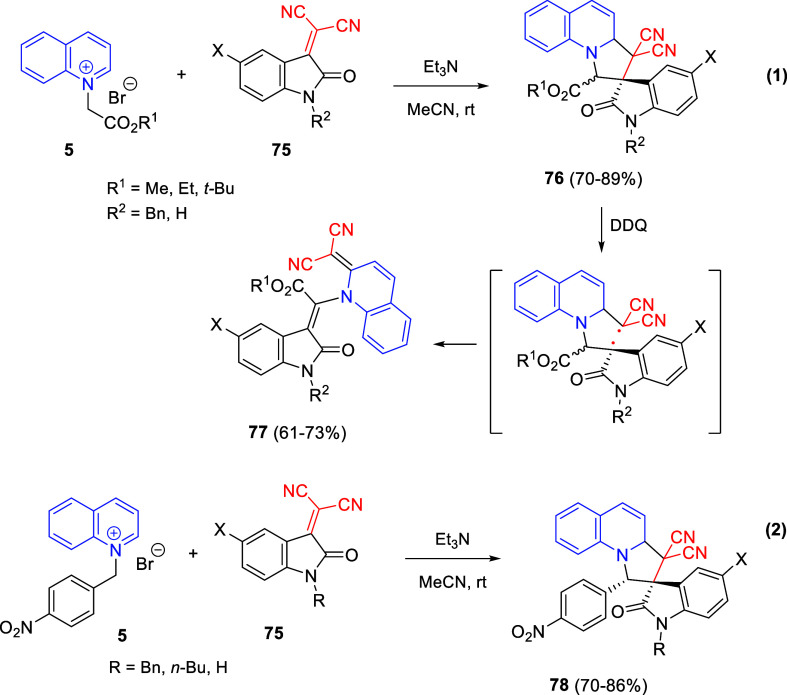
1,3-Dipolar Cycloaddition of Quinolinium Ylides with Isatylidene Malononitriles

In 2017, Sun, Yan, and co-workers reported a base-promoted reaction of quinolinium bromides **5** with isatylidene malononitriles **75** (Scheme 57).^93^ The corresponding quinolinium ylides, *in situ* formed with triethylamine, underwent the (3 + 2) cycloaddition reaction with the dicyanoalkene moiety to render spiro[indoline-3,2′-pyrrolo[1,2-*a*]quinolines] **76** in good yields as nearly equimolecular mixtures of diastereoisomers. These cycloadducts proved to be unstable and they were further treated with 2,3-dichloro-5,6-dicyano-1,4-benzoquinone (DDQ). In this manner, the less stable C–C bond was broken to give a diradical intermediate, which evolved to the final products **77** by dehydrogenation (Scheme 57, eq 1).

This 1,3-dipolar cycloaddition reaction was extended to *N*-(4-nitrobenzyl) quinolinium bromide as the ylide precursor. Its reaction with isatylidene malononitriles **75** provided spiro[indoline-3,2′-pyrrolo[1,2-*a*]quinolines] **78** as single diastereoisomers in good yields. The high diastereoselectivity observed in this case might be conferred to the large steric effect of the 4-nitrobenzyl group (Scheme 57, eq 2).

**Scheme 58:**
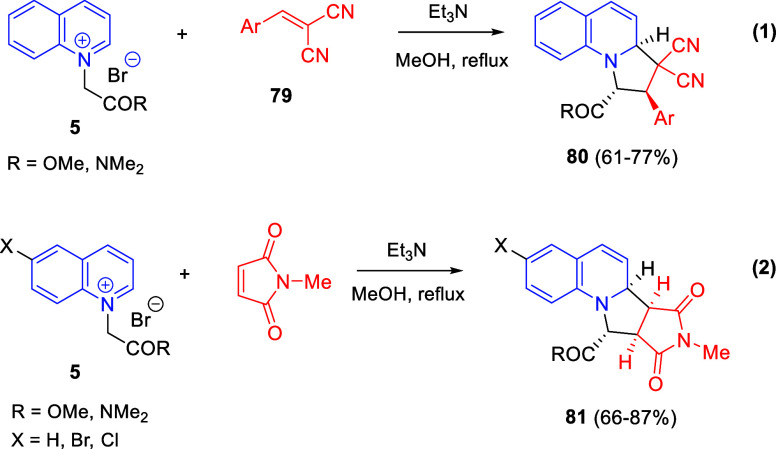
1,3-Dipolar Cycloaddition Reaction of Quinolinium Ylides with Arylidene Malononitriles and Maleimide

A similar transformation was evaluated by Coldham and co-workers in 2019. It involved the reaction of quinolinium bromides **5** bearing an ester or amide group with arylidene malononitriles **79** in the presence of trimethylamine (Scheme 58).^94^ After deprotonation of the quinolinium salts, the *in situ* generated quinolinium ylides underwent (3 + 2) dipolar cycloaddition with the electron-poor alkenes to afford pyrrolo[1,2-*a*]quinolines **80** as single regio- and diasteroisomers in good yields (Scheme 58, eq 1). The reaction was further extended to *N*-methyl maleimide as dipolarophile, rendering tetracyclic pyrroloquinolines **81**, again as single diastereoisomers in very good yields (Scheme 58, eq 2). In addition, when the starting materials were functionalized with a Cl or Br atom, the final products were suitable substrates for a subsequent Suzuki coupling, thus expanding the scope of the process.

**Scheme 59:**
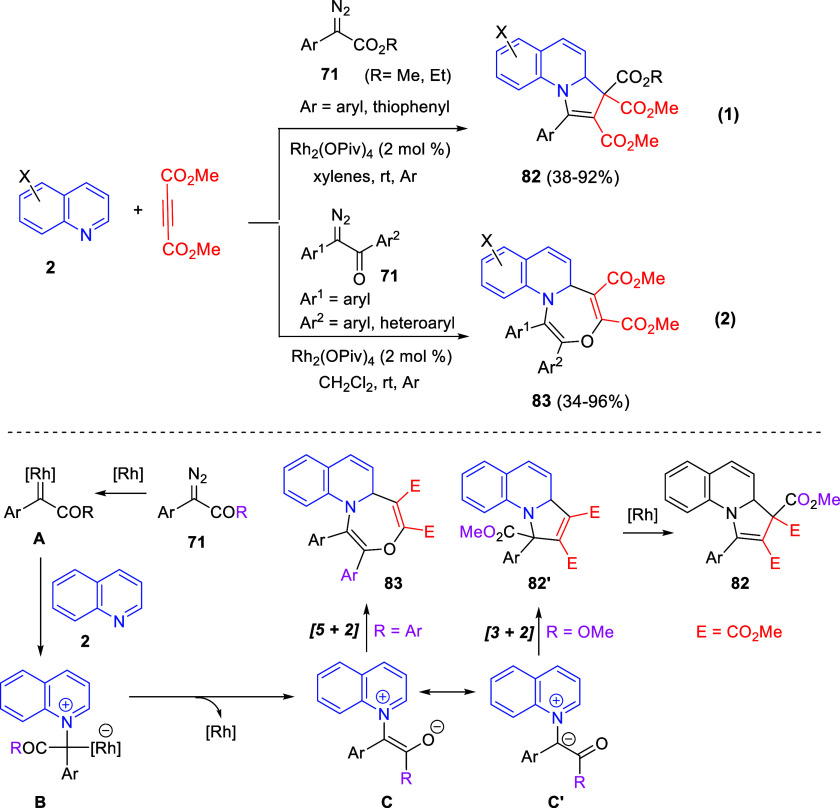
Rhodium-Catalyzed Regiodivergent (3 + 2) and (5 + 2) Cycloadditions of Quinolinium Ylides with Alkynes

Peng and co-workers employed diazocompounds under rhodium catalysis to generate quinolinium ylides that reacted with electron-deficient alkynes as dipolarophiles (Scheme 59).^95^ The authors were able to perform regiodivergent (3 + 2) and (5 + 2) cycloadditions depending on the type of donor–acceptor diazocompound. Thus, the multicomponent reaction of quinolines **2**, aryl diazoacetates **71** and dimethyl acetylendicarboxylate provided (3 + 2) indolizidine derivatives **82**, with 1,3-ester migration, in moderate to excellent yields (Scheme 59, eq 1). Electron-donating and electron-withdrawing groups were tolerated on the aryl ring of diazoacetates, as well as at the C6- and C7-positions of the quinoline counterpart. When α-diazoketones were employed as substrates, the corresponding (5 + 2) cycloaddition with the electron-deficient alkyne took place, leading to 1,4-oxazepine derivatives **83** in good yields (Scheme 59, eq 2). The reaction was compatible with a variety of diazoketones bearing electronically different substituents on both aryl rings, and also with heteroaryl moieties.

Regarding the mechanism of this transformation, the authors proposed that the reaction of the rhodium complex with the diazocompound would generate the metallacarbene species **A**, with elimination of N_2_. Then, the nucleophilic addition of quinoline **2** would render intermediate **B**, which would dissociate the rhodium salt to form quinolinium ylides **C** and **C′**. At this point, intermediate **C** would react with the alkyne in a 1,5-dipolar (5 + 2) cycloaddition process to afford oxazepines **83**; while quinolinium ylide **C′** would undergo 1,3-dipolar (3 + 2) cycloaddition with the alkyne to give compound **82′**, which could be transformed into the final indolizidines **82** by means of an unusual rhodium-catalyzed 1,3-ester migration process (Scheme 59).

**Scheme 60:**
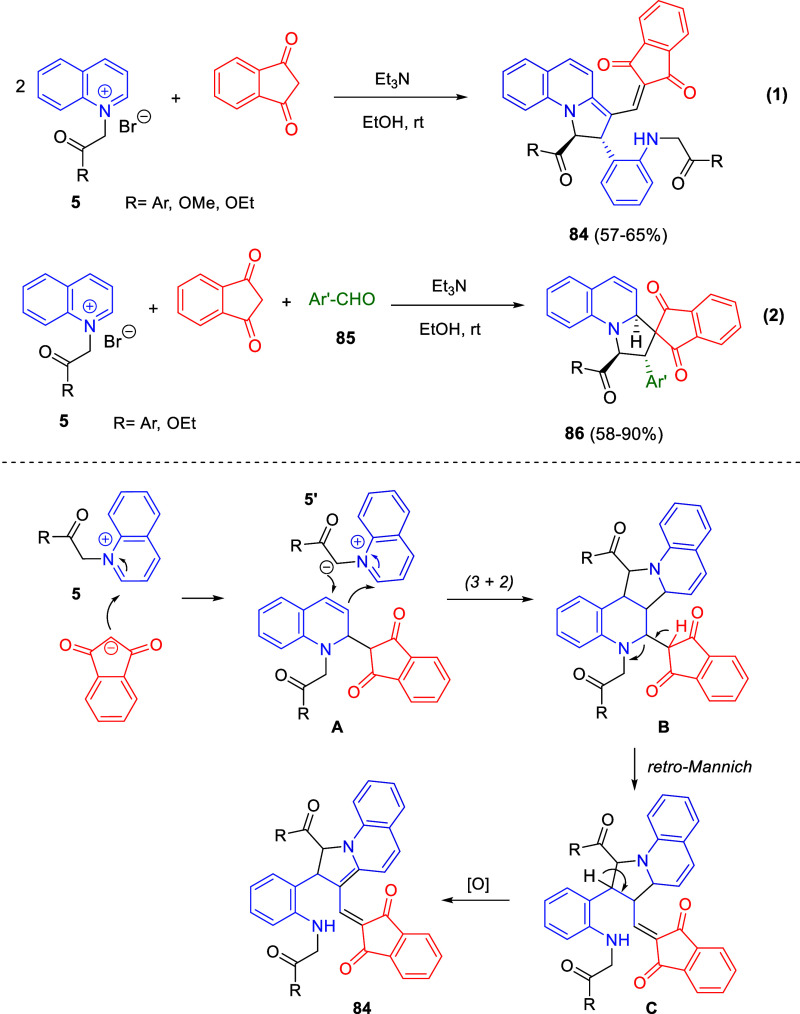
Cycloaddition Reaction of Quinolinium Ylides with 1,3-Indanedione and 2-Arylidene-1,3-indanediones

In 2020, Yan and co-workers described the base-promoted reaction of quinolinium salts **5** (2 equiv) with 1,3-indanedione to mainly render functionalized dihydropyrrolo[1,2-*a*]quinolines **84**, in which one quinoline ring was opened and the other one was incorporated into the final compound (Scheme 60, eq 1).^96^ On the other hand, the three-component reaction of quinolinium salts **5**, 1,3-indanedione and aromatic aldehydes **85** in the presence of trimethylamine afforded spiropyrroloquinolines **86** in good yields (Scheme 60, eq 2). Electron-withdrawing and electron-donating substituents were tolerated on the aldehyde as well as on the quinolinium salt.

The reaction would start with the addition of deprotonated 1,3-indanedione to the C2-position of the quinolinium salt **5**. Then, a (3 + 2) cycloaddition process between the *in situ* generated quinolinium ylide **5′** and the dihydroquinoline intermediate **A** would take place, followed by ring-opening through a retro-Mannich-type reaction and partial oxidation in air to furnish polycyclic products **84** (Scheme 60). In the presence of the aromatic aldehyde, a Knoevenagel-type condensation would generate 2-arylidene-1,3-indanedione derivatives, which would undergo the subsequent 1,3-dipolar cycloaddition with the quinolinium ylides to afford spirocompounds **86**.

### 3.3 Isoquinolinium Ylides

Despite the first example of a dipolar cycloaddition reaction with isoquinolinium ylides as 1,3-dipoles was reported more than six decades ago, their interest as a useful tool in the synthesis of pyrrolidine-containing polyheterocycles is still today an intense area of research.

**Scheme 61:**
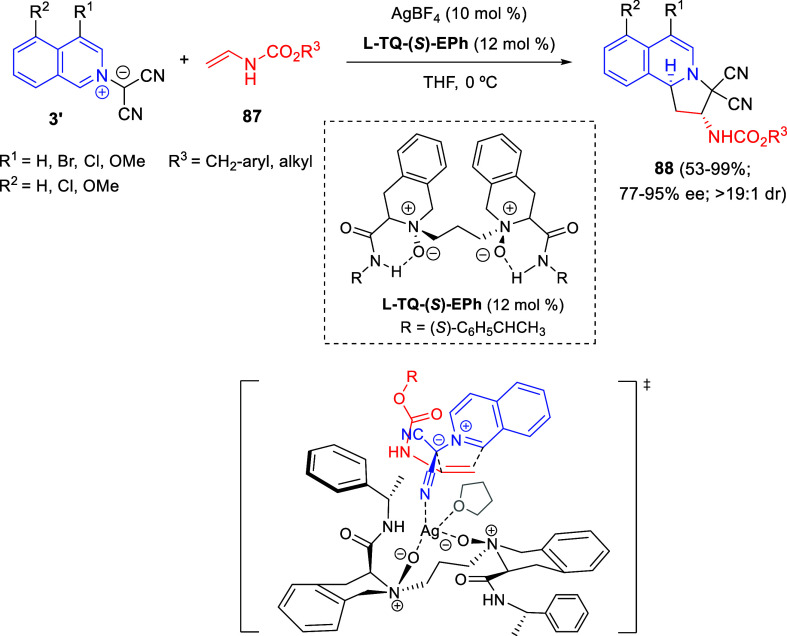
Enantioselective Inverse-Electron Demand 1,3-Dipolar Cycloaddition of Isoquinolinium Methylides with Enecarbamates

In 2016, Feng and co-workers developed an enantioselective inverse-electron demand 1,3-dipolar cycloaddition of isoquinolinium methylides **3′** with enecarbamates **87** (Scheme 61).^97^ This reaction was catalyzed by a chiral *N*,*N*′-dioxide complex of Ag(I), *in situ* generated from AgBF_4_ and the chiral ligand **L-TQ-(***S***)-EPh** bearing tetrahydroisoquinoline and (*S*)-phenylethanamine motifs. After 24 h in THF at 0 °C, chiral pyrroloisoquinolines **88** were obtained in high yields, excellent diastereoselectivity and good to excellent ee values. In general, enecarbamates **87** with electron-withdrawing substituents on the benzyl group (R^2^ = CH_2_-aryl) provided best results in terms of yield and enantiocontrol. Alkyl substituents at the carboxyl group (R^2^ = alkyl) were also tolerated, although the enantioselectivity decreased with the hindered *tert*-butyl group. Regarding the isoquinoline counterpart, electronically different substituents were tolerated at the C4- and C5-positions. In addition, phthalazinium dicyanomethylide, with two nitrogen atoms on the ring, was a suitable dipole for this reaction.

According to different experiments, the authors proposed a reasonable transition state to explain the stereochemical outcome of the reaction. Thus, Ag(I) would form a tetrahedral intermediate with one cyano group of the isoquinolinium methylide, the two oxygen atoms of the chiral *N*,*N*′-dioxide ligand and a molecule of THF. In this organized arrangement, the *Re* face of the ylide would be shielded by the (*S*)-phenylethyl moiety on the left-hand side, producing the addition of the dipole *Si* face to the enecarbamate (Scheme 61).

**Scheme 62:**
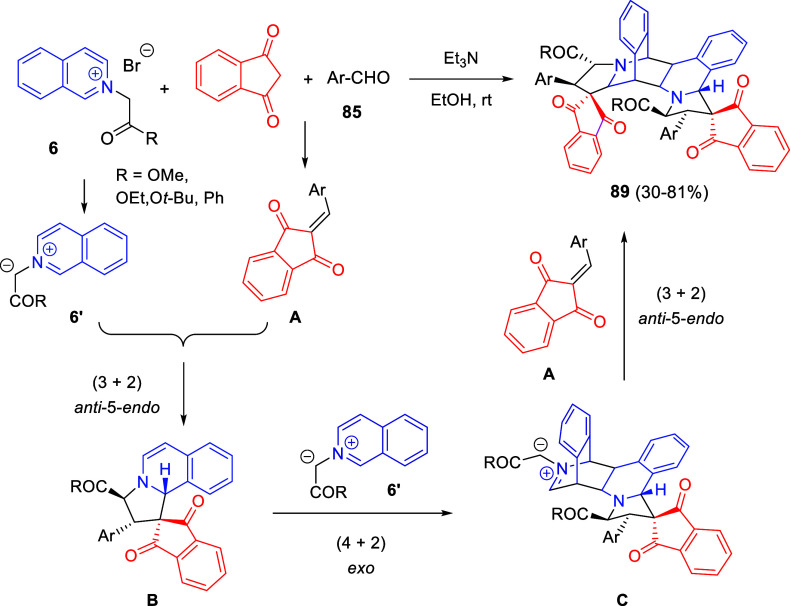
(3 + 2)-(4 + 2)-(3 + 2) Cycloaddition Sequence of Isoquinolinium Ylides with Aromatic Aldehydes and Indan-1,3-dione

Liu, Yan, and co-workers disclosed a unique (3 + 2)-(4 + 2)-(3 + 2) cycloaddition sequence in a three-component reaction involving isoquinolinium bromides **6**, 1,3-indanedione and aromatic aldehydes **85** in the presence of Et_3_N as a base (Scheme 62).^98^ This reaction afforded polycyclic products **89** with ten stereogenic centers as single diastereoisomers in moderate to good yields. The electronic nature of the substituents on the aromatic aldehydes did not affect the domino process. On the other hand, *N*-alkoxyl isoquinolinium salts **6** (R = OMe, OEt, O*t*-Bu) were suitable substrates, while when a phenacyl group was used (R = Ph), yields decreased significatively.

A mechanistic rationale for this transformation was proposed by the authors, starting from the generation of the isoquinolinium ylide **6′** under basic conditions and the 2-arylidene-1,3-indanedione **A** as the dipolarophile. These two intermediates would undergo a (3 + 2) cycloaddition in an *anti*-5-*endo* mode to achieve spiropyrroloisoquinoline **B**, with three setereogenic centers. Then, a (4 + 2) cycloaddition between the 2-azadiene moiety of a second isoquinolinium ylide and the enamine portion of intermediate **B** acting as the dienophile would afford polycyclic ylide **C**. Finally, another (3 + 2) cycloaddition with a second 2-arylidene-1,3-indanedione **A** would lead to the final products **89** (Scheme 62). It is worth noting that the isoquinolinium salt has a triple role in this process: as a 1,3-dipole, an electron-rich dienophile and an electron-deficient diene.

**Scheme 63:**
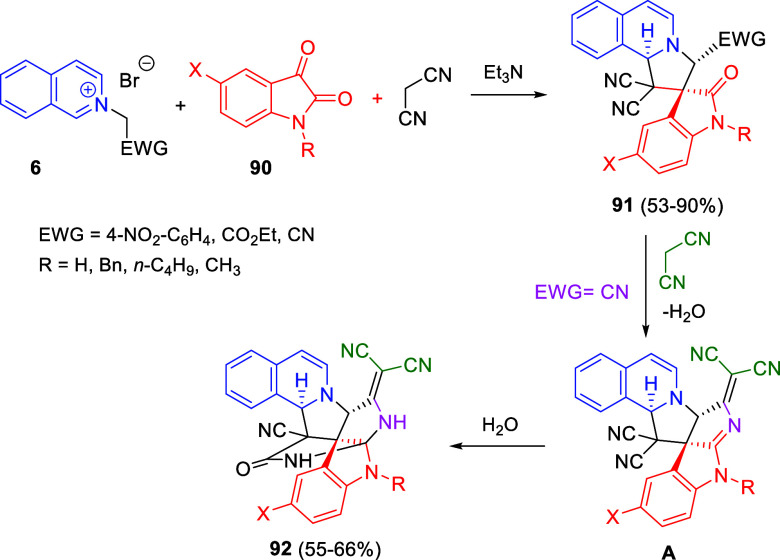
Three-Component Reaction of Isoquinolinium Salts, Isatins, and Malononitrile

Again, Yan and co-workers described a three-component reaction of isoquinolinium salts **6**, isatins **90**, and malononitrile (Scheme 63).^99^ Initial base-promoted condensation of malononitrile with isatines would generate the corresponding isatylidene malononitriles, which would be the dipolarophiles in the (3 + 2) cycloaddition with the *in situ* generated isoquinolinium ylides by deprotonation of the isoquinolinium salts **6**. In this manner, spiro[indoline-3,2′-pyrrolo[2,1-*a*]isoquinolines] **91** were synthesized in good yields and with high diastereoselectivity. The electronic nature of substituents on the isatin moiety did not affect the process. The reaction with *N*-cyanomethylisoquinolinium chloride **6** (R = CN) led to complex polycyclic compounds **92** in moderate yields. In this case, after the 1,3-dipolar cycloaddition, a second equivalent of malononitrile would be added to the spiro compound **91** to afford intermediate **A**. Upon hydrolysis, the intramolecular attack of one cyano group to the cyclic imine functionality, would render final compounds **92**.

**Scheme 64:**
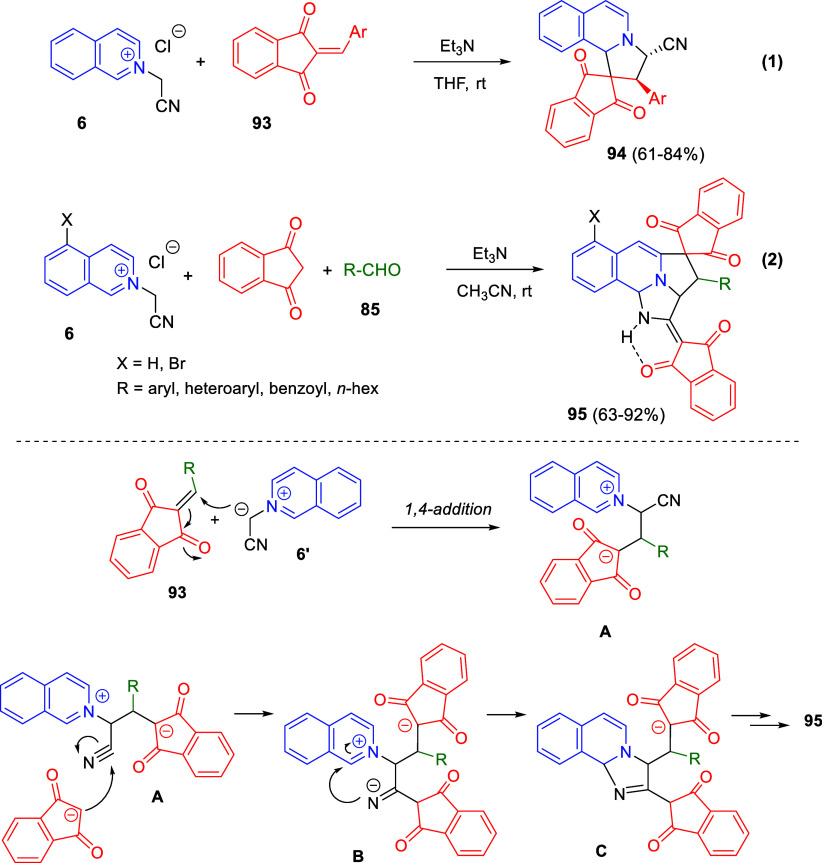
Tandem Double (3 + 2) Cycloaddition Reactions of *N*-Cyanomethylisoquinolinium Ylides

The same group reported the base-promoted cycloaddition reaction of *in situ* generated isoquinolinium ylides from *N*-cyanomethylisoquinolinium chloride with 2-arylidene-1,3-indanediones **93** in dry THF at room temperature to render spiro[indene-2,1′-pyrrolo[2,1-*a*]isoquinoline] derivatives **94** in good yields as single diastereoisomers (Scheme 64, eq 1).^100^ The authors envisioned the possibility of performing the reaction in a multicomponent manner starting from 1,3-indanedione and aromatic aldehydes **85**. However, in this case, different reaction products with incorporation of two scaffolds of 1,3-indanedione (**95**) were isolated in good yields, starting from a variety of aldehydes. The optimized reaction conditions involved the use of acetonitrile at room temperature in the presence of Et_3_N (Scheme 64, eq 2).

The proposed mechanism for the synthesis of these polycyclic spirocompounds **95** would involve first, the base-promoted generation of isoquinolinium ylide **6′** and the 2-arylidene-1,3-indanediones **101** by a Knoevenagel-type condensation. The conjugate addition between them would afford zwitterionic intermediate **A** (Scheme 64). Then, the nucleophilic addition of the carbanion of 1,3-indanedione to the cyano group followed by cyclization onto the cyclic iminium ion would lead to intermediate **B**. Finally, addition of the resulting enolate to the enamine moiety and tautomerization would give the observed final products.

**Scheme 65:**
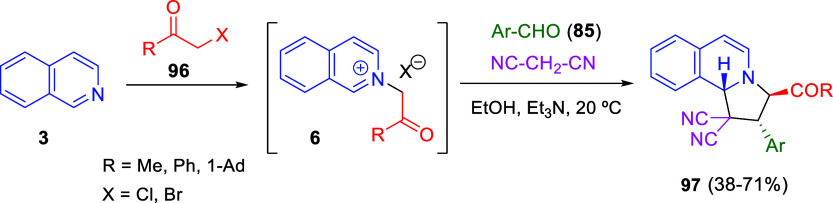
One-Pot Four-Component Synthesis of Benzanulated Indolizidine Scaffolds

Zubarev and co-workers developed a one-pot procedure for the synthesis of benzannulated indolizidine scaffolds by means of the reaction of isoquinoline (or quinoline), α-halocarbonyl compounds, aromatic aldehydes and malononitrile (Scheme 65).^101^ Under the reaction conditions, isoquinolinium salts **6** were prepared *in situ* by heating isoquinoline with the α-halo carbonyl compounds **96**. Then, deprotonation with triethylamine generated the corresponding isoquinolinium ylides. On the other hand, malononitrile condensed with the aromatic aldehydes **85** and the resulting dicyanoalkenes underwent a dipolar (3 + 2) cycloaddition to afford the final tetrahydroindolizidine derivatives **97** in moderate to good yields as single diastereoisomers.

**Scheme 66:**
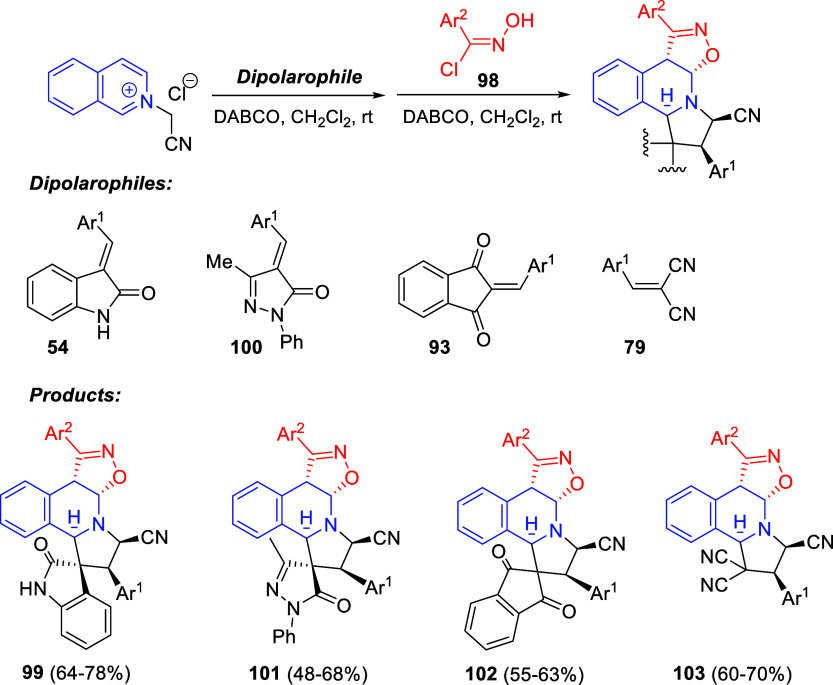
Diastereoselective Synthesis of Spirocyclic Isoxazolo[5,4-*c*]pyrrolo[2,1-*a*]isoquinolines via Cascade Double (3 + 2) Cycloadditions

Sun, Yan, and co-workers accomplished two consecutive (3 + 2) dipolar cycloaddition reactions of *N*-cyanomethylisoquinolinium ylide with several dipolarophiles and *N*-hydroxybenzimidoyl chlorides (Scheme 66).^102^ First, the authors performed the reaction of (*E*)-3-arylideneindolin-2-ones **54** with *N*-cyanomethylisoquinolinium chloride in the presence of DABCO as a base. Once the first (3 + 2) cycloaddition was completed, *N*-hydroxybenzimidoyl chlorides **98** and more DABCO were added, and a second cycloaddition with the *in situ* generated nitrile oxide took place, affording polycyclic spiropyrroloisoquinoline derivatives **99** in good overall yields and with high diastereoselectivity. This cascade double (3 + 2) cycloaddition procedure was extended to other dipolarophiles such as 4-arylidene-5-methyl-2-phenylpyrazol-3-ones **100**, 2-arylidene-1,3-indanediones **93**, and arylidene malononitriles **79** (Scheme 66). In general, the electronic nature of both aryl groups present in the substrates had little influence on the yields of the final polycyclic products.

**Scheme 67:**
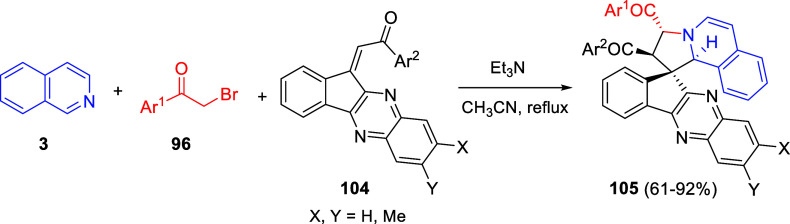
Regio- and Stereoselective Three-Component (3 + 2) Cycloaddition Reaction of Isoquinolinium Ylides and Indeno-quinoxaline Derivatives

In 2020, Moghaddam and co-workers reported a one-pot three-component reaction involving a 1,3-dipolar cycloaddition of isoquinolinium ylides, generated *in situ* through the reaction of isoquinoline with phenacyl bromides **96**, with 1-aryl-2-(11H-indeno[1,2-*b*]quinoxalin-11-ylidene)-ethanones **104** as dipolarophiles (Scheme 67).^103^ This reaction allowed the authors to obtain spiroheterocycles **105**, containing both pyrrolo[2,1-*a*]isoquinoline and quinoxaline moieties, in moderate to good yields and excellent diastereoselectivities. A wide variety of substituents were tolerated on the starting quinoxalines and phenacyl bromides. It is important to mention that this dipolar cycloaddition between the *in situ* generated isoquinolinium ylides and the quinoxaline derivatives was completely regioselective, with the initial addition taking place to the α-position of the conjugated ketone in the quinoxaline derivative.

**Scheme 68:**
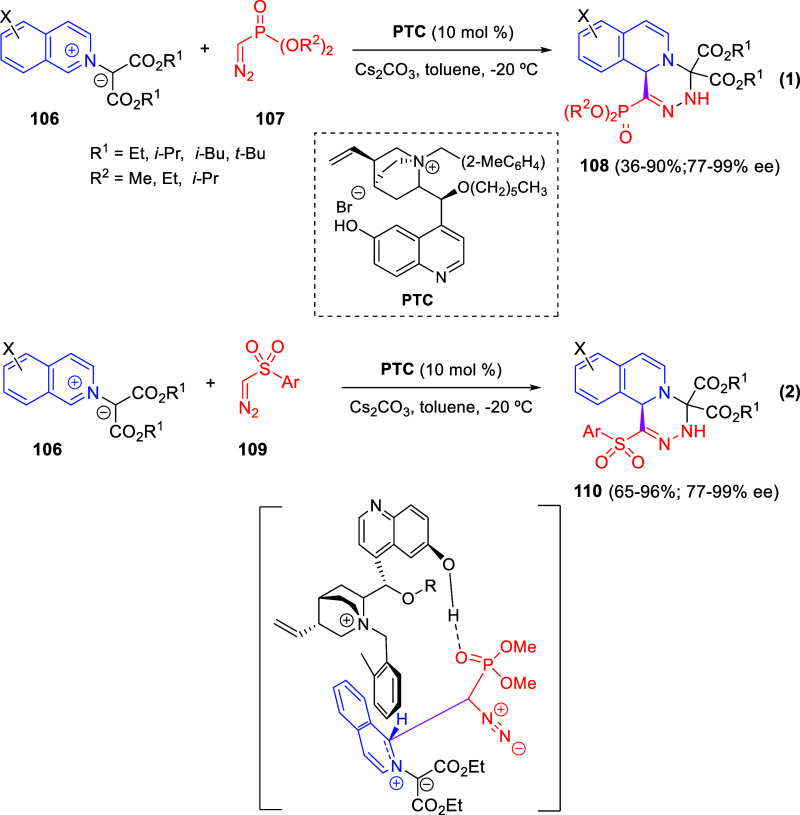
Enantioselective (3 + 3) Cycloaddition Reaction of Diazocompounds with Isoquinolinium Methylides

Recently, Peng, Ouyang, and co-workers reported the reaction of isoquinolinium methylides with diazocompounds under phase transfer catalysis (Scheme 68).^104^ The authors found a bifunctional chiral phase-transfer catalyst (PTC) capable of promoting the enantioselective (3 + 3) cycloaddition reaction of isoquinolinium methylides **106** with α-(diazomethyl)phosphonates **107** to render [1,2,4]triazino[5,4-*a*]isoquinoline derivatives **108** in moderate to high yields with excellent enantioselectivities (Scheme 68, eq 1). A variety of ester groups both on the phosphonate and the ylide were tolerated in the process, although the reaction with *tert*-butyl α-(diazomethyl)phosphonate proceeded in low yield. Moreover, substituents at the C5-, C6-, and C7-positions of the starting isoquinolinium ylides were compatible with the reaction, independently of their electronic properties. This protocol was successfully extended to diazosulfones **109** to yield triazino[5,4-*a*]hydroisoquinoline sulfones **110** (Scheme 68, eq 2).

Based on the experimental work and DFT computational calculations, the authors proposed a plausible catalytic cycle for this asymmetric (3 + 3) cycloaddition reaction. Within this mechanism, the hydroxyl group of the phase-transfer catalyst would be deprotonated and it would act as a base, removing the α-hydrogen of the diazomethylphosphonate (Scheme 68). Coordination of the catalyst hydroxyl group with the phosphonate would deliver the nucleophilic addition to the *Si* face of the isoquinolinium ylide. Further addition of the ylide to the diazo moiety would complete the cycloaddition process.

### 3.4 *N*-Imino-pyridinium, -quinolinium, and -isoquinolinium Ylides

Pyridinium, quinolinium, and isoquinolinium imides are a special class of azomethine imines. These dipoles have been used in the synthesis of a wide variety of heterocycles that preserve the aromaticity of the heterocycles. However, their use in cycloaddition reactions have not been explored much due to the energy barrier needed for the dearomatization of the heterocycles. The examples found since 2016 are summarized below.

**Scheme 69:**
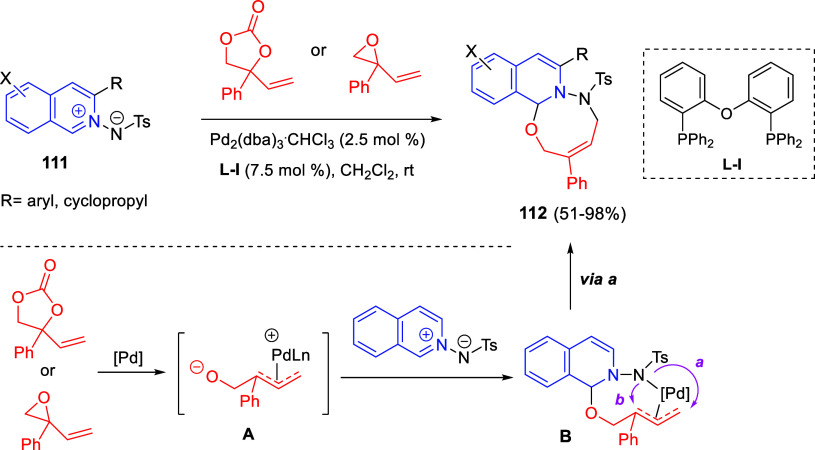
Formal (5 + 3) Cycloaddition of Zwitterionic Allylpalladium Intermediates with Azomethine Imines

In 2018, Guo and co-workers described a formal (5 + 3) cycloaddition reaction of zwitterionic allylpalladium intermediates, catalytically generated from vinylethylene carbonates or vinyloxiranes, with 1,3-dipoles such as *N*-iminoisoquinolinium ylides **111** (Scheme 69).^105^ Differently substituted azomethine imines and dipolarophiles allowed the synthesis of a variety of *N*,*O*-containing eight-membered heterocyclic compounds **112** in good yields with excellent regioselectivity.

In the presence of the Pd(0) catalyst, vinylethylene carbonates or vinyloxiranes would generate the zwitterionic allylpalladium intermediates **A** through decarboxylation or ring-opening, respectively (Scheme 69). Then, nucleophilic addition to azomethine imines **111** would produce allylpalladium intermediates **B**, which could evolve through two alternative pathways, leading to the formal (3 + 3) or (5 + 3) cycloadducts. Experimental results showed that this *N*-alkylation reaction was highly regioselective, rendering the eight-membered rings **112** as the major products (Scheme 68, ***via a***). After DFT calculations, the authors found two key points that promoted this cyclization, i.e. the presence of a phenyl ring in the starting precursors of the organopalladium species and the phosphine ligand (**L-I**) employed. Under the reaction conditions, the formation of products **112** would be thermodynamically controlled, while the formation of the regioisomer, in the absence of the phenyl ring, would be consequence of kinetic control.

**Scheme 70:**
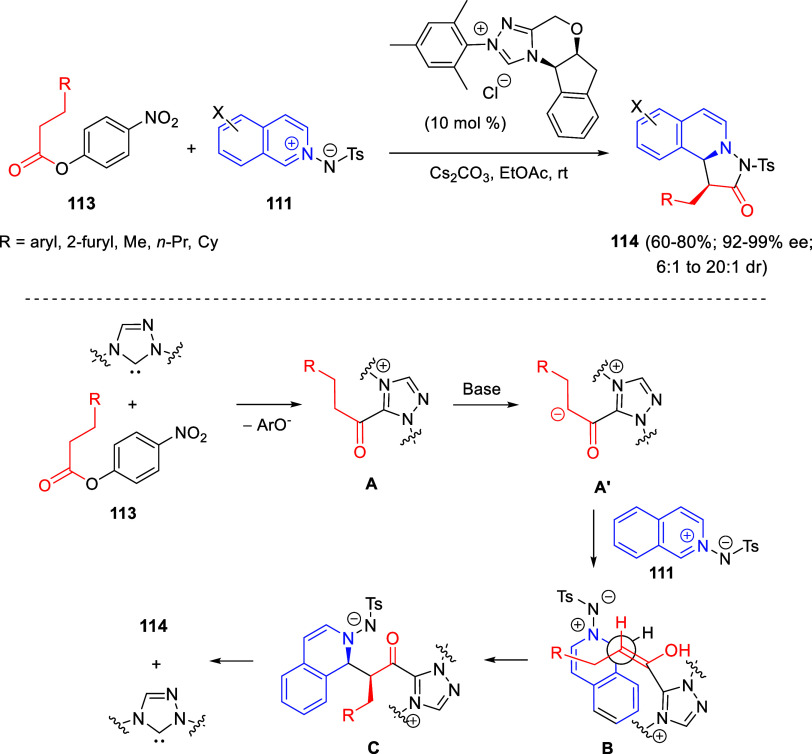
NHC-Catalyzed Enantioselective (3 + 2) Dearomatizing Annulation of Saturated Carboxylic Esters with *N*-Iminoisoquinolinium Ylides

The same year, Liu, Xu, and co-workers reported an enantioselective synthesis of tricyclic dihydroisoquinoline derivatives by means of a chiral NHC-catalyzed (3 + 3) dearomatizing annulation reaction between *p*-nitrobenzoate esters **113** and *N*-iminoisoquinolinium ylides **111** (Scheme 70).^106^ In the presence of an imidazolium salt as the NHC precatalyst and Cs_2_CO_3_ as the base, tricyclic chiral products **114** were achieved in good yields with excellent enantio- and diastereoselectivities. Different substituents on the ester counterpart were tolerated, although the introduction of alkyl groups led to lower yields of the final products. On the other hand, electron-withdrawing substituents on the iminoisoquinolinium ylide were tolerated, while electron-donating ones were compatible only at the C8-position.

The mechanism proposed by the authors to explain this reaction would start with the addition of the NHC catalyst to the ester substrate **113** to give the acylazolium intermediate **A** (Scheme 70). Its deprotonation would form enolate **A′**, which would react with the *N*-iminoisoquinolinium ylide **111**, effecting the dearomatization of the isoquinoline ring through intermediate **B**. Final intramolecular cyclization would release product **114** and regenerate the NHC catalyst.

**Scheme 71:**
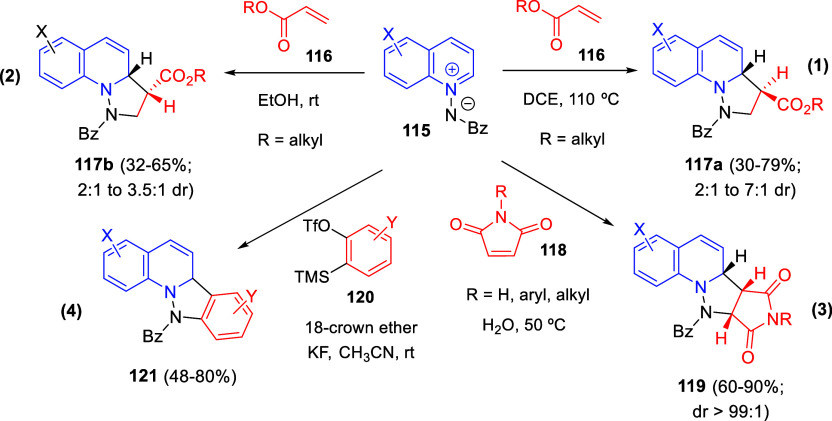
Diastereoselective 1,3-Dipolar Cycloaddition of Quinolinium Imides with Acrylates, Maleimides, and Benzynes

Sharma and co-workers developed the (3 + 2) cycloaddition reaction of quinolinium imides with activated olefins for the synthesis of various fused *N*-heterocyclic compounds (Scheme 71).^107^ The reaction of *N*-benzoyliminoquinolinium ylides **115** with a variety of acrylates **116** in dichloroethane at 110 °C afforded the *trans*-tetrahydropyrazoloquinolines **117a** as the major products in good yields and moderate to good diastereoselectivities (Scheme 71, eq 1). Interestingly, the authors found that when the reaction was performed in ethanol at room temperature, *cis*-*N*,*N*′-heterocyclic compounds **117b** were obtained as the major products in low to good yields and moderate diastereoselectivities (Scheme 71, eq 2). Next, the reaction was extended to maleimides **118** as dipolarophiles, leading to tetracyclic *N*-heterocyclic products **119** in good yields, excellent *cis*-diastereoselectivities and complete *endo* selectivity (Scheme 71, eq 3). Furthermore, when benzyne precursors **120** were employed, the corresponding (3 + 2) adducts **121** were also obtained in good yields (Scheme 71, eq 4). In all cases, different substituents on the starting quinolinium imides were tolerated.

**Scheme 72:**
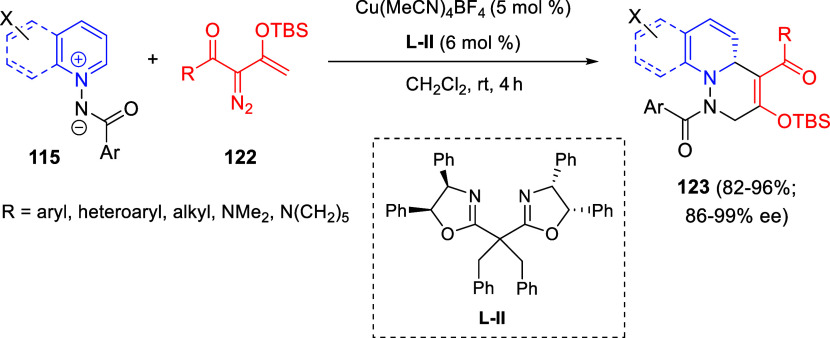
Enantioselective Copper(I)-Catalyzed Reaction of Enoldiazo-ketones and -amides with Acyliminopyridinium Ylides

In 2018, the group of M. P. Doyle described an enantioselective (3 + 3) cycloaddition reaction of enoldiazocarbonyl compounds with acyliminopyridinium, quinolinium and isoquinolinium ylides catalyzed by copper(I) with a chiral bisoxazoline (box) ligand (Scheme 72).^108^ Thus, pyridinium and quinolinium imides **115** reacted with enoldiazoketones and amides **122** in the presence of Cu(MeCN)_4_BF_4_ as the catalyst and chiral box ligand **L-II** to render pyrazinodihydropyridines and pyrazinodihydroquinolines **123** in very good yields and excellent ee values. Different substitution on either the benzoyl group of the ylide or the enoldiazocarbonyl compound had no effect in terms of chemical yields and enantioselectivities. However, the cycloaddition with benzoyliminoisoquinolinuium ylide proceeded with low enantioselectivity (58% ee).

**Scheme 73:**
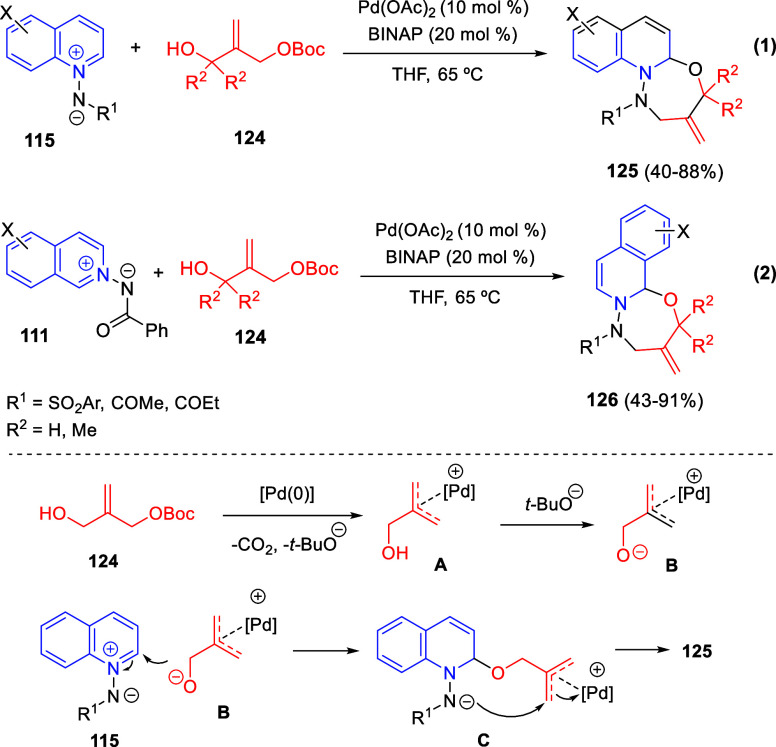
Palladium-Catalyzed (4 + 3) Dearomatizing Cycloaddition Reaction of *N*-Imino-quinolinium and -isoquinolinium Ylides

Liu, Chen, and co-workers disclosed a palladium-catalyzed (4 + 3) dearomatizing cycloaddition reaction of *N*-imino-quinolinium and -isoquinolinium ylides and 2-(hydroxymethyl)allyl *t*-butyl carbonates (Scheme 73).^109^ On the one hand, allyl carbonates **124** reacted with *N*-iminoquinolinium ylides **115** in the presence of Pd(OAc)_2_ and BINAP to render dihydroquinoline-fused seven membered rings **125** in good yields (Scheme 73, eq 1). Starting ylides **115** bearing electron-withdrawing groups afforded the corresponding products in higher yields compared to those with electron-donating groups. In addition, C6-substituted quinolinium ylides gave higher yields of the desired products than those substituted at the C5-position. Regarding the substituent attached to the nitrogen, best results were obtained with aryl sulfones, while the yields decreased with acetyl and propionyl moieties. On the other hand, the cycloaddition reaction with *N*-iminoisoquinolinium ylides **111** also provided the desired cycloadducts **126** in good yields (Scheme 73, eq 2).

The authors proposed a plausible mechanism for this palladium-catalyzed (4 + 3) cycloaddition, which would start by the oxidative addition of Pd(0) to allyl carbonates **124** to form a π-allyl palladium intermediate **A**, carbon dioxide, and a *tert*-butoxy anion (Scheme 73). This would act as a base and deprotonate the hydroxyl group to form dipole **B**. Subsequent attack of the oxygen anion to the 2-position of the quinolinium ring would effect its dearomatization and then, the addition of the nitrogen anion of intermediate **C** to the π-allyl palladium moiety would deliver the final product by intramolecular cyclization.

### 3.5 *N*-Heteroaromatic Zwitterions

**Scheme 74:**
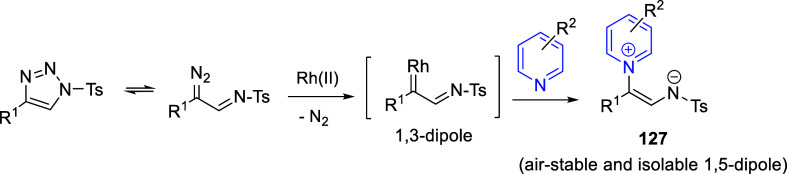
Rh(II)-Catalyzed Formation of Isolable Azomethine Ylides

Higher-order cycloadditions, i.e. those proceeding with the participation of more than 6π electrons, allow for the generation of complex molecular scaffolds from readily available starting materials in a predictable manner.^110−112^ However, important regioselectivity issues have to be overcome since those transformations require starting substrates bearing extensive insaturation and they can participate in several different reactions at the same time. In the recent years, a great number of higher-order cycloaddition reactions have emerged in the literature, and the most representative examples are those involving the use of *N*-heteroaromatic zwitterions as 1,5-dipoles. The key for the successful preparation of these 1,5-dipoles took advantage of the Rh(II)-catalyzed ring opening of *N*-sulfonyl 1,2,3-triazoles to form α-azavinyl Rh(II) carbenoids.^113^ In 2014, You and co-workers found that Rh-azavinyl carbenes react with pyridines to generate air-stable and isolable azomethine ylides **127** that can act as 1,5-dipoles (Scheme 74).^114^ These dipoles undergo (5 + 2) cycloaddition reactions under mild conditions with a wide variety of activated 2π dipolarophiles, despite those reactions involve the thermodynamically unfavorable dearomatization step.^115,116^

**Scheme 75:**
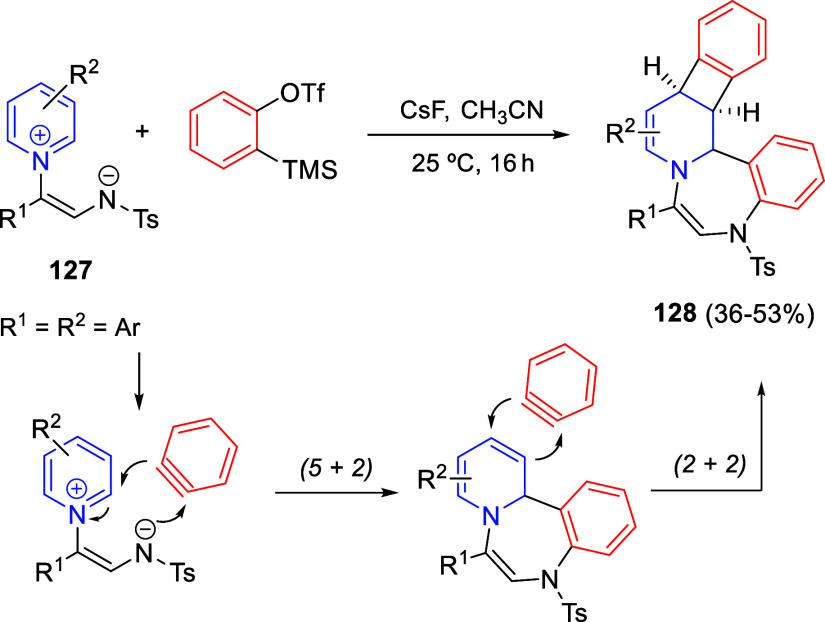
Synthesis of Fused Polycyclic 1,4-Benzodiazepines via Cascade (5 + 2)/(2 + 2) Cycloadditions

In 2017, the group of Yoo described the (5 + 2) cycloaddition reaction between pyridinium zwitterions **127**, acting as 1,5-dipoles, and arynes for the synthesis of polycyclic 1,4-benzodiazepines, which further underwent (2 + 2) cycloaddition in a cascade process (Scheme 75).^117^ The use of two equivalents of 2-(trimethylsilyl)phenyl triflate in the presence of CsF as the *in situ* promoter of the benzyne formation led the authors to isolate pentacyclic benzodiazepines **128** in moderate to good yields.

**Scheme 76:**
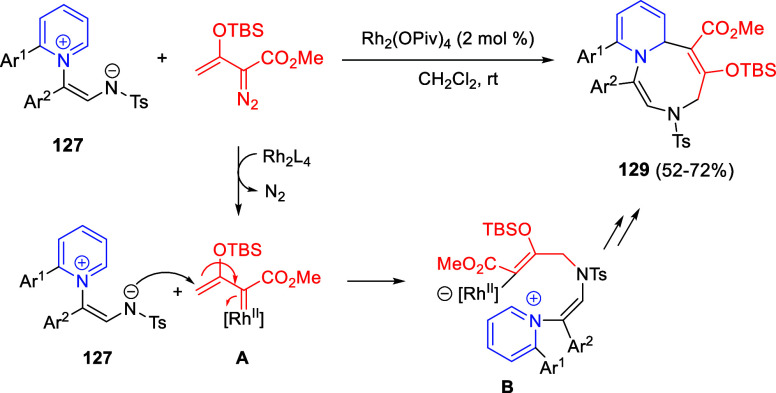
Rh(II)-Catalyzed (5 + 3) Cycloaddition of Pyridinium Zwitterions and Enol Diazoacetates

**Scheme 77:**
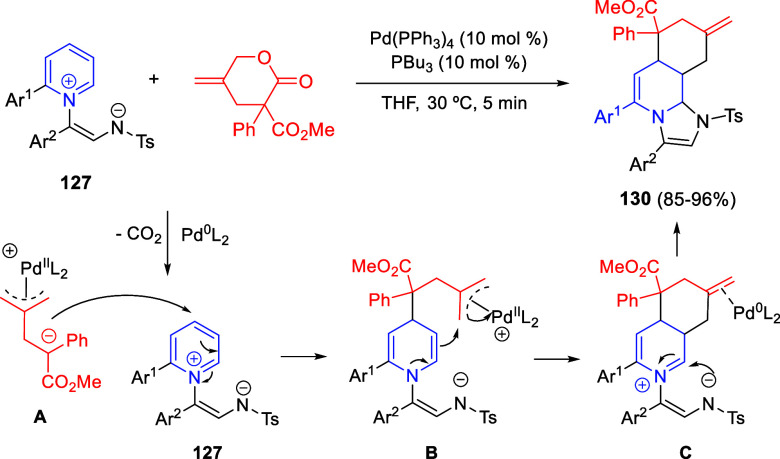
Pd(0)-Catalyzed (4 + 2) Cycloaddition of Pyridinium Zwitterions and γ-Methylidene-δ-valerolactone

The same authors demonstrated the regiodivergent behavior of pyridinium zwitterions **127** with two different types of transition metal catalysts (Schemes 76 and 77). Thus, compounds **127** can act as nucleophilic 1,5-dipoles and be engaged in (5 + 3) cycloaddition reactions when the reaction partner is an electrophile, while they react through the 4-position of the pyridine ring with nucleophilic partners, in a formal (4 + 2) cycloaddition after subsequent ring closure.

On the one hand, Yoo and co-workers had reported the rhodium(II)-catalyzed (5 + 3) cycloaddition between pyridinium zwitterions **127**, acting as nucleophilic 1,5-dipoles, and enol diazoacetates to afford eight-membered *N*-heterocycles **129** in good yields (Scheme 76).^115^ Regarding the mechanism of this transformations, DFT calculations showed that the pyridinium zwitterion would attack the vinylogous position of the *in situ* generated rhodium(II)enol carbenoid **A**, acting as an electrophile.^118^ The resulting intermediate **B** would undergo an intramolecular cyclization to end up with the final bicyclic products **129** (Scheme 76).

On the other hand, the authors investigated the reaction of pyridinium zwitterions **127** with palladium-bound zwitterionic species **A** generated from γ-methylidene-δ-valerolactone in the presence of a Pd(0) catalyst (Scheme 77).^118^ Under the optimized reaction conditions, tricyclic heterocycles **130** arising from a (4 + 2) cycloaddition were isolated in very good yields. Different aryl substituents at the C2-position of the pyridinium skeleton as well as on the enamide moiety were tolerated.

DFT calculations led the authors to rationalize the mechanism of this cycloaddition reaction. Thus, the reactive palladium species **A** would be generated by oxidative addition of γ-methylidene-δ-valerolactone with decarboxylation (Scheme 77). Then, the nucleophilic carbon of intermediate **A** would attack the most electrophilic position of the pyridine ring, which was calculated to be the C4-position. The resulting dihydropyridine intermediate **B** would undergo enamine attack to the π-allyl palladium complex, rendering bicyclic structure **C** with concomitant reductive elimination of palladium. Finally, an intramolecular cyclization would give product **130** and regenerate the Pd(0) catalyst.

**Scheme 78:**
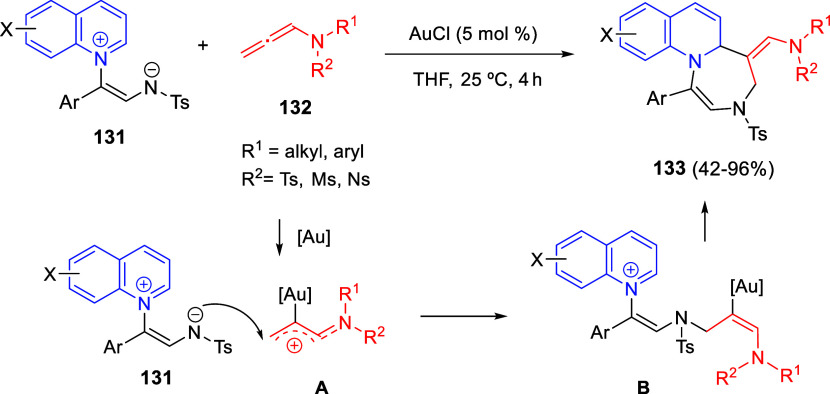
Gold-Catalyzed (5 + 2) Cycloaddition of Quinolinium Zwitterions and Allenamides

Yoo and co-workers also described the use of isolable quinolinium zwitterions **131** as 1,5-dipoles in their (5 + 2) cycloaddition with allenamides **132** under gold(I) catalysis (Scheme 78).^119^ This reaction furnished fused 1,4-diazepine derivatives **133** in good to excellent yields, with various *N*-substituents (R^1^) on the allenamide counterpart. Specifically, *N*-tosyl (R^2^) allenamides gave the best results. Regarding the 1,5-dipole, both electron-donating and electron-withdrawing groups were tolerated on the quinoline backbone (Ar^1^), as well as on the enamide moiety (Ar^2^). It was known that nucleophilic addition of gold(I) salts to allenamides renders Au-bound allylic cation intermediate **A**, which would be attacked by the zwitterionic 1,5-dipole **131** to generate vinyl-gold intermediate **B**. Subsequent intramolecular cyclization would afford the seven-membered ring and regenerate the catalyst (Scheme 78).

**Scheme 79:**
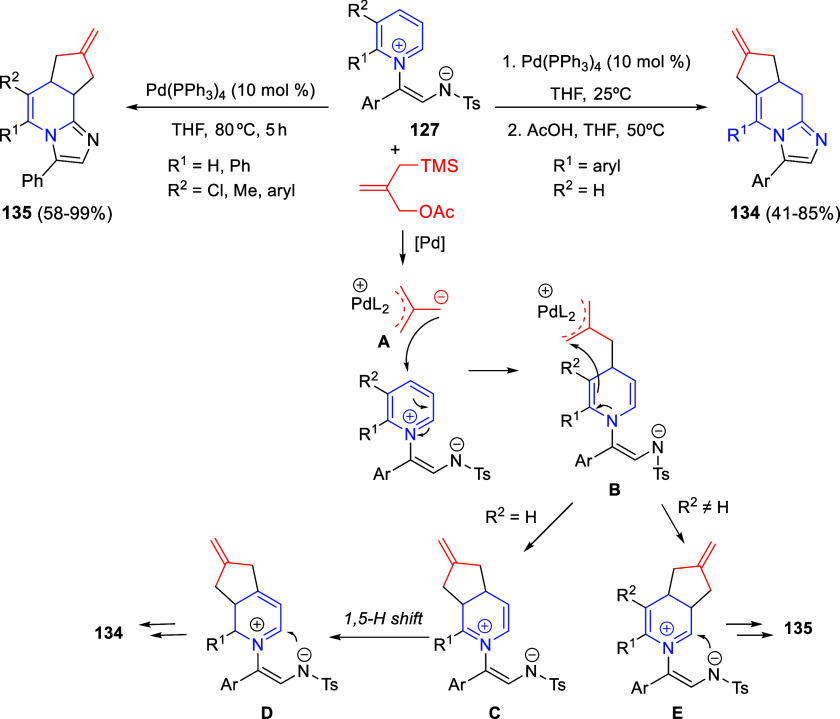
Palladium-Catalyzed Reaction of Pyridinium Zwitterions with Trimethylenemethane via Dearomative (3 + 2) Cycloaddition and Intramolecular Cyclization

In 2019, the groups of Yoo and Baik explored the reactivity of pyridinium zwitterions with trimethylenemethane (TMM) in the presence of a palladium catalyst (Scheme 79).^120^ Thus, when the pyridinium zwitterions **127** were exposed to the Pd-TMM complex generated from 3-acetoxy-2-(trimethylsilylmethyl)-1-propene, a dearomative (3 + 2) cycloaddition reaction took place. However, the resulting cycloadducts were unstable and they were transformed into the corresponding cyclopentane-fused tricyclic imidazole derivatives **134**. Different aryl substituents at the C2-position of the pyridinium ring (R^1^ = aryl, R^2^ = H) were tolerated in the process, leading to final products in good yields. On the other hand, pyridinium zwitterions **127** bearing substituents at the C3-position (R^2^ ≠ H) reacted through the less hindered C5-position to render imidazole derivatives **135** with complete regioselectivity.

DFT calculations were performed in order to understand the origin of the observed regioselectivity. Nucleophilic palladium-TMM species **A** would attack the more electrophilic C4-position of the pyridinium zwitterion to generate dihydropyridine intermediate **B** (R^2^ = H), which would undergo ring closure and 1,5-hydrogen shift, rendering intermediate **D** (Scheme 79). Final C–N bond formation and aromatization upon heating with acetic acid, would yield tricyclic products **134**. Alternatively, when the pyridine ring was substituted at C3 (R^2^ ≠ H), the enamine attack to the π-allyl palladium complex would occur at the C5-position, finally giving regioisomers **135**.

**Scheme 80:**
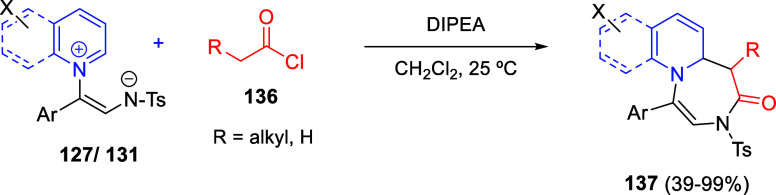
(5 + 2) Cycloaddition Reaction of *N*-Aromatic Zwitterions with *In Situ* Generated Ketenes

Again, Yoo and co-workers reported the use of *N*-aromatic zwitterions in a (5 + 2) cycloaddition reaction with ketenes for the preparation of diazepine derivatives (Scheme 80).^121^ Thus, pyridinium and quinolinium dipoles **127** and **131**, respectively, reacted with *in situ* generated ketenes from acyl chlorides **136** to render 1,5-diazepine derivatives **137** in good yields. The substitution on the pyridine backbone did not affect the process; however, only aliphatic acyl chlorides were tolerated.

**Scheme 81:**
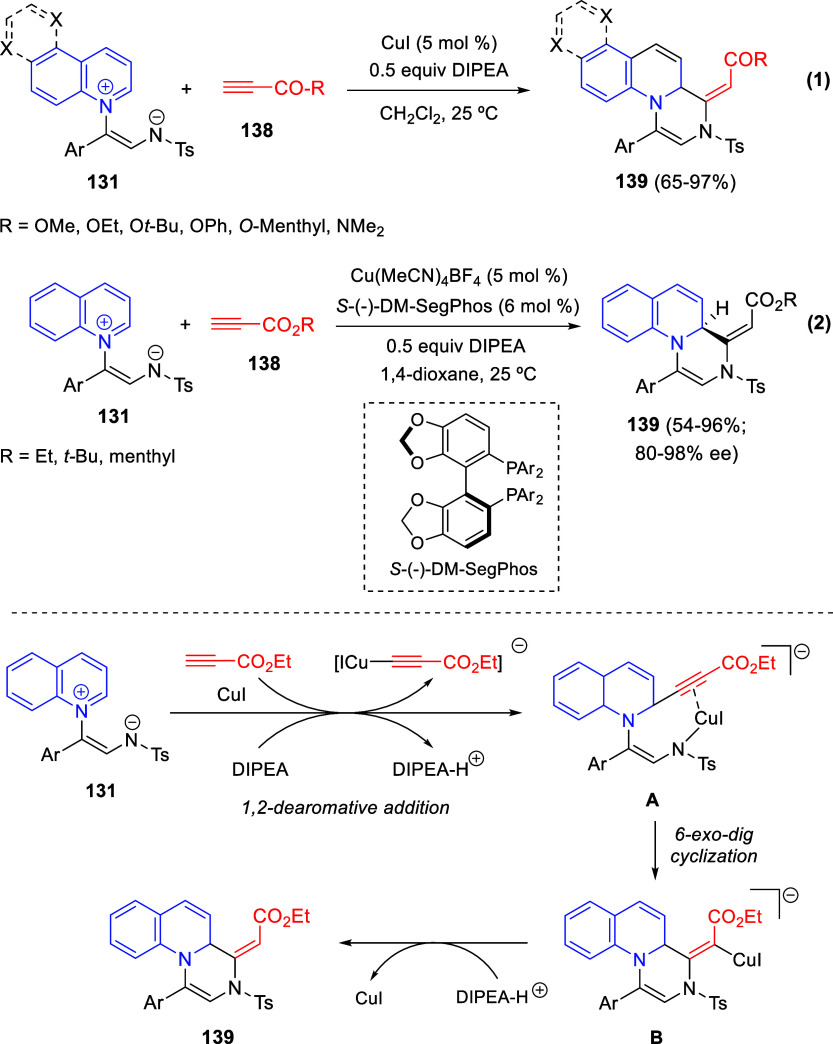
Cu(I)-Catalyzed (5 + 1) Cycloaddition Reaction of Quinolinium Zwitterions and Alkynes

In 2020, Yoo and Baik developed a (5 + 1) copper-catalyzed dearomative cycloaddition reaction of quinolinium zwitterions **131** with electron-deficient terminal alkynes **138** (Scheme 81). Under the optimized reaction conditions, with CuI as the catalyst and diisopropylethylamine (DIPEA) as a base, differently substituted pyrazino[1,2-*a*]quinoline scaffolds **139** were isolated in good yields (Scheme 81, eq 1).^122^ The enantioselective version of this cascade annulation process was achieved by using Cu(MeCN)_4_BF_4_ as the catalyst in the presence of the *S*-(−)-DM-SegPhos chiral ligand, providing the desired optically enriched six-member cyclic systems with excellent enantiocontrol (Scheme 81, eq 2).

Several control experiments and DFT calculations were performed in order to understand the reaction mechanism, particularly in terms of the regioselectivity of the cycloaddition. Once the nucleophilic copper acetylide is formed, through reaction of the propiolate with CuI and deprotonation by DIPEA, it would undergo the dearomative addition to the C2-position of the quinoline ring, leading to intermediate **A**, albeit DFT calculations indicated that the most electrophilic position is the C4-position. This regioselective 1,2-dearomative addition would be favored by the tosylamide functionality, which would act as a directing group because copper would be firmly attached to the amide nitrogen. The pyrazine core would be subsequently generated by means of a 6-*exo*-cyclization through intermediate **B** (Scheme 81).

**Scheme 82:**
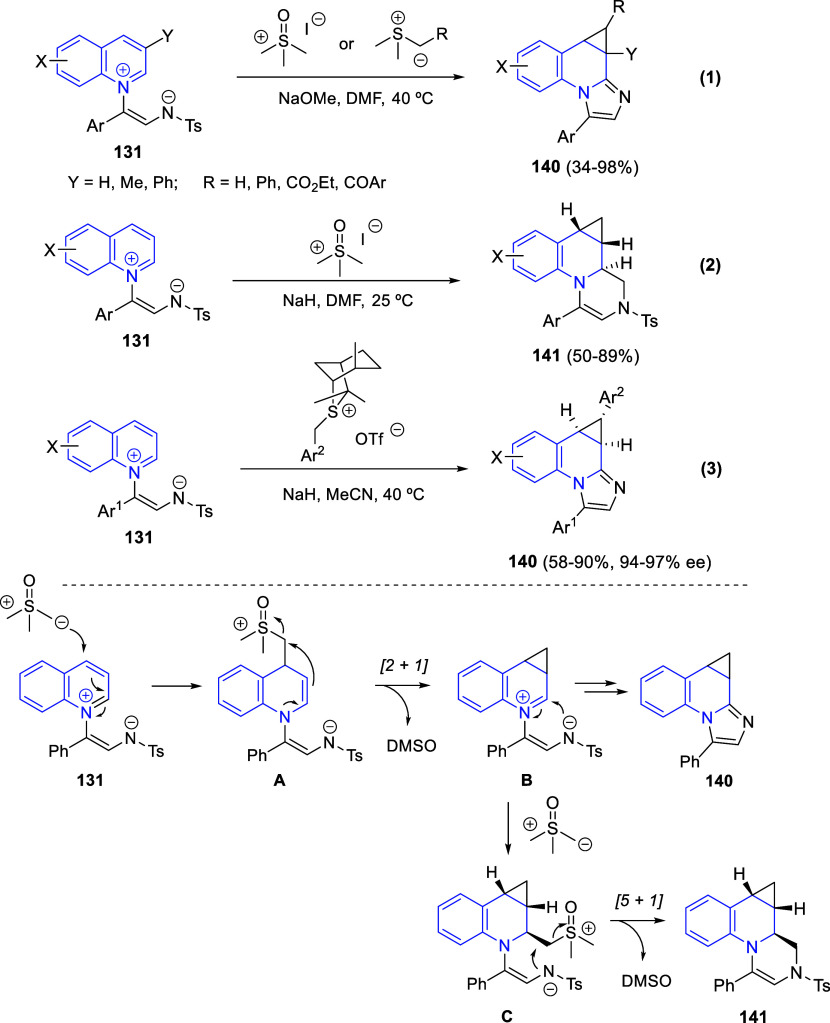
Cyclopropanation of Quinolinium Zwitterions via Site- and Stereoselective Dearomative Cycloadditions

Sulfur ylides have been also employed as reagents for formal cycloaddition reactions of *N*-aromatic zwitterions through a site-selective 1,4-dearomative addition. Specifically, Yoo and co-workers developed a divergent cyclopropanation reaction of quinolinium zwitterions **131** with *in situ* generated sulfur ylides from trialkylsulfoxonium iodides (Scheme 82).^123^ When sodium methoxide was the base employed, in DMF as the solvent, cyclopropane-fused imidazole derivatives **140** were isolated in good yields (Scheme 82, eq 1). Alternatively, the use of a stronger base such as NaH, provided access to cyclopropane-fused tetrahidropyrazinoquinolines **141** as single diastereoisomers (Scheme 82, eq 2). In both cases, the reaction yields were not significantly influenced by the electronic variation on the quinolinium backbone. The synthesis of enantiomerically enriched cyclopropane-fused heterocycles was also attempted by using sulfur ylides as chiral nucleophiles in the 1,4-dearomative reaction. Thus, the reaction of quinolinium zwitterions **131** with a chiral sulfonium salt in the presence of NaH as the base and MeCN as the solvent, afforded cycloadducts **140** with excellent enantiomeric excesses, albeit poor diastereoselectivities. In this case, even with the use of NaH, the formation of the corresponding cycloadducts **141** was not observed, presumably due to the steric bulkiness of the chiral ylide (Scheme 82, eq 3).

Regarding the mechanism for this transformation, it was proposed that the reaction would proceed through the (2 + 1) cycloaddition of the quinolinium zwitterion **131** and the *in situ* generated sulfur ylide, followed by cyclization of intermediate **B** and aromatization to the imidazole moiety of products **140**, when the relatively weak base NaOMe was employed (Scheme 82). The use of a stronger base, NaH, would favor an intermolecular nucleophilic addition of a second sulfur ylide to intermediate **B**, rendering final products **141** through a formal (5 + 1) cycloaddition process.

**Scheme 83:**
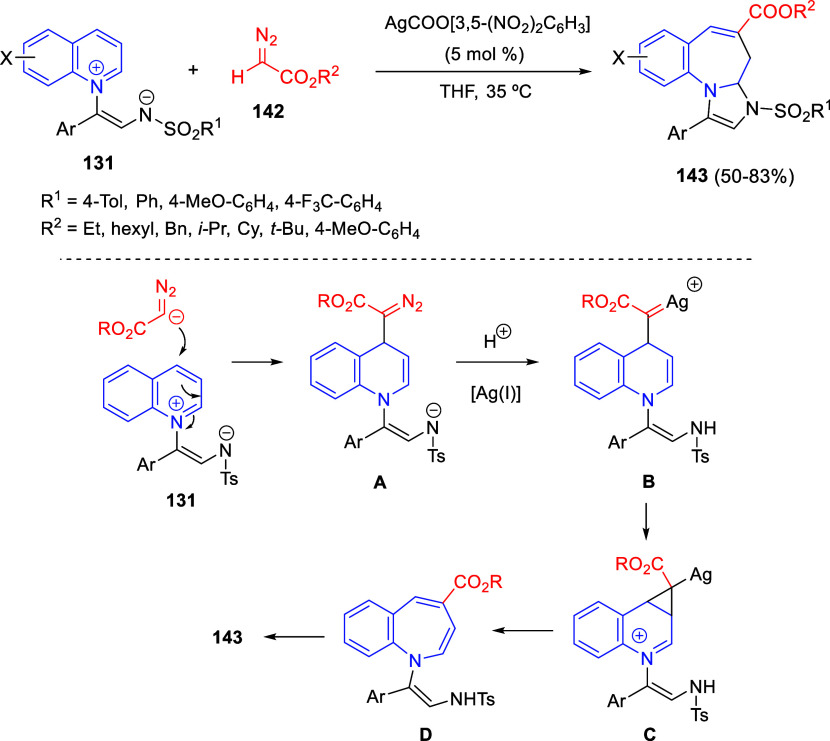
Silver-Catalyzed Ring Expansion Reaction of Quinolinium Zwitterions through 1,4-Dearomative Addition of Diazoacetates

Quinolinium zwitterions **131** also reacted with diazoacetates **142** in a regioselective silver-catalyzed dearomative ring expansion process, which provided access to tricyclic azepine derivatives **143** (Scheme 83).^124^ Although silver catalysts had been scarcely employed to generate carbenoids of diazoacetates, the presence of a benzoate derivative bearing an electron-withdrawing substituent as a counteranion was found to be crucial to obtain the (5 + 1) cycloadducts in good yields. The reaction was compatible with various substituents on the quinolinium, enamine, and sulfonyl moieties of the zwitterions, as well as on the diazoacetate counterpart.

Mechanistic studies revealed that, after generation of the anion from diazoacetate, this would regioselectively attack the C4-position of the quinolinium zwitterion to form intermediate **A** (Scheme 83). Then, reaction with the silver catalyst would occur with N_2_ release to give silver carbenoid **B**, which would undergo intramolecular cyclopropanation to iminium intermediate **C**. Subsequent ring expansion with neutralization of the iminium salt and silver release would provide bicyclic azepine intermediate **D** and, finally, an intramolecular hydroamination process would afford the final tricyclic products **143**.

**Scheme 84:**
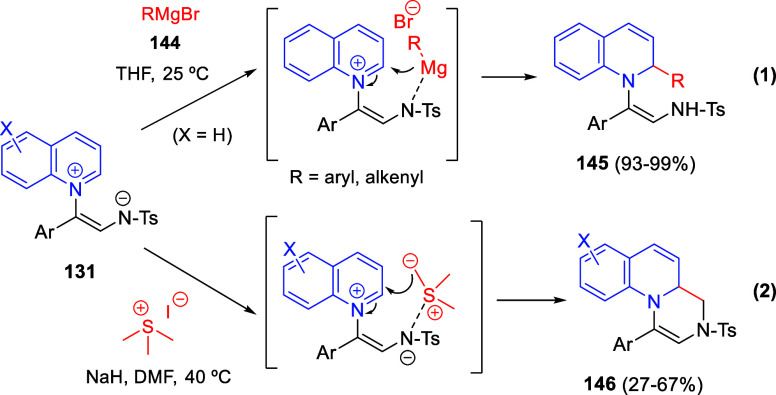
Coordination-Driven Regioselective 1,2-Dearomatizations of Quinolinium Zwitterions

Continuing with the work of the Yoo group on the development of regioselective dearomative reactions of *N*-aromatic zwitterions, their reactivity with organometallic species was studied, showing that coordination between the metal and the nitrogen anion affected the site selectivity of the dearomatization process (Scheme 84).^125^ After testing several organometallic reagents (Mg, Zn, Cu), the authors found that all of them underwent regioselective 1,2-addition to quinolinium zwitterions. Specifically, various Grignard reagents **144** were employed for the dearomatization of quinolinium zwitterions **131**, affording the corresponding dihydroquinolines **145** in excellent yields (Scheme 84, eq 1). Coordination between the metal and the nitrogen anion controlled the site selectivity of this dearomatization process.

On the other hand, while sulfoxonium ylides added to quinolinium zwitterions in a 1,4-dearomative fashion to render cyclopropane-fused derivatives (see Scheme 82),^123^ the addition of trimethylsulfonium iodide, which could be coordinated with the nitrogen anion of the dipole, provided the (5 + 1) cycloadducts **146** via 1,2-dearomative addition in the presence of a strong base (Scheme 84, eq 2).

**Scheme 85:**
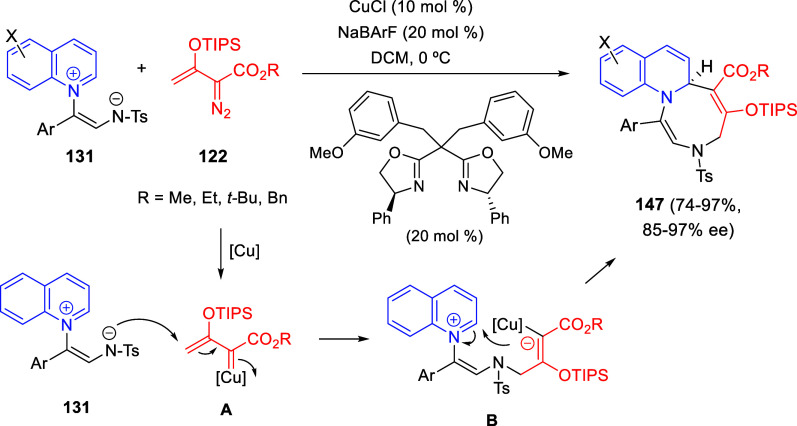
Enantioselective Copper-Catalyzed (5 + 3) Cycloaddition between Quinolinium Zwitterions and Enol Diazoacetates

Very recently, the same authors found that copper(I) chloride catalyzed an enantioselective (5 + 3) cycloaddition of quinolinium zwitterions **131** and enol diazoacetates **122** in the presence of a sterically encumbered chiral BOX-type ligand and NaBArF as a crucial additive (Scheme 85).^126^ In this manner, enantioenriched diazocine derivatives **147** were obtained in very good yields and enantiomeric excesses. Substituents at the C4–C7-positions of the quinolinium ring, irrespective to their electronic properties, were tolerated in the cycloaddition process, as well as several aryl substituents on the enamide moiety. Variations in the ester group of diazo compounds were also accepted, while neither the enol diazoamide derivative nor the pyridinium zwitterion were appropriate substrates for this reaction.

Regarding a plausible mechanism for this transformation, it would be initiated by reaction of the copper salt with the enol diazoacetate to give the copper-carbenoid intermediate **A**, which would undergo conjugate addition of the quinolinium zwitterion, generating tethered intermediate **B**. This would evolve through the stereoselective dearomative cyclization to render the final products **147** (Scheme 85).

**Scheme 86:**
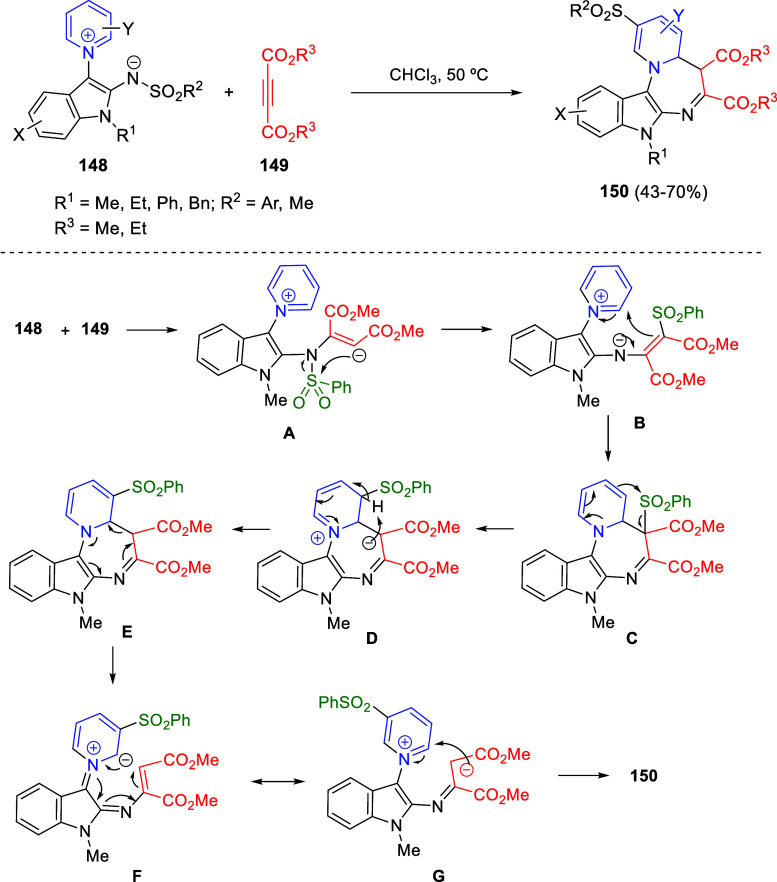
Sequential 1,3-*N*- to *C*- and 1,3-*C*- to *C*-Migrations of Sulfonyl Groups in Aza-(5 + 2) Cycloaddition of Indoloazomethine Ylides

In 2020, Lee and co-workers evaluated the use of a different type of pyridinium zwitterions bearing an indole moiety. These indoloazomethine ylides **148** were stable and could be isolated and subjected to higher-order cycloaddition reactions with dipolarophiles such as dialkylacetylenedicarboxylates **149** (Scheme 86).^127^ However, they do not behave as usual azomethine ylides and 1,4-diazepine derivatives **150** were formed through a sequential aza-(5 + 2) cycloaddition followed by 1,3-*N*- to *C*- and 1,3-*C*- to *C*-migration of the sulfonyl group. Electronic variation of substituents on the phenyl ring of the sulfonyl moiety, as well as on the indole and pyridinium rings did not affect the process. In addition, only one diasteroisomer of the sulfonyl-migrated 1,4-diazepines was observed in all cases.

On the basis of several mechanistic experiments, the authors proposed that the addition of the starting dipole **148** to dimethylacetylenedicarboxylate would result in the formation of intermediate **A**, which would undergo an intramolecular nucleophilic substitution to render azomethyne ylide **B** (Scheme 86). Then, intramolecular enamine attack to the pyridinium moiety would provide pyridodihydro-1,4-diazepinoindole **C**. This intermediate would evolve through intramolecular dienamine attack on the sulfonyl group followed by proton transfer to intermediate **E**. Finally, opening of the 1,4-diazepine followed by rotation to avoid steric interactions, and subsequent cyclization, would render the final pyridodihydro-1,4-diazepinoindoles **150** (Scheme 86).

**Scheme 87:**
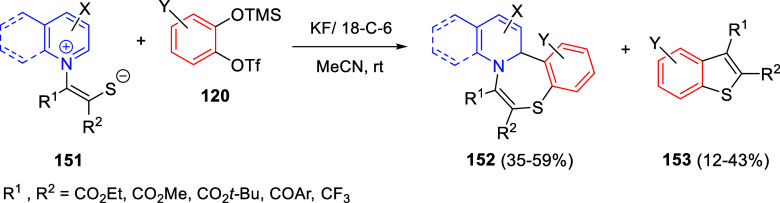
Cyclization Reaction of Pyridinium 1,4-Zwitterionic Thiolates and Arynes

In 2011, Bazgir reported the synthesis of air-stable pyridinium 1,4-zwitterionic thiolates from dialkyl acetylenedicarboxylates, elemental sulfur, and pyridine.^128^ However, the use of those dipoles as versatile synthons was recognized in 2019 by Zhai and co-workers for the synthesis of sulfur-containing heterocycles through two reaction modes, with pyridine as a reactive moiety and with pyridine as a leaving group.^129^ The first example of the participation of pyridinium 1,4-zwitterionic thiolates **151** in cycloaddition reactions was reported by Zhai and Cheng in 2020 (Scheme 87).^130^ Thus, their reaction with aryne precursors **130** in the presence of KF/18-C-6 as a fluoride reagent and additive, gave benzopyridothiazepines **152**, through a 1,5-dipolar cycloaddition reaction wherein pyridine was a reactive moiety. In this reaction, benzothiophenes **153** were also observed as side products, generated by means of a (3 + 2) cascade cyclization reaction, including *S*-nucleophilic addition, *C*-Michael addition, and release of pyridine as a leaving group in a retro-Michael event. A variety of substituents both on the aryne precursor and the zwitterionic thiolates were compatible with the process, although these required the presence of two electron-withdrawing groups, either aromatic ketones, esters or CF_3_ moieties.

**Scheme 88:**
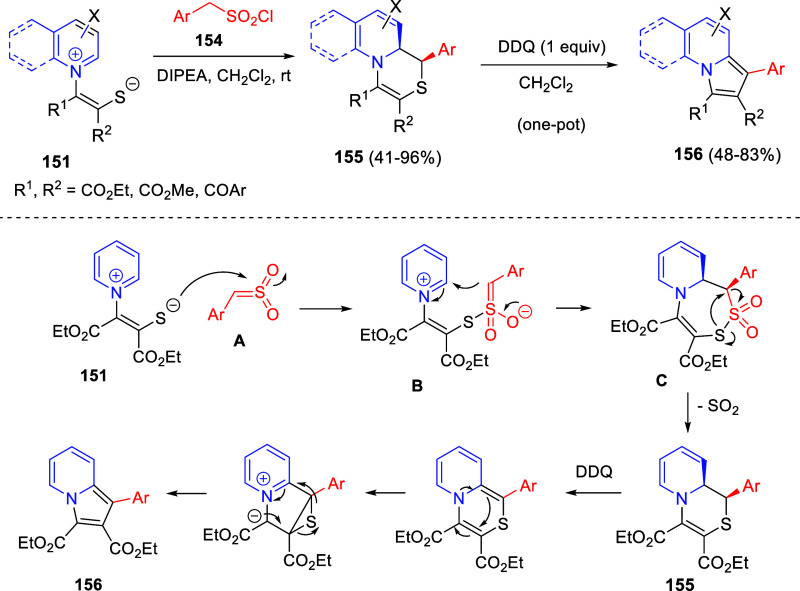
Formal (5 + 1) Cycloaddition Reaction of Pyridinium 1,4-Zwitterionic Thiolates with Sulfenes

The same authors described the reaction of pyridinium zwitterionic thiolates **151** with sulfenes, easily generated *in situ* from arylmethanesulfonyl chlorides **154** in the presence of a base (Scheme 88).^131^ This reaction gave rise to a family of 1,9a-dihydropyrido[2,1-*c*][1,4]thiazines **155** in a formal (5 + 1) cycloaddition procedure, which was compatible with different electron-withdrawing groups on the thiolate counterpart. In addition, the authors found that the final products could be oxidized with DDQ and, after sulfur extrusion, indolizines **156** were obtained in a one-pot manner. When alkylmethanesulfonyl chlorides were employed, 3*H*-1,2-dithiole 2,2-dioxides were formed instead, arising from a formal (3 + 2) pathway, with pyridine loss.

Mechanistically, once sulfene **A** was generated from arylmethanesulfonyl chloride **154** in the presence of a base, it would undergo nucleophilic attack from the zwitterionic thiolate **151** to form thiosulfonate intermediate **B** (Scheme 88). Then, the α-carbon of the sulfonyl group would attack the 2-position of the pyridine ring, leading to intermediate **C** with excellent diastereoselectivity. Finally, the spontaneous extrusion of SO_2_ would deliver products **155**. The subsequent addition of DDQ would promote the oxidation/ring-contraction upon sulfur extrusion, to render indolizines **156**.

**Scheme 89:**
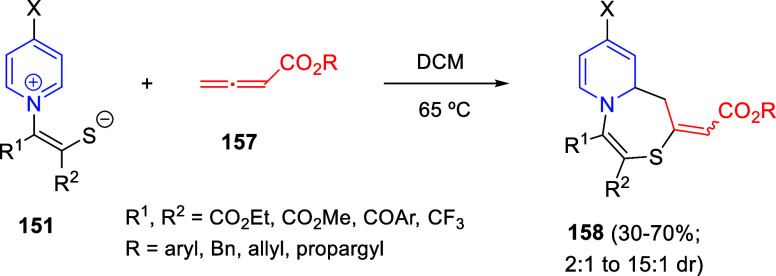
1,5-Dipolar Cycloaddition Reaction between Pyridinium 1,4-Zwitterionic Thiolates and Activated Allenes

Cheng, Wang, Zhai, and co-workers also evaluated the reactivity of pyridinium 1,4-zwitterionic thiolates in cycloaddition reactions (Scheme 89). Thus, thermal heating of dipoles **151** with allenoates **157** in dichloromethane gave rise to pyridothiazapines **158** through a (5 + 2) cycloaddition process that would involve a *S*-nucleophilic addition of the thiolate to the allenoate and subsequent *C*-nucleophilic addition to the pyridinium moiety.^132^ Final products were obtained as variable diastereoisomeric mixtures at the double bond. Different electron-withdrawing groups (R^1^/R^2^), as well as several pyridinium substituents had little effect on the overall yield. However, with electron-donating substituents at the 4-position of the pyridine ring, a formal (3 + 2) pathway occurred, leading to tetrasubstituted thiophenes via the extrusion of 3-thioxoacrylates.

**Scheme 90:**
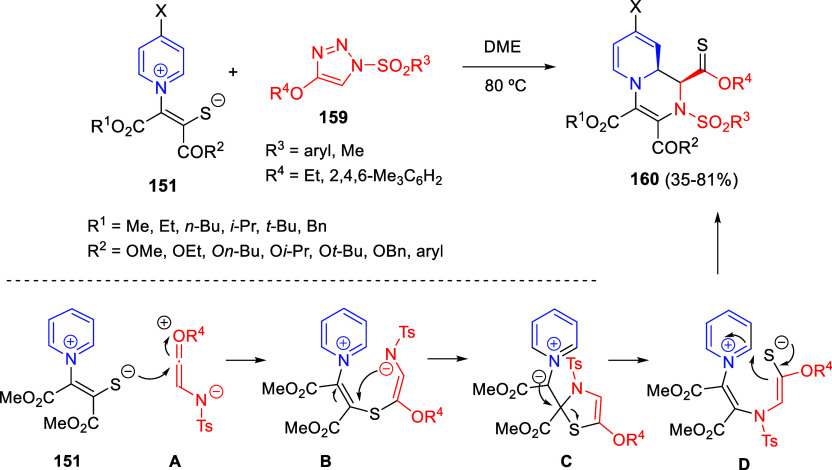
Formal (4 + 2) Cycloaddition Reaction of Pyridinium 1,4-Zwitterionic Thiolates with 1-Sulfonyl-1,2,3-triazoles

Another example describing the reactivity of pyridinium 1,4-zwitterionic thiolates was reported by Li and co-workers in 2021 (Scheme 90).^133^ The reaction involved the use of 1-sulfonyl-1,2,3-triazoles **159**, which reacted with the thiolate dipoles **151** acting as five-membered synthons, in a formal (4 + 2) cycloaddition reaction to render pyrido[1,2-*a*]pyrazine derivatives **160** in moderate to good yields. A series of sulfonyl groups in the triazole counterpart were successfully applied in this reaction. Different substituents at the ester or aryl ketone moieties of the thiolates were also compatible in this transformation.

The proposed mechanism of the reaction would start with the *S*-thiolate attack to intermediate **A**, *in situ* generated by thermal heating of triazole **159** (Scheme 90). The resulting intermediate **B** would undergo Michael-type addition and elimination to form intermediate **D**. This thioester enolate would intramolecularly attack the 2-position of the pyridine ring, delivering final pyrazine derivatives **160**.

**Scheme 91:**
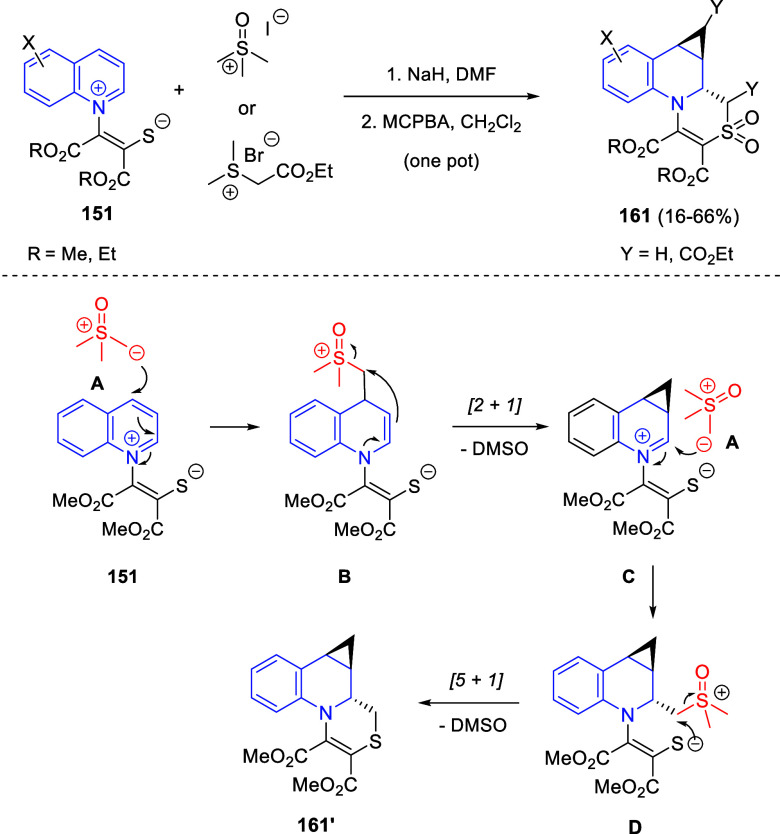
Cyclopropanation of Quinolinium Zwitterionic Thiolates via Dearomative Reactions with Sulfur Ylides

The groups of Jin and Zhang reported the cyclopropanation reaction of quinolinium zwitterionic thiolates via dearomative addition with sulfur ylides as C1 synthons (Scheme 91).^134^ Specifically, the reaction of thiolates **151** and trimethylsulfoxonium iodide or (ethoxycarbonylmethyl)dimethylsulfonium bromide in DMF with NaH as a base afforded cyclopropa[*c*][1,4]thiazino-[4,3-*a*]quinolones that were not stable and they were further oxidized with *m*-CPBA in a one-pot manner to the corresponding sulfones **161**. However, only electron-donating substituents on the quinolinium moiety of the zwitterionic thiolates were tolerated in the tandem process.

According to experimental findings and previous studies, sulfur ylide **A**, generated *in situ* by deprotonation of trimethylsulfoxonium iodide, would attack the quinolinium thiolate **151** to form intermediate **B** (Scheme 91). This would be transformed into cyclopropane-fused intermediate **C** via (2 + 1) cycloaddition. Then, an intermolecular nucleophilic attack of a second equivalent of sulfur ylide **A** would generate intermediate **D**, which would cyclize to afford tricyclic products **161′** in a formal (5 + 1) cycloaddition event.

**Scheme 92:**
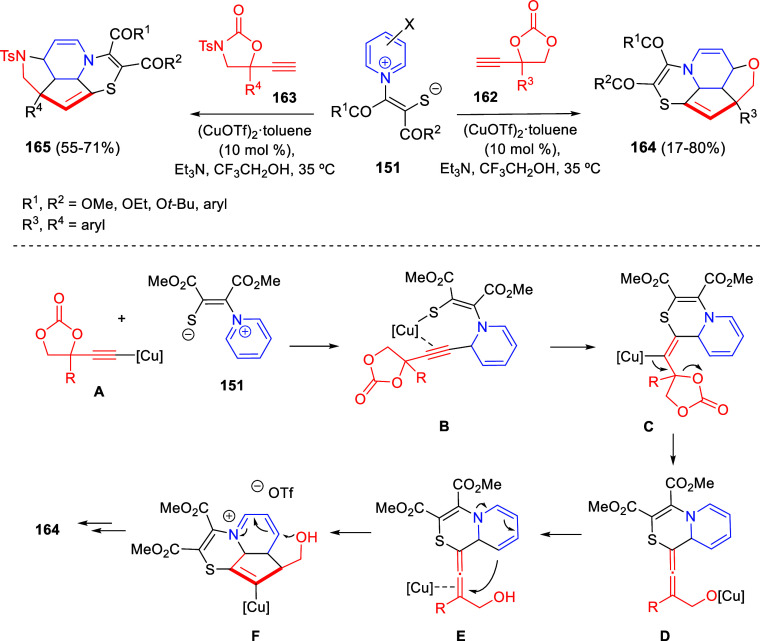
Decarboxylative Cascade Cyclization of Propargylic Cyclic Carbonates/Carbamates and Pyridinium 1,4-Zwitterionic Thiolates

You, Yuan, and co-workers recently developed a copper-catalyzed cascade cyclization of pyridinium 1,4-zwitterionic thiolates **151** with propargylic cyclic carbonates **162** and carbamates **163** (Scheme 92).^135^ This decarboxylative cyclization of alkyne-substituted carbonates took place in 2,2,2-trifluoroethanol with copper(I) triflate toluene complex as the catalyst and triethylamine as a base, rendering fused polyheterocyclic compounds **164** and **165** as single diastereoisomers in moderate to good yields. Interestingly, four new bonds and four new stereocenters were formed in a single step. The catalytic process was compatible with differently substituted aryl carbonates and carbamates; however, it did not proceed with alkyl-substituted cyclic carbonate and carbamate substrates. Regarding the thiolate counterpart, diversely substituted pyridinium zwitterions were tolerated, as well as compounds bearing ester and ketone groups.

A possible catalytic cycle for this decarboxylative cascade cyclization would start with the activation of the alkyne by the copper catalyst and deprotonation to give copper acetylide **A** that would effect the dearomative 1,2-addition over the pyridine ring (Scheme 92). The resulting intermediate **B** would evolve to intermediate **C** through 6-*exo*-cyclization and this would undergo a sequential carbonate ring-opening and decarboxylation to allenolated copper species **D**. After protonation, copper-activated allenol intermediate **E** would intramolecularly cyclize from the C3-position of the 2*H*-pyridine ring to the β-carbon of the allenic moiety, giving tricyclic intermediate **F**. Finally, an intramolecular formal oxa-Michael addition to the C4-position of the 2*H*-pyridine ring followed by protodemetalation would deliver tetracyclic products **164** and regenerate the copper catalyst.

**Scheme 93:**
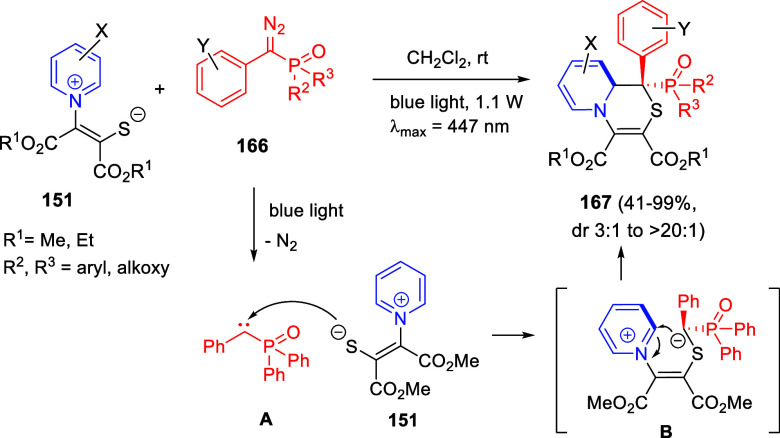
Visible-Light-Induced (1 + 5) Annulation of Phosphoryl Diazomethylarenes and Pyridinium 1,4-Zwitterionic Thiolates

The last example of dearomative cycloaddition reactions employing pyridinium 1,4-zwitterionic thiolates took advantage of the photoredox methodology. In this context, Xu and co-workers described a visible-light-promoted reaction of phosphoryl diazomethylarenes **166** and pyridinium 1,4-zwitterionic thiolates **151** to afford dialkyl 1-phosphoryl-1,9a-dihydropyrido[2,1-*c*][1,4]thiazine-3,4-dicarboxylates **167** in good to excellent yields and diasteroselectivities (Scheme 93).^136^ Differently substituted aryl substituents on the starting diazocompounds were compatible with the process; while pyridinium thiolates with electron-rich *ortho*-/*para*-substituents did not undergo the annulation process. This reaction would involve the nucleophilic attack of thiolates to the electron-deficient carbene intermediates **A**, generated from phosphoryldiazocompounds **166** under blue light irradiation, followed by intramolecular cyclization through nucleophilic addition to the C=N bond of the pyridinium ring (Scheme 93).

### 3.6 Other Types of *N*-Heterocyclic Dipoles

In this section, we will include those dipolar-type cycloaddition reactions involving pyridines, quinolines, and isoquinolines distinct from ylides and imides.

**Scheme 94:**
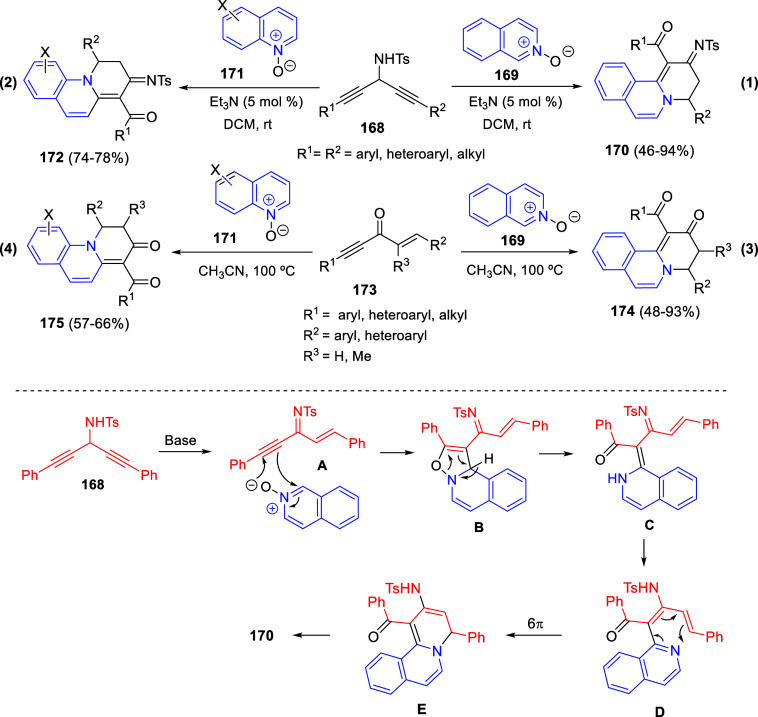
Metal-Free Reactions of 1,4-Diynes and 1-En-4-yn-3-ones with Isoquinoline and Quinoline *N*-Oxides

In 2017, Wang and co-workers described a metal-free cascade reaction of isoquinoline- and quinoline-*N*-oxides with 1,4-diynes and 1-en-4-yn-3-ones (Scheme 94).^137^ First, the authors found that isoquinoline *N*-oxides **169** reacted with 1,4-dyines **168** in the presence of trimethylamine as a base, to render 3,4-dihydro-2*H*-pyrido[2,1-*a*]isoquinolines **170** in good yields (Scheme 94, eq 1). Symmetrical starting diynes bearing aryl, heteroaryl, or alkyl groups were tolerated in the process, as well as unsymmetrical diynes, which provided regioisomeric mixtures of products. The reaction was further extended to differently substituted quinoline *N*-oxides **171** to access the corresponding 3,4-dihydro-1*H*-pyrido[1,2-*a*]quinolines **172** (Scheme 94, eq 2).

Moreover, the authors proved that 1-en-4-yn-3-one derivatives **173** could also react with isoquinoline- and quinoline-*N*-oxides to afford the analogous substituted polycyclic products **174** and **175**, respectively, in moderate to good yields (Scheme 94, eqs 3 and 4). In this case, the participation of a base was not necessary. In addition, different substituents were allowed at the alkyne and alkene terminal positions, without entailing regioselectivity issues.

A plausible mechanism for the formation of 3,4-dihydro-2*H*-pyrido[2,1-*a*]isoquinolines **170** was proposed by the authors. Accordingly, 1,4-diyne **168** would tautomerize to conjugated imine **A** in the presence of the base (Scheme 94). This electron-poor alkyne **A** would react with the isoquinoline *N*-oxide **169** via a (3 + 2) cycloaddition process to generate *N*,*O*-heterocyclic intermediate **B**. Subsequent opening of the isoxazole ring and tautomerization would lead to intermediate **D**, which would undergo 6π-electrocyclization to give intermediate **E**. Finally, tautomerization of the enamine to the conjugated imine would render final products **170**.

**Scheme 95:**
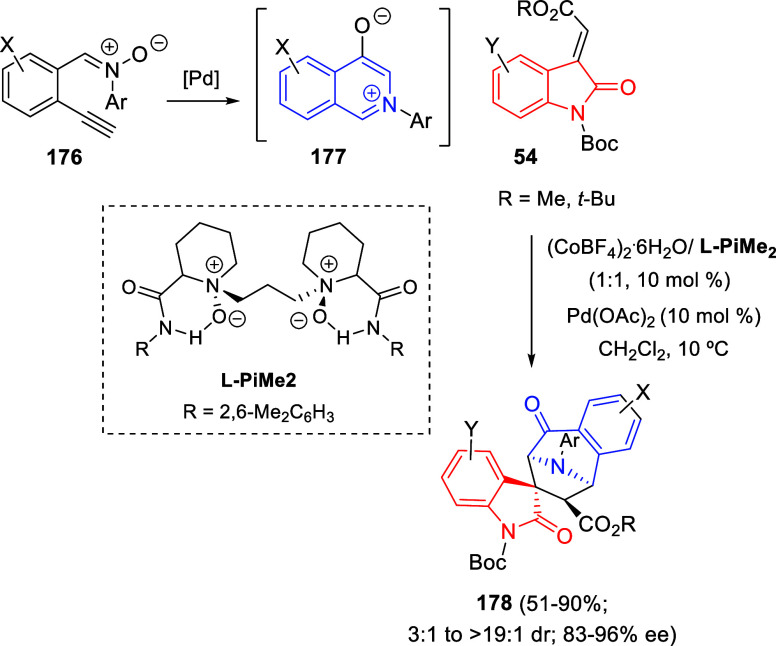
Catalytic Asymmetric Tandem Cycloisomerization/(5 + 2) Cycloaddition Reaction of *N*-Aryl Nitrone Alkynes with Methyleneindolinones

Feng and co-workers disclosed the reaction of *in situ* generated isoquinoline-containing *N*-azomethine ylides with methyleneindolinones (Scheme 95).^138^ The reacting azomethine ylides **177** were formed *in situ* from 2-ethynylphenyl-substituted nitrones **176** through a palladium(II)-catalyzed cycloisomerization involving N–O bond cleavage and further addition of the imine to the α-carbonyl carbenoid. Those azomethine ylides **177** underwent an asymmetric intermolecular (5 + 2) cycloaddition reaction with methyleneindolinones **54** in the presence of a chiral *N*,*N*′-dioxide-Co(II) complex. This dual metallic relay catalysis strategy rendered a series of spiro-tropanyl oxindole derivatives **178** in good yields with high diastereo- and enantioselectivities. In all cases, only trace amounts of the regioisomeric cyclic azomethine ylides were observed. The substitution on the oxindole counterpart was evaluated, showing that the process is compatible with both electron-donating and electron-withdrawing groups at the C5- and C6-positions. The ester moiety had a marked effect on the stereoselectivity and the *tert*-butyl group provided the best results in terms of diastereoselection. Regarding the starting nitrones **186**, the electronic properties of the substituents had little impact on the reaction outcome.

**Scheme 96:**
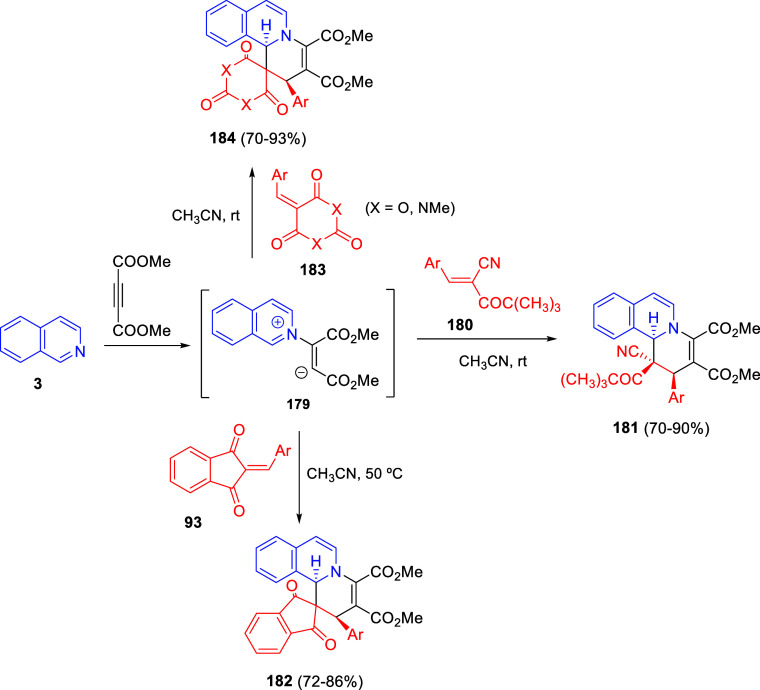
Tandem Reactions of Isoquinoline-Derived Huisgen’s 1,4-Dipoles with Various Alkene Dipolarophiles

Huisgen’s 1,4-dipoles are versatile zwitterionic intermediates generated by addition of nitrogen heterocycles such as pyridine, quinoline, or isoquinoline to electron-deficient alkynes. They can react *in situ* with various electrophiles and dipolarophiles to give a variety of heterocyclic scaffolds. The participation of those dipoles in cycloaddition reactions for the construction of six-membered *N*-heterocyclic compounds was studied by Yan, Han, and co-workers (Scheme 96).^139^ Huisgen’s 1,4-dipoles **179**, *in situ* generated through the addition of isoquinoline to dimethyl acetylenedicarboxylate (DMAD), were subjected to a formal (4 + 2) cycloaddition reaction with arylidene pivaloylacetonitriles **180** to render pyrido[2, 1-*a*]isoquinolines **181** in good yields and with very high diastereoselectivity. The three-component reaction was further extended to other dipolarophiles such as 2-arylidene-1,3-indanediones **93**, arylidene-substituted Meldrum acids (X = O), and *N*,*N*-dimethylbarbituric acids (X = NMe) **183**. These reactions yielded the corresponding functionalized spiropyridoisoquinolines **182** and **184** in good yields (Scheme 96).

One year later, the same authors extended the developed three-component protocol to quinoline-derived Huisgen’s 1,4-dipoles.^140^

**Scheme 97:**
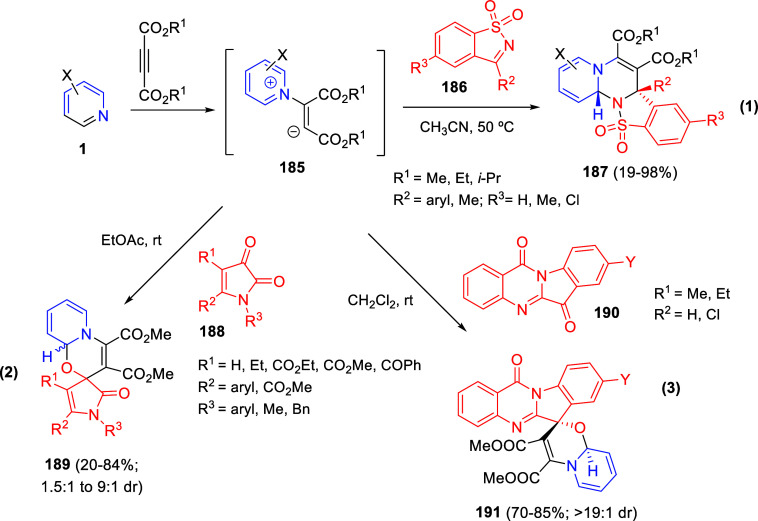
Tandem Cyclizations of Pyridine-Derived Huisgen’s 1,4-Dipoles with Various Dipolarophiles

Pyridine-derived Huisgen 1,4-dipoles have also been employed in several cycloaddition reactions. For example, Zhao and co-workers developed a tandem cyclization process of cyclic *N*-sulfonyl ketimines **186** with *in situ* generated dipoles **185** from dialkyl acetylenedicarboxylates and pyridines (Scheme 97).^141^ This reaction rendered tetracyclic tetrahydropyrimidine-fused benzosultams **187** as single diastereoisomers in low to excellent yields. Pyridines with electron-donating groups provided higher chemical yields than that bearing electron-withdrawing groups (Scheme 97, eq 1).

The same pyridine-derived Huisgen 1,4-dipoles **185** reacted with 1*H*-pyrrole-2,3-diones **188** as dipolarophiles in a regioselective (4 + 2) cycloaddition process (Scheme 97, eq 2).^142^ A variety of 1*H*-pyrrole-2,3-diones bearing electronically diverse substituents at the 4-, 5-, and *N*^1^-positions were employed, without significantly affecting the yields of the spirocyclic cycloadducts **189** albeit they were obtained as mixtures of diastereoisomers. The authors also extended this methodology to *N*-alkyl isatins, 1*H*-indeno[1,2-*b*]quinoxalin-11-one and isatylidene malononitrile as dipolarophiles with similar results.^142^

A third example regarding cycloaddition reactions of pyridine-derived Huisgen 1,4-dipoles **185** entailed their reaction with tryptanthrins **190** as dipolarophiles, to render spirooxazine derivatives **191** in good yields with excellent diastereoselectivity (Scheme 97, eq 3).^143^ Huisgen’s dipoles derived from isoquinoline also performed successfully in this three-component reaction.

**Scheme 98:**
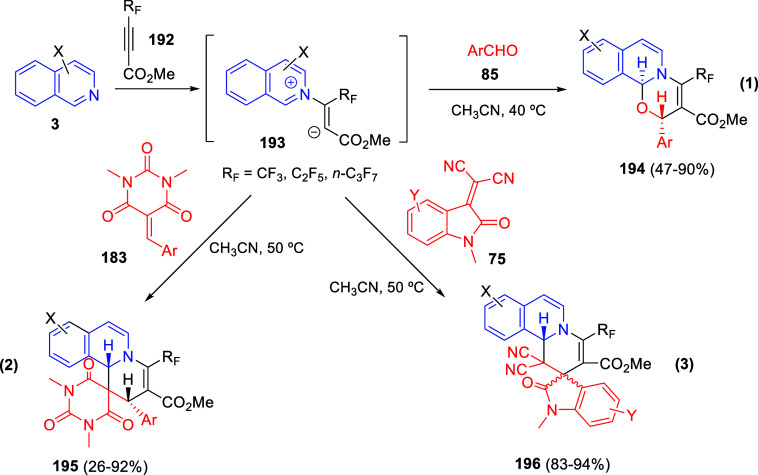
Reactivity of Isoquinoline-Derived Huisgen 1,4-dipoles *In Situ*-Prepared from Methyl Perfluoroalk-2-ynoates

Huisgen 1,4-dipoles can also be generated by addition of nitrogen heterocycles to other activated acetylenes instead of acetylene dicarboxylates. In this context, Zhang, Cao and co-workers explored the reactivity of isoquinoline-derived Huisgen 1,4-dipoles *in situ*-prepared from methyl perfluoroalk-2-ynoates (Scheme 98). When quinolones **3**, fluorinated propiolates **192**, and aromatic aldehydes **85** were heated in acetonitrile, *trans*-perfluoroalkylated [1,3]oxazino[2,3-*a*]isoquinoline derivatives **194** were obtained as single diastereoisomers in moderate to good yields (Scheme 98, eq 1).^144^ The reaction was limited to aromatic aldehydes bearing electron-withdrawing groups at the 4-position of the aromatic ring.

The same 1,4-dipoles **193**, derived from isoquinolines and methyl perfluoroalk-2-ynoates, also reacted with arylidene-substituted *N*,*N*-dimethylbarbituric acids **183** as dipolarophiles, to render *cis*-spiropyrido[2,1-*a*]isoquinoline-1,5′-pyrimidines **195** in moderate to good yields, after proton-promoted transformation of the *trans*-isomers (Scheme 98, eq 2).^145^ A variety of substituents on the aromatic ring of 1,3-dimethylbarbituric acids as well as on the starting isoquinolines were tolerated in this reaction.

In 2021, the same groups extended the three-component cyclization to isatin derivatives **75** as dipolarophiles, for the synthesis of fluorinated spirooxindole-fused benzo[a] quinolizidines **196** in very good yields, albeit as equimolecular mixtures of diastereoisomers (Scheme 98, eq 3).^146^

**Scheme 99:**
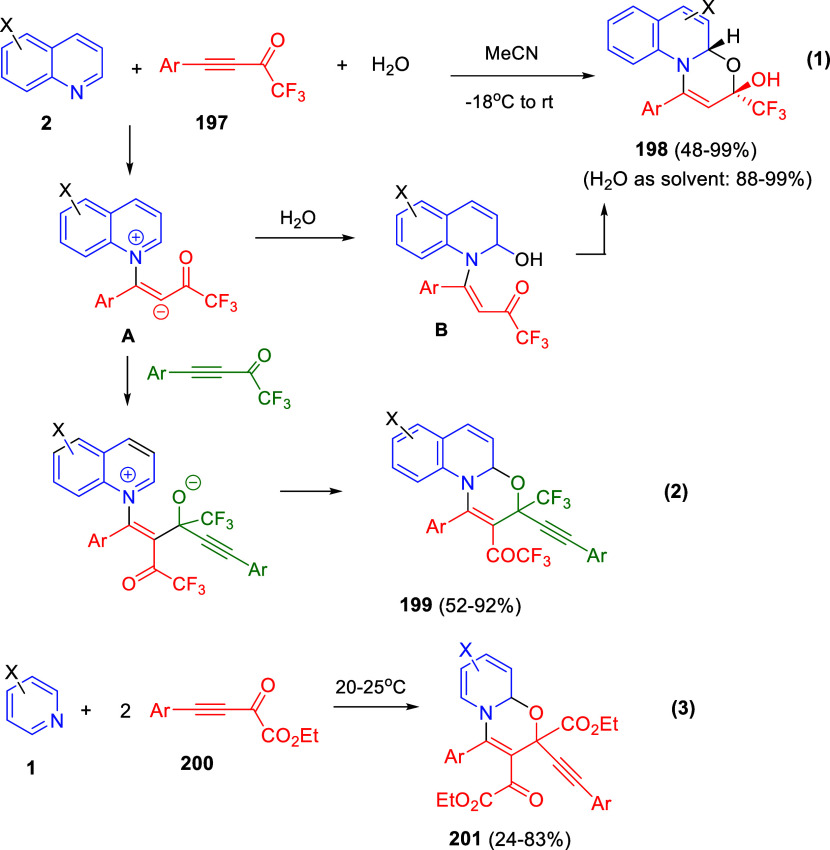
Catalyst-Free Annulation Reactions of Quinolines and Pyridines with Electron-Deficient Acetylenes

Nenajdenko and Trofimov explored the use of trifluoroacetylacetylenes as precursors of 1,3-dipoles. In 2018, they reported a reaction between quinolines, aryltrifluoroacetylacetylenes **197**, and water in acetonitrile as the solvent, which rendered trifluoromethylated oxazinoquinolines **198** as single diastereoisomers in very good yields (Scheme 99, eq 1).^147^ This three-component annulation strategy was general for differently substituted trifluoroacetylacetylenes and quinolines, although acetylenes bearing electron-donating groups required longer time to be complete and substituents at the 2- and 8-positions of the quinoline ring were not tolerated. The proposed mechanism for this annulation reaction would involve first the formation of the 1,3-dipole intermediate **A**, followed by conjugate addition of a molecule of water to the 2-position of the quinoline ring and further hemiacetalization with the carbonyl group (Scheme 99, eq 1). In addition, the authors found that, when the reaction was performed in water as the solvent, it proceeded up to 20 times faster to provide the oxazinoquinoline products in almost quantitative yields.^148^

The reaction of quinolines with two molecules of aryltrifluoroacetylacetylenes **197** in the absence of water and solvent afforded 3-arylethynyl-3-trifluoromethyl-1,3-oxazinoquinolines **199** (Scheme 99, eq 2), through formation of the zwitterionic intermediate **A**, followed by addition of the second molecule of trifluoromethyl ketone and intramolecular cyclization.^149^ In this case, the final products were obtained as mixtures of diasteroisomers. The same authors extended this protocol to pyridines as the heterocyclic counterpart.^150^

More recently, the same group reported a similar cascade procedure employing, in this case, oxalylarylacetylenes **200** as precursors of the corresponding 1,4-dipoles upon reaction with pyridine (Scheme 99, eq 3).^151^ The reaction took place at room temperature with two molecules of acetylene, to render densely functionalized tetrahydropyrido[2,1-*b*][1,3]oxazines **201** as mixtures of diasteroisomers in moderate to good yields. The process was further extended to quinolines with comparable results.^152^

**Scheme 100:**
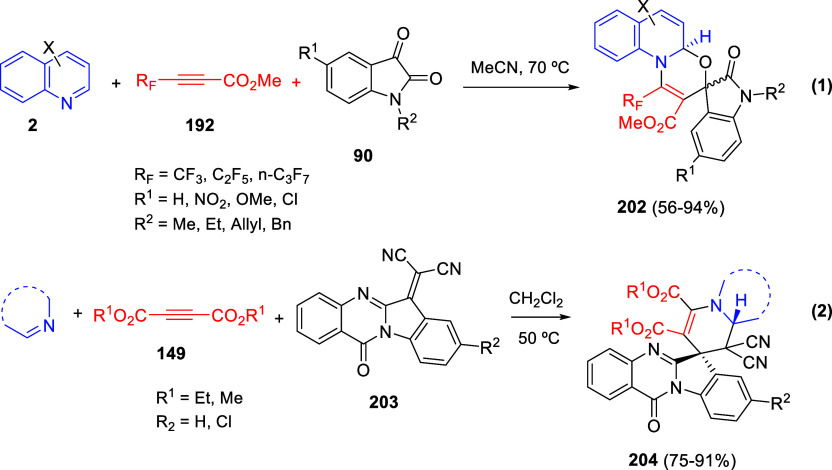
Three-Component Reactions Involving Huisgen’s Zwitterionic 1,4-Dipole Intermediates

Cao, Zhang, and co-workers evaluated the three-component domino reaction of quinolines **2**, methyl perfluoroalkyl-2-ynoates **192** and isatin derivatives **90**, for the synthesis of perfluoroalkyl-substituted spiro-1,3-oxazines **202**, which were obtained as almost equimolecular mixtures of diasteroisomers (Scheme 100, eq 1).^153^ This reaction proceeded via an intermolecular Michael addition of the quinoline ring to the more electrophilic carbon center of the triple bond to generate a Huisgen’s 1,4-dipole intermediate, which evolved through nucleophilic addition to the carbonyl group of isatin and cyclization. The electronic nature and position of substituents on the quinoline ring had negligible effects on the reaction, as well as electron-withdrawing and electron-donating groups at the 5-position of the isatin moiety. Regarding the fluorinated group, bulkier substituents afforded lower yields of the corresponding products.

A similar strategy involved Huisgen zwitterionic intermediates generated by addition of aromatic *N*-heterocycles (pyridines, quinolones, and isoquinolines) to acetylenic esters **149** and subsequent reaction with tryptanthrin-malononitrile adducts **203** (Scheme 100, eq 2).^154^ The three-component reaction took place in dichloromethane to render functionalized spiroindolo[2,1-*b*]quinazolines **204** as single diastereoisomers in good yields.

**Scheme 101:**
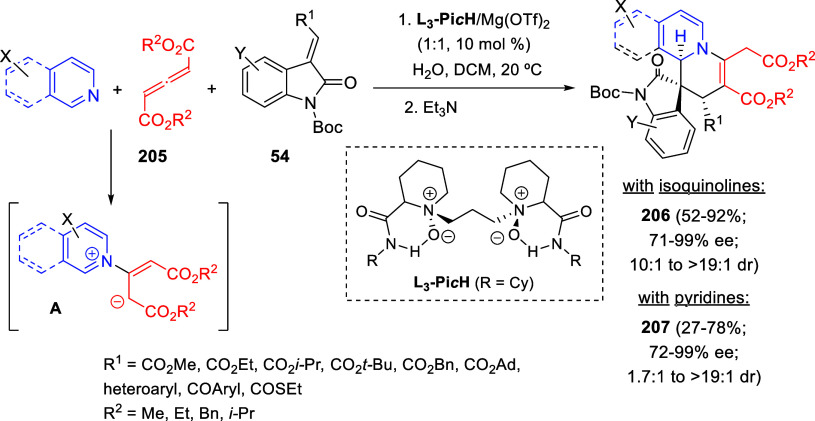
Enantioselective Dearomative Three-Component Reaction of Isoquinolines and Pyridines, Allenoates, and Methyleneindolinones

Last year, Cao and Feng developed an enantioselective three-component reaction involving a dearomative (4 + 2) dipolar cycloaddition of isoquinolines and pyridines (Scheme 101).^155^ The nucleophilic addition of these *N*-heterocycles to allenoates **205** generated transient 1,4-dipoles **A**, which in turn cyclized with methyleneindolinones **54** in the presence of a chiral *N*,*N*′-dioxide/magnesium complex. Despite the challenge that those dearomative cyclizations entailed, mainly due to the high reactivity of Huisgen’s 1,4-dipoles which promotes strong background reactions, the authors found that chiral ligand *N*,*N*′-dioxide **L3-Pi***c***H** in combination with Mg(OTf)_2_ provided the best results in the synthesis of 1,2-dihydroisoquinoline and 1,2-dihydropyridine derivatives **206** and **207**. A wide variety of ester groups as well as thioester, heteroaryl and benzoyl groups at the 3-position of the methyleneindolinone were tolerated in the process, delivering the corresponding dihydroisoquinoline products in good yields with excellent diastereo- and enantioselectivities. Regarding the substitution on the phenyl ring of the oxindole, electron-donating substituents yielded better results than electron-withdrawing ones. Isoquinoline rings bearing electronically different substituents at the C4-, C5-, C6-, or C8-positions provided good yields and moderate to excellent diastereoselectivities and enantioselectivities. Finally, when the tandem reaction was applied to pyridines, it was found to be less efficient, in general, than that of isoquinoline derivatives.

### 3.7 Pyridines, Quinolines, and Isoquinolines as Dienes, Dienophiles, and Dipolarophiles in Cycloaddition Reactions

Besides acting as precursors of *N*-ylides, *N*-imides, *N*-heterocyclic zwitterions and Huisgen 1,4-dipoles, pyridine, quinoline and isoquinoline rings, both in neutral manner or as salts, can also be themselves good electron-deficient dienes in several inverse electron demand Diels–Alder reactions, and perform as good dienophiles and dipolarophiles with appropriate dipoles.

**Scheme 102:**
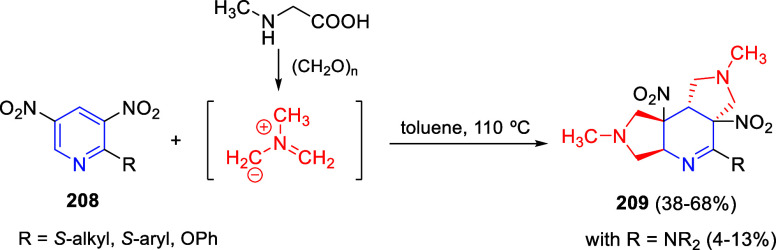
1,3-Dipolar Cycloaddition of 2-Substituted 3,5-Dinitropyridines and Unstabilized *N*-Methyl Azomethine Ylide

Regarding the dearomatization of *N*-heterocycles by means of dipolar cycloadditions, it had been reported that dinitropyridines and nitro (iso)quinolines were capable of acting as 2π-electron components in (3 + 2) cycloaddition processes with nonstabilized azomethine ylides to afford highly functionalized polycyclic structures.^156^ In this context, Bastrakov and co-workers described the (3 + 2) cycloaddition of *N*-methyl azomethine ylide, generated *in situ* from sarcosine and paraformaldehyde, with 2-substituted 3,5-dinitropyridines **208** (Scheme 102).^157^ The corresponding decahydrodipyrrolopyridine derivatives **209** resulting from the double addition of the dipole to the pyridine ring were isolated in moderate yields. When the process was extended to dinitropyridines containing a tertiary amine in the 2-position, the final products were obtained in poor yields (Scheme 102).^158^

**Scheme 103:**
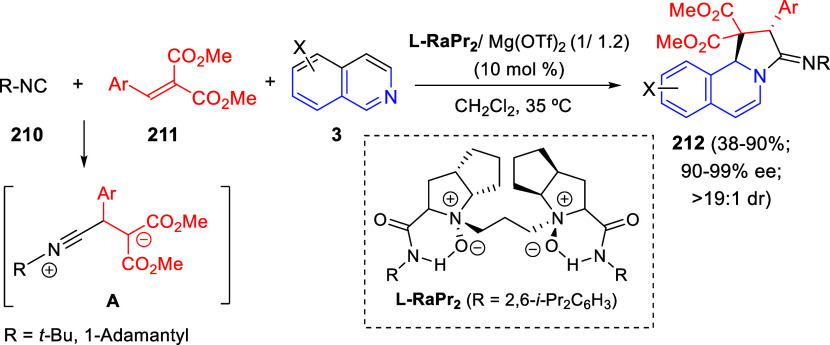
Enantioselective Dearomative (3 + 2) Annulation Reaction of Isoquinolines with Zwitterionic Intermediates from Isocyanides and Alkylidene Malonates

In 2019, Feng and co-workers reported a dearomative (3 + 2) annulation reaction involving isoquinolines, isocyanides, and alkylidene malonates (Scheme 103).^159^ The process started with the generation of zwitterionic intermediate **A** by means of the α-nucleophilic addition of isocyanides **210** to different aryl-substituted alkylidene malonates **211**. Then, this zwitterionic intermediate, as a 1,3-dipole, reacted with nonactivated isoquinolines **3** in the presence of Mg(OTf)_2_ as a Lewis acid and the chiral *N*,*N*′-dioxide ligand **L-RaPr**_**2**_, to furnish chiral 1,2-dihydroisoquinolines **212** in, generally, good yields and excellent diastereo- and enantioselectivities. Alkylidene malonates arising from aromatic aldehydes were well tolerated in the process, while those from aliphatic malonates were less efficient. Differently substituted quinolines were also suitable partners for this reaction. On the other hand, steric hindrance of the isocyanides had a crucial effect on the enantioselectivity, so the *t*-butyl and 1-adamantyl groups exhibited very good enantiocontrol, while the less bulky *i*-propyl, 2-naphythyl, and CH_2_CO_2_Me groups afforded the final products with much lower enantioselectivities.

**Scheme 104:**
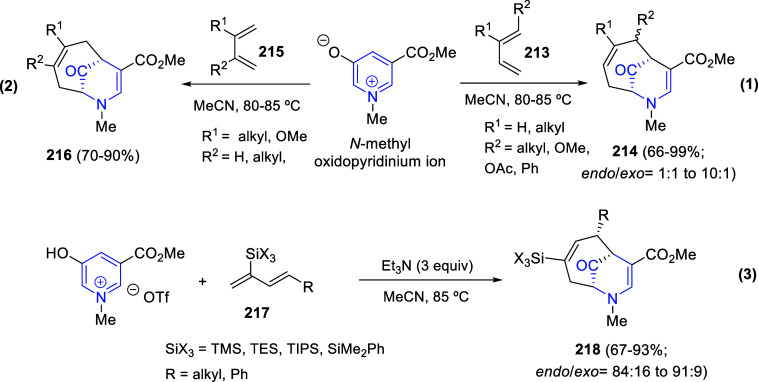
(4 + 3) Cycloaddition Reactions of *N*-Methyloxidopyridinium Ion with Dienes

In 2017, Krenske, Harmata, and co-workers reported the (4 + 3) cycloaddition reaction of oxidopyridinium ions derived from methyl 5-hydroxynicotinate with dienes in the presence of trimethylamine (Scheme 104).^160^ With 1-substituted and 1,2-disubstituted dienes **213**, *N*-containing cycloadducts **214** were obtained in good yields with excellent regioselectivity. However, the *endo*/*exo* selectivity was generally low (*endo*:*exo* = 1:1) except for the 1-phenyl-substituted diene (Scheme 104, eq 1). The authors also examined 2-substituted and 2,3-disubstituted dienes **215**. Their reaction with the *N*-methyloxidopyridinium ion took place successfully with symmetrical dienes, giving rise to single bicyclic cycloadducts **216**, whereas reactions with unsymmetrical dienes were not regioselective (Scheme 104, eq 2).

Later on, the authors developed a diastereoselective variant of this (4 + 3) cycloaddition protocol with the *N*-methyloxidopyridinium ion, which achieved high levels of *endo* selectivity, by using a 2-silyl substituent on the diene as a directing group (Scheme 104, eq 3).^161^ Thus, heating the oxidopyridinium precursor in acetonitrile with triethylamine and different 2-trialkyl(phenyl)silyl-4-alkylbutadienes **217**, the corresponding bicyclic adducts **218** were isolated in good yields and excellent *endo* selectivity, which was rationalized in terms of steric effects. The scope and optimization of this (4 + 3) cycloaddition reaction of oxidopyridinium ions to afford the 7-azabicyclo[4.3.1]decane ring system were compiled by the authors in 2021.^162^

**Scheme 105:**
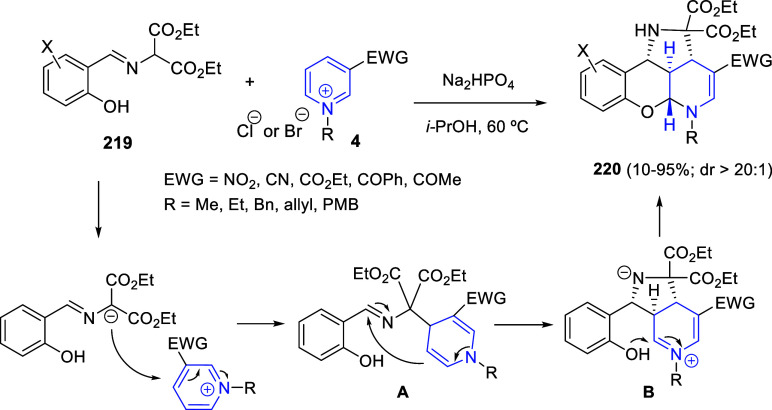
Dearomative Trifunctionalization of Pyridinium Salts by Reaction with Multifunctional *o*-Hydroxyl Aromatic Azomethine Ylides

Recently, Bu, Zhao, and Wang reported a highly diastereoselective dearomative trifunctionalization of pyridinium salts **4** with *o*-hydroxyaromatic azomethine ylides **219**, containing two nucleophilic sites with different reactivities and one electrophilic site (Scheme 105).^163^ Through a formal intermolecular azomethine ylide cycloaddition followed by an intramolecular *O*-addition, chroman-pyrrolidine-tetrahydropyridine tetracyclic structures **220** were achieved in generally good yields and excellent diastereoselectivities. A wide variety of substituents were tolerated on the aromatic azomethine ylide, regardless their position or electronic properties. Regarding the scope of pyridinium salts, various alkyl groups on the nitrogen atom were tolerated, as well as several electron-withdrawing groups at the 3-position, although with ester and ketone substituents yields decreased significantly. The presence of the electron-withdrawing group at the 3-position of the pyridinium salt would promote the regioselective nucleophilic addition at the 4-position, rendering a bis-enamine intermediate **A**, which would close the 5-membered ring, generating an iminium ion **B**, susceptible to react with the *ortho*-hydroxyl group (Scheme 105).

**Scheme 106:**
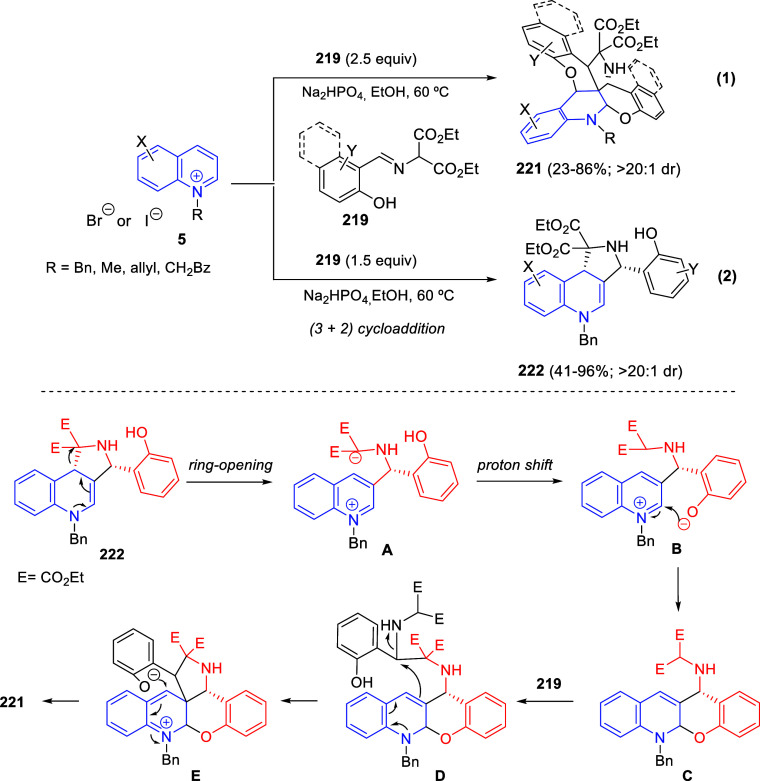
Dearomative Multifunctionalization of Quinolinium Salts through Reaction with *o*-Hydroxyl Aromatic Azomethines

In 2022, Wang’s research group disclosed the dearomative periphery multifunctionalization of quinolinium salts through reaction with *o*-hydroxyl aromatic azomethines **219** (Scheme 106).^164^ On the one hand, when 2.5 equiv of azomethine and 2.0 equiv of Na_2_HPO_4_ as a base were employed, an unexpected cascade reaction took place leading to highly encumbered polycycles **221** as single diastereoisomers in generally good yields (Scheme 106, eq 1). These products contained one tetrahydroquinoline, two chromans and one pyrrolidine ring. Regarding the scope of the process, it tolerates a wide range of substituents on the quinolinium salt, although those with electron-donating groups gave lower yields. Likewise, a variety of substituents were allowed on the aromatic azomethine ylide, regardless their position or electronic properties. On the other hand, when the ratio of azomethine ylide/quinolinium salt was 1:1, the reaction stopped after the (3 + 2) cycloaddition reaction and compounds **222** were isolated, again as single diastereoisomers (Scheme 106, eq 2).

In order to explain the results observed, the authors proposed a plausible mechanism that would start with a regio- and diastereoselective (3 + 2) cycloaddition between the quinolinium salt and the azomethine ylide to form compounds **222**. These would undergo ring-opening to deliver intermediate **A** (Scheme 106), which would evolve through intramolecular proton transfer to intermediate **B** followed by intramolecular cycloaddition to give intermediate **C**. Then, a second equivalent of azomethine would undergo nucleophilic addition to the imine functionality, rendering intermediate **D**. This would participate in an intramolecular annulation with release of diethyl 2-amino malonate. Final proton transfer and intramolecular cyclization would account for the formation of the products **221**.

**Scheme 107:**
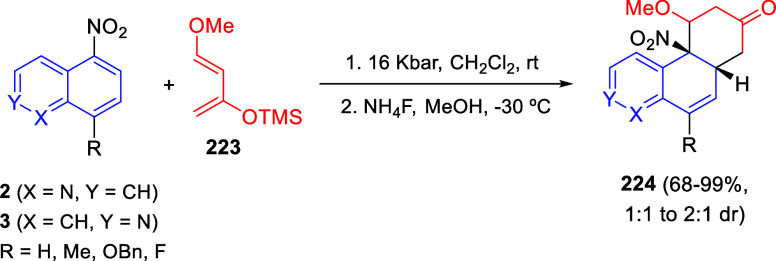
Dearomatization of Nitro(iso)quinolines through (4 + 2) Cycloaddition with Silyloxydienes under High Pressure

The last work within this section was recently accomplished by Chataigner and co-workers and it dealed with the dearomatization of 5-nitro(iso)quinolines acting as dienophiles in (4 + 2) cycloaddition reactions with silyloxydienes **223** under high pressure, without thermal or chemical activation (Scheme 107).^165^ The process took place at room temperature and produced the dearomatization of the arene moiety. After hydrolysis of the silylenol ether, polycyclic scaffolds **224** were obtained with complete regioselectivity, in good yields, as nearly equimolecular mixtures of diastereoisomers at the methoxy-containing stereocenter. Moreover, quinolines **2** bearing the nitro group at the C6-, C7-, and C8-positions also rendered the corresponding cycloadducts, again as mixtures of diastereoisomers. In an attempt to improve the diastereoselectivity of the process, the authors employed silyloxydienes bearing bulky groups (such as *t*-BuMe_2_); however, a 2:1 ratio of *trans*/*cis* diastereoisomers was achieved.

## 4 INTRAMOLECULAR CYCLIZATIONS

Cyclization reactions that entail the dearomatization of pyridines, quinolines, and isoquinolines usually take advantage of the nucleophilicity of the nitrogen atom. Accordingly, the generation of highly electrophilic species, usually by means of a metal-catalyzed process, favors the intramolecular nucleophilic addition of the heteroaromatic ring, ending in its final dearomatization. Alternatively, the formation of nucleophilic species, such as enolates, in heteroarene substituents and further treatment with electrophiles, can generate intermediates that would evolve with subsequent intramolecular cyclization in a tandem fashion, rendering multifunctionalized dearomatized polyheterocycles.

For a better understanding, this section is subdivided in metal-catalyzed and base-catalyzed processes.

### 4.1 Metal-Catalyzed Intramolecular Dearomatizations

#### 4.1.1 Copper-Catalyzed Protocols

**Scheme 108:**
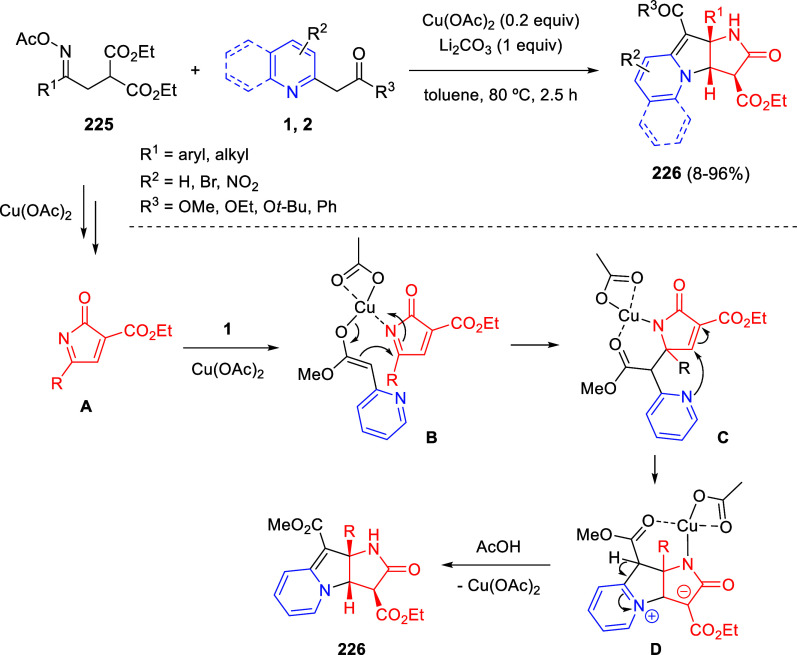
Copper-Catalyzed Bis-annulations of Malonate-Tethered *O*-Acyl Oximes with Pyridine Derivatives

In 2021, Miao, Yang, and co-workers described a copper-catalyzed bis-annulation reaction of malonate-tethered *O*-acyl oximes **225** with pyridine and quinoline derivatives to construct dihydroindolizine-fused pyrrolidinones **226** (Scheme 108).^166^ This approach relies on the *in situ* generation of a pyrrol-2-one intermediate (**A**) from the *O*-acyl oxime, mediated by a copper catalyst. This pyrrolone intermediate underwent nucleophilic addition of electron-withdrawing group-substituted 2-methylpyridines **1**. Both, aromatic and aliphatic substituted *O*-acetyl ketoximes **225** were well tolerated in the reaction, whereas esters or ketones could be used as electron withdrawing substituents on 2-methylpyridines **1**. Importantly, the presence of a carbonyl group on the pyridine counterpart was essential for coordination to the copper catalyst, since switching the carbonyl-containing group to another electron-withdrawing group completely inhibit the formation of the final product.

Regarding the mechanism of this transformation, after the formation of the key pyrrolone intermediate **A**, triggered by *in situ* generated Cu(I) from Cu(OAc)_2_, the carbonyl group of the pyridine would coordinate with copper to generate complex **B**, which would undergo regioselective intramolecular enolate addition to form intermediate **C** (Scheme 108). Subsequent intramolecular aza-Michael addition would generate zwitterion **D**, which, after intramolecular proton transfer would produce the final polycyclic products **226** in a highly stereoselective manner.

**Scheme 109:**
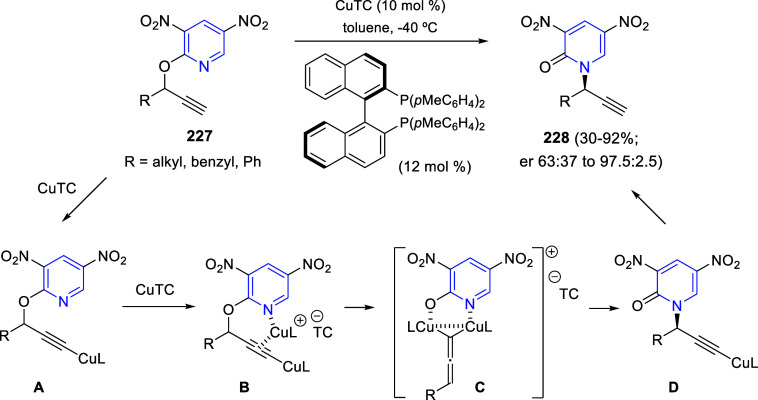
Enantioseelctive Copper-Catalyzed *O*-to-*N* (1,3)-Rearrangement of 2-Propargyloxy-Pyridines

An enantioselective copper-catalyzed *O*-to-*N* formal (1,3)-rearrangement of 2-propargyloxypyridines was reported by Cordier and co-workers (Scheme 109).^167^ The process employed copper(I) thiophene-2-carboxylate (CuTC) and a chiral diphosphine ligand as the catalytic system that promoted the enantioconvergent dearomative rearrangement of racemic 2-propargyloxypyridines **227** to form *N*-propargylic-2-pyridones **228** in generally good yields with high enantioselectivity. Only 3,5-dinitropyridine substrates were able to perform the rearrangement, although many different functional groups were tolerated at the propargylic position.

Several experiments were conducted in order to understand the stereochemical and kinetic features of this transformation, concluding that, after Cu-acetylide formation (**A**), a second Cu coordination to the pyridyl nitrogen and the triple bond would lead to a bimetallic intermediate **B** (Scheme 109). This would facilitate the C–O bond cleavage, as the turnover limiting step, to generate bimetallic copper-pyridone intermediate **C**. Collapse of this intermediate would allow the C–N bond formation, leading to Cu-acetylide **D**, which would close the catalytic cycle by protodecupration.

**Scheme 110:**
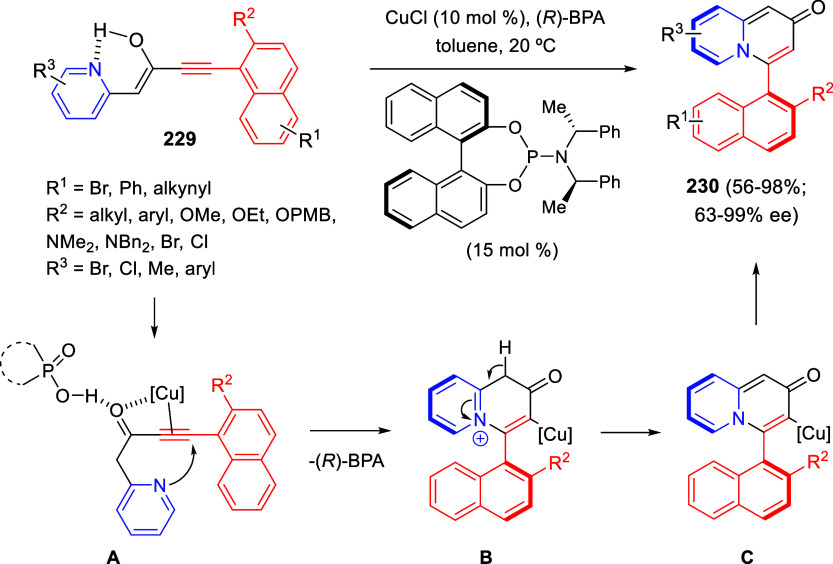
Brønsted Acid-Enhanced Copper-Catalyzed Atroposelective Cycloisomerization to Axially Chiral Arylquinolizones via Dearomatization of Pyridine

In 2022, He and Yi described a CuCl-catalyzed intramolecular atroposelective cycloisomerization of pyridine alkynes **229** for the enantioselective synthesis of axially chiral arylquinolizinones **230** (Scheme 110).^168^ The dearomatization of the pyridine ring was enhanced by the addition of chiral BINOL phosphoric acid (BPA), which cooperates with the chiral Cu-catalyst by stabilizing the ketone form of the substrate instead of the enol form in intermediate **A** (Scheme 110). Then, the intramolecular nucleophilic addition of the pyridyl nitrogen to the activated alkyne would form the chiral intermediate **B**. Dearomatization by deprotonation and final protodecupration would occur to yield products **230** and regenerate the copper catalyst. A great variety of substituents both on the naphthol core (R^1^ and R^2^) and on the pyridine ring (R^3^) were tolerated in this reaction, although enantioselectivity was improved with bulky substituents, especially at the R^2^-position. In addition, the synthetic utility of the developed strategy was demonstrated through the synthesis of several axially chiral arylquinolizone analogues, such as a thiourea derivative that was tested as a new catalyst for an enantioselective Michael addition.

#### 4.1.2 Palladium-Catalyzed Protocols

**Scheme 111:**
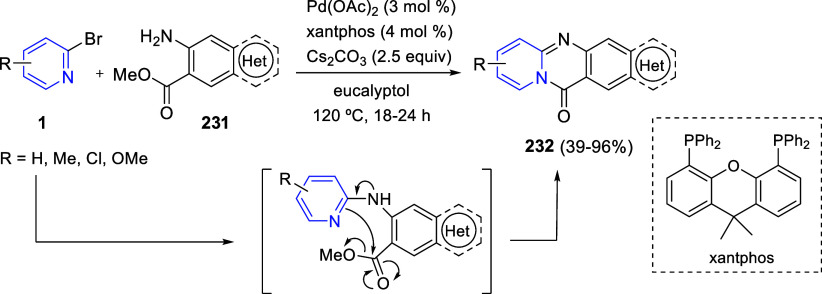
Buchwald–Hartwig Coupling/Pyridine Dearomatization Sequence in Eucalyptol

The Pd-catalyzed reaction between 2-bromopyridines **1** and polycyclic anthranilic acid derivatives **231** allowed Berteina-Raboin and co-workers the synthesis of a library of polyheterocyclic compounds **232** derived from the 11*H*-pyrido[2,1-*b*]quinazolin-11-one ring system (Scheme 111).^169^ Since the direct nucleophilic aromatic substitution did not work, Pd catalysis with xantphos phosphine ligand was applied to perform the Buchwald-Hartwig amination, followed by dearomative cyclization to afford the tetracyclic heterocycles **232** in moderate to excellent yields. The authors found that traditional solvents such as toluene or dioxane could be replaced by the thermally stable and sustainable solvent eucalyptol.

**Scheme 112:**
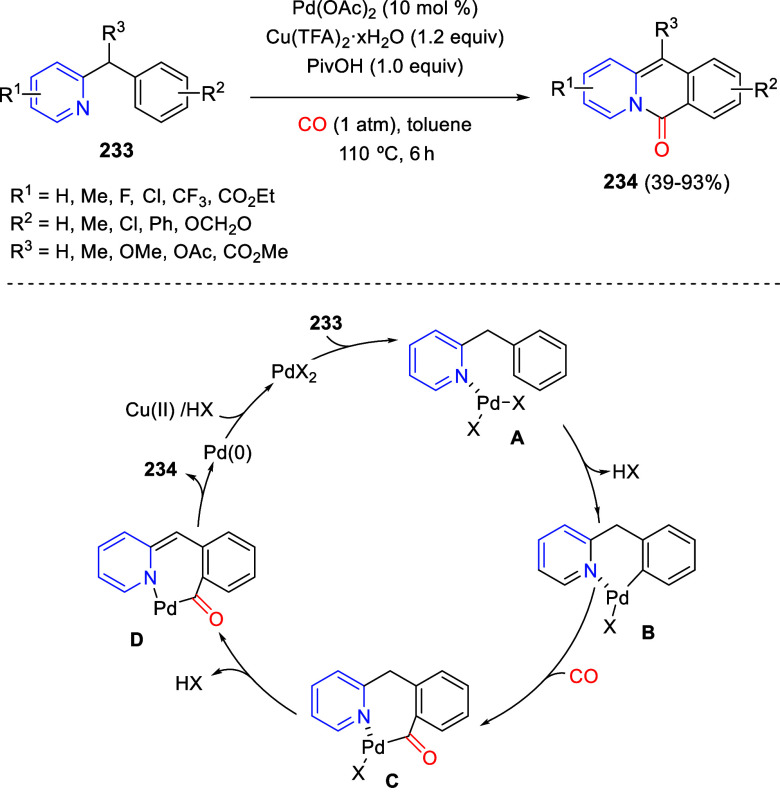
Pd-Catalyzed C(sp^2^)–H Cyclizative Carbonylation of 2-Benzylpyridines

The Pd-catalyzed dearomative cyclocarbonylation reaction of pyridine derivatives has been employed for the synthesis of functionalized quinolizinones and related compounds. For example, this strategy was applied to the synthesis of pyridoisoquinolinones **234** from 2-benzylpyridines **233** (Scheme 112).^170^ The piridinyl moiety acted as a directing group for the C(*sp*^2^)–H activation and as an internal nucleophile for the intramolecular pyridocarbonylation. The reaction was compatible with electronically different substituents on both rings (R^1^, R^2^), as well as at the benzylic position of the substrate (R^3^), and also with 1-benzylisoquinoline as starting material. However, it was very sensitive to steric hindrance close to the reacting positions.

Mechanistically, coordination of the pyridine nitrogen with Pd(II) would generate complex **A**, which would activate the *ortho*-C–H bond of the benzene ring through cyclopalladation to produce intermediate **B** (Scheme 112). Afterward, migratory insertion of CO into the C–Pd bond would form a seven-membered palladacycle **C**. Deprotonation and ligand exchange would afford the dearomatized intermediate **D** and, finally, reductive elimination would provide the cyclocarbonylation product **234** with the simultaneous release of Pd(0). This would be reoxidized to Pd(II) species by Cu(TFA)_2_·*x*H_2_O in the presence of pivalic acid to complete the catalytic cycle.

A variant of this palladium-catalyzed cyclizative carbonylation of 2-benzylpyridines was accomplished by employing a combination of 8 equiv of formic acid and acetic anhydride to *in situ* generate the CO.^171^

**Scheme 113:**
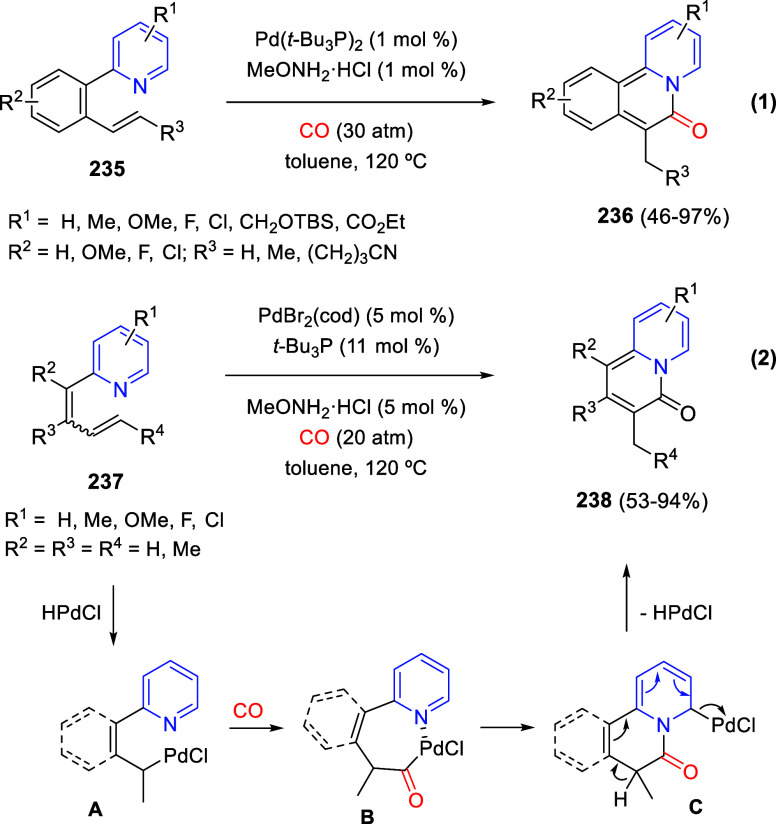
Palladium-Catalyzed Hydrocarbonylative Cyclization of Pyridine-Tethered Alkenes and Dienes

A related palladium-catalyzed hydrocarbonylative cyclization of pyridine-tethered alkenes or dienes was developed by the group of Huang for the synthesis of substituted quinolizinone derivatives (Scheme 113).^172^ The optimized reaction conditions entailed the use of Pd(*t*-Bu_3_P)_2_ or PdBr_2_(cod) (cod = 1,5-cyclooctadiene)/*t*-Bu_3_P as the catalysts and MeONH_2_·HCl as an acid additive. The reaction with 2-(2-vinylphenyl)pyridines **235** was compatible with electronically diverse groups on both rings, although it was less efficient for substrates bearing electron-withdrawing groups on the pyridine ring (Scheme 113, eq 1). In addition, replacement of the pyridine with quinoline or isoquinoline moieties was also tolerated. On the other hand, the reaction with pyridine dienes **237** requiered higher catalyst loadings to obtain the desired quinolizinones **238** in good yields (Scheme 113, eq 2). Both *E* and *Z* isomers performed successfully in the reaction and yields were not affected by the electronic properties of the substituents.

On the basis of several control experiments, a plausible reaction mechanism was proposed. Upon formation of the Pd-H species, C=C bond insertion, followed by carbonylation to form the seven-membered palladacycle **B**, and formal C=N bond insertion into the Pd-acyl bond, would generate the key dearomatized intermediate **C** (Scheme 113). Then, reductive elimination promoted by the base would give the final products and Pd(0), which would react with MeONH_2_·HCl to form the active Pd-H species.

**Scheme 114:**
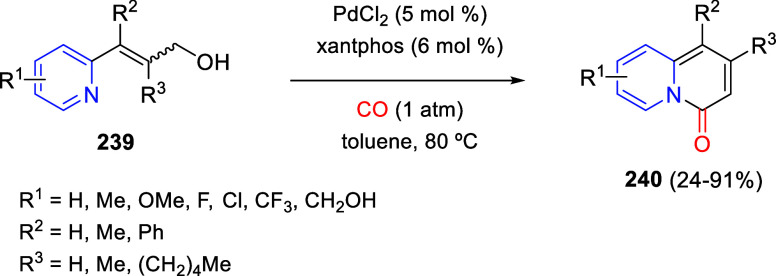
Palladium-Catalyzed Dearomative Cyclocarbonylation of Allyl Alcohols

Huang research group had previously reported a similar palladium-catalyzed dearomative cyclocarbonylation of pyridines for the synthesis of quinolizinones employing azaarene-substituted allylamines as starting materials.^173^ In 2021, the same group described an extension of this methodology that involved the use of easily obtainable allyl alcohols, with the advantage of having water as byproduct (Scheme 114).^174^ To accelerate the leaving rate of the hydroxyl group, acid chloride generated *in situ* from the reduction of palladium(II) to palladium(0) was utilized, thus avoiding the need of any external additive to perform the reaction. Substituted pyridine-allyl alcohols **239** were transformed into quinolizinones **240** in the presence of CO (1 atm) and PdCl_2_/xantphos as the catalytic system. Chemical yields were generally good with different substituents on the pyridine ring, although the reaction was less efficient with substituents at the *ortho*-position. In addition, alkyl and phenyl substituents in the double bond were tolerated.

**Scheme 115:**
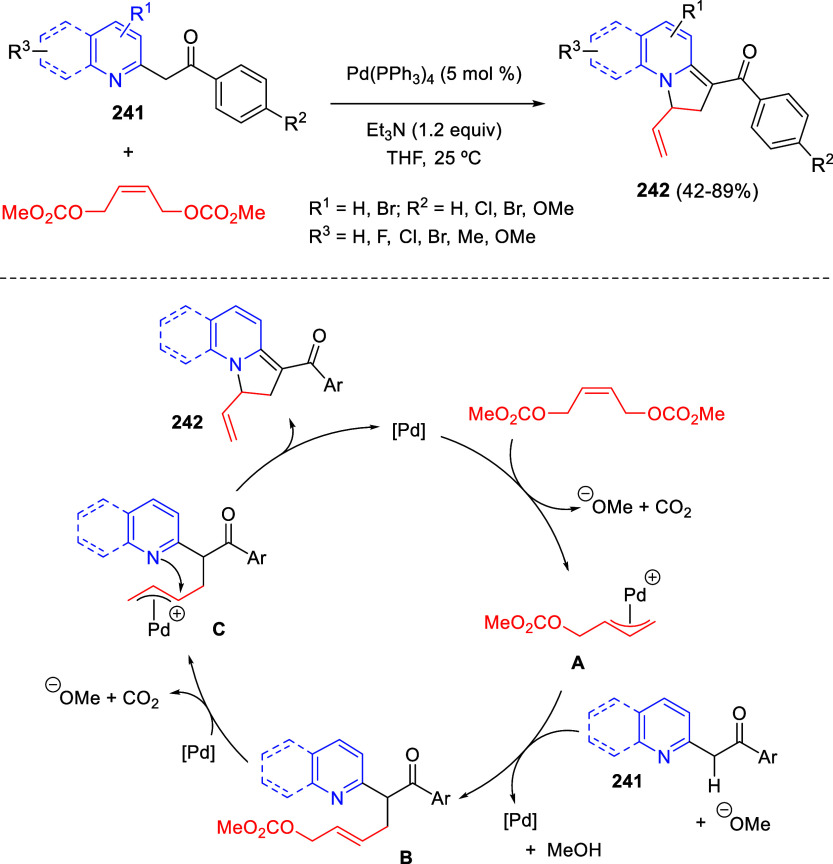
Tandem Pd-Catalyzed Allylic Alkylation/Allylic Dearomatization Reaction of *N*-Heteroarenes

A tandem Pd-catalyzed intermolecular allylic alkylation/intramolecular allylic dearomatization reaction of 2-benzoylmethyl-substituted pyridines and quinolines **241** was reported by You and co-workers (Scheme 115).^175^ These heteroarenes reacted as bis-nucleophiles, through the nitrogen and the benzylic positions, with but-2-ene-1,4-diyl dimethyl dicarbonate as the bis-electrophile, to obtain 2,3-dihydroindolizine and 1,2-dihydropyrrolo[1,2-*a*]quinolin derivatives **242** in moderate to good yields. The reaction was performed in the presence of Et_3_N as a base, which had a great influence on the reaction outcome. Pyridine-derived substrates bearing electron-withdrawing groups at the *para*-position of the phenyl ring gave better yields than those with electron-donating groups. Quinoline substrates substituted at the C4-position performed worse than those bearing substituents at the C6- or C7-positions.

A plausible reaction pathway would start with the oxidative addition of Pd(0) to dicarbonate followed by decarboxylation to generate the π-allylpalladium **A** (Scheme 115). Nucleophilic addition of the carbanion derived from α-deprotonation of substrate **241** would furnish the allylic substituted product **B**. Then, a new π-allylpalladium intermediate **C** would facilitate the intramolecular allylic dearomatization reaction to give product **242** and regenerate the Pd(0) catalyst.

**Scheme 116:**
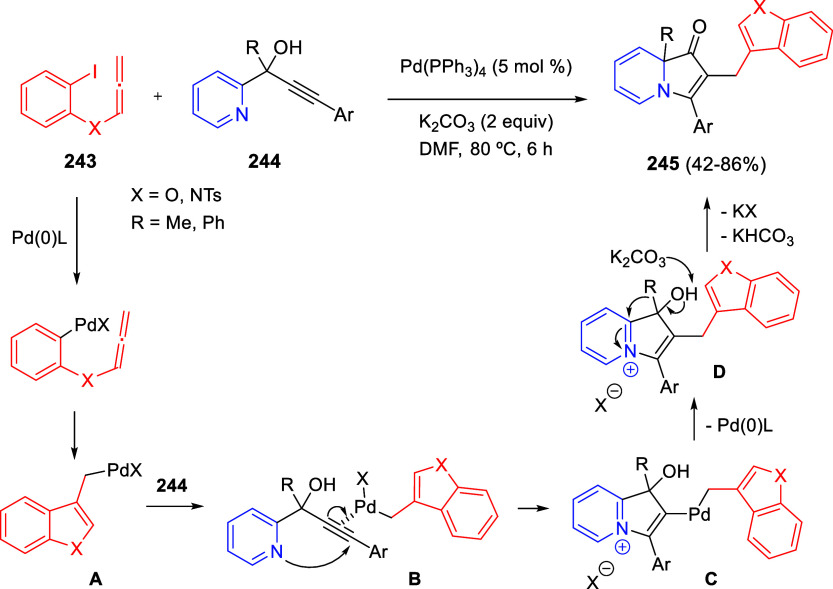
Palladium-Catalyzed Difunctionalization of Allenes through Relay Coupling with Propargylic Pyridines

The last example of a Pd-catalyzed intramolecular dearomatization reaction was reported by Liu and co-workers for the synthesis of indolizinone-containing bis-heterocycles via palladium-catalyzed difunctionalization of allenes through relay coupling with propargylic pyridines (Scheme 116).^176^ The authors employed a Heck-type carbometalation of aryl iodide-tethered allenes **243** to intercept the σ-alkylmetal intermediate with tertiary propargylic alcohols **244**, thus facilitating the cyclization and formation of indolizinone-containing bis-heterocycles **245**. The reaction was compatible with different substitution patterns on the benzene ring of propargylic pyridines **244**, and both methyl and phenyl groups at the propargylic position (R) underwent the reaction to render the corresponding bis-heterocycles **245**, bearing an indole or a benzofuran moiety, in moderate to good yields.

After the oxidative addition of palladium(0) to the aryl-halogen bond of iodides **243**, an intramolecular carbopalladation would give the σ-alkylpalladium species **A** (Scheme 116). Then, coordination of the triple bond of propargylic pyridines **244** to palladium would form complex **B**, which would cyclize by nucleophilic addition of the pyridyl nitrogen to the activated alkyne. Subsequent reductive elimination of Pd(0) would generate pyridinium cation **D** and, after deprotonation and 1,2-migration of the R group, the dearomatized product would be formed.

#### 4.1.3 Rhodium-Catalyzed Protocols

**Scheme 117:**
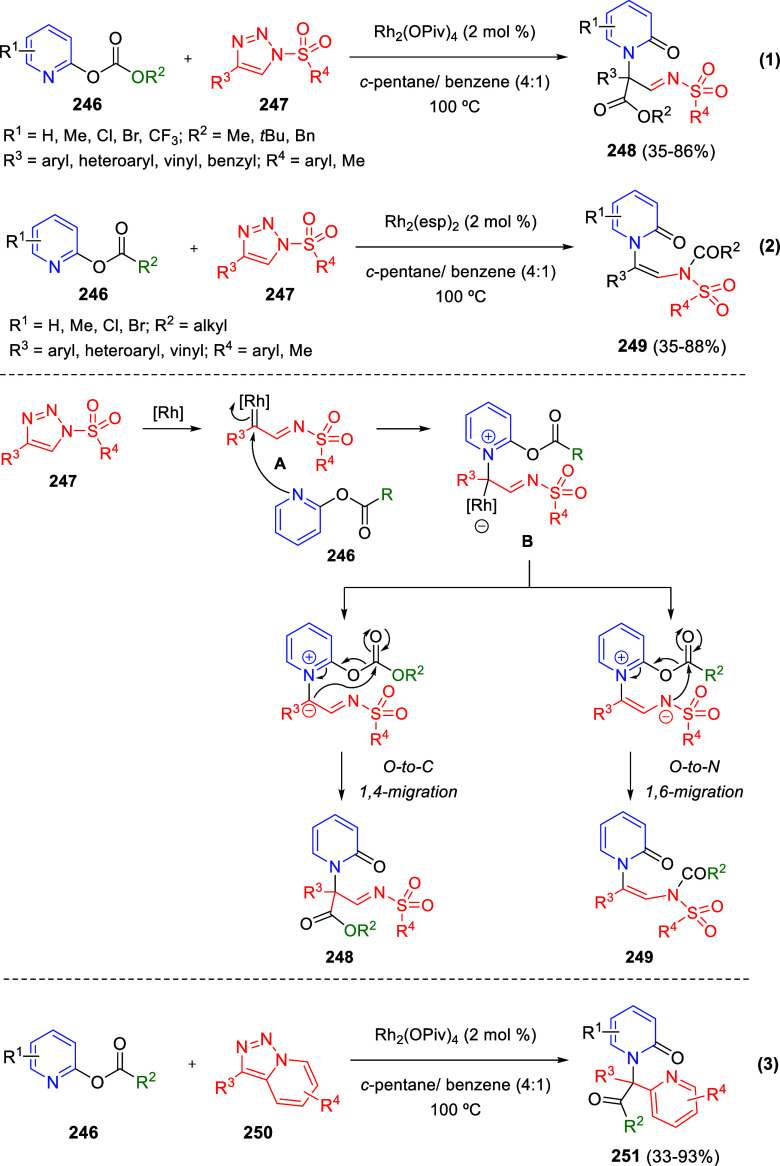
Dearomative Migratory Rearrangement of 2-Oxypyridines with *N*-Sulfonyl-triazoles and Pyridotriazoles

In 2020, Chen and Sun described a dearomative migratory rearrangement reaction of 2-oxopyridines **246** with *N*-sulfonyl-1,2,3-triazoles **247** under rhodium catalysis for the synthesis of *N*-substituted 2-pyridones (Scheme 117).^177^ This transformation proceeded through highly electrophilic α-imino rhodium carbene species **A** (formed by reaction of *N*-sulfonyl-1,2,3-triazoles with rhodium complexes) which, after the nucleophilic addition of 2-oxypyridines, formed ylide intermediates **B**. These ylides evolved through *O*-to-*C* 1,4-acyl migration, in the case of 2-carbonate pyridine substrates; or through *O*-to-*N* 1,6-migration, in 2-acyloxy pyridines.

For the 1,4-migration reaction, several alkyl groups on the carbonate and substituents of different electronic nature on the 2-carbonate pyridine ring **246** were tolerated; while triazoles **247** with aromatic substituents (R^3^ and R^4^) gave the corresponding products **248** in higher yields (Scheme 117, eq 1). On the other hand, 2-acyloxypyridine substrates reacted properly only with alkyl ester groups, while substitution at the pyridine ring was well tolerated. Triazoles containing aryl and heteroaryl groups (R^3^) reacted well and, for the sulfonyl amide moiety, aryl substituents (R^4^) gave better yields than the methyl group (Scheme 117, eq 2). Moreover, when pyridotriazoles **250** were used instead of *N*-sulfonyl triazoles, the reaction proceeded via 1,4-acyl migration and *N*-substituted 2-pyridones **251** were obtained regardless of the type of *O*-acyl substituent in the 2-position of the pyridine substrates **246** (Scheme 117, eq 3).

**Scheme 118:**
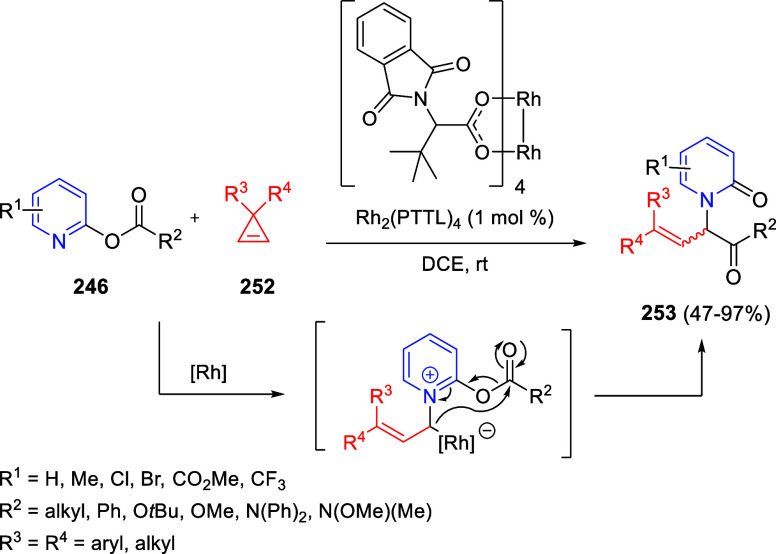
Rhodium-Catalyzed Dearomative Rearrangement of 2-Oxypyridines with Cyclopropenes

The same authors extended this rhodium-catalyzed dearomative rearrangement of 2-oxopyridines employing vinyl rhodium carbenes generated *in situ* from cyclopropenes **252** (Scheme 118).^178^ Diaryl and dialkyl cyclopropenes (R^3^ = R^4^) gave the final *N*-alkylated 2-pyridone derivatives **253** in good yields, while aryl-alkyl substituted cyclopropenes (R^3^ ≠ R^4^) gave mixtures of *E*/*Z* isomers.

**Scheme 119:**
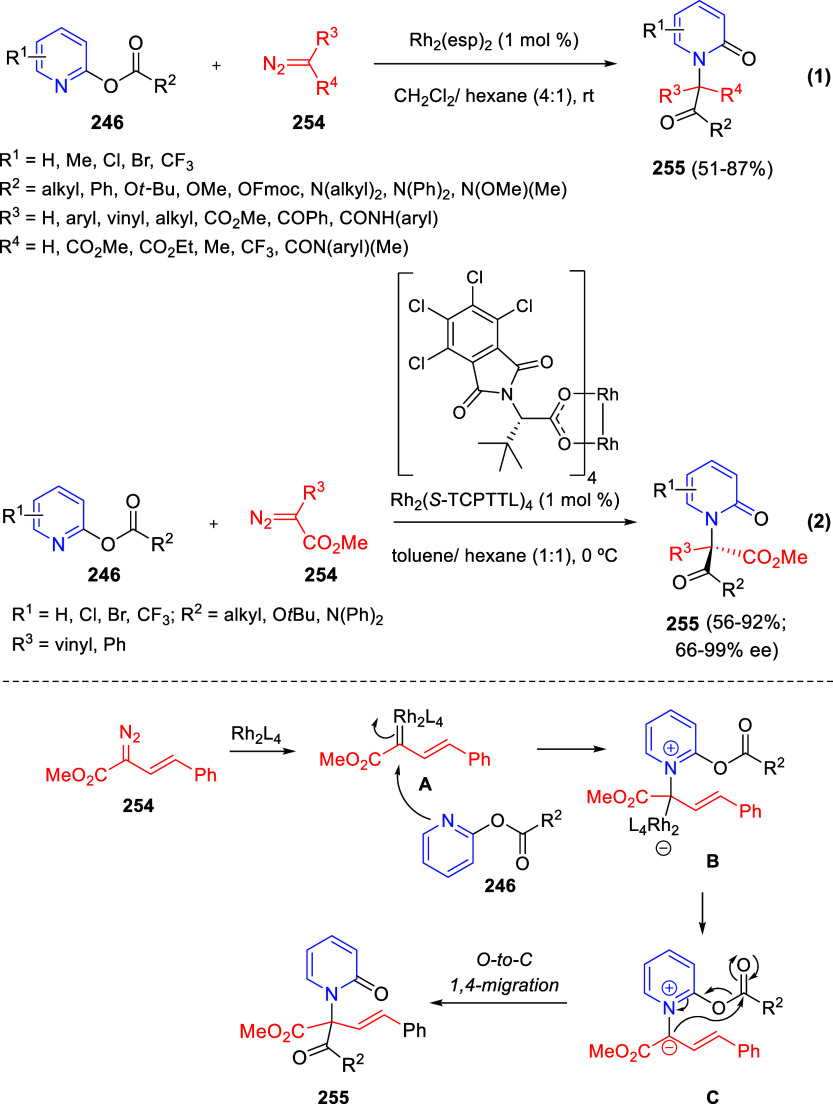
Rhodium-Catalyzed Dearomative Migratory Rearrangement of 2-Oxypyridines with Diazocompounds

Finally, the same group had previously reported a rhodium-catalyzed dearomatization reaction of substituted 2-oxypyridines **246** with diazocompounds **254** as the alkylation reagents, for the synthesis of *N*-substituted 2-pyridones **255** (Scheme 119).^179^ This transformation was catalyzed by Du Bois’ catalyst, [Rh_2_(esp)_2_], and many different substitution patterns could be used on both substrates, to obtain the desired products in moderate to good yields (Scheme 119, eq 1). The enantioselective version of this protocol was achieved employing dirhodium tetrakis[*N*-tetrachlorophthaloyl-(*S*)-*tert*-leucinate], [Rh_2_(*S*-TCPTTL)_4_], as the chiral catalyst (Scheme 119, eq 2).

The authors conducted computational studies in order to understand the reaction mechanism. Thus, aryl vinyldiazoacetate **254** would initially form rhodium carbene **A**. Then, addition of the pyridyl nitrogen would generate rhodium ylide intermediate **B**, which would evolve to pyridine ylide **C** after rhodium dissociation (Scheme 119). Rotation of the C–N bond in this pyridine ylide is restricted by the substituents and therefore, axial chirality might be transferred to the quaternary stereogenic center of the *N*-substituted 2-pyridones **255**, resulting from the 1,4-acyl rearrangement event.

#### 4.1.4 Protocols Catalyzed by Other Metals

**Scheme 120:**
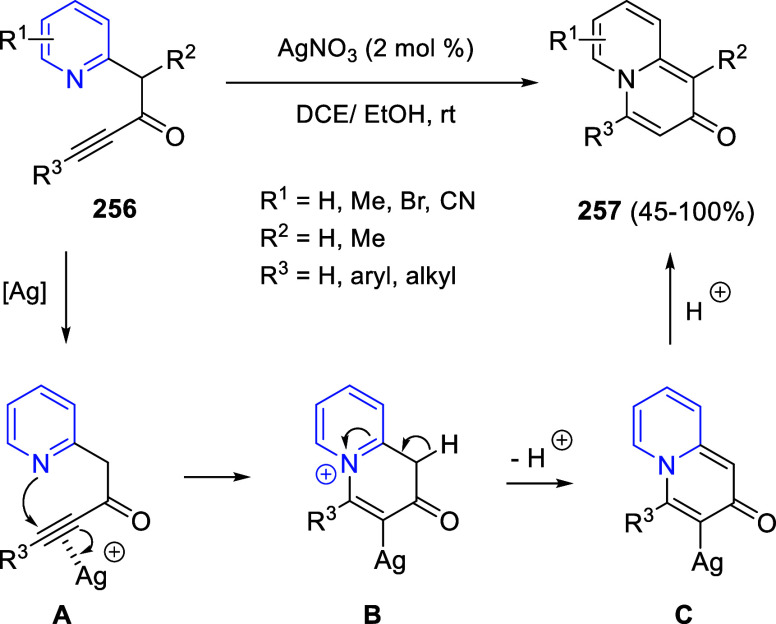
Silver(I)-Catalyzed Dearomatization of Pyridine-ynones for the Synthesis of Quinolizinone Derivatives

The quinolizidine skeleton and its derivatives are key pharmacophores in many biological- and pharmaceutically active products,^180^ which makes them highly attractive synthetic targets. In this context, the intramolecular dearomatization reaction of pyridinoalkynes is a convenient catalytic method to access functionalized quinolizinones. The activation of alkynes with π-acidic catalysts to promote the cyclization can be followed by protodemetalation or functionalization. Unsworth and Taylor employed this strategy to cyclize pyridine-ynones **256** into quinolizinones **257** at room temperature under silver(I) catalysis (Scheme 120).^181^ Good to excellent yields were obtained with cyano, bromo or methyl substituents on the pyridine ring (R^1^), and also with isoquinoline-ynone as starting material. Moreover, the reaction proceeded in very good yields with both aromatic and aliphatic substituents on the alkyne (R^3^).

The ynone starting material would be activated by the silver(I) catalyst (**A**), promoting the intramolecular nucleophilic addition of the pyridine and forming pyridinium intermediate **B** (Scheme 120). Deprotonation at the α-keto position would form vinyl silver species **C**, which, after protodemetalation, would afford quinolizinone **257** and regenerate the silver(I) catalyst. In addition, the authors demonstrated the utility of this methodology through the five-step dearomative synthesis of the lasubine II alkaloid.

**Scheme 121:**
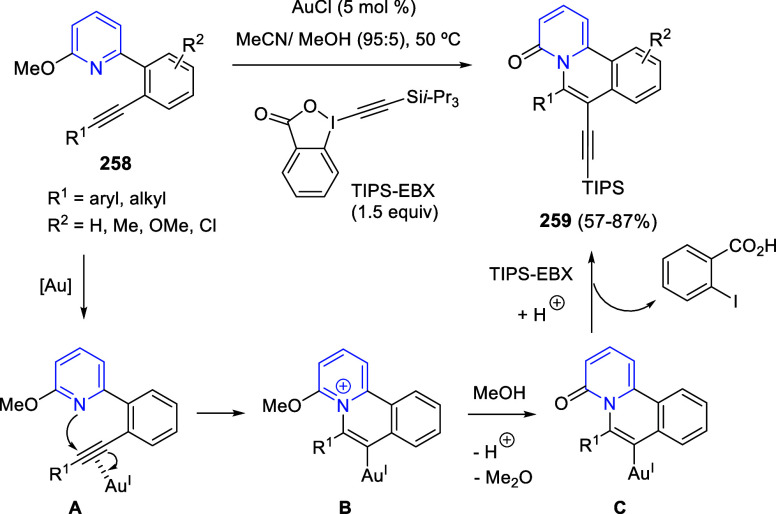
Gold-Catalyzed Aminoalkynylation of Alkynes for the Synthesis of Quinolizinone Derivatives

Similarly, Patil and co-workers described a gold-catalyzed aminoalkynylation of alkynes for the synthesis of quinolizinones (Scheme 121).^182^ Thus, the reaction of pyridine-alkynes **258** with 1-[(triisopropylsilyl)-ethynyl]-1,2-benziodoxol-3(1H)-one (TIPS-EBX) in the presence of a catalytic amount of AuCl afforded alkynylated quinolizinones **259** in good yields. Substrates bearing aromatic groups (R^1^ = aryl) efficiently underwent the aminoalkynylation reaction, and various substitution patterns on the phenyl ring (R^2^) were also tolerated.

A plausible mechanism would involve the alkyne activation by the gold catalyst (**A**), followed by nucleophilic addition of the pyridyl nitrogen to generate vinyl-gold intermediate **B** (Scheme 121). Demethylation by MeOH would lead to another vinyl-gold intermediate **C**, which would evolve to product **259** after alkynylation with TIPS-EBX, with concomitant regeneration of the catalyst.

**Scheme 122:**
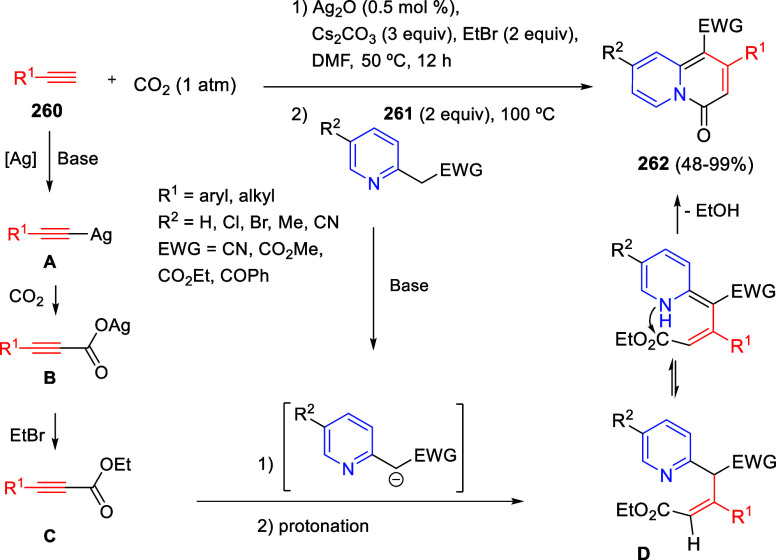
One-Pot Multicomponent Approach to Quinolizinones from 2-Substituted Pyridines via Orthogonal Tandem Catalysis

In 2018, the groups of Gong and Xu reported a one-pot multicomponent approach to quinolizinones via orthogonal tandem catalysis (OTC) (Scheme 122).^183^ In this process, CO_2_ was combined with a terminal alkyne **260** and a 2-substituted pyridine **261** in the presence of Ag_2_O, Cs_2_CO_3_ and EtBr and, under the optimized reaction conditions, a variety of substituted 4*H*-quinolizin-4-ones **262** were obtained in moderate to very good yields. The reaction was compatible with aliphatic and aromatic terminal alkynes, either with electron-withdrawing or electron-donating groups. In the pyridine counterpart, different substituents were tolerated at the 5-position. In addition, the replacement of the pyridine core with quinoline or isoquinoline was also possible.

Control experiments were carried out in order to obtain insight into the mechanism of this one-pot reaction. It would start with the activation of the terminal alkyne by Ag(I) species and Cs_2_CO_3_ to form silver acetylide intermediate **A** (Scheme 122). Insertion of CO_2_ into the Ag-alkyne bond followed by esterification with ethyl bromide would produces ethyl propynoate **C**. This alkynyl ester would be attacked by the anion from 2-substituted pyridines **261** to give alkene intermediate **D** after protonation. Tautomerization and intramolecular *N*-acylation of the pyridine would lead to the dearomatized final products **262**.

**Scheme 123:**
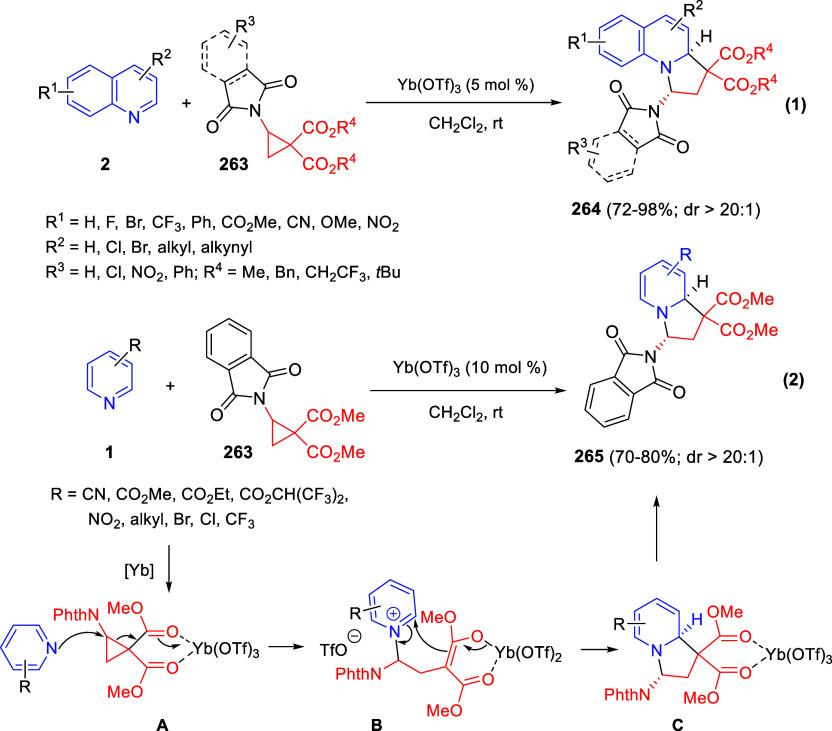
Yb-Catalyzed Dearomative (3 + 2) Annulation Reactions of Quinolines and Pyridines with 2-Aminocyclopropanes

The dearomative (3 + 2) annulation of pyridines, quinolines, and isoquinolines with aminocyclopropanes catalyzed by ytterbium(III) is a useful strategy for the synthesis of tetrahydroindolizine derivatives (Scheme 123).^184^ In this approach, the electron poor six-membered *N*-heterocycles acted as dipolarophiles in their reaction with imido-substituted donor–acceptor (DA) diester cyclopropanes **263**, achieving the corresponding products **264** in high yields with excellent diastereoselectivities as *anti*-isomers. A wide range of differently substituted quinolines **2** and DA aminocyclopropanes **263** were well tolerated in the reaction. Only the more hindered 2- and 8-substituted quinolines did not react under the optimized conditions, with the exception of 8-fluoroquinoline. Regarding the aminocyclopropane counterpart, substituted phthalimides, maleimide, succinimide, or 2,3-naphthalimide could be used, as well as different ester groups (Scheme 123, eq 1). In addition, several 4-substituted isoquinolines reacted equally well.

When pyridines **1** were employed, the reaction proved to be more difficult. Unsubstituted pyridine or pyridines substituted only with electron-donating groups did not render the final products, although pyridines substituted with electron-withdrawing groups were better electrophiles for the final cyclization and afforded the tetrahydroindolizine derivatives **265** in good yields and high diastereoselectivity (>20:1) (Scheme 123, eq 2). The final aminal products were converted into secondary and tertiary amines through iminium formation followed by reduction or nucleophile addition.

Based on control experiments and the known activation of DA diester cyclopropanes with Lewis acids, the author proposed a mechanism that would initiate by coordination of Yb(OTf)_3_ with the cyclopropane. Subsequent nucleophilic addition of the *N*-heterocycle would open the cyclopropane ring and form enolate intermediate **B** (Scheme 123). Final cyclization through enolate addition to the iminium cation and ligand exchange on ytterbium would close the catalytic cycle.

**Scheme 124:**
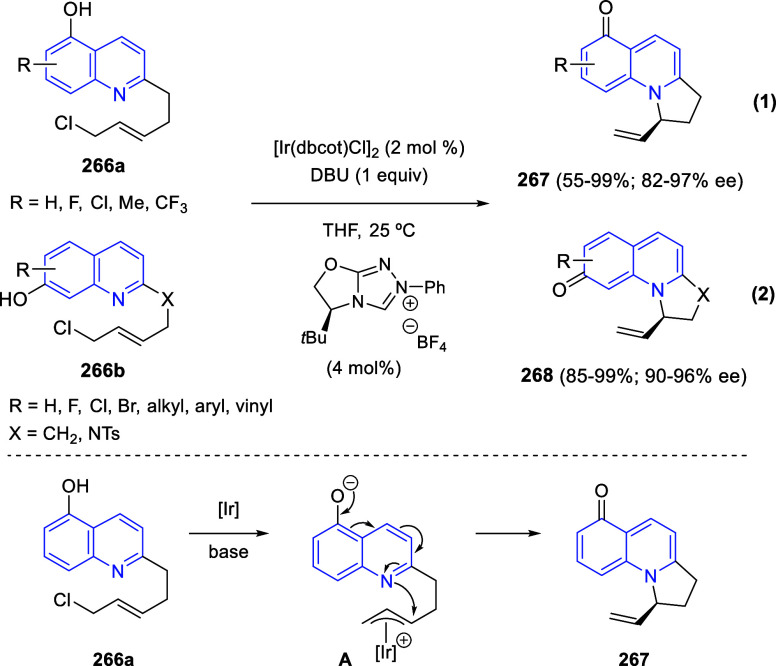
Iridium-Catalyzed Intramolecular Asymmetric Allylic Alkylation of Hydroxyquinolines

In 2018, Zheng and You reported an intramolecular asymmetric allylic alkylation of hydroxyquinolines catalyzed by an iridium/*N*-heterocyclic carbene (NHC) complex, which proceeded with the simultaneous weakening of the aromaticity of two consecutive aromatic rings (Scheme 124).^185^ The optimal reaction conditions entailed the catalyst derived from [Ir(dbcot)Cl]_2_ (dbcot = dibenzo[*a*,*e*]cyclooctatetraene), l-*t*-Butylalaninol-derived triazolium salt as precursor of the NHC chiral ligand and one equivalent of DBU as a base. In this manner, several dihydropyrroloquinolinones **267** were obtained in very good yields and high enantioselectivity from 5-hydroxyquinolines **266a** bearing electron-withdrawing groups at the C7- or C8-positions (Scheme 124, eq 1). 7-Hydroxyquinoline-derived allylic chlorides **266b** were also suitable substrates in the reaction and substituents at the C5- or C6-position of the quinoline core had little influence on the yield and enantioselectivity (Scheme 124, eq 2). An *N*-linkage between the allylic chloride and the quinoline ring was also well tolerated. The dearomative process would be facilitated by deprotonation of the acidic hydroxyl group and formation of the π-allylic complex **A**, followed by the intramolecular asymmetric allylic alkylation (Scheme 124). The authors demonstrated the utility of this methodology by the formal synthesis of the alkaloid (−)-gephyrotoxin.

**Scheme 125:**
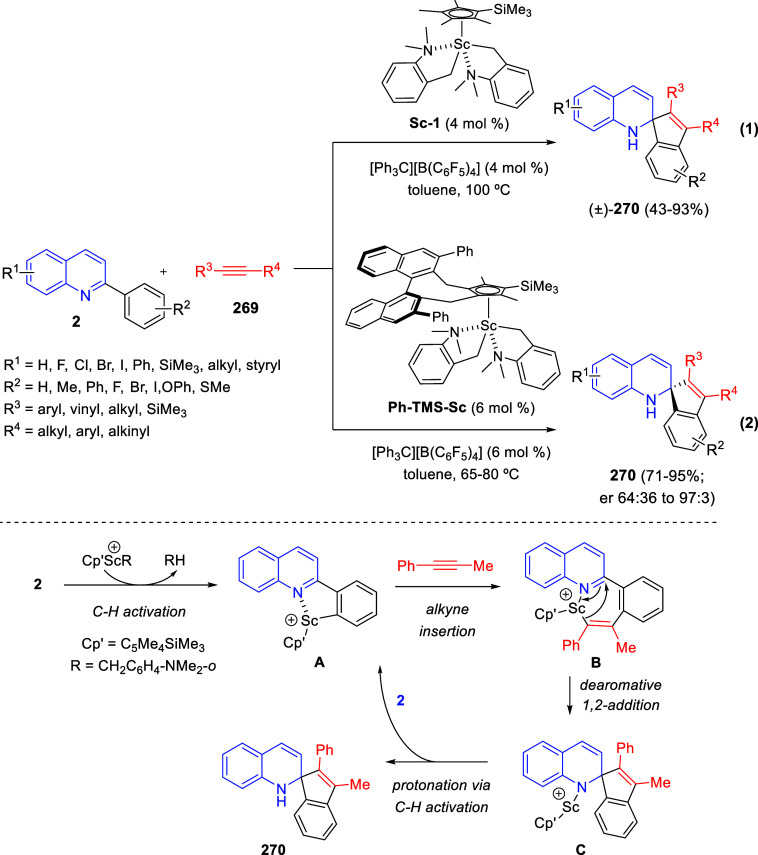
Scandium-Catalyzed Dearomative Spiro-annulation of Quinolines with Alkynes

The last example of this section is the scandium-catalyzed dearomative spiro-annulation of quinolines with alkynes developed by Hou and Luo in 2021 (Scheme 125). They found that the reaction of 2-arylquinolines **2** with internal alkynes **269** in the presence of C_5_Me_4_SiMe_3_-ligated scandium catalyst **Sc-1**, afforded exclusively the spiro-dihydroquinoline derivatives **270** containing a quaternary carbon stereocenter via dearomative (3 + 2) cyclization.^186^ The half-sandwich scandium catalyst **Sc-1** was able to catalyzed the spiro-annulation reaction in the presence of a wide range of functional groups as substituents on both phenyl rings of 2-arylquinolines **2**, including benzofused quinoline substrates (Scheme 125, eq 1). Similarly, the reaction was compatible with many different types of substituents on the internal alkynes and, remarkably, it was highly regiospecific. For alkynes bearing both aromatic and aliphatic substituents, the aromatic one ended adjacent to the quaternary stereocenter (R^3^), due to steric factors, while, in the case of alkynes bearing a silyl substituent directly bonded to the alkyne, this substituent was placed adjacent to the quaternary stereocenter, due to the electronic effects. Moreover, the authors found that the chiral half-sandwich scandium catalyst bearing binaphthyl-substituted cyclopentadienyl ligands **Ph-TMS-Sc** showed both high activity and high enantioselectivity for the construction of spiro-hydroquinolines **270** with a wide range of substituted 2-arylquinolines and alkynes (Scheme 125, eq 2).

Experimental and DFT studies revealed that the reaction would proceed through C–H activation of the 2-aryl substituent of quinoline substrate **2** to form phenyl-metalated intermediate **A**, followed by alkyne insertion into the Sc-aryl bond to generate scandium-alkenyl intermediate **B** and subsequent dearomative 1,2-addition of the Sc-alkenyl bond to the C=N unit of the quinoline ring to yield Sc-amido spiroquinoline intermediate **C** (Scheme 125). Finally, recoordination of substrate **2** to the Sc atom and its C–H activation would protonate the Sc-amido bond releasing the N–H free spiro-dihydroquinoline product **277** and regenerating the catalytic species.

### 4.2 Base-Catalyzed Processes

**Scheme 126:**
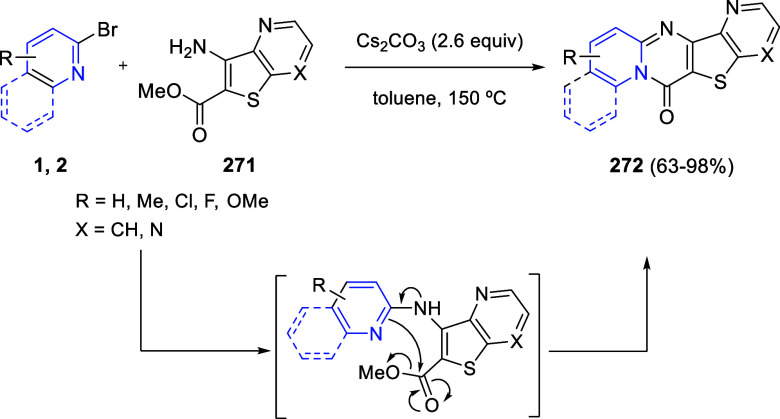
Metal-Free Synthesis of Tetracyclic Pyrido and Pyrazino Thienopyrimidinones

Trying to develop greener methodologies to access tetracyclic heterocycles based on the C–N Buchwald–Hartwig cross coupling reaction, Berteina-Raboin and co-workers found that 2-bromopyridines **1** and quinolines **2** reacted with 3-aminothieno[3,2-*b*]pyridine-2-carboxylate and 7-aminothieno[2,3-*b*]pyrazine-6-carboxylate (**271**) using only Cs_2_CO_3_ and toluene as the solvent, without a palladium catalyst (Scheme 126). The process involved nucleophilic aromatic substitution followed by dearomative cyclization.^187^

**Scheme 127:**
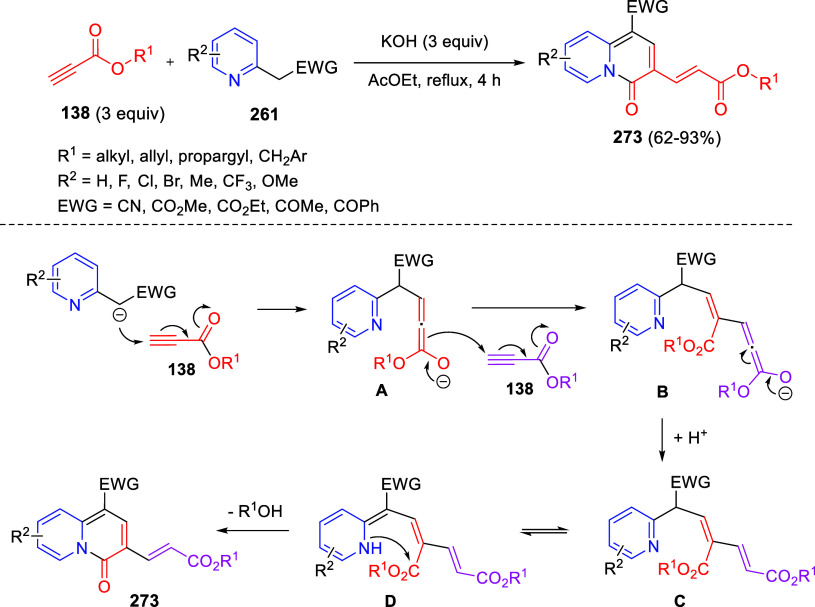
One-Pot Synthesis of 3-Substituted Quinolizinones from 2-Methylene Pyridines and Alkynyl Esters

A sequential nucleophilic addition followed by cyclization reaction between 2-methylene pyridines **261** and terminal alkynyl esters **138** was observed by Gong and Xu (Scheme 127).^188^ The reaction was performed just with KOH as a base in refluxing ethyl acetate and furnished 3-substituted 4*H*-quinolizin-4-ones **273** in good yields. Regarding the scope of the reaction, electron-withdrawing or -donating groups on the pyridine ring (R^2^), and also isoquinoline, were well tolerated, as well as several aliphatic esters (R^1^) on the alkyne substrate. In all cases, only the product with the *E* configuration at the double bond was observed.

Control experiments suggested the addition of a second alkynyl substrate to the intermediate **A**, resulting from the addition of pyridine methylene carbanion, to the alkyne (Scheme 127). After that second addition, allene intermediate **B** would be protonated and subsequently cyclized by nucleophilic addition of the pyridine to produce the final quinolizinone product **273**.

**Scheme 128:**
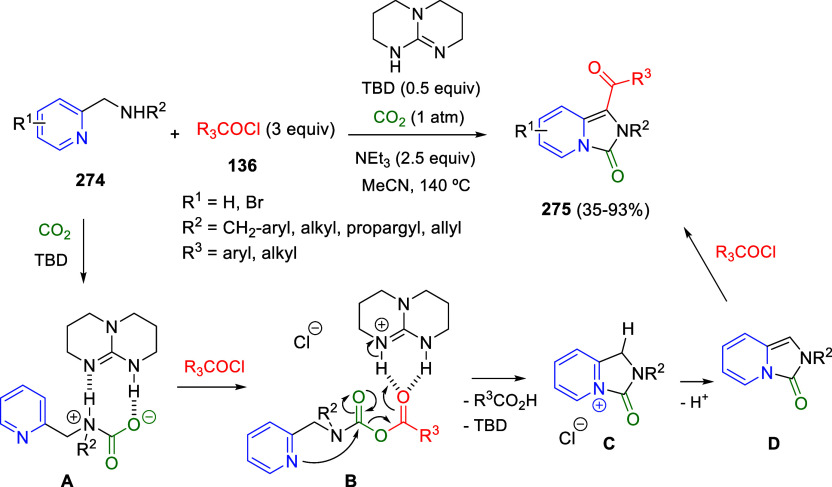
Redox-Neutral Metal-Free Three-Component Carbonylative Dearomatization of Pyridine Derivatives with CO_2_

In 2019, Bandini and Lombardo described a metal-free carbonylative dearomatization of pyridine derivatives employing CO_2_ as a nontoxic CO surrogate (Scheme 128).^189^ Specifically, a three-component reaction of pyridine-2-methanamines **274**, acyl chlorides **136**, and CO_2_ in the presence of substoichiometric amounts of 1,3,5-triazabicyclodec-5-ene (TBD) enabled the synthesis of functionalized imidazo-pyridinones **275** in good yields and excellent chemoselectivity through a redox-neutral process. Secondary benzylamines **274** (R^2^ = CH_2_-aryl) worked excellently regardless size, electronic features and substitution patterns at the benzylic site, as well as other secondary alkyl amines; however, no reaction took place with primary benzylamines (R^2^ = H).

Regarding the reaction mechanism, an unprecedented RCOCl/TBD concerted electrophilic activation of carbon dioxide was revealed by experimental and computational investigations. Initial carbonation of benzylamine **274** would be facilitated by TBD acting as a bifunctional activator, first templating the approach of benzylamine and CO_2_ to form intermediate **A**, then deprotonating it to facilitate the reaction with acid chloride and, finally, stabilizing the mixed anhydride intermediate **B** through hydrogen-bonding with the carbamate moiety (Scheme 128). Cyclization to pyridinium cation **C** and final dearomatization by deprotonation would afford intermediate **D**, which would be subjected to Friedel–Crafts-type acylation to yield the final three-component adducts **275**.

**Scheme 129:**
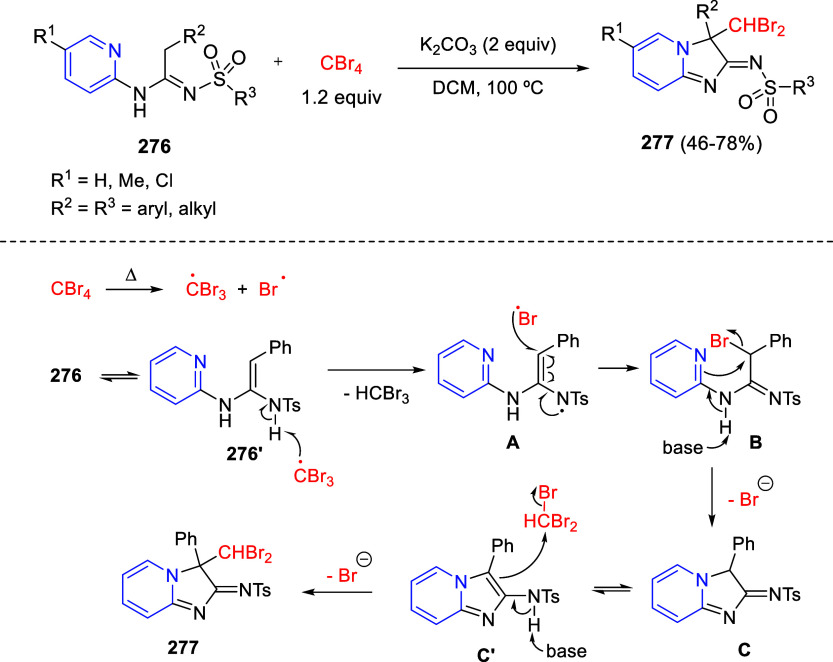
Double Cross-Dehydrogenative-Coupling Cyclization of *N*-(2-Pyridyl)amidines Mediated by CBr_4_

Yu, Yi, and co-workers developed an intramolecular double cross-dehydrogenative coupling (CDC) cyclization of *N*-(2-pyridyl)amidines **276** for the synthesis of 2-iminoimidazo[1,2-*a*]-pyridines **277** bearing a −CHBr_2_ group and an aza-quaternary carbon center at the 3-position (Scheme 129).^190^ The oxidative CDC reaction was promoted by CBr_4_, without the need of a radical initiator, in the presence of a base. Different substitution patterns were allowed on the starting pyridine derivatives **276**, which were transformed into the corresponding bicyclic products **277** in moderate to good yields.

A tentative mechanism would involve thermal homolytic cleavage of CBr_4_ and tautomerization of *N*-(2-pyridyl)amidines **276** into enamines **276′**. Then, abstraction of a hydrogen by the CBr_3_ radical would generate the *N*-centered radical intermediate **A**, which would rapidly react with a Br radical to deliver α-bromo *N*-sulfonyl imine **B** (Scheme 129). A base-promoted intramolecular nucleophilic substitution would effect the dearomative cyclization and, after tautomerization, the final product would be formed by means of a nucleophilic substitution with CHBr_3_.

**Scheme 130:**
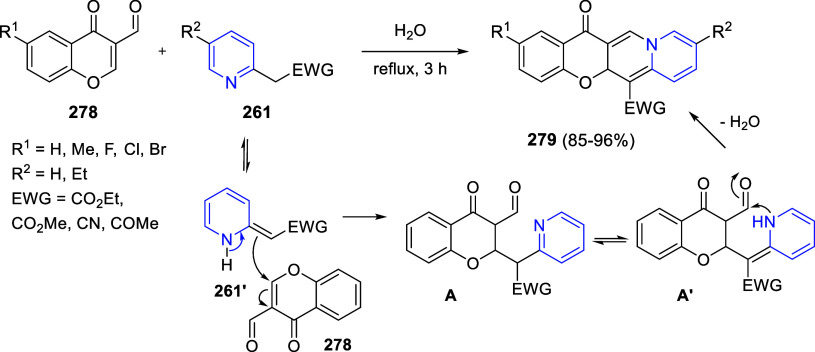
Cascade Michael Addition/Cyclocondensation Reaction of Chromone-3-carboxaldehydes with 2-Methylenepyridine Derivatives

A simple and environmentally friendly protocol to synthesize functionalized quinolizines was described by Yan and co-workers in 2020 (Scheme 130).^191^ It involved the reaction of chromone-3-carboxaldehydes **278** as bis-electrophiles and 2-methylenepyridines **261** as bis-nucleophiles by means of a sequential Michael addition/cyclocondensation reaction. This practical approach afforded products **279** in excellent yields without the need of any catalyst or base, just refluxing the mixture of reactants in water. The reaction is believed to proceed through tautomerization of 2-methylenepyridines **261** and Michael addition to chromone-3-carboxaldehydes **278** to generate intermediate **A** (Scheme 130). Subsequent tautomerization followed by cyclocondensation with the aldehyde functional group would produce final quinolizines **279**.

**Scheme 131:**
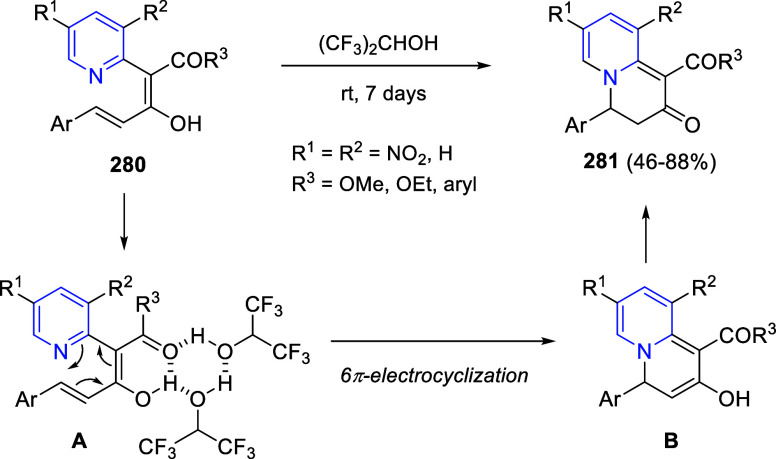
HFIP-Promoted Intramolecular Dearomative Annulation of Pyridylacetate Derivatives

The group of Yuan reported the intramolecular dearomative annulation of 2-dienyl-pyridine derivatives **280** (in turn prepared by reaction of 2-halopyridines with Nazarov reagents under basic conditions) to synthesize substituted 3,4-dihydroquinolizin-2-ones **281** in moderate to good yields (Scheme 131).^192^ The existence of the 2-pyridylacetate substrates **280** in complete enol form was a key to the success of this reaction, together with the use of hexafluoroisopropanol (HFIP) as the solvent. This dearomative annulation was compatible with substrates bearing electron-withdrawing or electron-donating groups in different positions of the aryl moiety (Ar), as well as alkoxy or benzoyl substituents in the R^3^-position.

The strong hydrogen bond donor ability of HFIP is believed to activate intermediate **A** through hydrogen bonding clusters. This would facilitate the 6π-electrocyclization reaction to form dienol intermediate **B**, and final tautomerization would afford 3,4-dihydroquinolizin-2-one products **281** (Scheme 131).

**Scheme 132:**
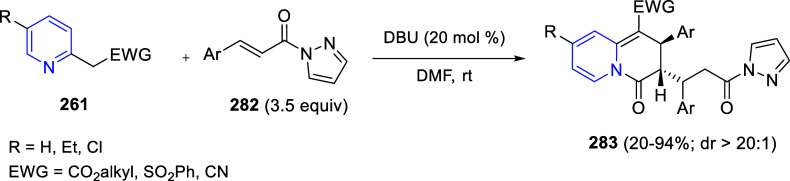
DBU-Catalyzed Dearomative Annulation of 2-Pyridylacetates with α,β-Unsaturated Pyrazolamides

The same authors developed a DBU-catalyzed dearomative annulation approach for the diastereoselective synthesis of quinolizinone derivatives (Scheme 132).^193^ Thus, electron-withdrawing group-substituted 2-methyl pyridines **261**, as 1,3-dinucleophiles, reacted with α,β-unsaturated pyrazolamides **282**, as 1,3-dielectrophiles, in a formal (3 + 3) annulation process involving two molecules of pyrazolamide. This reaction afforded multisubstituted 2,3-dihydro-4*H*-quinolizin-4-ones **283** with satisfactory yields and excellent diastereoselectivities, and it was compatible with some substituents at the 5-position of the pyridine ring and several electron-withdrawing groups, as well as different substituents on the aromatic ring of pyrazolamides. Control experiments revealed that the *N*-acylpyrazole moiety in the unsaturated pyrazolamides **282** was crucial for the DBU-catalyzed dearomative (3 + 3) annulation reaction.

**Scheme 133:**
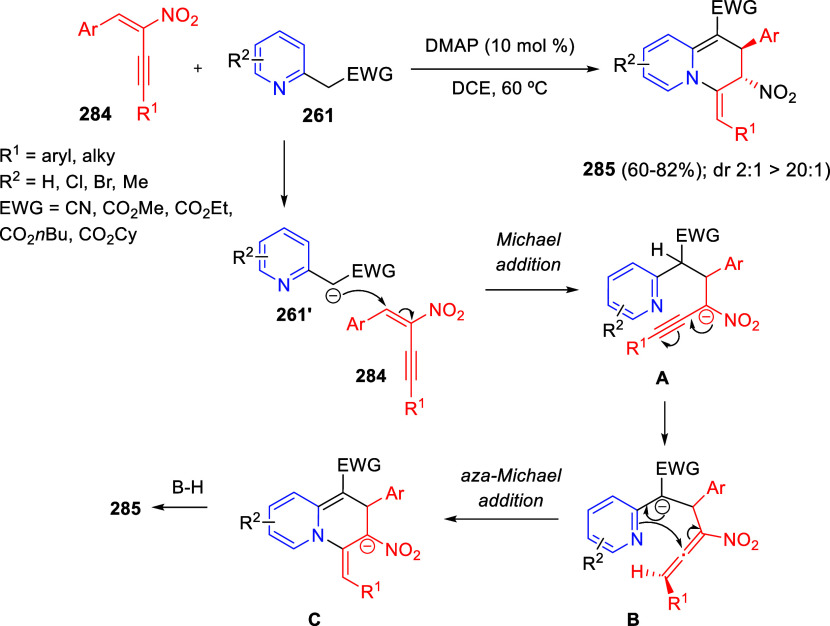
Synthesis of Functionalized Quinolizine Scaffolds via the Dearomative Annulation of 2-Pyridylacetates with Nitroenynes

Another base-catalyzed dearomative (3 + 3) annulation reaction of 2-pyridylacetates **261**, this time with nitroenynes **284** as bis-electrophiles, gave access to highly functionalized quinolizine scaffolds **285** in moderate to good yields and high diastereo- and *E*/*Z* selectivities, independently of the electron properties of substituents in the aromatic ring of nitroenynes **284** (Scheme 133).^194^ Some limitations were found regarding the substituents of the pyridine ring, since strong electron-donating or electron-withdrawing groups failed to deliver the desired products. On the other hand, replacement of the pyridine ring with quinoline or isoquinoline was well tolerated. The reaction would start with a Michael addition of deprotonated 2-pyridyl acetate **261′** to nitroenyne **284** to give intermediate **A**, which can be drawn, after proton transfer, as the allenyl intermediate **B** (Scheme 133). The subsequent dearomative cyclization would occur through an aza-Michael addition to generate allylic carbanion **C**, delivering the final products **285** with *trans* configuration upon protonation.

## 5 PHOTOCHEMICALLY-DRIVEN REACTIONS

Traditionally, metal (Birch-type) reductions represent the method of choice to perform selective dearomatization reactions mediated by radicals.^195^ However, this scenario dramatically changed with the advent of photoredox catalysis, which offers the possibility of generating radicals in a catalytic manner under mild reaction conditions, through the use of both organic and inorganic visible light-absorbing molecules [photocatalyst (PC)].^196,197^ In this context, visible-light-induced single electron transfer (SET) is the most common strategy to promote the dearomatization, but recently, triplet–triplet (EnT) energy transfer catalysis, in which the light-absorbing molecule acts as a photosensitizer (PS),^198^ has also been revealed as a very effective methodology to achieve those transformations.^199^ Furthermore, in special cases, direct visible light excitation (DE) of the substrate has been used to initiate the dearomatization reaction. Regarding the application of these photoredox methodologies, several examples of dearomatizations of pyridines, quinolines, and isoquinolines have recently been reported.

### 5.1 Reactions Promoted via Single Electron Transfer (SET)

**Scheme 134:**
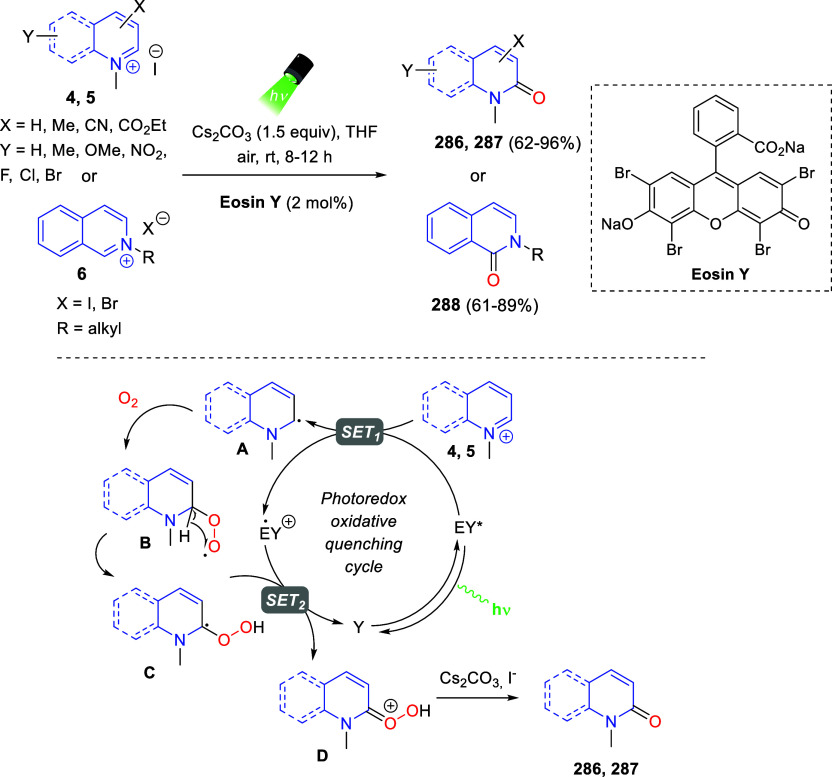
Visible-Light-Mediated Aerobic Oxidation of *N*-Alkylpyridinium Salts under Organic Photocatalysis with Eosin Y

The first dearomatization of *N*-heteroarenes using photoredox catalysis since 2016 was reported by Fu and co-workers (Scheme 134).^200^ They developed an aerobic oxidation of pyridine, quinoline and isoquinoline salts employing Eosin Y (EY) as a photocatalyst, cesium carbonate as a base, and air as a final oxidant to generate *N*-alkyl pyridones **286**, quinolones **287**, and isoquinolones **288** in very good yields and with high tolerance to functional groups. Mechanistically, this process would involve two SET’s in an oxidative quenching cycle to achieve the target products. First, excited eosin Y (EY*) would react, via SET_1_, with the *N*-heterocyclic salt, providing the corresponding radicals **A** (Scheme 134). Then, the heterocyclic radical would react with oxygen to form the dioxyl radical **B**, which would produce the carbon radical **C** through intramolecular migration of hydrogen. Finally, the photocatalyst would be regenerated by SET_2_ from the carbon radical to EY^**+**^, affording the hydroperoxyl heterocycle **D**, which would be transformed into the final products by treatment with Cs_2_CO_3_ and I^–^.

**Scheme 135:**
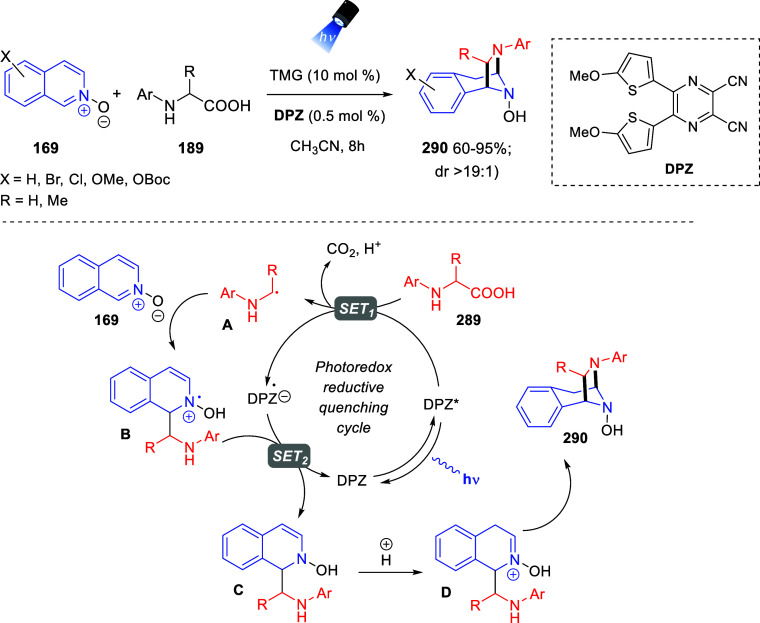
Photoredox-Catalyzed formal (3 + 2) Cycloaddition of *N*-Aryl α-Amino Acids with Isoquinoline *N*-Oxides

In 2019, the group of Jiang described a dearomatization reaction of isoquinoline *N*-oxides using an organic dye as a photocatalyst but in this case, a reductive quenching cycle was implemented (Scheme 135).^201^ It represented a formal (3 + 2) cycloaddition between substituted isoquinoline *N*-oxides **169** and *N*-aryl α-amino acids **289** in the presence of 1,1,3,3-tetramethylguanidine (TMG) as a proton shutter and dicyanopyrazine-derived chromophore (DPZ) as a photocatalyst. Initially, excited DPZ (DPZ*) would generate the α-amino radical **A** via SET_1_. Then, it would add to the isoquinoline *N*-oxide to form intermediate **B**, which would be reduced by DPZ^–^ via SET_2_ to intermediate **C** (Scheme 135). Finally, the reduced intermediate **C** would evolve by another protonation to iminium ion **D** which, after an intramolecular Mannich-type reaction, would give rise to the corresponding diazabicyclo-[3.2.1]octane-based *N*-heterocyclic compounds **290** in high yields and almost complete diastereoselectivity. Unfortunalety, this methodology was not suitable when amino acids with benzyl and phenyl substituents were used as starting materials.

**Scheme 136:**
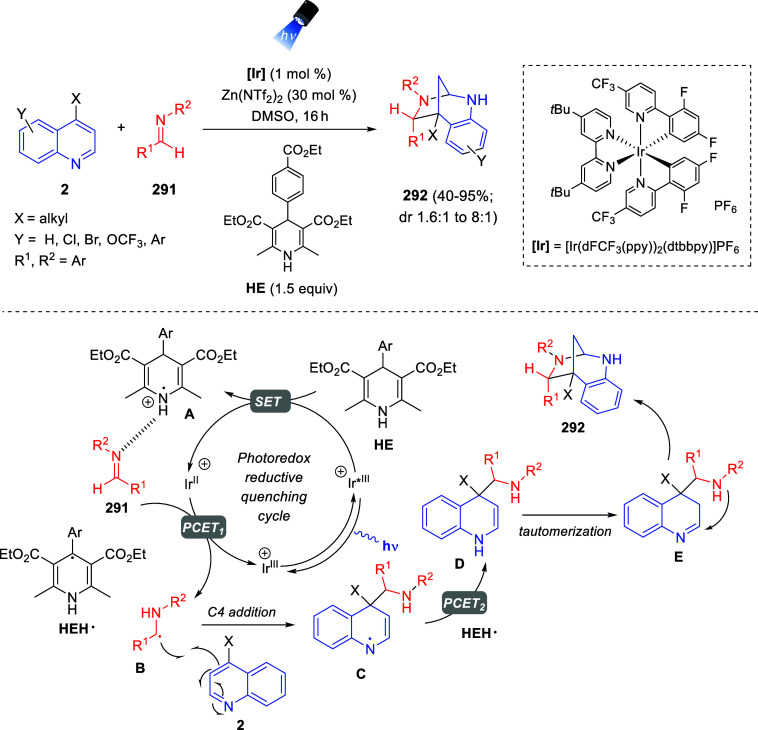
Ir-Mediated Dearomative Photocatalytic Synthesis of Bridged 1,3-Diazepanes

One year later, Dixon and co-workers reported the synthesis of bridged 1,3-diazepanes employing a photocatalytic reductive diversion of the Minisci reaction (Scheme 136).^202^ The authors used catalytic amounts of [Ir(dFCF_3_(ppy))_2_(dtbbpy)]PF_6_ in combination with a stoichiometric reductant derived from Hantzsch ester (HE) to promote the coupling of substituted *N*-arylimines **291** with quinolines **2** under blue light irradiation. The reaction provided access to 2,7-diazabicyclo[3.2.1]octanes **292** in good yields and modest diastereoselectivities, which improved with increasing steric demand at the C7-position of the quinoline ring. Regarding the mechanism, a photocatalytic single electron transfer (SET) would occur between excited [Ir(dFCF_3_(ppy))_2_(dtbbpy)]PF_6_ and the Hantzsch ester derivative to initiate the process. Then, the partially oxidized Hantzsch ester radical cation **A** would facilitate proton-coupled electron transfer (PCET_1_) reduction to obtain the nucleophilic α-amino radical **B**, which would undergo a regioselective C4 addition to the quinoline (Scheme 136). After that, the Hantzsch ester radical intermediate (HEH**·**), would assist a concerted PCET_2_ to afford benzofused dihydropyridine **D**, which would evolve to 1,3-diazepane **292** after tautomerization/intramolecular Mannich-type addition sequence.

**Scheme 137:**
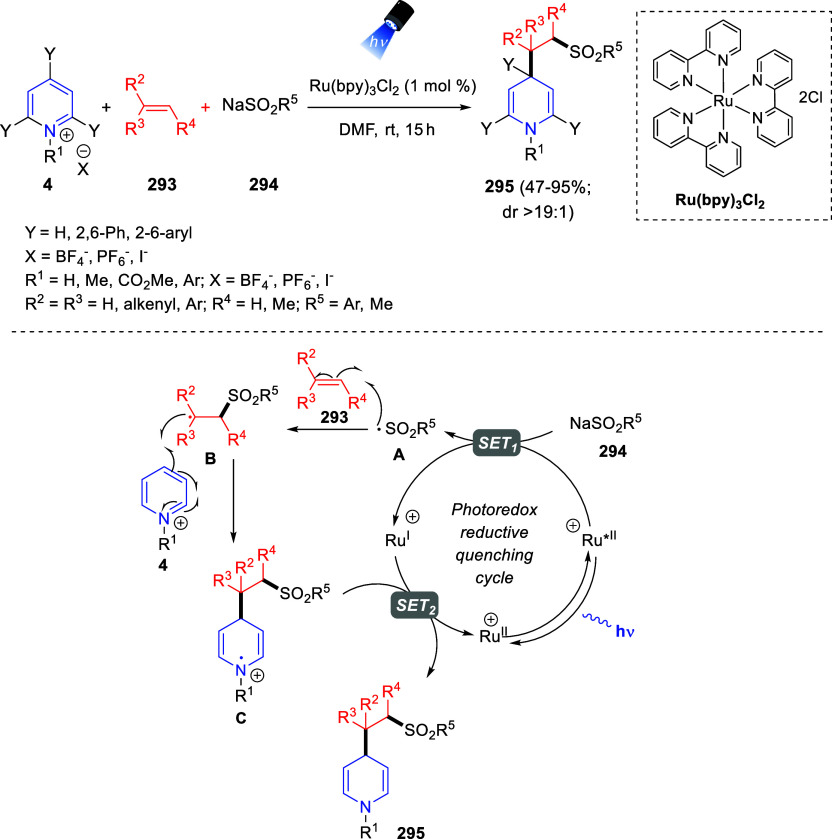
Dearomatization-Enabled Visible-Light-Induced 1,2-Alkylsulfonylation of Alkenes with Sodium Sulfinates and Pyridinium Salts

The group of Li reported a three-component alkylsulfonylation of alkenes **293** using sodium sulfonates **294** and pyridinium salts **4** through visible-light photoredox catalysis to achieve the synthesis of 2,4-dihydropyridines derivatives **295** in generally good yields with excellent stereoselectivity (Scheme 137).^203^ In this work, the excited [Ru(bpy)_3_]^2+^* would undergo a reductive single electron transfer (SET_1_) with sodium sulfonate **294** to form the sulfonyl radical **A**, which would undergo a Giese-type addition with the alkene **293**, generating the most stable ethylsulfonyl radical **B** (Scheme 137). This radical would be added to the 4-position of the pyridinium ring, forming the radical cation intermediate **C**, which would evolve into the dihydropyridine **295** through SET_2_, also regenerating the active species of the catalyst. It is important to note that the use of 2,6-disubstituted pyridinium salts and the introduction of electron-withdrawing groups at the 4-position of these substrates was the key to direct the addition to the 4-position. Furthermore, the scope was later extended to quinolinium salts, which underwent the addition to the 2-position of the heterocycle ring, although in significantly lower yields.

**Scheme 138:**
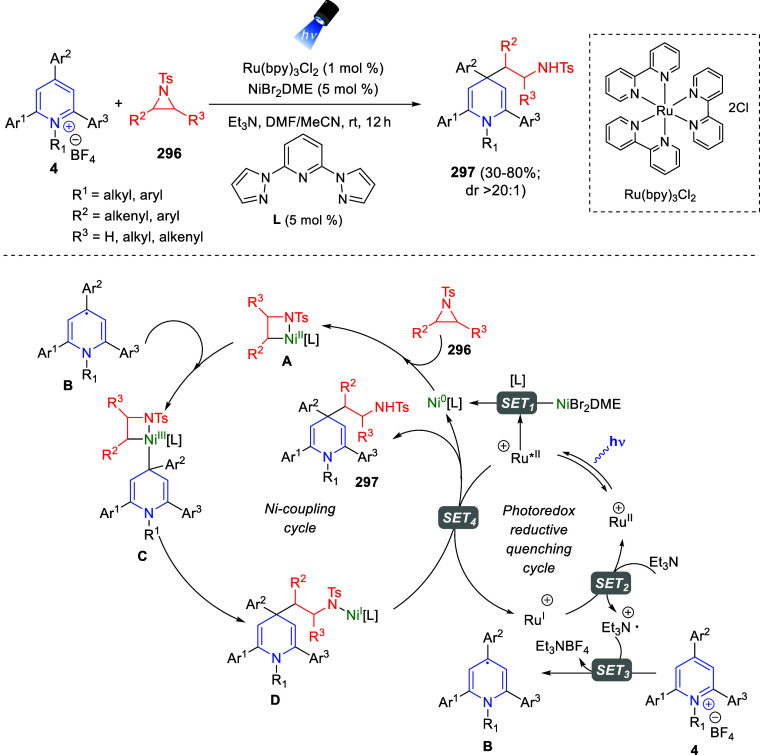
Cooperative Photoredox/Nickel Catalysis for Cross-Electrophile Coupling of Aziridines with Pyridinium Salts

The same group continued exploring the dearomatization of pyridinium salts, this time taking advantage of the cooperative combination of Ru-photoredox catalysis and Ni cross coupling catalysis (Scheme 138).^204^ This dual catalytic system allowed the coupling of pyridinium salts **4** and different electrophilic *N*-tosylaziridines **296**, with the subsequent dearomatization of the pyridine ring. This protocol provided β-(1,4-dihydropyridin-4-yl)-ethylamines **297**, in generally good yields, and tolerated differently substituted aryl and vinyl aziridines, with excellent stereocontrol when these were disubstituted. Additionally, different bioactive building blocks analogues were introduced at the R^1^-position without loss in chemical yield.

Regarding a possible mechanism for this cross-electrophile coupling protocol, the authors proposed that coordination of ligand **L** with the NiBr_2_·DME complex would produce the active Ni^0^[L] species via SET_1_, which would undergo the regioselective oxidative addition of the aziridine **296** to give Ni-azetidine intermediate **A** (Scheme 138). This would react with the 1,4-dihydropyridin-4-yl radical **B**, generated via SET_3_ from pyridinium salt **4** and the radical cation of Et_3_N, to afford the Ni^III^ intermediate **C**. Subsequently, reductive elimination would take place on this intermediate, forming the Ni^I^ coordinated ethylamine **D**. Finally, the Ru^I^ species would assist the reductive SET_4_ of the Ni^I^ intermediate **D**, affording the final product **304** and regenerating the Ni^0^ catalyst and the Ru^II^ photocatalyst.

**Scheme 139:**
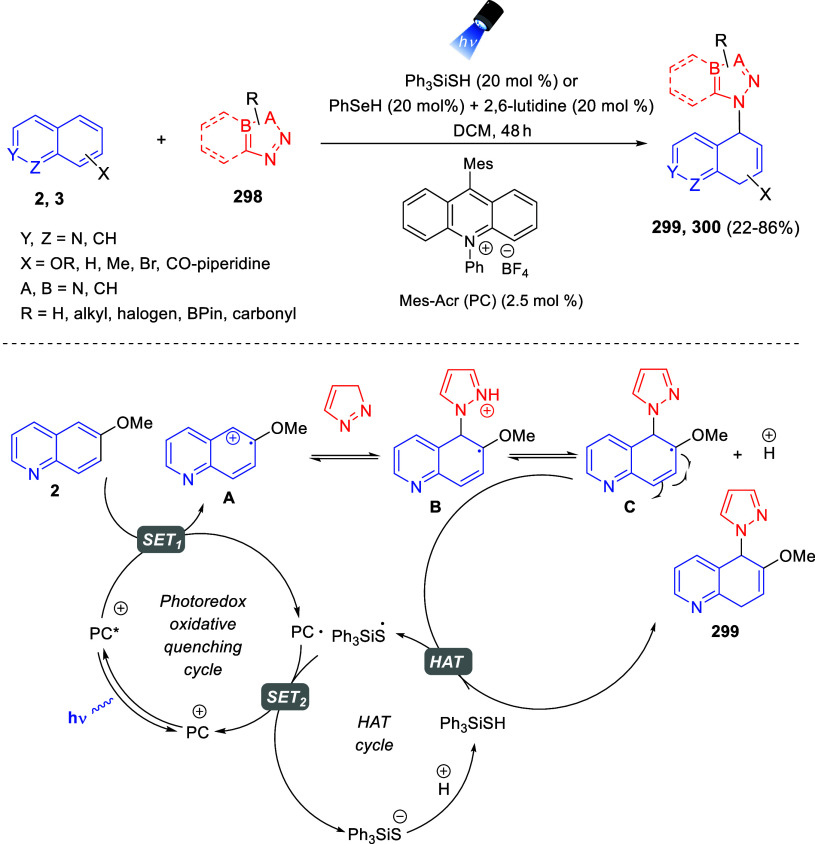
Organophotoredox Approach for the Dearomatization of Quinolones and Isoquinolines with Azoles

Very recently, the groups of Houk, Chen, and Wang developed an elegant organophotoredox approach for the chemo- and regioselective dearomatization of different benzofused arenes, including quinoline and isoquinoline (Scheme 139).^205^ The nucleophilic addition of different azoles **298** to the C5/C4-position of quinolines **2** and isoquinolines **3** using *N*-phenylmeso-acridinium tetrafluoroborate (Mes-Acr) as a photocatalyst and Ph_3_SiSH or PhSeH as hydrogen atom transfer (HAT) agents led to a wide range of dihydroquinolines **299** and dihydroisoquinolines **300**. Due to the mild conditions employed, this protocol was applicable to late-stage functionalizations of pharmaceutically relevant quinoline derivatives.

The authors studied the reaction mechanism in detail using 6-methoxyquinoline and pyrazole as model substrates. The process would start with the oxidation of quinoline with the excited state of Mes-Acr (PC^+^*) via SET_1_ reaction. Then, pyrazole would add to the 5-position of the quinoline radical cation **A** (most reactive position according to computational calculations) to form intermediate **B**, which would evolve into the neutral radical **C** by deprotonation (Scheme 139). Finally, Ph_3_SiH would promote the HAT reaction to produce the final 5-substituted dihydroquinoline **299** and Ph_3_Si radical, which regenerates the photocatalyst via SET_2_. It is important to highlight that the introduction of electron-donating groups in the quinoline and isoquinoline rings was key for the reaction to work, since they decrease the oxidation potential of the heterocycles, which allows them to be oxidized with the photocatalyst. For this reason, although the reaction tolerated a wide range of groups on the azole counterpart, it did not work with electron-withdrawing groups on the quinoline and isoquinoline rings.

### 5.2 Reactions Promoted via Triplet–Triplet Energy Transfer (EnT)

**Scheme 140:**
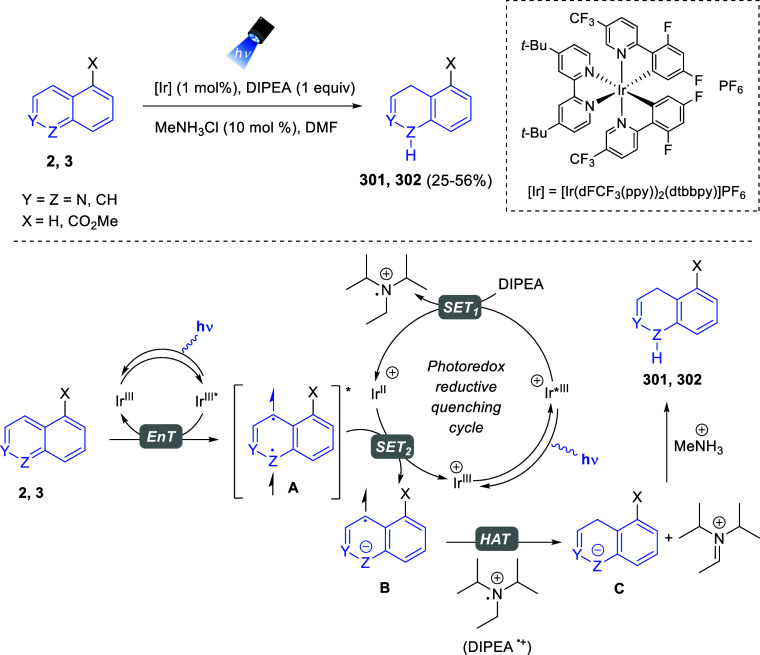
Direct Reduction of Quinolines and Isoquinolines Using Visible-Light Photoredox Catalysis

The first dearomative reduction of quinolines and isoquinolines promoted by triplet–triplet energy transfer was reported by Köning in 2019.^206^ They combined [Ir(dFCF_3_(ppy))_2_(dtbbpy)]PF_6_, as a metal-based photocatalyst, with stochiometric DIPEA, as the sacrificial electron donor, to achieve the synthesis of tetrahydroquinolines **301** tetrahydroisoquinolines **302** in moderate yields, using MeNH_3_Cl as the proton source (Scheme 140).

Specifically, the [Ir(dFCF_3_(ppy))_2_(dtbbpy)]PF_6_ complex played a dual role in the process. On the one hand, it would act as a photosensitizer, promoting the formation of the excited triplet state **A** of the corresponding heterocycle via EnT upon visible-light photoexcitation. On the other hand, upon reduction with DIPEA thought SET_1_, the resulting Ir^II^ species would reductively quench the excited triple state **A** via SET_2_, generating the radical anion of the heterocycle (**B**), which would evolve to the corresponding carbanion **C** after extracting a hydrogen atom from the DIPEA^**·+**^ (HAT). Finally, protonation from MeNH_3_Cl would give rise to the final reduced *N*-heterocycles (Scheme 140).

**Scheme 141:**
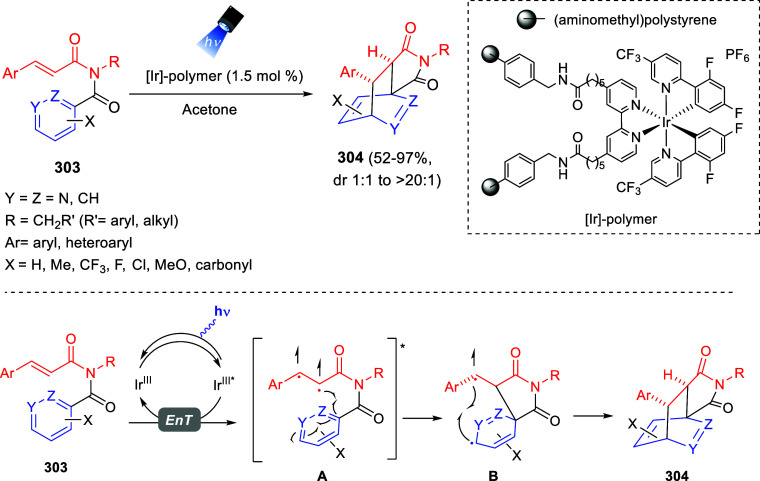
Dearomative (4 + 2) Cycloaddition Reaction of Pyridines Mediated by a Polymer Immobilized Ir-Based Photocatalyst

Almost at the same time, Glorius and co-workers described a visible-light energy-transfer-catalyzed intramolecular dearomative (4 + 2) cycloaddition of pyridines (Scheme 141).^207^ Interestingly, this energy transfer process was enabled by an iridium-based photocatalyst immobilized on a polymer of (aminomethyl)polystyrene through anchoring carboxylic acid chains to the pyridine ligands of the iridium complex. Once the polymer-supported photocatalyst was synthesized, it was used to promote the intramolecular cycloaddition of pyridine-containing cinnamyl amides **303** under blue LEDs irradiation in acetone as the solvent. The reaction worked successfully and provided numerous functionalized isoquinuclidine derivatives **304** in high yields and excellent functional group tolerance. In fact, both electron-rich and electron-deficient substituents at the heteroarene- and R-positions gave rise to the desired products without much difference in yield. Furthermore, to demonstrate the suitability of the process, it was shown that the catalyst could be recycled up to ten times on gram-scale reactions without any loss in chemical yield.

Computational and experimental investigations supported a mechanism that would start by triplet–triplet energy transfer (EnT) from the visible-light-excited iridium complex to the cinnamyl moiety of the *N*-heterocycle, producing the 1,2-biradical intermediate **A** (Scheme 141). This would undergo a regioselective dearomative intramolecular (4 + 2) cycloaddition, leading to the isoquinuclidine pruducts. The process was further extended to quinolines and isoquinolines with comparable yields and increased diastereoselectivities.

**Scheme 142:**
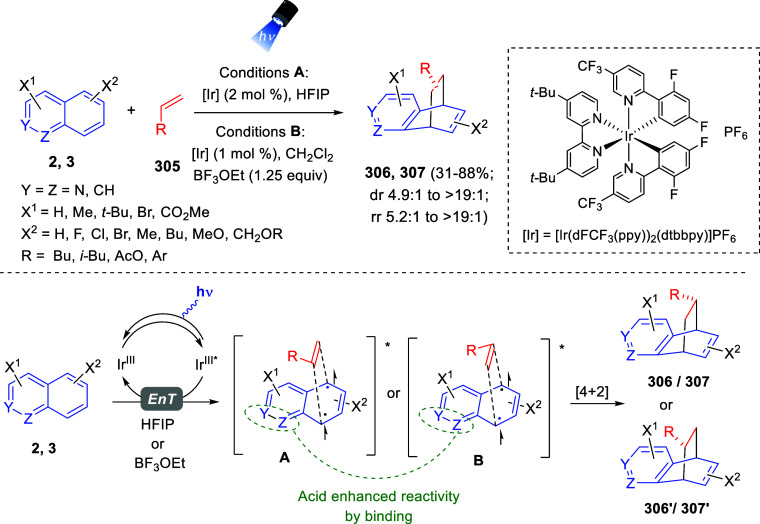
Photochemical Intermolecular Dearomative (4 + 2) Cycloaddition Reaction of (Iso)quinolines with Alkenes

In 2021, the groups of Glorius, Brown, and Houk studied an intermolecular variant of this photochemical dearomative (4 + 2) cycloaddition of quinolines **2** and isoquinolines **3** with alkenes **305** in the presence of a Brønsted [hexafluoroisopropanol (HFIP)] or Lewis acid (BF_3_OEt_2_) mediator and [Ir(dFCF_3_(ppy))_2_(dtbbpy)]PF_6_ as the photosensitizer (Scheme 142).^208^ A wide variety of bridged regioisomeric polycycles **306**/**306′** and **307**/**307′** from quinolines and isoquinolines, respectively, were obtained. Specifically, the photochemically excited sensitizer would transfer energy to the (iso)quinoline, generating a highly reactive biradical intermediate, which would undergo the carbocyclic (4 + 2) cycloaddition with the alkene through transition state **A** or **B** to afford the target products (Scheme 142). It was assumed that HFIP or BF_3_·OEt_2_ would bind to the heterocyclic substrate through hydrogen bonding or Lewis acid–base interaction, respectively, decreasing triplet energy and rendering the resulting adducts more amenable to EnT by the photosensitizer.

The protocol proceeded with good *endo*-diastereocontrol and the regioselectivity was also satisfactory, although it was highly dependent on the substitution patterns in the heterocyclic rings. Curiously, formation of regioisomers **306**/**307** was predominant in the 2-, 3-, 4-, 5-, or 7-substituted quinolines, whereas products **306′**/**307′** were the major ones in the 6- or 8-substituted quinolines. Furthermore, a broad scope of activated and unactivated alkenes **305** was well tolerated without erosion of diastereo- and regioselectivity. It is also important to note that the reaction was carried out on a gram scale without any loss of yield and selectivity, and that many of the products were also derivatized to obtain very interesting molecules in the field of pharmaceutical chemistry.

Subsequent mechanistic studies showed that both Lewis and Brønsted acids enhanced the reactivity of the triplet state of the *N*-heterocycles toward alkenes, but did not facilitate energy transfer (EnT).^209^

**Scheme 143:**
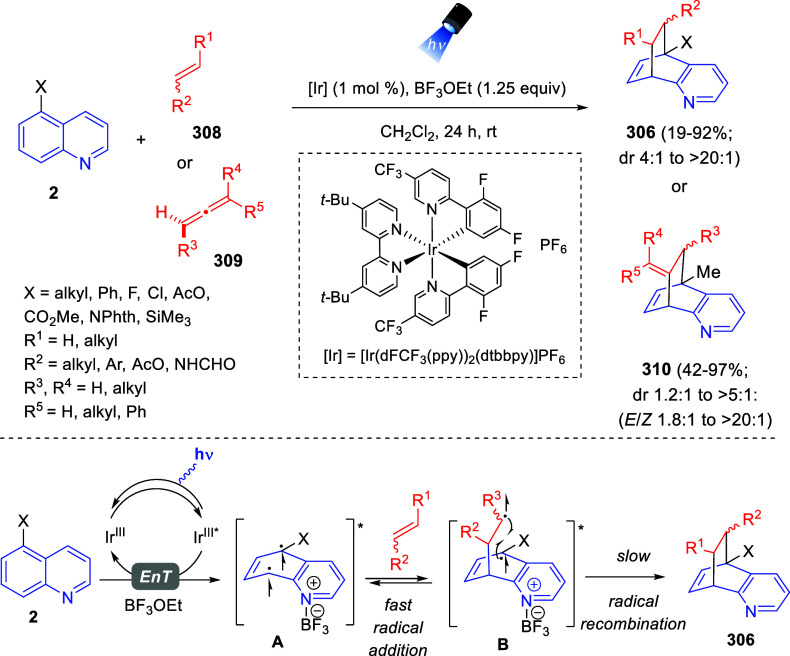
Photochemical Dearomative Cycloadditions of Quinolines and Alkenes or Allenes

Afterward, the same authors extended the scope of the photochemical dearomative cycloaddition of quinolines to disubstituted alkenes **308** and allenes **309** under the optimized Lewis acid-mediated conditions (Scheme 143).^210^ To ensure complete regioselectivity, the reaction was studied employing 5-substituted quinolines **2** as starting substrates, leading to products **306** and **310** containing a sterically congested quaternary carbon. The protocol was very efficient and diastereoselective with all types of quinoline substituents, regardless of their electronic properties and steric hindrance, except when a 4-pentene moiety was introduced, which decreased the yield dramatically. In addition, a wide range of monosubstituted, disubstituted, activated and inactivated alkenes were well tolerated, although monosubstituted alkenes favored the formation of *cis* products while disubstituted alkenes gave rise to the *trans* heterocycles mainly. The process also worked with different allenes; however, the results were worse in terms of diastereoselectivity and mixtures of *E*/*Z* isomers were also obtained when starting from 1,3-disubstituted allenes.

Concerning the reaction mechanism, the authors demonstrated that the cycloaddition was not concerted as proposed previously, so it would comprise two steps after the energy transfer from the photosensitizer. The first step would be a reversible intermolecular radical addition between the heterocyclic biradical **A** and the alkene and it would occur rapidly. After that, the second biradical intermediate **B** would be slowly transformed into the target product **313** by an irreversible intramolecular radical recombination, this being the step that would determine the selectivity of the reaction, as the long-lived biradical intermediate allows for bond rotation before cyclization (Scheme 143).

**Scheme 144:**
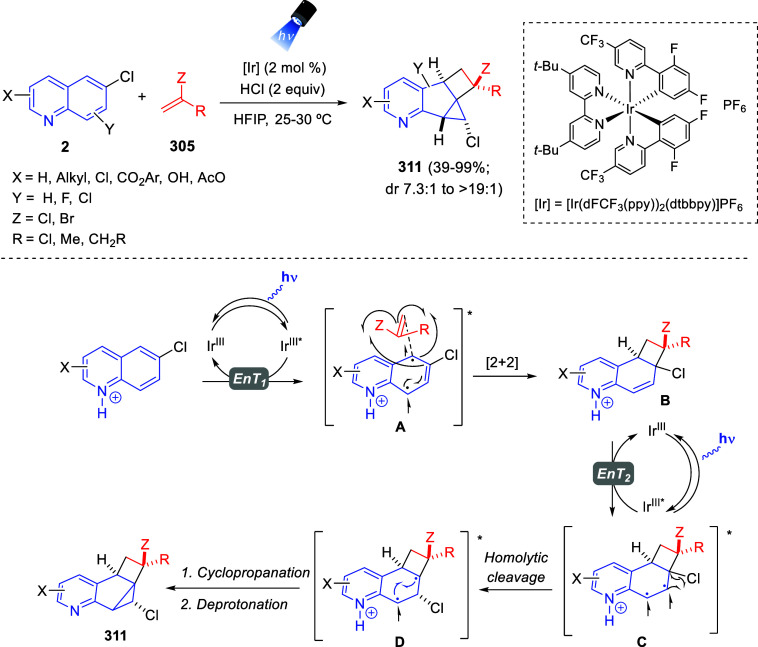
Photocatalytic Cascade Dearomative (2 + 2) Cycloaddition/Cyclopropanation Reaction of Quinolines with Alkenes

Glorius and co-workers continued exploring the reactivity of quinolines with alkenes under photoredox catalysis and they developed a cascade dearomative (2 + 2) cycloaddition/cyclopropanation reaction. For this study, the authors employed 6-chloroquinoline derivatives **2** and disubstituted halogenated alkenes **305** in the presence of the Ir-based photosensitizer and HCl and using hexafluoroisopropanol (HFIP) as the solvent, under blue LEDs irradiation (Scheme 144).^211^ In this manner, a family of highly functionalized pyridine-fused 6-5-4-3-membered ring systems **311** was obtained in good yields and diastereoselectivity. The process would begin in a similar way than that described in the pioneering work.^208^ However, after the energy transfer (EnT_1_) from the photosensitizer to the quinolone ring, the resulting biradical species **A** would react by means of a dearomative (2 + 2) cycloaddition with the olefin, generating intermediate **B**. Then, a second energy transfer (EnT_2_) event would occur, producing another biradical **C**, which would undergo homolytic C–Cl cleavage to intermediate **D**, followed by C–C and C–Cl bond formations to furnish the final tetracyclic products **311** bearing two adjacent quaternary stereocenters with excellent diastereoselectivity (Scheme 144). Importantly, this transformation presented broad functional group tolerance and it was applied to gram-scale reactions. In addition, several derivatizations of the final products were performed, including the synthesis of an advanced intermediate of chemokine receptor antagonists.

**Scheme 145:**
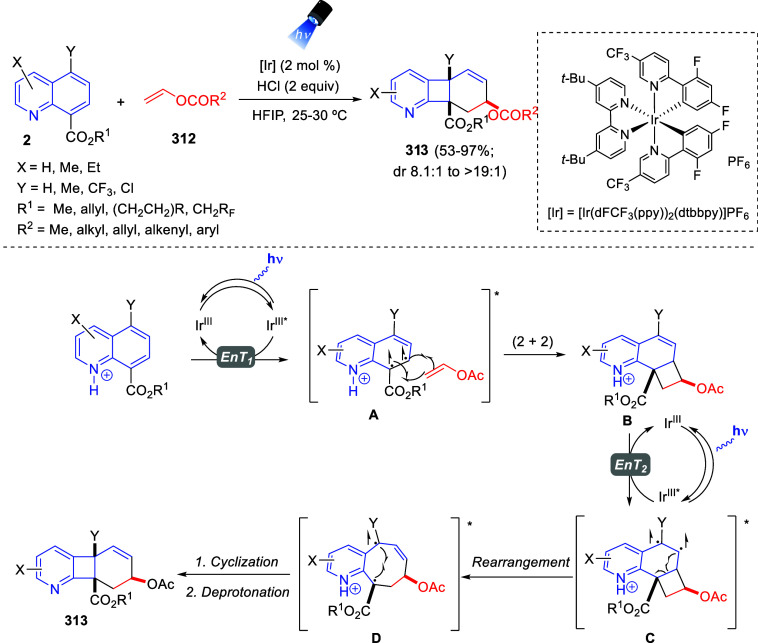
Photocatalytic Cascade Dearomative (2 + 2) Cycloaddition/Cyclobutane Rearrangement of Quinolines with Alkenes

In view of the mechanism proposed for this transformation, the authors designed another cascade dearomatization process, employing 5-substituted quinoline-8-carboxylates **2** and vinyl acetates **312** as a starting materials, under the same reaction conditions (Scheme 145).^208^ Due to the change in the substitution pattern of the heterocyclic ring, it was possible to direct the dearomative (2 + 2) cycloaddition at the 7- and 8-positions of the quinoline ring, leading to vinylcyclobutane **B**. A second energy transfer (EnT_2_) event would trigger a ring rearrangement and subsequent ring closure, giving rise to 6-4-6-membered fused tricycles **313** in high yields and exceptional *syn*-diastereocontrol at the two adjacent quaternary centers (Scheme 145).

It is important to highlight that these examples constitute an original methodology to generate structural complexity from simple substrates. They entailed, for the first time, two consecutive energy transfers to promote cascade dearomatizations that furnished complex pyridine-fused ring systems with high diastereoselecivity. Furthermore, the demonstration that tailor-made substitution at the quinoline framework can divert its reactivity may lead to new interesting transformations in the future.

### 5.3 Reactions Promoted by Direct Visible-Light Excitation (DE)

**Scheme 146:**
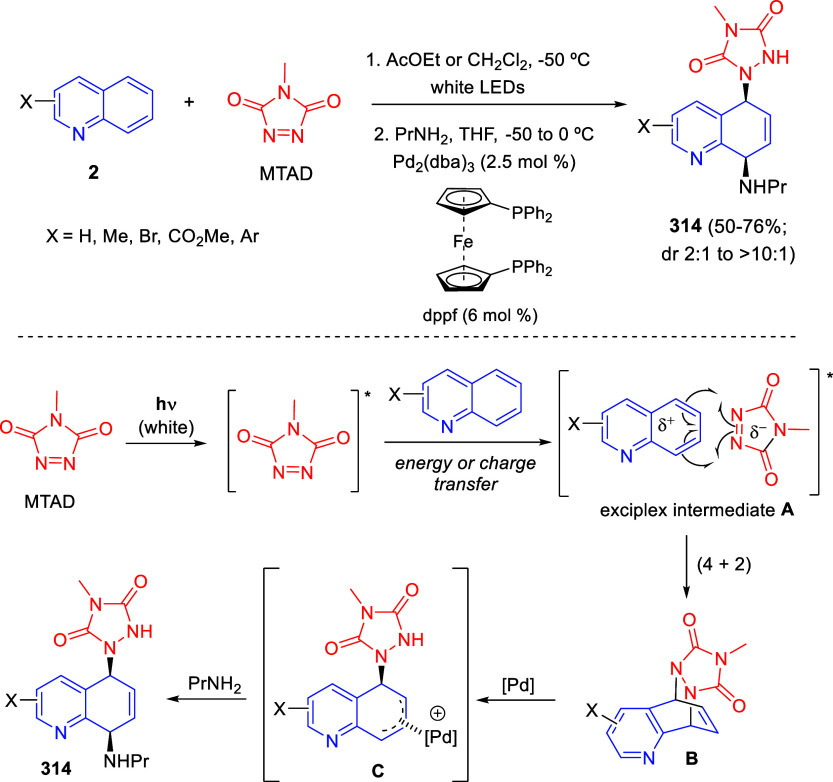
Catalytic Dearomative *syn*-1,4-Diamination via Visible-Light-Mediated (4 + 2) Cycloaddition/Pd-Catalyzed Allylic Amination of Quinolines

The direct application of visible light on substrates to promote dearomatization processes has remained poorly developed. This is because organic molecules normally require high-energy ultraviolet light to access their highly reactive excited states.^208^ In this context, the first dearomatization of quinolines in the absence of any photoredox catalyst or sensitizer was developed by the group of Sarlah in 2019 (Scheme 146).^212^ In this work, a one-pot visible-light-mediated (4 + 2) cycloaddition/Pd-catalyzed allylic amination sequence was optimized to obtain *syn*-5,8-diamino dihydroquinolines **314** with high efficiency. It involved the exposure of quinoline **2** and *N*-methyl-1,2,4-triazoline-3,5-dione (MTAD) to white light irradiation at low temperature, followed by addition of an amine and a Pd catalyst. The process would start with the formation of an exciplex intermediate **A** (excited complex) between excited MTAD and quinoline. This complex would undergo intramolecular (4 + 2) cycloaddition, generating the strained *para*-cycloadduct **B** (Scheme 146). Then, palladium would catalyze the ring-opening reaction of the cycloadduct through oxidative addition, giving rise to the corresponding organometallic intermediate **C**, which would be captured by the amine. This protocol was later extended to other carbon^213^ and oxygen^214^ nucleophiles with different arenes.

**Scheme 147:**
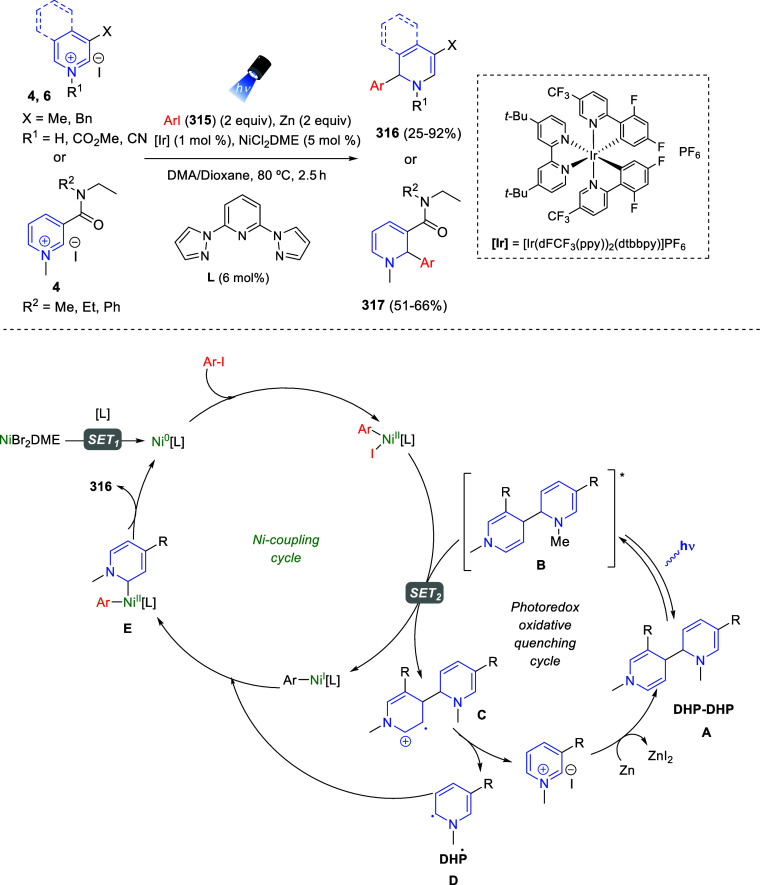
Light-Promoted Dearomative Cross-Coupling of Heteroarenium Salts and Aryl Iodides via Nickel Catalysis

Finally, in 2022, Karimov and co-workers developed a dearomatization process of pyridinium and isoquinolinium salts using direct visible-light excitation (Scheme 147).^215^ The authors took advantage of the nickel (Ni/bpp)/photoredox dual catalysis to achieve the coupling between 3-substituted pyridinium salts **4** and 4-substituted isoquinolinium salts **6** and different aryl iodides **315** in the presence of Zn as a terminal reductant. The process would take place in two independent catalytic cycles. On the one hand, the coordination of ligand **L** with NiCl_2_DME via SET_1_ would produce the active Ni^0^[L] species, which would undergo oxidative addition of the aryl iodide. On the other hand, Zn would promote the dimerization of the heteroarenium salt to form the dihydroheteroaryl dimer **A**, which would be capable of being excited under blue light irradiation to species **B**. Then, a SET_2_ between the excited dimer and the Ni(II) aryl iodide complex would generate the aryl Ni^I^ complex and the cation radical dimer **C**, which would decompose into dihydroheteroaryl radical **D** and pyridinium iodide. After that, the radical would recombine with the Ni^I^ complex to form the dihydroheteroaryl Ni^II^ complex **E** and the pyridinium iodide would be recycled to generate a new dimer. Finally, complex **E** would undergo reductive elimination, giving the final product **316** and regenerating the Ni^0^[L] catalyst (Scheme 147).

Dihydropyridines **317** and dihydroisoquinolines **316** were obtained in high yields with excellent functional group tolerance, although electron-withdrawing group-bearing aryl iodides gave slightly lower yields. Furthermore, the authors were able to direct the coupling at different positions on the heterocyclic ring. Thus, when using C3 amide-substituted pyridiniums, the reaction led to coupling at position 2, whereas when using isoquinoliniums or C3 cyano-/ester-substituted pyridiniums, the reaction produced the C6-addition products. This difference in regioselectivity was probably due to the ability of the amide group to coordinate with the nickel catalyst and direct the coupling. The role of the iridium catalyst in the reaction was not clear, since it could act as a sensitizer helping the excitation of the dimer or only assisting SET_1_ to produce the active nickel species.

## 6 ADDITION OF OTHER NUCLEOPHILES

In the present section of this review, dearomatization processes of pyridines, quinolines, and isoquinolines via intermolecular reactions involving the addition of carbon- and heteroatom-centered nucleophiles to heteroarenes or their salts will be covered. Additionally, dearomatizations initiated by nucleophilic additions of the heterocyclic nitrogen (especially those containing electron-donating groups such as hydroxyl or amine groups) to electrophiles will also be included in this section. Tandem protocols initiated by hydrogen transfer reactions and subsequent *N*-addition to electrophiles, which usually proceed with dearomatization/functionalization of the heterocycle, will be also addressed in this section.

For a better understanding, these dearomatizations of heteroarenes will be classified in metal-promoted and metal-free protocols, the last in turn distinguished between organocatalytic and base-mediated reactions.

### 6.1 Metal-Catalyzed Reactions

#### 6.1.1 Copper-Based Protocols

**Scheme 148:**
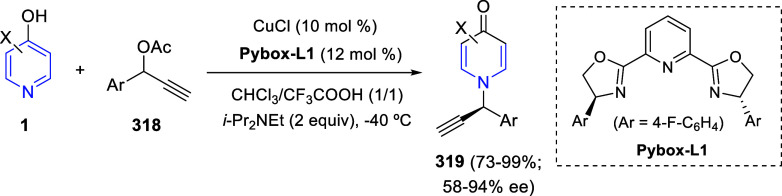
Synthesis of *N*-Alkylated 4-Pyridones by Copper-Catalyzed Intermolecular Asymmetric Propargylic Amination

You and co-workers reported a highly regio- and enantioselective copper-catalyzed intermolecular propargylation of 4-hydroxypyridines **1** (Scheme 148).^216^ Their reaction with propargylic acetates **318**, in the presence of a copper complex derived from chiral Pybox ligand and diisopropylamine as a base, gave access to *N*-propargylated-4-pyridones **319** in excellent yields and high levels of enantiocontrol. A wide variety of aryl and heteroaryl substituents on the propargylic acetates were compatible with the process, while aliphatic substituents did not provide the final products. Regarding the hydroxypyridine counterpart, a variety of substituents at the C3-position were well tolerated, whereas substitution at C2 resulted in a clear decrease in enantioselectivity. This transformation could be carried out on a gram-scale, and the products could be transformed into the core structure of quinolizidine alkaloids.

**Scheme 149:**
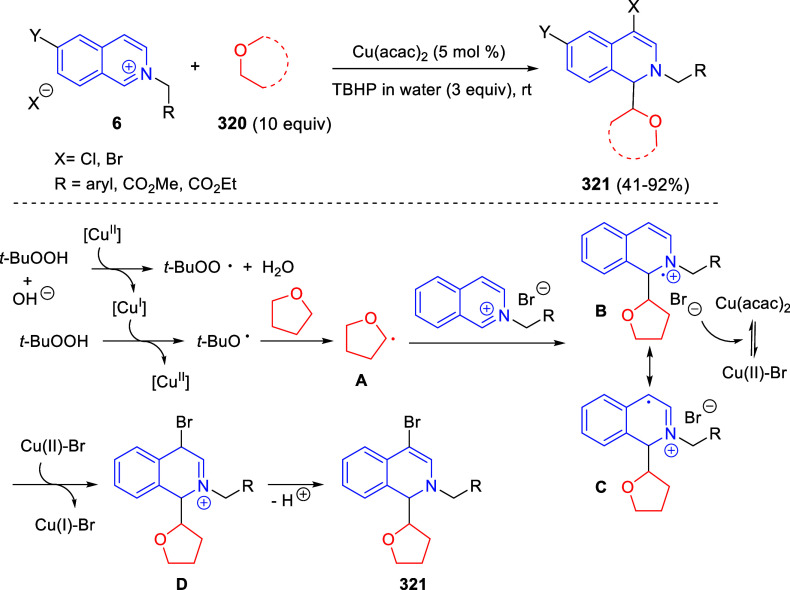
Cu-Catalyzed 1,4-Difunctionalization of Isoquinolinium Salts through an Atom-Transfer Radical Process

In 2018, a copper-catalyzed solvent-free 1,4-difunctionalization of isoquinolinium salts under mild conditions was described by Yan and co-workers (Scheme 149).^217^ The reaction of cyclic ethers and acetals **320** with isoquinolinium chlorides and bromides **6** in the presence of catalytic amounts of Cu(acac)_2_ and a water solution of *t*-butyl hydroperoxide (TBHP) rendered 1,4-difunctionalized dihydroisoquinolines **321** in good yields, with incorporation of the counterion of the starting isoquinolinium salt at the 4-position of the dearomatized isoquinoline ring (Scheme 154). This transformation involved the combination of oxidative coupling and copper-catalyzed halogen atom-transfer radical processes. Different substituents at the C5-position of the isoquinoline core were tolerated. In addition, the reaction was not sensitive to the electronic properties of the substituents on the aryl ring of the benzyl moiety (R = Ar). The ether component of the reaction could be benzodioxoles, dioxolanes, tetrahydrofurans and tetrahydropyranes.

Control experiments were performed to gain insight into the mechanism of this transformation. In this context, the initial Cu-catalyzed homolytic decomposition of TBHP would generate a *t*-BuO radical, responsible for the hydrogen abstraction from the 2-position of THF to give the nucleophilic α-oxyalkyl radical **A**. This radical would add to the C1-position of the isoquinolinium bromide, leading to the electrophilic radical cation **B** with its resonance structure **C** (Scheme 149). At the same time, the bromine counterion would incorporate in the Cu(acac)_2_ coordination sphere and this would mediate an atom transfer radical, rendering intermediate **D**, which would evolve to the final product by deprotonation.

**Scheme 150:**
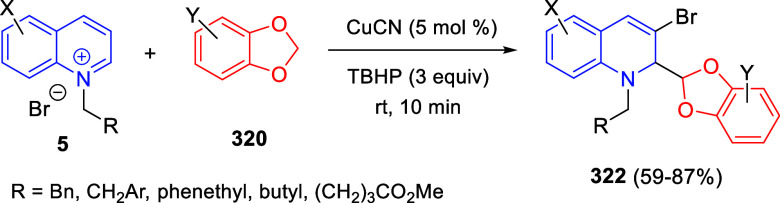
Cu-Catalyzed Difunctionalization of Quinolinium Salts through an Atom-Transfer Radical Process

This protocol was later extended to quinoline derivatives (Scheme 150).^218^ In this case, CuCN was employed as the catalyst and benzo[*d*][1,3]dioxoles **320** as precursors of the α-oxyalkyl radical, providing the 2-functionalized dihydroisoquinolines **322**, with incorporation of the quinolinium salt counterion at the 3-position of the dearomatized quinoline ring. Other acetals such as dioxolane, tetrahydrofuran, tetrahydropyran, 1,4-dioxane, or acyclic ethers provided the desired products in very low yields.

**Scheme 151:**
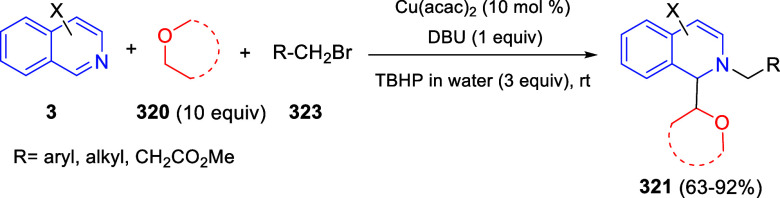
Cu-Catalyzed 1,2-Difunctionalization of Isoquinolines Salts via a Radical Addition/Reduction Process

The same authors found that, when the reaction was performed in the presence of a base and an alkyl halide, a reduction process took place, rendering 1,2-dialkylation products (Scheme 151).^219^ In this work, isoquinolines **3** and different ether derivatives **320** reacted in the presence of alkyl bromides **323**, *tert*-butyl hydroperoxide (TBHP), 1,8-diazabicyclo[5.4.0]-undec-7-ene (DBU), and Cu(acac)_2_ as the catalyst, affording 1,2-dihydroisoquinolines **321** in good yields. The reaction efficiency was not sensitive to the electronic properties of the substituents on the isoquinoline ring. Likewise, alkyl, aryl, and ester groups on the alkyl bromide (R^2^) worked efficiently. The process was also extended to quinolines, leading to the corresponding dihydroquinolines. This transformation involved the combination of an oxidative coupling by Cu/TBHP and a reduction process by DBU. The radical cation generated after the addition of the α-oxyalkyl radical (see Scheme 149) to the isoquinoline would capture an electron from the tertiary amine to render the final products.

**Scheme 152:**
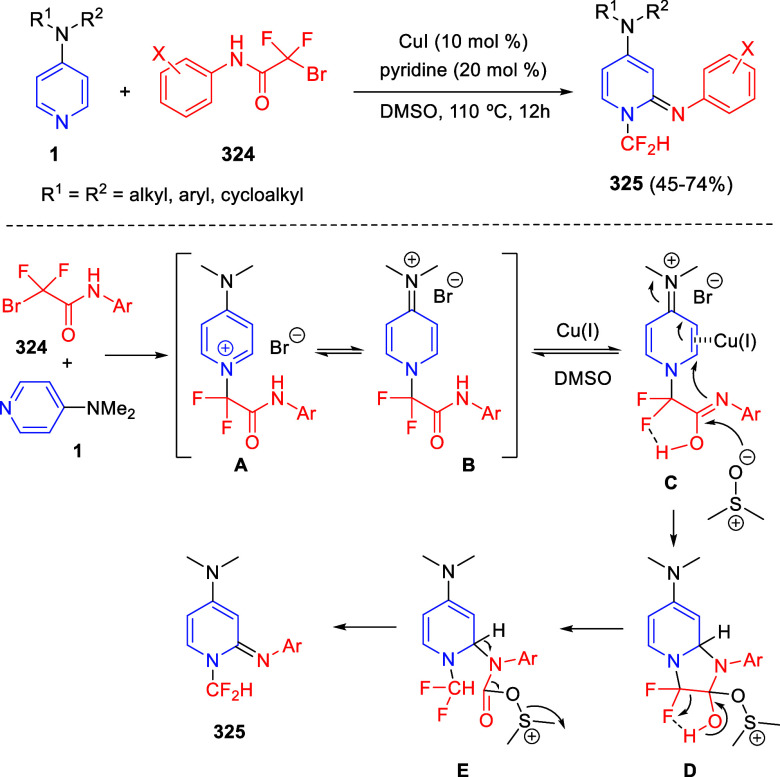
Copper-Catalyzed Dearomatization and Difunctionalization of Pyridines with *N*-Aryl Bromodifluoroacetamides

In 2020, Wang and co-workers disclosed a copper-catalyzed dearomatization and 1,2-difunctionalization of 4-aminopyridines **1** with *N*-aryl bromodifluoroacetamides **324** (Scheme 152).^220^ The reaction took place in the presence of CuI as the catalyst and pyridine as a base, and rendered *N*-difluoromethyl-2-imine dihydropyridines **325** in moderated to good yields. During the process, bromodifluoroacetamides were broken and two of the fragments were incorporated into the final product. A wide variety of substituents on the *N*-aryl bromidifluoroacetamide was compatible with the process. On the other hand, several amines were also good partners for this reaction, including aromatic, aliphatic, or cyclic amines. When the reaction was performed in the presence of oxygen, the bromine atom from the starting difluoroacetamide was incorporated at the 2-position of the aromatic ring, in an electrophilic bromination event.

The reaction of the difluoroacetamide with the aminopyridine would lead to pyridinium salt **A** or **B**, which could undergo keto–enol tautomerism to give intermediate **C** (Scheme 152). Then, nucleophilic addition of DMSO would trigger the copper-catalyzed intramolecular aza-Michael addition, leading to intermediate **D**. This would rearrange to intermediate **E**, which would fragment into CO_2_, dimethyl sulfide, and the final product **325**.

**Scheme 153:**
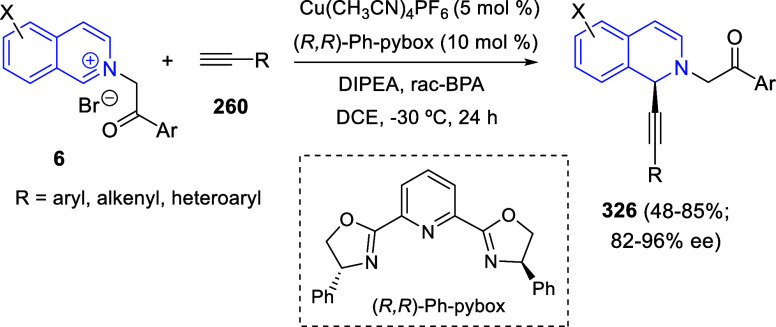
Cu/(Ph-pybox)-Catalyzed Asymmetric Dearomative Alkynylation of Isoquinolines

The Cu/Ph-pybox-catalyzed enantioselective dearomative 1,2-alkynylation of isoquinolines was reported by Guan and co-workers in 2020 (Scheme 153).^221^ The optimized conditions involved the reaction of quinolinium salts **6** and alkynes **260** with Cu(CH_3_CN)_4_PF_6_ as the catalyst, 2,6-bis(oxazolinyl)pyridine (Py-box) as a chiral ligand, BINOL phosphoric acid (BPA) as an additive, and diisopropylethylamine (DIPEA) as a base (1.5 equiv). Under these conditions, 1-alkynyl dihydroisoquinolines **326** were obtained in good yields with excellent enantioselectivities. Substitution on the isoquinoline ring was tested at the C6-position with halogens, while on the aryl ketone-position, different 4-substituted aromatic rings provided good results either with electron-donating and electron-withdrawing groups. Finally, a variety of terminal alkynes **260**, with aryl, heteroaryl, and alkynyl substituents were compatible with the process.

**Scheme 154:**
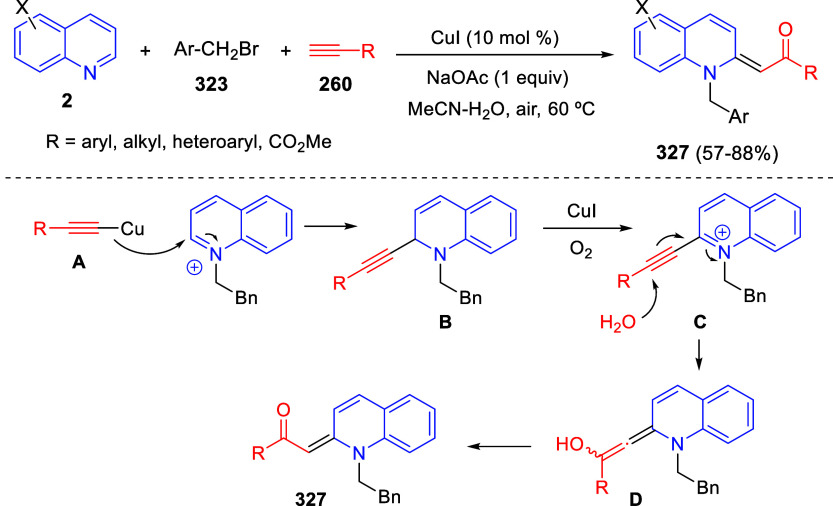
*N*-Alkylation/Alkenylation Tandem Process for the Construction of 1,2-Difunctionalized Quinoline Derivatives

An efficient strategy for constructing 1,2-difunctionalized quinoline derivatives entailed the copper-catalyzed three-component cascade coupling of quinolines, benzyl bromides **323** and terminal alkynes **260** (Scheme 154).^222^ The combination of these type of compounds with CuI as the catalyst and NaOAc as a base under air atmosphere, provided 1,2-dihydroquinoline derivatives **327** bearing a conjugated ketone moiety. Alkyl, aryl, and heteroaryl alkynes and propiolates were tolerated in the process. Substitution on starting quinolines was allowed at the C5–C7-positions of the quinoline ring, with little effect on the process. After the initial formation of copper acetylide **A**, 1,2-addition over the *in situ* generated quinolinium salt would render dihydroquinoline **B** (Scheme 154). In the presence of the copper catalyst and oxygen, this compound would be oxidized to the quinolinium salt **C**, which would undergo 1,4-addition of a water molecule to render allene derivative **D**. Final tautomerization would give rise to the final products.

**Scheme 155:**
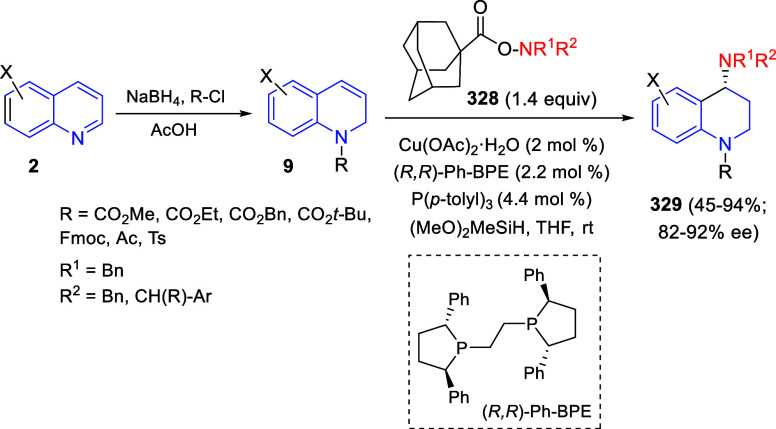
1,2-Reductive Dearomatization of Quinolines and Copper(I) Hydride-Catalyzed Asymmetric Hydroamination

The groups of Zhang and You developed a 1,2-reductive dearomatization of quinolines followed by a copper(I) hydride-catalyzed asymmetric hydroamination sequence to provide amine-substituted tetrahydroquinolines with high enantiocontrol (Scheme 155).^223^ Once the initial 1,2-nucleophilic addition of the hydride was effected, the resulting *N*-protected 1,2-dihydroquinolines **9** were subjected to the hydroamination reaction. The combination of Cu(OAc)_2_·H_2_O, the chiral ligand (*R*,*R*)-Ph-DPE, *p*-tolyl phosphine as an additive, and dimethoxymethylsilane as the hydride transfer reagent generated the catalytically active copper hydride species, and then 1-adamantyl acid-derived hydroxylamine esters **328**, as the sources of electrophilic amines, reacted to provide 4-amino tetrahydroquinolines **329** in good yields and enantioselectivities. Several protecting groups on the nitrogen atom of the 1,2-dihydroquinolines were well tolerated, as well as various substituents at the 6-position of the dihydroquinoline ring. A variety of nitrogen sources **3** bearing various benzylic moieties were also compatible with this methodology.

#### 6.1.2 Iridium-Based Protocols

**Scheme 156:**
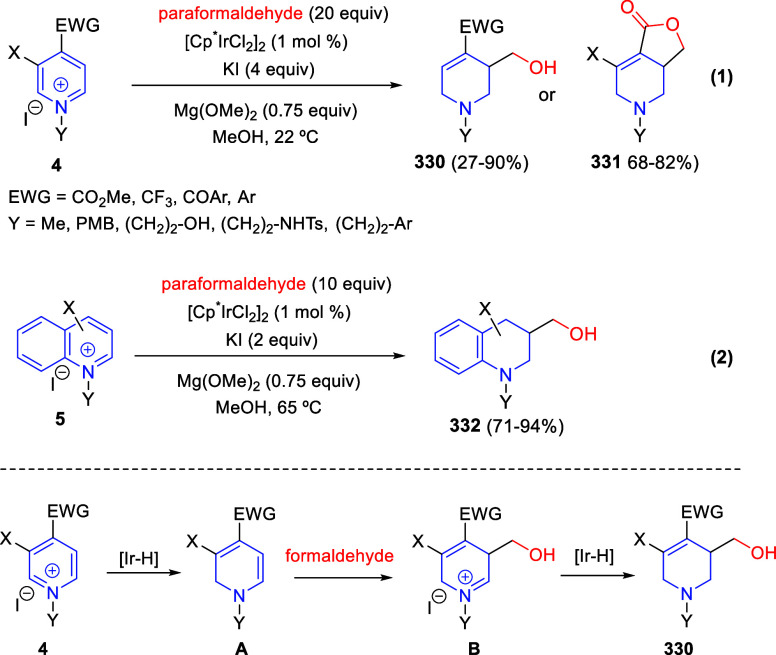
Reductive C3-Hydroxymethylation of Pyridinium and Quinolinium Salts through Iridium-Catalyzed Interrupted Transfer Hydrogenation

Donohoe and co-workers developed a reductive C3-functionalization of pyridinium- and quinolinium salts through an iridium-catalyzed interrupted transfer hydrogenation protocol (Scheme 156).^224^ Methanol and formaldehyde were employed for the reductive hydroxymethylation of pyridinium salts **4** in the presence of [Cp*IrCl_2_]_2_ as the catalyst, Mg(OMe)_2_ as a base, and KI as an additive, rendering trisubstituted tetrahydropyridines **330** in generally high yields. Different electron-withdrawing groups were tolerated at the C4-position of the pyridinium salt, as well as several alkyl groups at the N atom. In addition, when a substituent was present at the C3-position (X ≠ H), the resulting products were found to lactonize, giving bicycles **331** also in good yields (Scheme 156, eq 1). This protocol was further extended to quinolinium salts **5** and, in this case, heating the reaction mixture was necessary in order to achieve excellent yields of tetrahydroquinolines **332** (Scheme 156, eq 2). Electronically diverse substituents at the C3-, C6-, and C7-positions of the starting quinolinium were tolerated in the process.

In this reaction, an iridium-hydride species would be initially formed through addition of methanol to formaldehyde followed by oxidation by iridium. Then, the 1,2-reduction of the pyridine ring would occur and the subsequent nucleophilic addition of the enamine moiety in intermediate **A** to formaldehyde would lead to iminium salt **B**, which would be again reduced by the Ir-hydride to render the final products (Scheme 156).

This methodology was further extended to pyridinium salts bearing heterocyclic rings at the C4-position.^225^

**Scheme 157:**
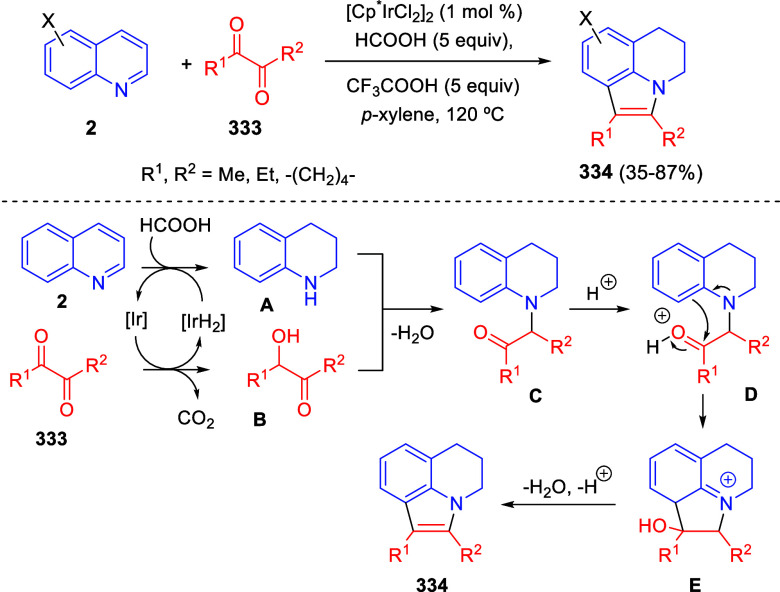
Iridium/Acid-Cocatalyzed Synthesis of Fused Indoles via Transfer Hydrogenative Annulation of Quinolines and 1,2-Diketones

In 2020, Zhang and co-workers reported the synthesis of fused indoles by means of a transfer hydrogenative annulation of nonactivated quinolines **2** and 1,2-diketones **333** employing formic acid as the hydrogen source and trifluoroacetic acid and [Cp*IrCl_2_]_2_ as the catalytic system (Scheme 157).^226^ A range of fused indoles **334** were obtained in moderate to good yields, with dearomatization of the quinoline ring. Quinolines bearing electron-donating groups afforded the final products in higher yields than those bearing electron-withdrawing groups. On the other hand, reactions with nonsymmetric diketones afforded mixtures of regioisomers, being the major products those arising from reacting the sterically less-hindered carbonyl group with the nitrogen site.

Control experiments were conducted to gain insights into the reaction mechanism. In this context, reaction of the hydrogen donor, HCOOH, with the Ir catalyst would form the metal hydride species [IrH_2_], which would transfer hydrogen to the quinoline ring to form tetrahydroquinoline **A** (Scheme 157). This redox process would be coupled with the carbonyl reduction to give hydroxyketone **B**, thereby regenerating the starting Ir species. Then, nucleophilic addition of the tetrahydroquinoline nitrogen to the hydroxy-ketone would render intermediate **C** after tautomerization. Intramolecular nitrogen-promoted addition of the quinoline C8 site to the carbonyl would generate intermediate **D** that would evolve to final products **324**. With the present catalyst system, the reduction of the quinoline ring would be faster than the reduction of the 1,2-diketone, thus offering high chemoselectivity.

**Scheme 158:**
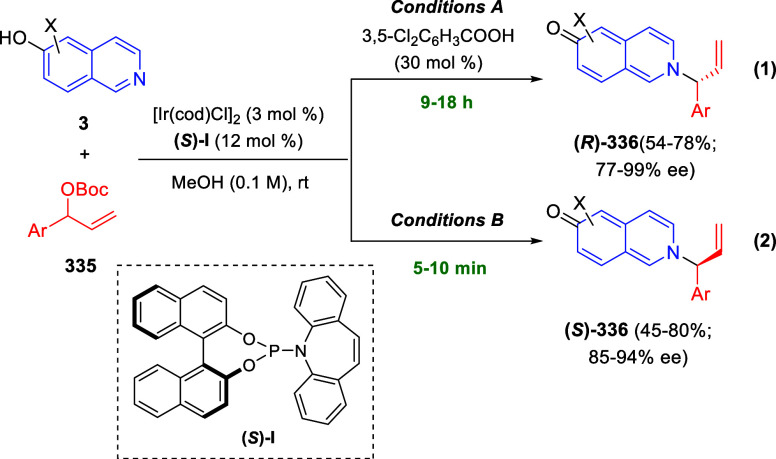
Time-Dependent Enantiodivergent Allylic Amination of Hidroxiquinolines Using the Same Chiral Ir Catalyst

In 2020, You and co-workers reported an elegant time-dependent enantiodivergent Ir-catalyzed allylic substitution reaction of 6-hydroxyquinolines that enabled the synthesis of both enantiomers of the dearomatized products employing the same catalyst (Scheme 158).^227^ The reaction of isoquinolinols **3** with racemic cinnamyl carbonates **335** and the [Ir(cod)Cl]_2_ complex in the presence of Carreira’s chiral phosphorimidite ligand (***S***)-**I** and 3,5-dichlorobenzoic acid afforded, after 9–18 h at room temperature, the amination isoquinolinone products (*R*)-**336** in good yields with excellent enantioselectivities (Scheme 158, eq 1). On the other hand, when the reaction was carried out without the Brønsted acid and quenched after 5–10 min, the opposite enantiomer (*S*)-**336** was obtained instead (Scheme 158, eq 2). Mechanistic studies revealed that two kinetic resolutions occurred sequentially, one with the cinnamyl carbonate and the other for the amination products. Different substituents were tolerated on the aromatic ring of cinnamyl carbonates. Substitution on starting 6-isoquinolinols was allowed at the C7- and C8-positions and also the process was compatible with 8-hydroxyquinolines.

**Scheme 159:**
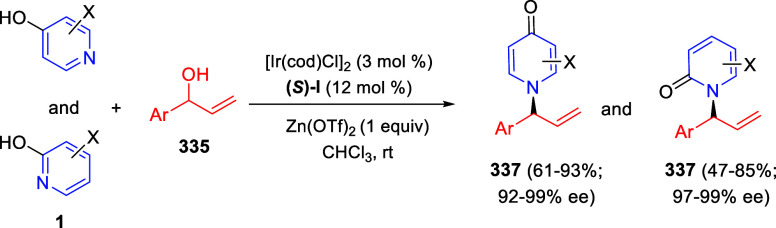
Iridium-Catalyzed Intermolecular Asymmetric Allylic Amination with Hydroxypyridines

An analogous iridium-catalyzed intermolecular asymmetric allylic amination was developed for 4- and 2-hydroxypyridines **1** with allylic alcohols **335** to render *N*-allylated 2-pyridones and 4-pyridones **337** in good yields with excellent enantioselectivities (Scheme 159).^228^ Mechanistic studies indicated that the reaction was a kinetic resolution process and it displayed a broad substrate scope for both pyridines and allylic alcohols. Moreover, the reaction was successfully performed on a gram-scale and the amination product was further derivatized to prove the practicality of this method.

**Scheme 160:**
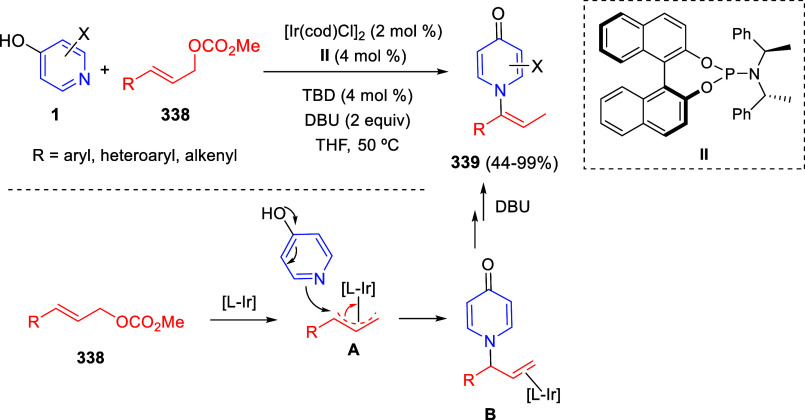
Iridium-Catalyzed Propenylation Reactions of 4-Hydroxypyridines with Allylic Carbonates

He and co-workers had previously described an iridium-catalyzed propenylation reaction of 4-hydroxypyridines with allylic carbonates (Scheme 160).^229^ The iridium catalyst was generated *in situ* from complex [Ir(cod)Cl]_2_ and Feringa’s phosphoramidite **II** with 1,5,7-triazabicyclo[4.4.0]dec-5-ene (TBD). 4-Hydroxypyridines **1** reacted with allylic carbonates **346** in the presence of the iridium catalyst and DBU as a base to give *N*-alkenyl pyridones **347** in good yields by means of a tandem allylic substitution/isomerization sequence. Different aryl substituents on the allylic carbonates were tolerated in the process, independently of their electronic properties. Heteroaryl and alkenyl carbonates also performed efficiently, while aliphatic carbonates afforded the nonisomerized product. In addition, the process was amenable to electron-rich and electron-poor pyridines.

Preliminary mechanistic studies allowed the authors to propose that this reaction would proceed through a regular allylic substitution reaction, by formation of the π-allyl iridium complex **A** and further addition of the isoquinolinol with final decomplexation. Then, isomerization of the alkene would be promoted by DBU, rendering the final propenylation products (Scheme 160).

**Scheme 161:**
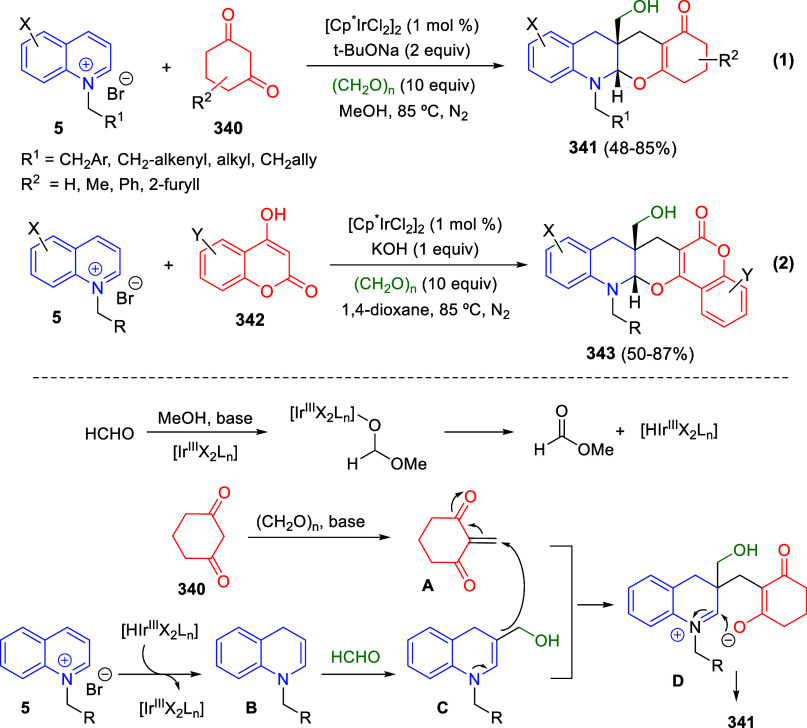
Iridium-Catalyzed Reductive Three-Component Annulation Reaction with Quinolinium Salts

A three-component reductive annulation reaction among quinolinium salts **5**, formaldehyde, and cyclic 1,3-diketones **340** was catalyzed by the iridium catalyst [Cp*IrCl_2_]_2_ in the presence of *t*-BuONa as a base, to give tetrahydroquinoline-fused pyran derivatives **341** in good yields as single diasteroisomers (Scheme 161, eq 1).^230^ Substituents at different positions of the quinoline ring were compatible with the process, although electron-donating groups provided lower yields of the final products than electron-withdrawing ones. The reaction was further extended to 4-hydroxycoumarins **342** and, employing KOH as a base, the corresponding pentacyclic derivatives **343** were obtained as single diasteroisomers (Scheme 161, eq 2).

Based on several control experiments, the authors proposed a plausible mechanism for this transformation that would initiate by the base-promoted methanol addition to formaldehyde, with anion exchange with the iridium complex, followed by β-hydride elimination to form ethyl formate (detected by GC-MS) and the catalytically active Ir-hydride species (Scheme 161). This hydride would react with the quinolinium salt to render dihydroquinoline **B** that would undergo enamine addition to formaldehyde, generating intermediate **C**. Simultaneously, Knoevenagel condensation of 1,3-diketone with formaldehyde would form conjugated diketone **A**, which would undergo another enamine attack from intermediate **C** to furnish intermediate **D**, which would cyclize by the addition of the alkoxide to the iminium salt.

**Scheme 162:**
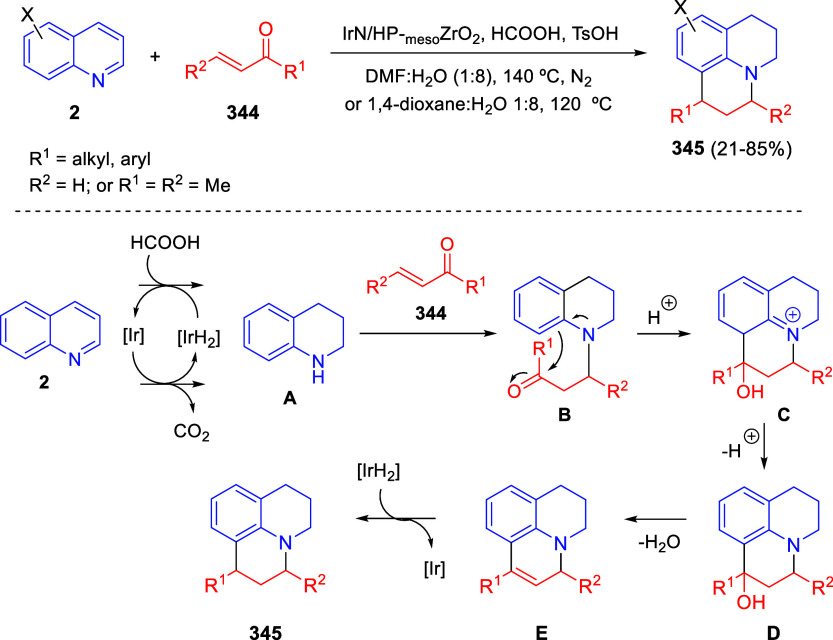
Reductive Annulation of Quinolines and Conjugated Enones by a MOF-Derived Iridium Catalyst

Very recently, Zhang’s group described the development of a MOF-derived hierarchically porous ZrO_2_-supported iridium catalyst (Ir-N@HP-_meso_ZrO_2_), which was applied to the synthesis of the julolidine skeleton by a reductive annulation protocol (Scheme 162).^231^ This is another example of merging the hydrogen-transfer-mediated activation of heteroarenes with *in situ* incorporation in a coupling sequence. Quinolines **2** reacted with conjugated ketones **344** in the presence of an iridium catalyst supported in a Zr-MOF, HCOOH as a hydrogen source, and TsOH as a cocatalyst, to render tetrahydropyridoquinolines **345** in moderate to good yields. Various conjugated aryl and alkyl terminal enones were employed, although the reaction with enones substituted at the C4-position (R^1^ = R^2^ = Me) provided lower yields. By the same token, C5- and C6-substituted quinolines were amenable for the process, regardless the electronic properties of the substituents.

The Ir-supported catalyst and HCOOH would initially form the Ir-hydride species, responsible for catalysis. The acidity of ZrO_2_ would facilitate the absorption of the quinoline on the catalyst surface, where a chemoselective reduction to tetrahydroquinoline **A** would take place. Then, 1,4-addition to the conjugated ketone would give intermediate **B** (Scheme 162). Under acidic conditions, the nitrogen of the tetrahydroquinoline would promote nucleophilic addition to the carbonyl group through the C8-position of the ring, rendering intermediate **C**, which, after loss of proton and water, would form intermediate **E**. Finally, transfer hydrogenation from the Ir-hydride species would render the final products **345**.

#### 6.1.3 Magnesium-Based Protocols

**Scheme 163:**
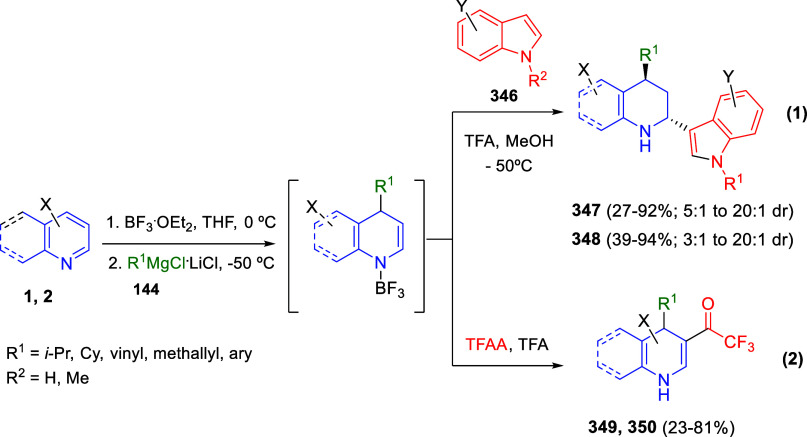
C–C Bond-Forming Dearomatizations of Pyridines and Quinolines

In 2019, the group of Wang described a one-pot dearomative double nucleophilic addition to pyridines and quinolines (Scheme 163).^232^ The activation of the quinoline or pyridine ring by BF_3_·OEt_2_ followed by addition of Grignard reagents **144** resulted in the C4-selective nucleophilic addition to form the corresponding dearomatized compounds that were protonated with methanol. The resulting electrophilic iminium ions underwent intermolecular Friedel–Crafts addition of indole derivatives **346** to render tetrahydropyridines **347** and tetrahydroquinolines **348** in good yields, with incorporation of both nucleophiles at the same time (Scheme 163, eq 1). In order to achieve good selectivity, the addition of the second nucleophile was performed at −50 °C, providing, in most cases, excellent diasteroselectivities in the formation of the *anti*-substituted kinetic products. Electron-donating and electron-withdrawing substituents at different positions of the heteroarene ring were well tolerated, as well as on the Grignard reagent and indole nucleophiles.

Soon after, the same group performed an analogous nucleophilic dearomatization of pyridines and quinolines with Grignard reagents, this time with trifluoroacetic anhydride (TFAA) as electrophile in the second step. Thus, enamine nucleophilic attack of the intermediate dearomatized heterocycles gave access to 3,4-disubstituted dihydropyridines **349** and dihydroquinolines **350** in moderate to good yields (Scheme 163, eq 2).^233^ Substitution at the C5–C6-positions of the quinoline ring and at the C5-position of the pyridine ring was compatible with the process, independently of the electronic properties of the substituents. Likewise, aryl, alkenyl, and alkyl Grignard reagents provided good yields of the final products.

**Scheme 164:**
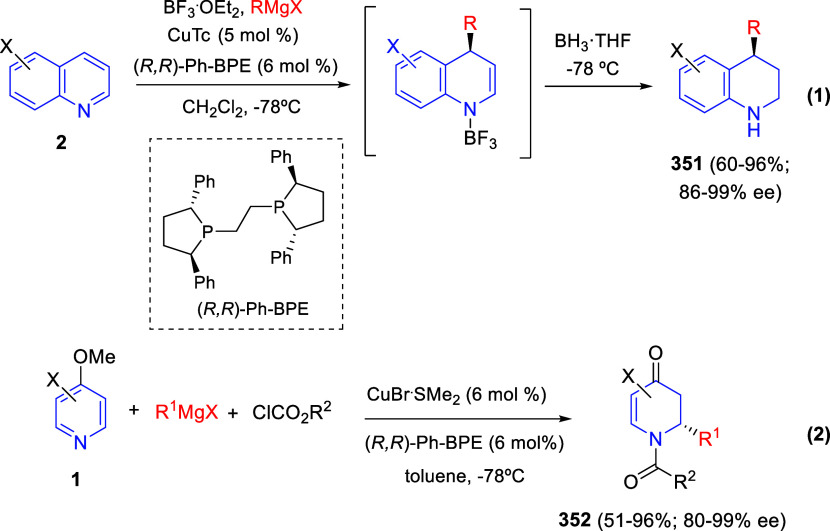
Nucleophilic Dearomatization of *N*-Heterocycles with Grignard Reagents Enabled by Chiral Copper Catalysis

The enantioselective version of the dearomative addition of Grignard reagents to quinolines was performed by Harutyunyan and co-workers employing a chiral copper catalyst (Scheme 164).^234^ The reaction of quinolines **2** with BF_3_·OEt_2_ and Grignard reagents in the presence of copper(I) thiophene-2-carboxylate (CuTc) as the catalyst and (*R*,*R*)-Ph-BPE as a chiral ligand proceeded in a complete regioselective manner, to form the corresponding 4-substituted dihydroquinoline compounds, which were treated *in situ* with borane, rendering the final tetrahydroquinoline derivatives **351** in good yields with excellent enantioselectivities (Scheme 164, eq 1). A variety of alkyl Grignards were compatible with the process, while different substituents at the C5–C8-positions of the quinoline ring were also tolerated.

This methodology for the enantioselective alkylation of quinolines with alkyl Grignard reagents was further applied to *in situ* generated *N*-acylpyridinium salts, in this case with CuBr·SMe_2_ as the catalyst. This transformation gave rise to enantiopure chiral dihydro-4-pyridones **352** in good yields and ee values (Scheme 164, eq 2).^235^

**Scheme 165:**
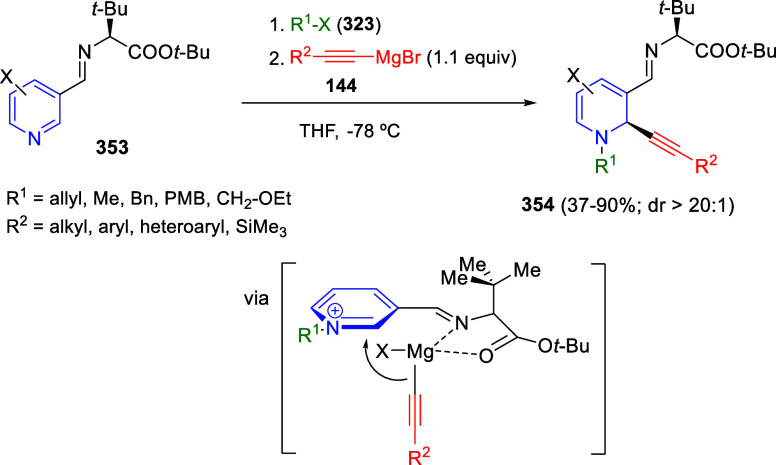
Regioselective Asymmetric Alkynylation of *N*-Alkyl Pyridiniums with Grignard Reagents

A detailed study on the dearomative addition of Grignard reagents to *N*-alkyl pyridinium electrophiles was carried out by Smith and co-workers in 2020.^236^ They found that the regiochemical outcomes had predictable trends associated with the pyridinium substitution patterns. The understanding of the innate electrophilic regioselectivity of substituted pyridines would help to design selective protocols, considering steric, electronic and chelating effects, and predict the place of the preferred addition. Chelating groups, such as amides, would exhibit an *ortho*-directing effect, due to chelation with the nucleophile; resonance electron-withdrawing groups primarily would promote addition to the *para*-position, while alkyl groups, halides, and heteroatoms also would promote *ortho*-addition to the pyrinium ring. This working model for the regioselective attack of Grignard nucleophiles on substituted pyridiniums was applied to the asymmetric addition of alkynyl nucleophiles to *N*-alkylpyridinium electrophiles (Scheme 165).^237^ Thus, 2-pyridine carbaldehydes were condensed with protected *tert*-leucine to obtain the starting imines **353**, which, by successive treatment with differently substituted alkyl halides **323** and alkynyl magnesium bromides **144**, afforded 2-alkynylated dihydropyridines **354** in moderated to good yields with complete regio- and diastereoselectivity. These products were very useful for the asymmetric synthesis of various aza-heterocyclic building blocks.

A stereochemical model for this asymmetric alkynylation reaction would involve that chelation of the magnesium center with the imino group would guide the nucleophile to the C2-position of the pyridine ring (Scheme 165). Additionally, the *t*-butyl group of the amino acid would block the top face of the pyridinium so the nucleophile approach should take place from the bottom face, providing excellent stereocontrol.

#### 6.1.4 Rhodium-Based Protocols

**Scheme 166:**
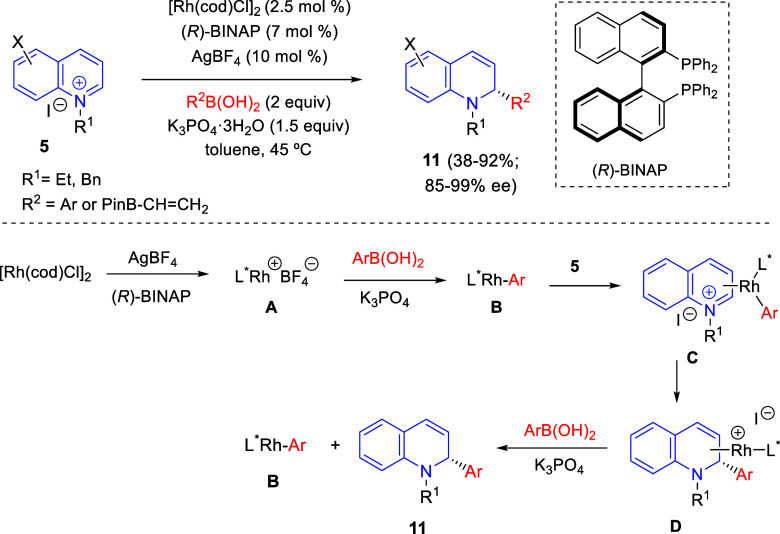
Enantioselective Rhodium(I)-Catalyzed Dearomative Arylation or Alkenylation of *N*-Alkylquinolinium Salts

An enantioselective rhodium(I)-catalyzed dearomative arylation or alkenylation of *N*-alkylquinolinium salts was developed by the groups of Wei and Wang (Scheme 166).^238^ This process involved the 1,2-nucleophilic addition of boronic acids to quinolinium salts **5** in the presence of [Rh(cod)Cl]_2_, AgBF_4_, K_2_HPO_4_, and the chiral phosphine (*R*)-BINAP, affording enantiomerically enriched 2-substituted 1,2-dihydroquinolines **11** in generally good yields with excellent enantioselectivities. The reaction tolerated a wide range of functional groups in both the organic boronic acids and the quinoline counterpart. Additionally, it was also efficient with vinyl boronic pinacol ester as the nucleophile.

Regarding a plausible catalytic cycle, the authors proposed that AgBF_4_, in combination with the chiral ligand, would convert the starting rhodium precatalyst, [Rh(cod)Cl]_2_, into a cationic rhodium complex **A**, which in turn would undergo transmetalation with the boronic acid to generate the nucleophilic arylrhodium species **B** (Scheme 166). Then, coordination of the quinoline ring would form iminium intermediate **C**. Its insertion into the C–Rh bond would give species **D** and final transmetalation would render product **11** and regenerate the aryl rhodium species **B**.

**Scheme 167:**
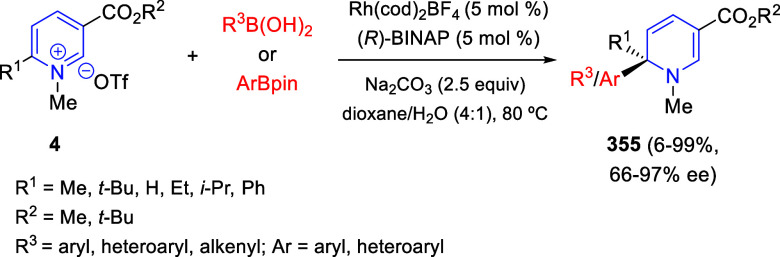
Rhodium-Catalyzed Enantioselective Dearomatizaton of Pyridinium Salts

An analogous catalytic enantioselective dearomatization of pyridine derivatives with boronic acids as nucleophiles was described by Karimov and co-workers (Scheme 167).^239^ Specifically, the reaction of *N*-alkyl nicotinic acid-derived pyridinium triflates **4** with aryl boronic acids, Rh(cod)_2_BF_4_/(*R*)-BINAP as the catalytic system and Na_2_CO_3_ as a base in dioxane/H_2_O, delivered 1,2-dihydropyridines **355** bearing a quaternary stereocenter. Substitution on the pyridine ring was well tolerated, independently of the position and electronic properties of the substituents. Likewise, boronic acids containing various aryl/heteroaryl and alkenyl substituents gave the corresponding dearomatization products in good yields and excellent enantioselectivities. It is important to note that dihydropyridines **355** contain two double bonds with different reactivities. Therefore, the authors performed several reductive derivatization reactions to demonstrate that those double bonds could be selectively functionalized.

Two years later, the same authors reported a related rhodium-catalyzed enantioselective dearomatization of pyridinium salts by means of the nucleophilic addition of aryl and heteroaryl boron pinacol esters (ArBpin), more stable than boronic acids.^240^ Under the same optimized conditions, 1,2-dihydropyridines **355**, containing either a tertiary or a quaternary stereocenter were achieved in good yields and enantiocontrol (Scheme 167). The methodology was also efficiently applied to quinoline derivatives.

**Scheme 168:**
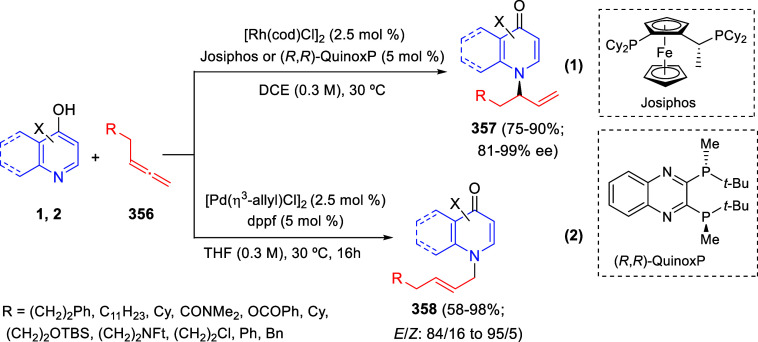
Regiodivergent and Stereoselective Transition Metal-Catalyzed Addition of 4-Pyridones to Allenes

In 2017, Breit and co-workers reported a regiodivergent addition of 4-hydroxypyridines and 4-hydroxyquinolines to allenes (Scheme 168).^241^ In this work, 4-hydroxypyridines **1** and 4-hydroxyquinolines **2** reacted with allenes **356** bearing a wide variety of alkyl moieties, in the presence of a rhodium catalyst [Rh(cod)Cl]_2_ and the chiral ligand Josiphos [or (*R*,*R*)-QuinoxP in some cases] to render, as the major products, branched *N*-allyl hydroxypyridones- and quinolones **357** in good yields with excellent enantioselectivities (Scheme 168, eq 1).

The authors also found that when palladium catalyst [Pd(η^3^-allyl)Cl]_2_ was employed, linear *N*-alkyl-pyridones and quinolones **358** were formed preferentially over the branched derivatives, being the *trans* isomer of the double bond the major or exclusive product in all cases (Scheme 168, eq 2). The scope of the process was comparable to that of the reaction with the rhodium catalyst.

**Scheme 169:**
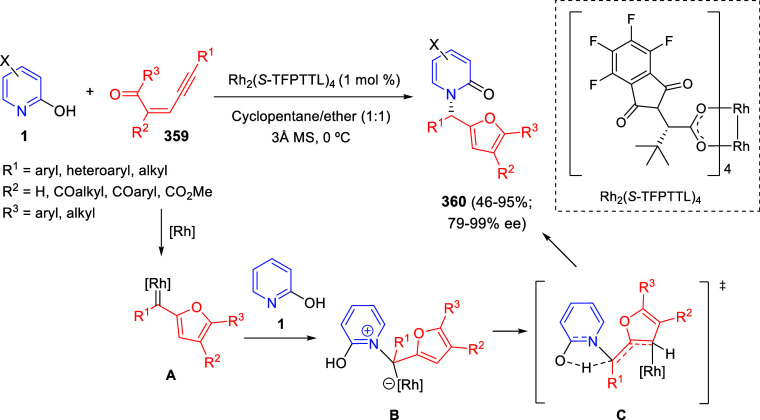
Rh-Catalyzed Chemo- and Enantioselective Insertion of Furyl Carbenes into the N–H Bond of 2-Pyridones

More recently, Sun, Zhang, and co-workers described an enantioselective rhodium-catalyzed insertion of electrophilic rhodium carbenes into 2-hydroxypyridines **1** (Scheme 169).^242^ Their reaction with enynones **359** in the presence of the chiral rhodium catalyst Rh_2_(S-TFPTTL)_4_ provided the *N*-alkylation 2-pyridone products **360** in generally good yields with excellent enantioselectivities. Although this insertion usually takes place preferentially in the O–H bond, under the optimized conditions, the authors were able to perform the N–H insertion. A wide range of functional groups on the starting enynones were well tolerated. Regarding the pyridine moiety, the process was efficiently carried out with C3–C5 substituted pyridines, regardless of the electronic nature of the substituents. DFT calculations were performed to understand the reaction mechanism. It it is well-known that enynones react with rhodium catalysts to form 2-furyl-metal carbenoid **A**. Then, the reaction would proceed through enantioselective pyridinium ylide formation and sequential 1,4-proton transfer (Scheme 169).

#### 6.1.5 Paladium-Catalyzed Protocols

**Scheme 170:**
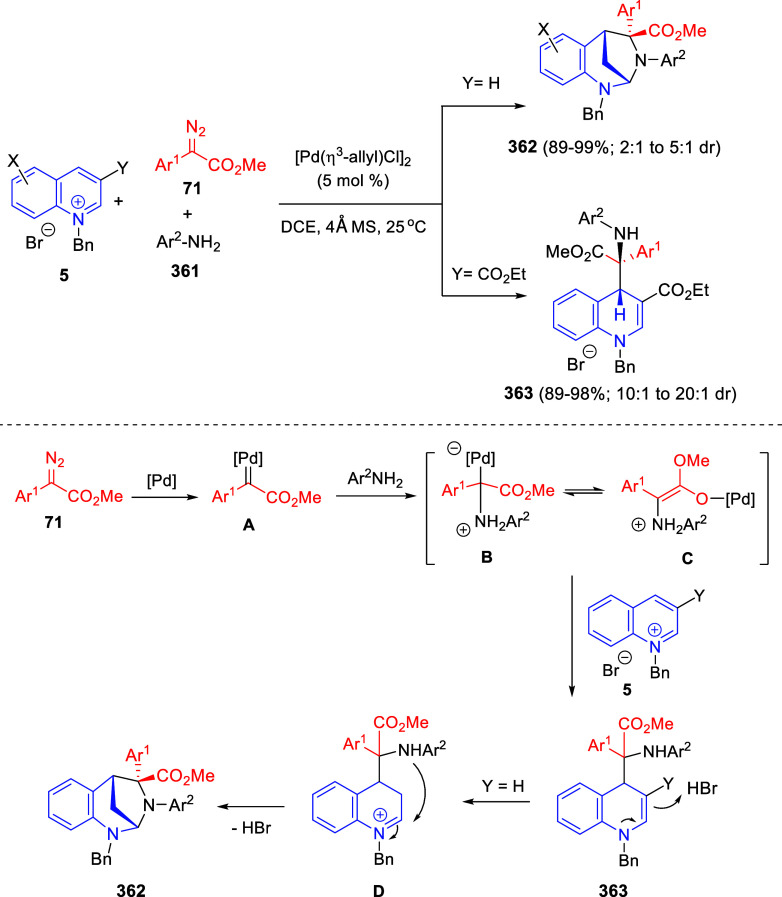
Regio- and Diastereoselective Three-Component Reactions via Trapping of Ammonium Ylides with Quinolinium Salts

In 2017, the group of Hu described a palladium-catalyzed three-component reaction of diazocompounds **71**, anilines **361**, and quinolinium salts **5** (Scheme 170).^243^ Ammonium ylides, generated *in situ* from diazocompounds and anilines, reacted with quinolinium salts **5** in the presence of palladium catalyst [PdCl(η^3^-C_3_H_5_)]_2_ to render bridged 1,3-benzodiazepine derivatives **362** in excellent yields with moderate to good diastereoselectivities. Anilines with various substituents at the *para*- and *meta*-positions were tolerated in the process, as well as substituted diazocompounds with electron-withdrawing or electron-donating groups on the phenyl ring. Regarding the quinoline ring, electronically different substitution was compatible at the C5–C8-positions. However, when an ester group was present at the C3-position, the reaction led to 1,4-dihydroquinolines **363** in excellent yields and dr values.

A mechanism for this reaction was proposed according to control experiments and previous investigations. Initially, the Pd catalyst would decompose the diazocompound to form the electrophilic palladium carbene species **A**, which would react with the aniline to generate Pd-derived ammonium intermediate **B** and its enolate counterpart **C** (Scheme 170). This would be trapped by the quinolinium salt via 1,4-conjugate addition to provide 1,4-dihydroquinolines **363**, along with HBr. Finally, the enamine moiety would be protonated to the iminium ion **D** that would undergo intramolecular nucleophilic cyclization to afford the bridged products **362**. When an ester group was present at the C3-position, this cyclization would not take place, most likely due to the stabilization of the enamine moiety by the electron-withdrawing ester group.

**Scheme 171:**
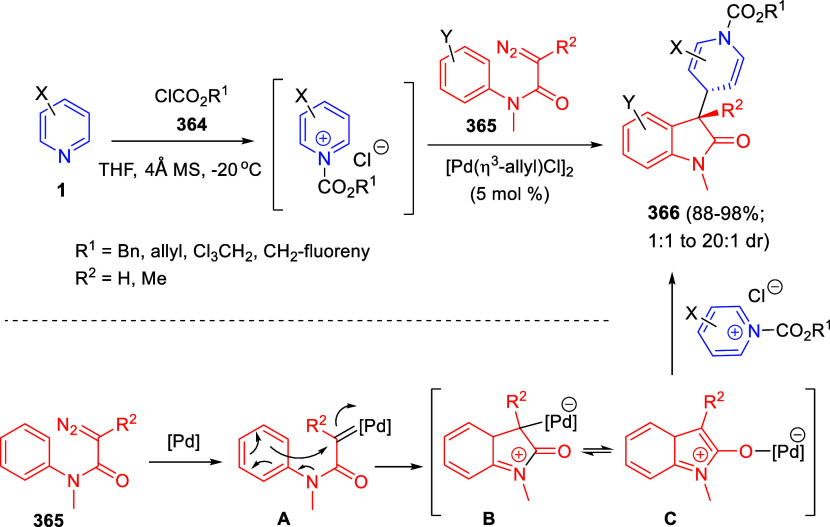
Palladium-Catalyzed Diastereoselective Three-Component Reaction of *N*-Aryl Diazoamides, Pyridines, and Chloroformates

The same authors also reported a palladium-catalyzed three-component reaction of pyridine derivatives **1**, chloroformates **364**, and *N*-aryl diazoamides **365** (Scheme 171).^244^ The reaction proceeded through trapping of transient zwitterionic intermediates (**B**–**C**) by *in situ* formed *N*-acylpyridinium salts in a regioselective 1,4-addition fashion, giving access to biologically relevant 4-(2-oxoindolin-3-yl)-1,4-dihydropyridine derivatives **366** in high yields with moderate to very good diastereoselectivities. Different chloroformates efficiently gave the corresponding products. Regarding the *N*-aryl diazoamides, different groups were allowed at the *para*-position, while *ortho*-substituted diazoamides failed to produce the final products. Finally, with respect to the substitution on the pyridine ring, an electron-withdrawing group was necessary at the C3-position. Several substituents were tested and only with *N*,*N*-diethyl nicotinamides (X = 3-CONEt_2_) the process took place with excellent diasteroselectivity.

**Scheme 172:**
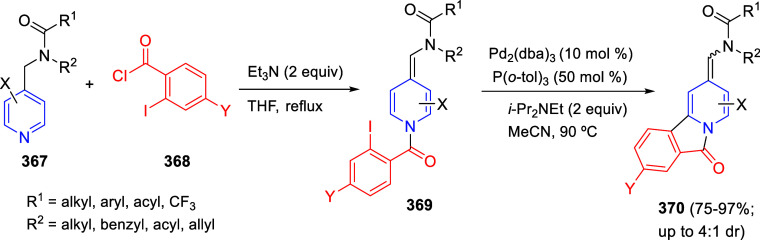
Sequential Pyridine Dearomatization/Mizoroki–Heck Cyclization for the Synthesis of Fused (Dihydropyrido)isoindolinone Derivatives

In 2018, Pigge and co-workers developed a sequence consisting of pyridine dearomatization followed by Pd-catalyzed Mizoroki–Heck cyclization for the synthesis of fused (dihydropyrido)isoindolinone derivatives (Scheme 172).^245^ To achieve this goal, 4-alkylpyridines **367** were treated with 2-iodo benzoyl chlorides **368** to generate functionalized 1,4-dihydropyridines **369**, suitable to undergo an intramolecular Heck reaction in the presence of Pd_2_(dba)_3_, P(*o*-tol)_3_ and Hünig’s base, affording tricyclic derivatives **370** in good yields, as *cis*/*trans* mixtures in the double bond. Different substituents on the lateral chain in the C4-position of the starting pyridines were compatible with the process. Regarding the pyridine ring, aromatic substituents were tested at the C3- and C5-positions with good results, while in the case of the acyl chlorides, different groups could be also incorporated at the C4-position.

**Scheme 173:**
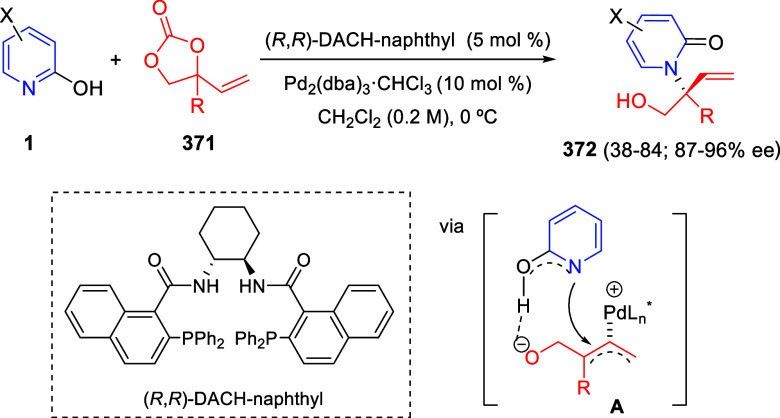
Palladium-Catalyzed Regio- and Enantioselective Amination of Vinyl Cyclic Carbonates with 2-Hydroxypyridines

Very recently, Khan and co-workers reported a regio- and enantioselective allylic amination of cyclic vinyl carbonates **371** and 2-hydroxypyridines **1** (Scheme 173).^246^ Their reaction in the presence of palladium catalyst Pd_2_(dba)_3_·CHCl_3_ and (*R*,*R*)-DACH-naphthyl Trost’s chiral ligand afforded branched *N-*allyl pyridones **372** in good yields with excellent regio- and enantioselectivity. Several substituents at the C3–C5-positions of the pyridine ring were compatible with the process; however, substitution at the C6-position prevented the reaction. On the other hand, a range of alkyl groups were allowed on carbonates **371**, while aromatic carbonates provided lower yields and poorer ee values. It is well-known that vinyl carbonates reacted with palladium complexes with loss of CO_2_ and formation of palladium π-allyl complexes (**A**), responsible for the formation of branched products **372** (Scheme 173).

#### 6.1.6 Ruthenium-Catalyzed Protocols

**Scheme 174:**
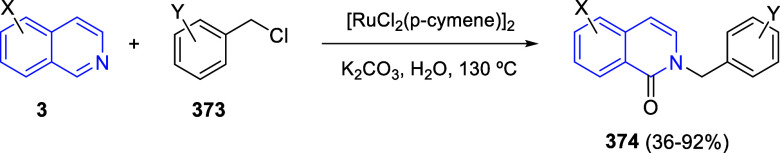
Ruthenium-Catalyzed Dearomatization of Isoquinolines with Alkyl Chlorides

A ruthenium-catalyzed dearomatization of isoquinolines was described by Ong and co-workers in 2016 (Scheme 174).^247^ Thus, the reaction of isoquinolines **3** with benzyl chlorides **373** in the presence of [RuCl_2_(p-cymene)]_2_ as the catalyst and K_2_CO_3_ as a base in water provided *N*-benzylated isoquinolones **374** in moderate to good yields. Various substituents at the C2–C4-positions of the benzyl chloride counterpart, with different electronic properties, were compatible with the process. With respect to the isoquinoline substrate, electron-withdrawing groups were incorporated at the C5-position. When the reaction was performed in an organic solvent, the alkylation of the isoquinoline ring at C1 occurred instead.

**Scheme 175:**
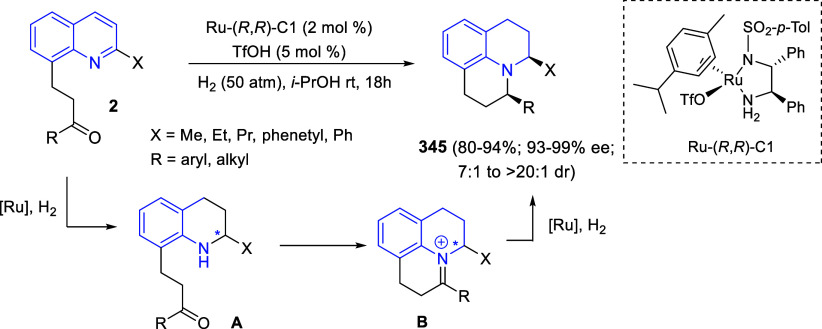
Ru-Catalyzed Cascade Enantioselective Hydrogenation and Reductive Amination for the Construction of Chiral Julolidine Derivatives

The ruthenium-catalyzed construction of the julolidine skeleton was accomplished in an asymmetric manner by means of a cascade process involving hydrogenation and reductive amination (Scheme 175).^248^ Specifically, treatment of quinolines **2** bearing a pendant carbonyl-containing moiety at C8 with H_2_ in the presence of the chiral ruthenium complex Ru-(*R*,*R*)-C1 gave rise to enantiomerically enriched julolidine derivatives **345** in very good yield with excellent levels of diastereo- and enantioselectivity. The process was compatible with a variety of aryl and alkyl ketones, as well as with several alkyl and aryl substituents at the C2-position of the quinoline ring. Mechanistically, the reduction of the quinoline ring to tetrahydroquinoline **A** would initially occur and then, the intramolecular cyclization to the iminium salt **B** followed by reduction would lead to the final tricyclic products (Scheme 175).

**Scheme 176:**
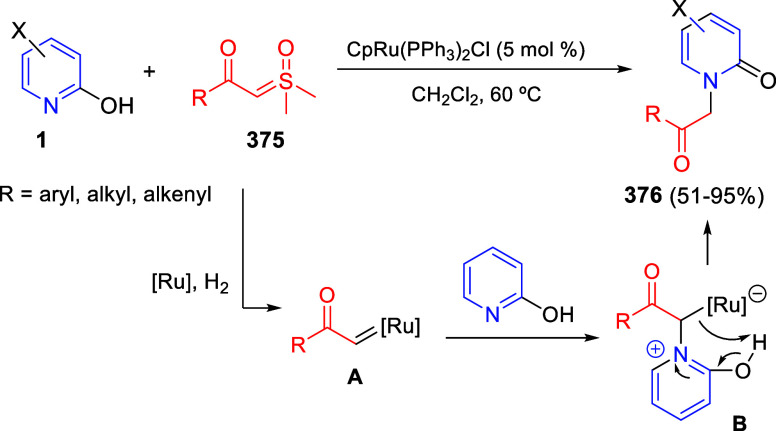
Ruthenium-Catalyzed Chemoselective N–H Bond Insertion Reactions of 2-Hydroxypyridines with Sulfoxonium Ylides

Sun and co-workers disclosed the ruthenium-catalyzed *N*-alkylation of 2-hydroxypyridines **1** with sulfoxonium ylides **375**, affording *N*-alkylated 2-pyridone derivatives **376** in good yields and excellent *N*-selectivity (Scheme 176).^249^ The use of ruthenium catalyst CpRu(PPh_3_)_2_Cl and sulfoxonium ylides as the alkylation reagents was crucial in order to achieve the N–H rather than O–H insertion reaction. Aryl, heteroaryl, and alkenyl sulfoxonium ylides were allowed in the process, as well as substituents with different electronic properties at the C3–C7-positions of the 2-hydroxypyridine ring.

Sulfoxonium ylides have been utilized as alternative to diazocompounds in carbene transfer reactions. In this case, sulfoxonium ylide **375** would react with the ruthenium catalyst to generate ruthenium carbene **A**. Subsequent reaction with 2-hydroxypyridine would form zwitterionic intermediate **B**, which would evolve to the final product by proton transfer, regenerating the ruthenium catalyst (Scheme 176).

**Scheme 177:**
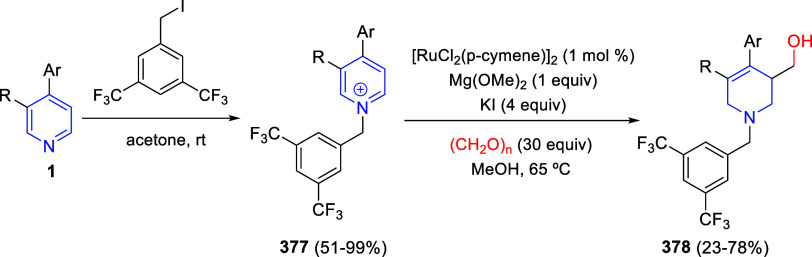
Ruthenium-Catalyzed Dearomative Hydroxymethylation of Pyridines

In 2021, Donohoe and co-workers reported a ruthenium-catalyzed dearomative hydroxymethylation of pyridines (Scheme 177).^250^ The use of the ruthenium catalyst allowed the authors to apply this type of pyridine dearomatization without the need of electron-withdrawing groups, expanding the scope of the analogous iridium-catalyzed reactions previously developed.^224,225^ In this case, the pyridine ring was activated through reaction with bis-3,5-trifluorobenzyl chloride. The resulting trifluoromethyl derivatives **377** enhanced the electron deficiency of the pyridine ring. Moreover, the use of 4-substituted pyridines ensured that initial reduction took place exclusively at the C2-position. These pyridinium salts were treated with [RuCl_2_(*p*-cymene)]_2_, Mg(OMe)_2_ as a base, KI as an additive and formaldehyde as both, hydride donor and electrophile, affording tetrahydropyridines **378** in moderated to good yields. A number of both electron-donating and electron-withdrawing substituents were well tolerated at the C4 aromatic group, as well as aryl and heteroaryl groups at the C3 of the pyridine ring. Alkyl groups in this position also provided the final products, albeit in low yields. Moreover, removal of the activating group could be achieved and the synthetic utility of the method was illustrated with the synthesis of the antidepressant drug paroxetine.

**Scheme 178:**
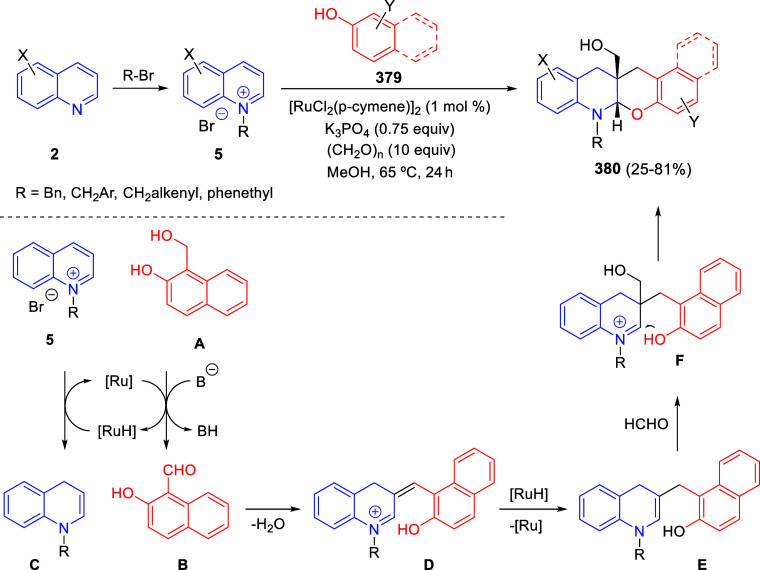
Ruthenium-Catalyzed Three-Component Reaction among Quinolinium Salts, Paraformaldehyde, and Phenols or Naphthols

A ruthenium-catalyzed three-component reaction among quinolinium salts, paraformaldehyde, and phenols or naphthols was developed by Zhang, Ci, and co-workers (Scheme 178).^251^ In this work, quinolines **2** were treated with alkyl bromides to generate quinolinium salts **5**, which were in turn treated with paraformaldehyde and phenols or naphthols **379** in the presence of the ruthenium catalyst [RuCl_2_(p-cymene)]_2_ and K_3_PO_4_ as a base, rendering polycyclic structures **380** in moderate to good yields and excellent *cis* diasteroselectivity. Substitution on the quinoline ring was evaluated at the C5-position, being tolerated both electron-donating and electron-withdrawing groups. The scope on the phenol or naphthol moiety was very wide, covering most positions with electronically different substituents with good results. In addition, several benzyl, phenethyl, and homoallyl bromides were also employed.

DFT calculations were performed to investigate the mechanistic details of this transformation. As previously commented (see Scheme 161), formaldehyde would act both as a hydrogen donor and an electrophile. Its reaction with methanol and the ruthenium catalyst would generate methyl formate and the Ru-hydride species responsible for catalysis. In a redox couple process, diol **A** (formed by reaction of naphthol with formaldehyde) would be oxidized to aldehyde **B**, while quinolinium salt **5** would be transformed into dihydroquinoline **C** by transfer hydrogenation (Scheme 178). Condensation of both molecules would render, after water loss, iminium salt **D**, which would be reduced by the [Ru-H] species to intermediate **E**. Then, the enamine attack to another equivalent of formaldehyde would give iminium salt **F** that would cyclize through intramolecular nucleophilic attack of the hydroxyl functionality.

**Scheme 179:**
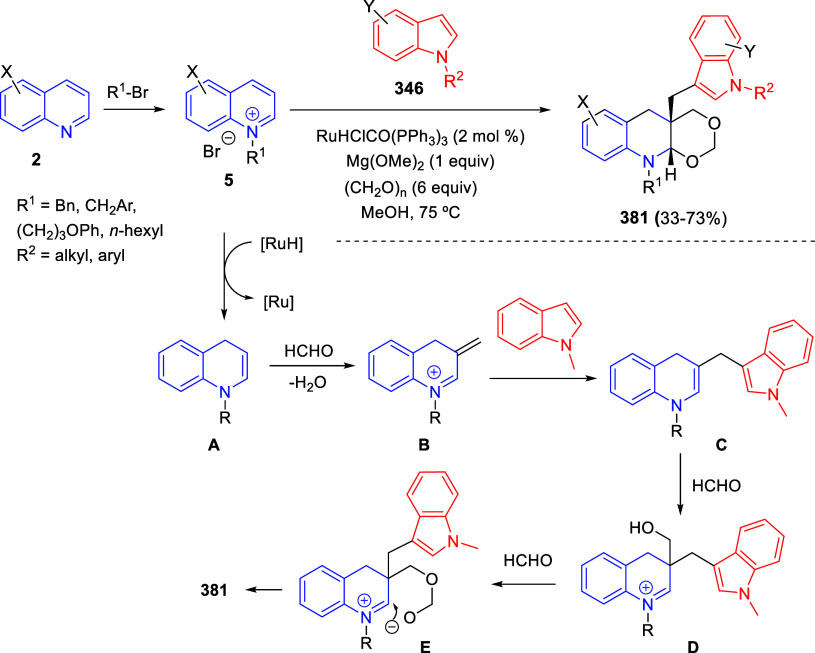
Ruthenium-Catalyzed Reductive Annulation Reaction of Quinolinium Salts with Indoles and Paraformaldehyde

An analogous ruthenium-catalyzed annulation reaction of quinolinium salts with indoles and paraformaldehyde was recently described by Zhang and Zhao (Scheme 179).^252^ Quinolinium salts **5**, in turn formed by addition of alkyl bromides to quinolines **2**, reacted with paraformaldehyde and indoles **346** in the presence of ruthenium catalyst RuHClCO(PPh_3_)_3_ and Mg(OMe)_2_ as a base to give fused tetrahydroquinolines **381** in good yields as single *cis*-diastereoisomers by means of a hydride transfer-initiated tandem protocol. Various *N*-alkyl quinolinium salts bearing a variety of functional groups were well tolerated in this transformation, as well as several indoles featuring different substitution patterns.

A plausible reaction pathway would start with a transfer hydrogenation from the ruthenium hydride to the quinolinium salt to form dihydroquinoline **A**. Enamine addition to formaldehyde and further dehydration would afford alkenyl iminium **B** (Scheme 179). Subsequent conjugate addition of indole followed by based-induced β-deprotonation would produce enamine intermediate **C**. Then, successive capture of two molecules of formaldehyde would generate iminium intermediate **E** and, finally, base-induced OH addition from the opposite side of the indoylmethylene group would render the product with exclusive *syn*-selectivity.

#### 6.1.7 Protocols Based on Other Metals

**Scheme 180:**
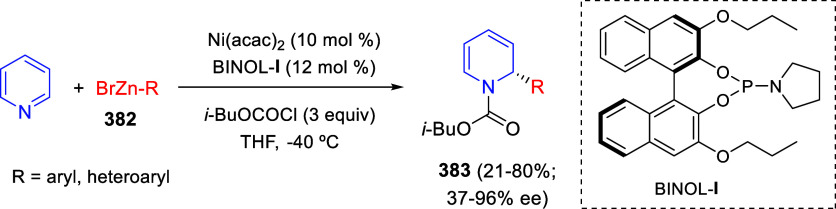
Nickel-Catalyzed Enantioselective Arylation of Pyridine

In 2016, the group of Doyle developed an enantioselective nickel-catalyzed cross coupling reaction of arylzinc reagents with pyridine (Scheme 180).^253^ This work was focused on the use of naked pyridine as the heteroarene. Its treatment with chloroformates as activating agents and aryl- or heteroarylzinc bromides **382** in the presence of Ni(acac)_2_ and BINOL-derived phosphoramidite chiral ligand BINOL-**I** provided moderate to good yields of the 2-aryl-1,2-dihydropyridine products **383** with generally good enantiocontrol. The authors found that the preparation of the arylzinc reagent played a crucial role in the reaction outcome. When they were prepared by transmetalation from aryllithium reagents, the reaction provided excellent ee values, while with arylzinc derivatives prepared by transmetalation from Grignard reagents, yield and enantioselectivity decreased dramatically, probably due to the presence of Mg(II) salts. Regarding the activating group, more sterically demanding chloroformates provided better results. Finally, derivatizations of the final products to several piperidine derivatives were achieved without no erosion in enantioselectivity.

**Scheme 181:**
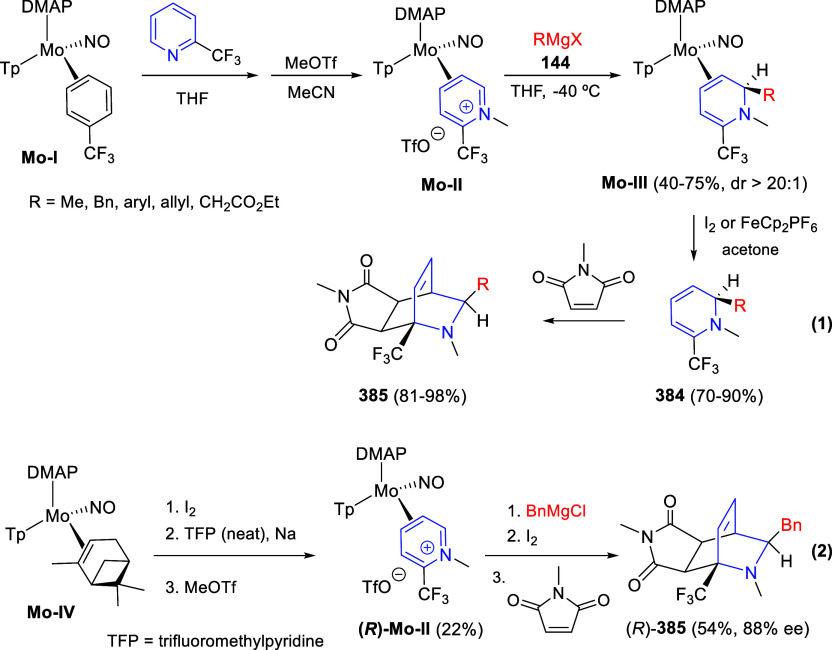
Molybdenum-Promoted Synthesis of Isoquinuclidines with Bridgehead CF_3_ Groups

Harman and co-workers demonstrated that functionalization of pyridines promoted by dihapto-coordination is possible with molybdenum complexes (Scheme 181).^254^ They found that the incorporation of a CF_3_ group into the pyridine ring would block nitrogen coordination with the metal and, at the same time, stabilize the molybdenum η^2^-pyridine complex, making possible to carry out organic transformations. Trifluoromethyl Mo-complex **Mo-II** was prepared by treatment of molybdenum complex MoTp(NO)-(DMAP)(η^2^-PhCF_3_) (**Mo-I**) with 2-trifluoromethylpyridine followed by quaternization with MeOTf. Addition of Grignard reagents **144** to this Mo-complex afforded the 1,2-addition products **Mo-III** in moderate to good yields with excellent diasteroselectivities. Free dihydropyridines **384** could be obtained by oxidative decomplexation with iodine or the iron complex FeCp_2_PF_6_ and they underwent Diels–Alder reaction with *N*-methyl maleimide to render the corresponding cycloadducts **385** in excellent yields (Scheme 181, eq 1).

The authors performed an enantioselective version of this protocol, starting from chiral complex **Mo-IV**. After formation of the chiral pyridium salt complex (*R*)-**Mo-II**, Grignard addition, metal release, and Diels–Alder reaction afforded compound (*R*)-**385** in moderate yield and 88% ee (Scheme 181, eq 2).

**Scheme 182:**
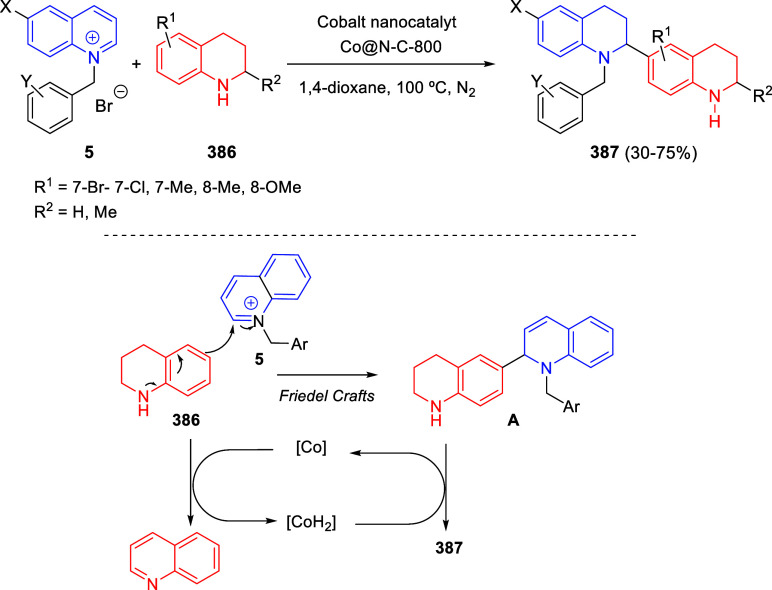
Hydrogen-Transfer Dearomative Coupling of Quinolinium Salts and Tetrahydroquinolines Catalyzed by Nitrogen-Doped Carbon Supported Nanocobalt Catalyst

Chen, Zhou, and Xie developed a nitrogen-doped carbon-supported nanocobalt catalyst (Co@N-C-800) and it was successfully applied to the hydrogen transfer dearomative coupling of quinolinium salts **5** and terahydroquinoline derivatives **386** (Scheme 182).^255^ The process led to 2-substituted *N*-alkyl-tetrahydroquinolines **387** arising from the selective coupling of the 6-position of the terahydroquinolines at the α-position of the quinolinium salts, these bearing electron-donating and electron-withdrawing groups at the 6-position, as well as at the *N*-benzyl moiety. Substituents at the C7- and C8-positions of the tetrahydroquinoline counterpart were also well tolerated in the process.

The authors performed control experiments and proposed a preliminary reaction mechanism. Initially, the tetrahydroquinoline would be dehydrogenated by the Co-catalyst, generating Co-hydride species and quinoline (Scheme 182). At the same time, tetrahydroquinoline would react in a Friedel–Crafts-type reaction with the 2-position of the quinolinium salt to render dihydroquinoline **A**, which would be reduced by the Co-hydride species to the final products, closing the catalytic cycle.

**Scheme 183:**
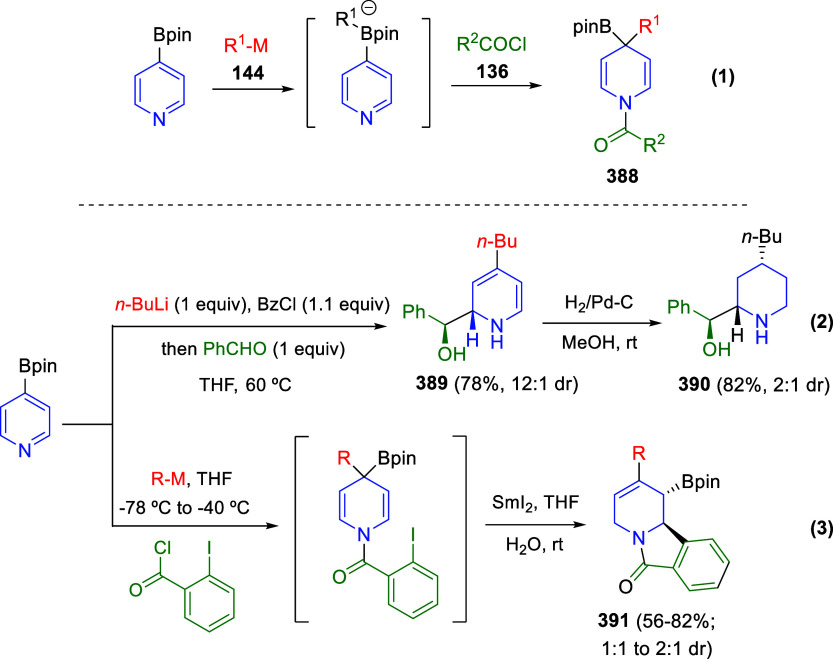
Dearomative Functionalization of 4-Pyridineboronic Acid Pinacol Ester

The reactivity of pyridineboronic acid esters with organometallic reagents in the presence of an acylating reagent was explored by Ready and co-workers (Scheme 183).^256^ Thus, upon activation of 4-pyridineboronic acid pinacol ester, the reaction with organometallic reagents **144** (RLi, RMgX or RZnX) originated dihydropyridine boronate ester intermediates that underwent 1,2-alkyl(aryl) migration, giving rise to dihydropyridine boronic esters **388**, with the creation of a new C–C bond (Scheme 183, eq 1). The authors explored the utility of these 4-boryl-4-dihydropyridine products for the synthesis of several dihydro-, tetrahydropyridine, and piperidine derivatives. For example, when *n*-Buli was added to 4-pyridineboronic acid pinacol ester, the resulting allyl boronate was added to benzaldehyde, rendering homoallylic alcohol **389** with good yield and diastereoselectivity (Scheme 183, eq 2). Then, hydrogenation led to the corresponding trisubstituted piperidine derivative **390**. On the other hand, when the addition of the organometallic reagent was performed with *ortho*-iodo benzoyl chloride, the resulting dihydropyridine intermediates were treated with samarium(II) iodide, giving rise to tricyclic tetrahydropyridines **391** in good yields (Scheme 183, eq 3). The C–C bond formation in this case was accompanied with the unexpected 1,2-boron shift and olefin transposition.

### 6.2 Organocatalytic Dearomatizations

#### 6.2.1 Anion-Binding Catalysis

**Scheme 184:**
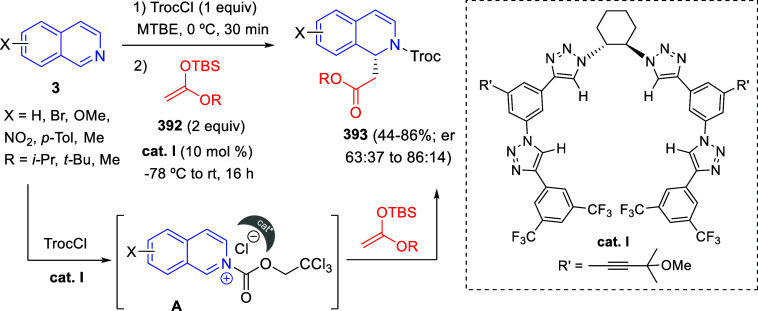
Enantioselective Reissert-Type Dearomatization of Isoquinolines with Triazole-Based Anion-Binding Catalysts

Hydrogen bond-donor type organocatalysts usually act as weak Lewis acids to activate basic sites of neutral electrophilic substrates, but they can coordinate counteranions of ionic electrophilic substrates as well, in the so-called anion-binding catalysis.^257^ On the other hand, Reissert-type reactions exploit the activation of *N*-heteroarenes with acylating or alkylating agents to perform nucleophilic additions to the resulting cationic heteroarenes, which results in their dearomatization. In this context, García Mancheño and co-workers employed this type of reaction as a model to study the development of chiral triazole-based anion-binding organocatalysts.^258^ One year later, the authors extended the protocol to substituted isoquinolines **3** as substrates, in the presence of 2,2,2-trichloroethoxycarbonyl chloride (TrocCl) as the best acylating agent (Scheme 184).^259^ The reaction of the acylated ionic intermediate **A** with silyl enol ethers **392** could be efficiently catalyzed by anion-binding catalyst **I** to render 1,2-dihydroisoquinoline derivatives **393** with moderate enantioselectivities. The catalyst C–H bonds of triazoles would be polarized enough to cooperatively perform an effective binding to the chloride counteranion.

**Scheme 185:**
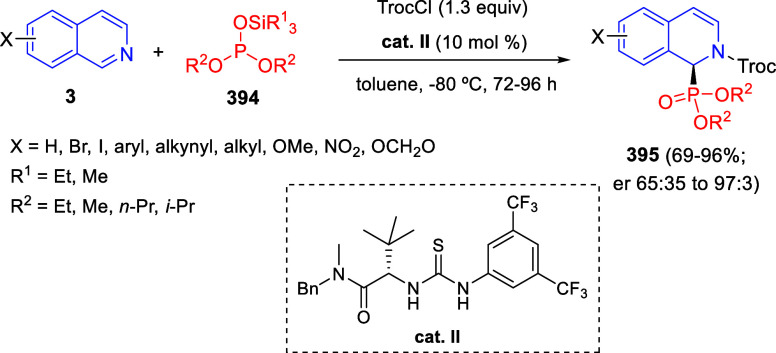
Enantioselective Dearomatization of Isoquinolines by a Thiourea-Based Anion-Binding Catalyst

The same year, Mukherjee and co-workers reported a similar enantioselective dearomatization of isoquinolines using chiral anion-binding catalysis (Scheme 185).^260^ This process made use of sylil phosphites **394** as nucleophiles and a *tert*-leucine-based thiourea derivative (**cat. II**) as the anion-binding catalyst and gave access to cyclic α-aminophosphonates **395** in moderate to excellent yields and enantiomeric ratios. This protocol was applicable to monosubstituted isoquinolines bearing substituents at nearly every position and even to disubstituted isoquinolines.

**Scheme 186:**
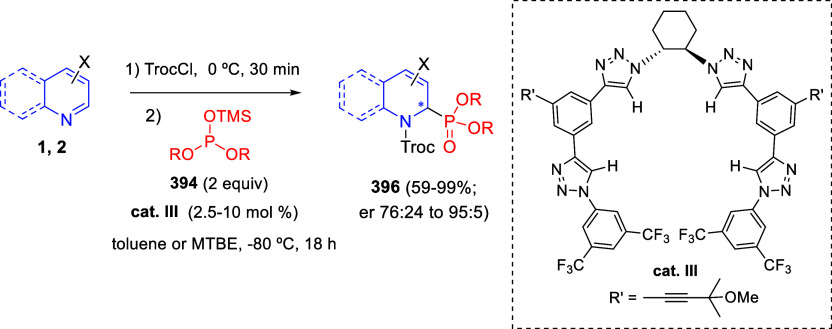
Triazole-Based Anion-Binding Catalysis for the Enantioselective Reissert-Type Dearomatization of Quinolines and Pyridines with Phosphorous Nucleophiles

In this context, García Mancheño also employed her triazole-based chiral catalyst **III** in the enantioselective nucleophilic addition of trimethylsilyl-substituted phosphites **494** to quinolines **2** and pyridines **1** to access cyclic α-amino phosponates **396** (Scheme 186).^261^ The strategy was analogous to that previously developed for isoquinoline derivatives (see Scheme 183), i.e. an anion-binding-catalyzed Reissert-type reaction with TrocCl as an acylating agent.

**Scheme 187:**
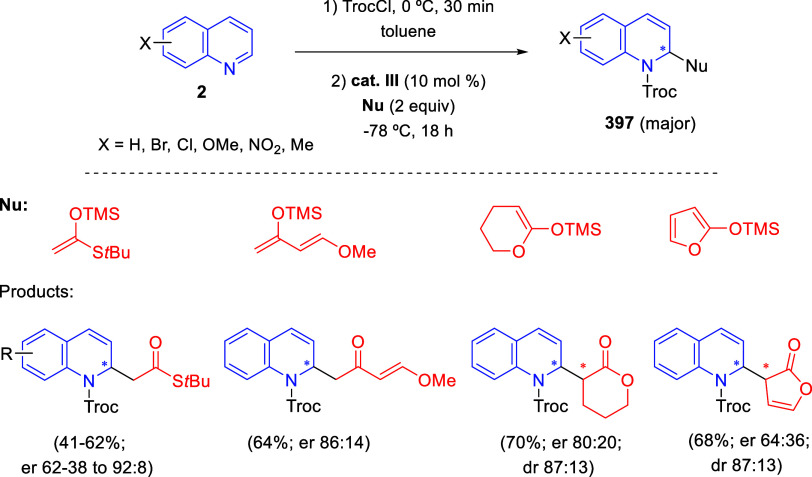
Nucleophile Screening in Triazole-Based-Catalyzed Reissert-Type Reactions

In 2019, the same group completed a systematic nucleophile screening for the Reissert-type reaction of quinolines **2** through anion-binding catalysis with the triazole-based chiral catalyst **III** (Scheme 187).^262^ They could identify several C-based nucleophiles, such as ketene thioacetals and other silyl enol ethers that reacted with good C2 regioselectivities and diastereo/enantioselectivities.

**Scheme 188:**
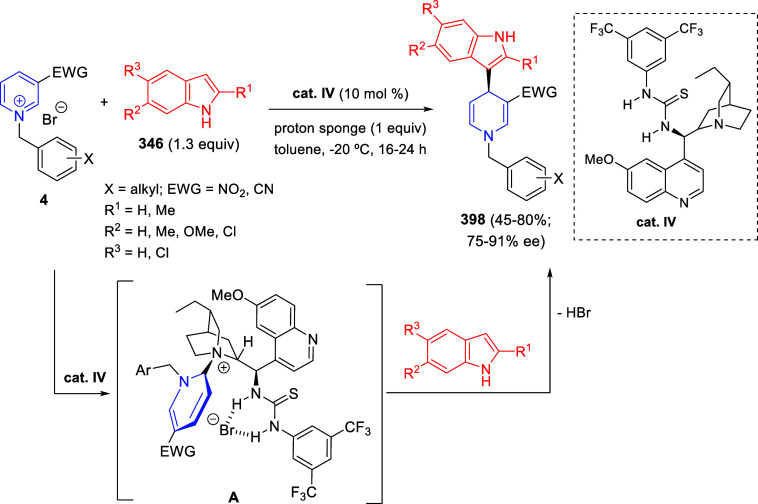
Nucleophilic Dearomatization of Pyridines by Enantioselective Addition of Indoles to Activate *N*-Benzylpyridinium Salts

Bernardi and co-workers found that bifunctional catalyst **IV**, bearing a tertiary amine moiety and a thiourea for anion-binding activation, efficiently catalyzed the enantioselective addition of indoles **346** to activated *N*-benzylpyridinium salts **4** with C4 regioselectivity, affording 1,4-dihydropyridines **398** in good yields with moderate to good enantioselectivities (Scheme 188).^263^ The electron-withdrawing group made the pyridine nucleus more electrophilic and stabilized the dihydropyridine adducts **398**. Moreover, an auxiliary base was required to neutralize the HBr formed in the reaction, and 1,8-bis(dimethylamino)naphthalene (proton sponge) was employed for this purpose. Key for the success of this transformation was the portion-wise addition of the base to avoid the formation of the isomeric *N*-alkylated byproducts **399** (Scheme 188). Regarding the scope of the process, bulky substituents at the *N*-benzyl moiety improved both reactivity and enantioselectivity. Changing the EWG of substrates from nitro to cyano group gave lower yields of products **398**. On the other hand, electron-donating groups at the 5-position of the indole counterpart gave better results than electron-withdrawing ones.

Since *N*-benzylpyridinium salts **4** are poorly soluble in toluene, the authors proposed the formation of a soluble intermediate **A**, through addition of the tertiary amine of the catalyst to the C6-position of the pyridinium ring, and the assistance of the thiourea coordinating the bromide in a bifunctional manner. Then, S_N_2′-like addition of the indole at the C4-position and rearomatization would generate the final products (Scheme 188).

**Scheme 189:**
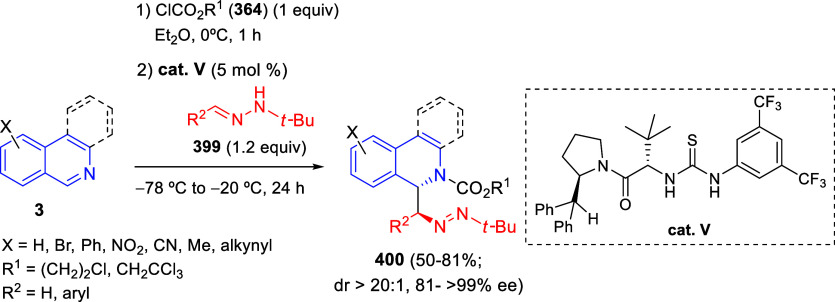
Enantio- and Diastereoselective Nucleophilic Addition of *N*-*tert*-Butylhydrazones to Isoquinolinium Ions through Anion-Binding Catalysis

More recently, the groups of Lassaletta, Fernández, Merino, and Monge took advantage of the nucleophilic character of *N*-*tert*-butyl hydrazones **399**, as masked acyl anion equivalents, in the Reissert-type dearomatization reaction of substituted isoquinolines **3** (Scheme 189).^264^ The reaction was efficiently catalyzed by the *tert*-leucine-derived thiourea catalyst **V**, affording functionalized dihydroisoquinolines **400** bearing two contiguous stereogenic centers in good to high yields with excellent diastereo- and enantiocontrol. Interestingly, the 2-chloropropionyl group at the carbamate moiety (R^1^) was tolerated, although other protecting groups such as benzyl, acetyl, benzoyl, alloc, or Cbz completely suppressed the reactivity. The process was also tolerant with aryl hydrazones **399** with diverse electronic properties as well as with the simplest formaldehyde-derived hydrazine. Regarding the isoquinoline counterpart, electron-deficient substrates improved yields and ee values, while crowded substrates such as 7-, 8-, or 1-substituted isoquinolines **3** gave lower enantiomeric excess, poor conversion, or no reaction at all. Similarly, pyridinium salts did not react under the optimized reaction conditions.

The authors performed anion exchange experiments and computational studies that supported the importance of the chloride anion as a template for the formation of a highly ordered transition state involving the catalyst, the hydrazone, and the isoquinolinium cation. This structure, stabilized by cooperative noncovalent interactions, would explain the excellent stereocontrol achieved in this transformation.

#### 6.2.2 Chiral Phosphoric Acid Catalysis

**Scheme 190:**
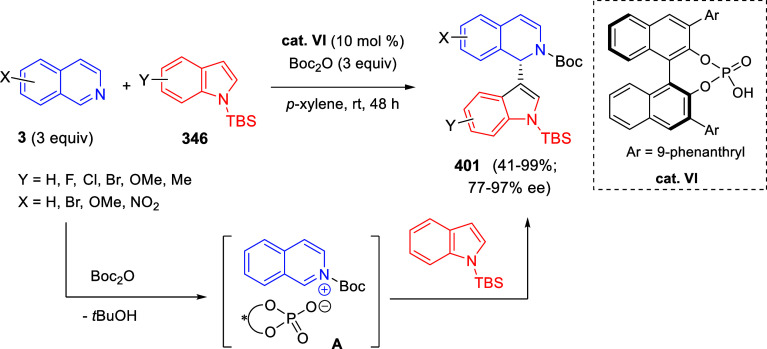
Enantioselective Dearomative Arylation of Isoquinolines

In 2016, Wang and co-workers described a phosphoric acid-catalyzed enantioselective dearomative arylation of isoquinolines **3** with indoles **346** (Scheme 190).^265^ The reaction allowed the synthesis of α-indole dihydroisoquinolines **401** in moderate to excellent yields, with high enantiocontrol, when the BINOL-derived phosphoric acid **cat. VI** was employed. This enantioselective arylation was based on the ion-pair interaction between the chiral anion catalyst and the *N*-Boc-activated isoquinolinium substrate (intermediate **A**). Both indoles **346** and isoquinolines **3** bearing electronically different substituents were compatible with the transformation.

**Scheme 191:**
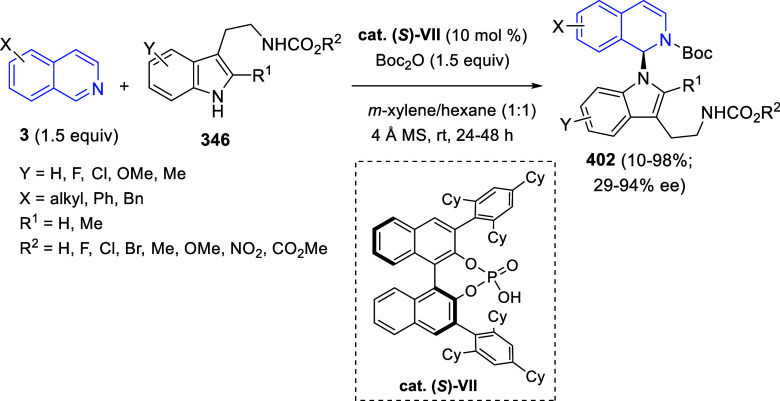
Chiral Phosphoric Acid-Catalyzed Dearomatization Reaction of Isoquinolines with Indole Derivatives

Inspired by this work, You and co-workers developed a chemoselective N–H functionalization of indole derivatives **346** through the Reissert-type dearomative reaction of isoquinolines **3** (Scheme 191).^266^ The best results were obtained with the BINOL-phosphoric acid catalyst (*S*)-**VII**, which was supposed to form ion-pair intermediates. The use of N-H free indoles substituted at the 3-position made possible the chemoselective nucleophilic addition of the indolyl nitrogen to the *N*-Boc activated quinolinium intermediates to furnish 1,2-dihydroisoquinolines **402**. The reaction generally worked with good yields and moderate to good enantioselectivities but 7- or 8-substituted isoquinolines displayed lower yields (10–40%) and ee values (29–50% ee) due to steric issues. The authors also showed the compatibility of this method to gram-scale, as well as the synthetic utility of the products through further derivatizations.

**Scheme 192:**
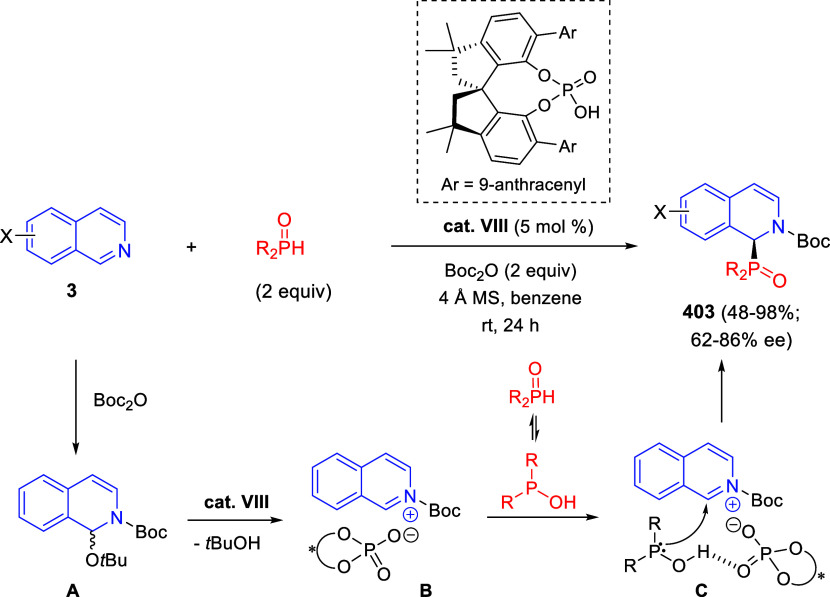
Phosphoric Acid-Catalyzed Enantioselective Dearomative Phosphonation of Isoquinolines

Very recently, Guo and Gao found that tetramethyl SPINOL-derived phosphoric acid **cat. VIII** was the most effective catalyst for the enantioselective phosphonation of isoquinolines **3** (Scheme 192).^267^ The reaction was performed with both dimethyl phosphonate and diphenylphosphine oxide as nucleophiles, and gave 1,2-dihydroisoquinolines **403**. Isoquinolines bearing electron-donating or weakly electron withdrawing groups led to the corresponding dearomatized products in good yields with moderate to good enantioselectivities. The authors proposed a plausible mechanism starting with formation of intermediate **A** by reaction of the starting isoquinolines with (Boc)_2_O. Then, catalyst **VIII** would facilitate the elimination of *tert*-butanol to form the ion-pair intermediate **B** (Scheme 192). Tautomerization of the phosphorus nucleophiles would permit the hydrogen-bonding with the catalyst in intermediate **C** and, finally, nucleophilic addition would provide dihydroquinolines **403** and regenerate the catalyst.

#### 6.2.3 Chiral *N*-Heterocyclic Carbene (NHC) Catalysis

**Scheme 193:**
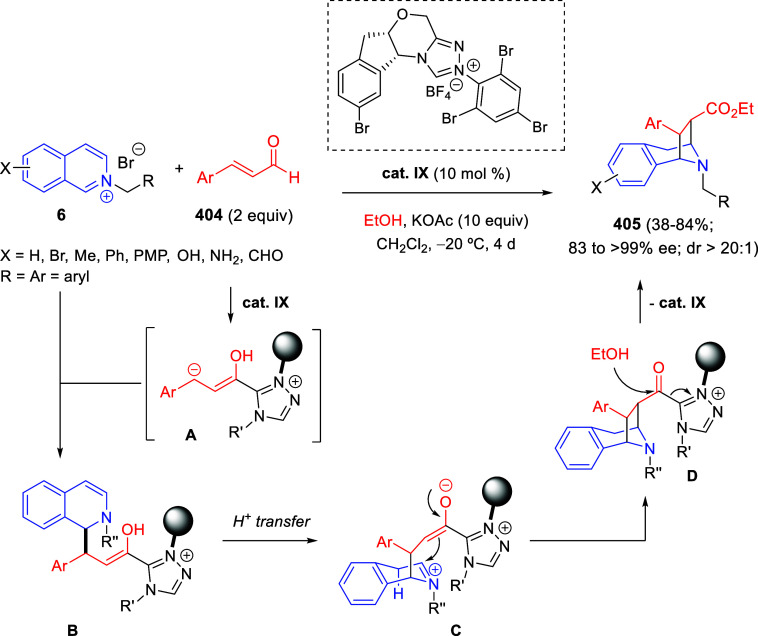
Construction of Tropane Derivatives by Organocatalytic Asymmetric Dearomatization of Isoquinolines

Tan and co-workers employed chiral *N*-heterocyclic carbenes (NHCs) to construct tropane derivatives through an isoquinoline dearomatization process (Scheme 193).^268^ The addition of the chiral triazolium-salt catalyst **VIII** to aromatic enals **404** formed homoenolate intermediates **A**, which acted as dinucleophiles in a double Mannich reaction with isoquinolinium salts **6** to afford bicyclic derivatives **405** with four contiguous stereocenters as single diastereoisomers, in moderate to good yields with excellent enantioselectivities. The reaction was compatible with the presence of many different functional groups in the aromatic rings of both substrates. Mechanistically, the first Mannich addition of homoenolate **A** to the C1-position of the substrate would form intermediate **B**. An internal proton transfer (**C**), followed by the intramolecular second Mannich addition, would deliver intermediate **D**, which would react with ethanol to generate the substituted tropane derivatives **405** and the catalytic species (Scheme 193).

**Scheme 194:**
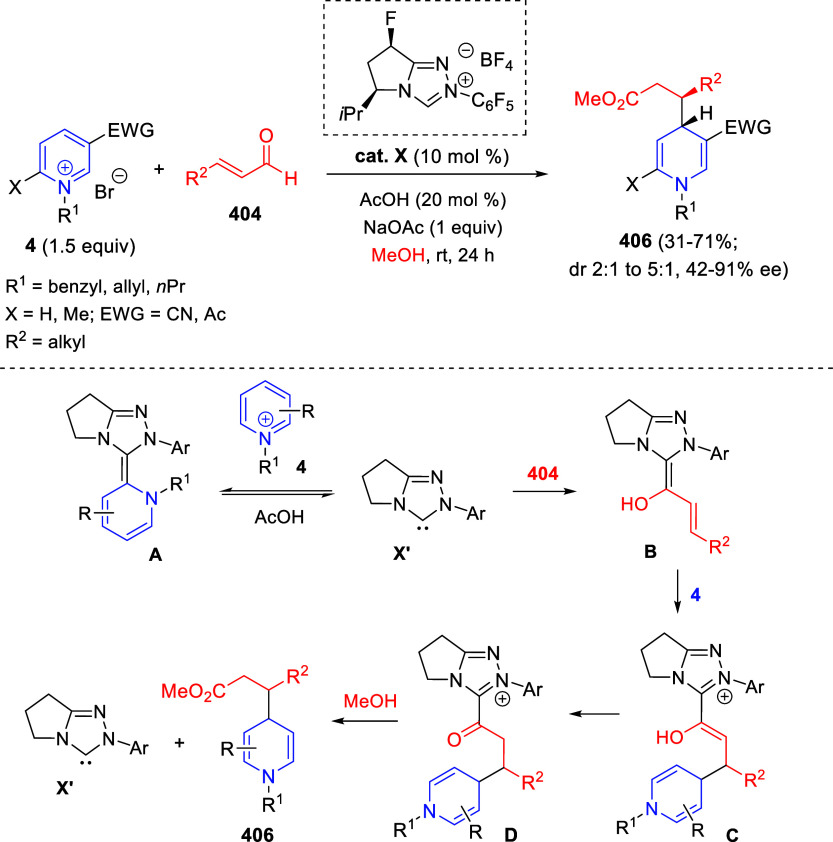
Enantioselective NHC-Catalyzed Nucleophilic Dearomatization of Alkyl Pyridiniums

Rovis and co-workers found that homoenolate intermediates derived from enals were added preferentially to the C4-position of alkyl pyridinium substrates **4** to generate 1,4-dihydropyridines **406** with high enantioselectivity (Scheme 194).^269^ These homoenolates were formed by the addition of the chiral triazole-based carbene catalyst **X** to aliphatic enals **404**. However, the use of phenyl or styryl enals afforded 1:1 mixtures of C2 and C4 addition products without diastereo- or enantiocontrol. Pyridinium substrates **4** had to be functionalized at the C3-position with an electron withdrawing group, while a broad scope of *N*-alkyl groups was tolerated in the process. The chemical yields were improved by exclusion of oxygen and addition of acetic acid (20 mol %), which helped to prevent an off-cycle catalyst-pyridinium adduct trap by formation of intermediate **A** (Scheme 194). After formation of homoenolate **B**, it would add to *N*-alkyl pyridiniums **4** through its β-position to deliver enol azolium **C**, which would tautomerize to keto azolium **D**. Finally, the addition of methanol would lead to the final product and catalyst turnover.

**Scheme 195:**
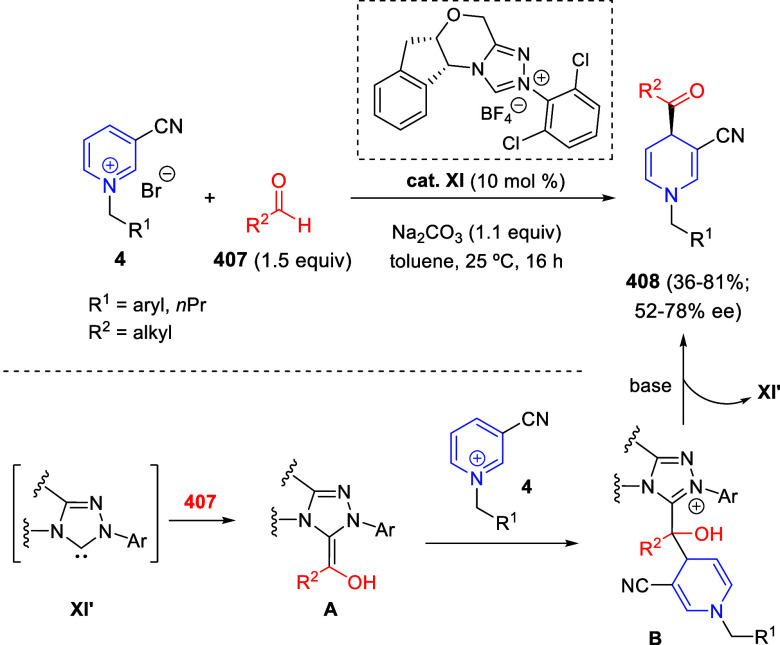
Enantioselective Dearomatization of Alkylpyridiniums by NHC-Catalyzed Nucleophilic Acylation

Complementary to the previous report, Massi and co-workers described a completely regioselective C4-acylation of activated pyridinium salts **4** with aliphatic aldehydes **407** (Scheme 195).^270^ The process was catalyzed by the chiral triazolium salt **XI** and allowed the synthesis of enantioenriched 1,4-dihydropyridines **408** in moderate to good yields. The authors postulated a mechanism starting from NHC **XI′** (generated by deprotonation of triazolium salt **XI**), which would be added to aldehydes **407**, forming Breslow intermediate **A**. Addition of this intermediate to the C4-position of pyridinium salt **4** would deliver adduct **B** and final deprotonation would form the 1,4-dihydropyridines, regenerating the catalytic species (Scheme 195).

#### 6.2.4 Protocols Based on Other Organocatalysts

**Scheme 196:**
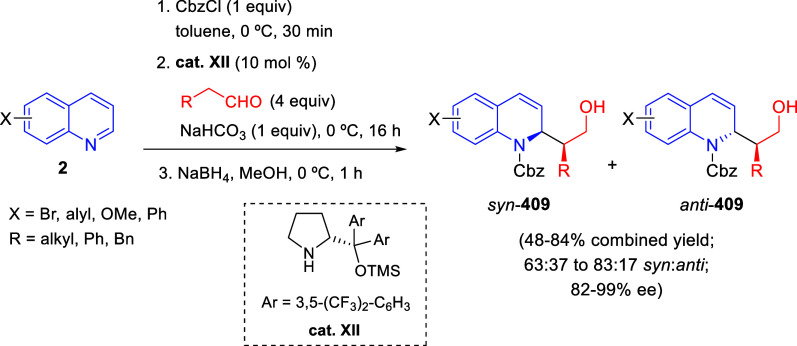
Organocatalytic Stereoselective Addition of Aldehydes to Acylquinolinium Ions

In 2016, Cozzi and co-workers reported an organocatalytic stereoselective addition of aldehydes to acylquinolinium ions employing the secondary amine catalyst **XII** (Scheme 196).^271^ In this work, aliphatic aldehydes were activated via enamine catalysis toward its addition to the C2-position of acylquinolinium derivatives, formed by reaction of quinolines **2** with benzyl chloroformate. The resulting 1,2 dihydroquinolines were further reduced with NaBH_4_ to isolate the corresponding diastereoisomeric alcohols **409** as major products in moderate to good yields with high enantioselectivities for both *syn* and *anti* diastereoisomers and moderate diastereoselectivities.

**Scheme 197:**
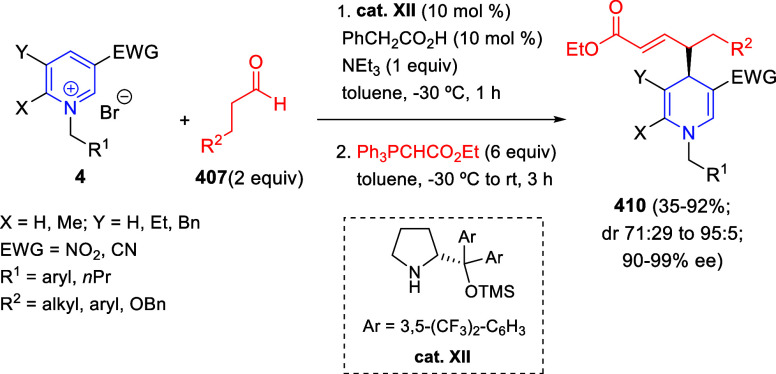
Organocatalytic Stereoselective Addition of Aldehydes to *N*-Alkylpyridinium Salts

Enamine catalysis was also employed for the enantioselective addition of aldehydes **407** to the C4-position of *N*-alkylpyridinium ions **4** (Scheme 197).^272^ The Hayashi–Jørgensen catalyst **XII** efficiently catalyzed the reaction, although an erosion of diastereo- and enantioselectivity was observed with time, probably due to the use of an equivalent of triethylamine, necessary to neutralize the HBr formed in the reaction. However, the use of phenylacetic acid as a cocatalyst improved the robustness of the process. The authors transformed the aldehyde reaction products through Wittig olefination, isolating the less labile α,β-unsaturated esters **410**.

**Scheme 198:**
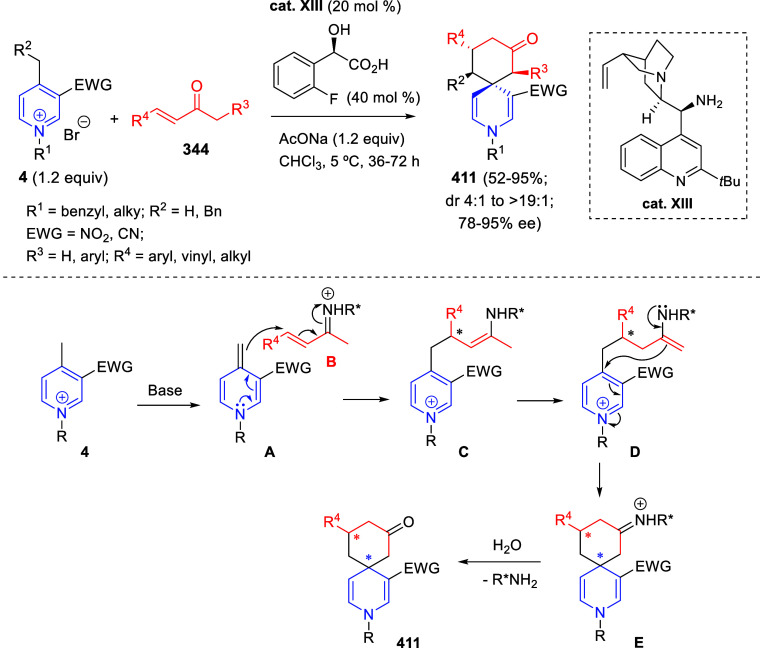
Asymmetric Dearomative formal (4 + 2) Cycloadditions of *N*,4-Dialkylpyridinium Salts and Enones

Chen and co-workers developed an enantioseletive formal (4 + 2) cycloaddition reaction of *N*,4-dialkylpyridinium salts **4** and enones **344** by means of a cascade iminium ion/enamine catalysis with a Cinchona-derived amine **XIII**, mandelic acid as a cocatalyst, and sodium acetate (Scheme 198).^273^ This reaction allowed the authors to construct a series of azaspiro[5.5]undecane derivatives **411** with multiple functionalities with moderate to excellent stereoselectivity. The cascade reaction was believed to start with the deprotonation of *N*,4-dialkylpyridinium salt **4** to generate the dienamine-type intermediate **A**, and subsequent Michael-type addition of this intermediate to the enone, activated by the chiral catalyst as iminium ion **B**. The resulting enamine intermediate **C** would tautomerize to enamine **D** before its intramolecular C4 addition to the pyridinium ring, delivering final adduct **411** after iminium hydrolysis of intermediate **E** (Scheme 198).

**Scheme 199:**
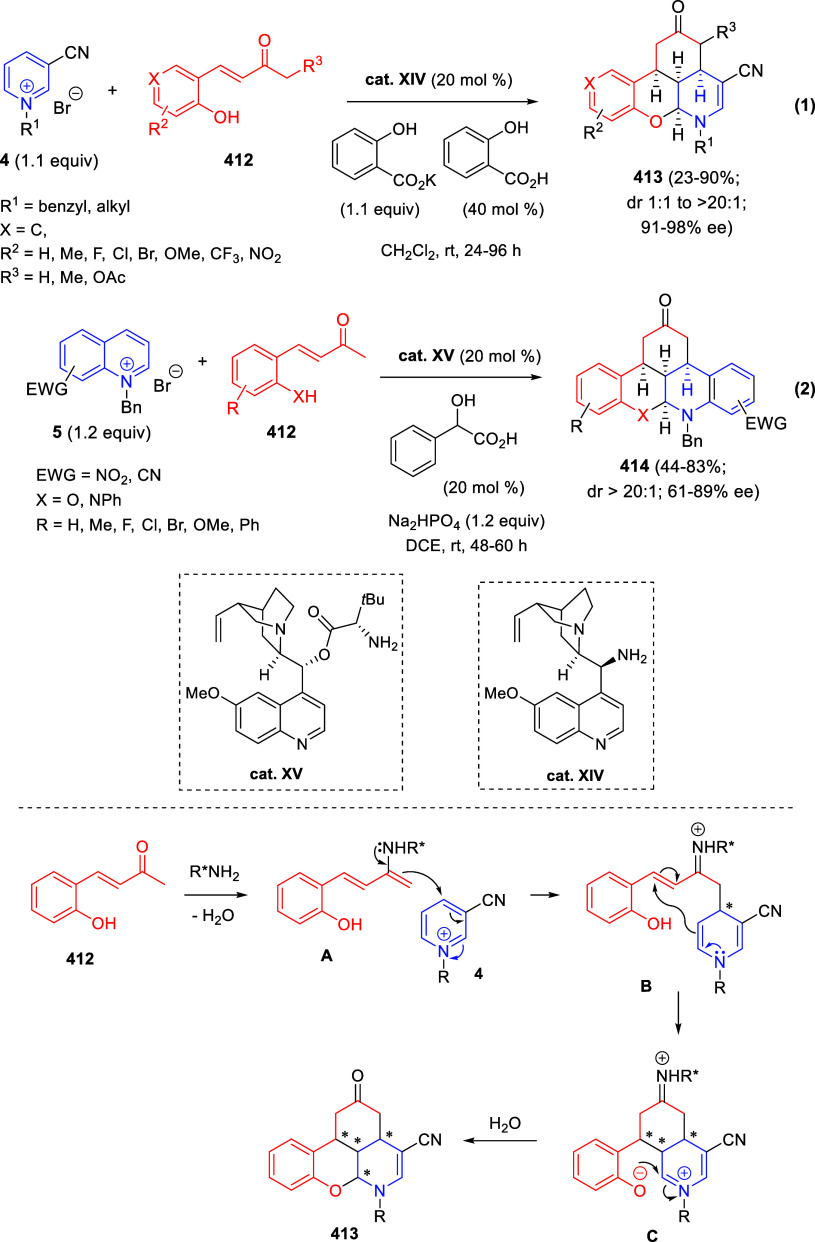
Asymmetric Cascade Multiple Functionalization of Pyridinium Salts with *o*-Hydroxybenzylidene Acetones

The same group also developed an asymmetric dearomative cascade reaction of *N*-alkylpyridinium salts **4** with *o*-hydroxybenzylidene acetones **412**, employing quinine-derived primary amine catalyst **XIV**, salicylic acid as a cocatalyst, and potassium salicylate (Scheme 199).^274^ This methodology allowed the access to fused polyheterocycles **413**, through tandem enamine/iminium ion activation, in moderate to good yields with high levels of stereocontrol (Scheme 199, eq 1). The *N*-alkylpyridinium salts **4** had to be substituted with a 3-cyano group to perform the reaction successfully, while enones **412** with a substituent at the α′-position (R^3^) showed lower diastereoselectivity and those *ortho*-substituted to the hydroxyl group (R^2^) gave the final products in low yields.

The reaction was extended to *N*-benzylquinolinium salts **5** bearing an electron-withdrawing group at the 5-position, this time in the presence of primary amine catalyst **XV** and racemic mandelic acid as the catalytic system (Scheme 199, eq 2). This reaction tolerated *o*-amino enone **412** (X = NHPh) as the substrate, although the corresponding product was obtained with modest enantioselectivity (61% ee).

A plausible mechanism for this transformation could start with the intermolecular C4 addition of enamine **A** to the pyridinium substrate **4**, followed by intramolecular Michael-type addition of the dearomatized enamine intermediate **B** to the α,β-unsaturated iminium cation (Scheme 199). Final intramolecular aminal formation on intermediate **C** and release of the organocatalyst would deliver the fused polyheterocyclic products **413**.

**Scheme 200:**
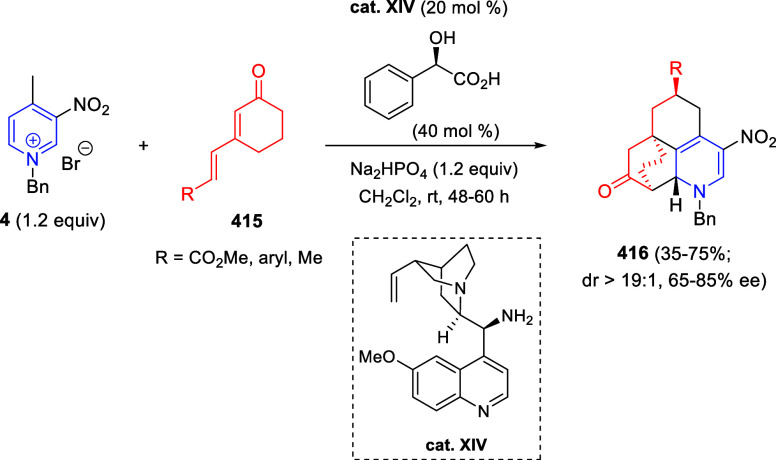
Asymmetric Cascade Multiple Functionalization of Pyridinium Salts with Cyclic 2,4-Dienones

This methodology was further extended in a cascade assembly of *N*-benzyl-4-methylpyridinium salt and cyclic 2,4-dienones **415** to generate bridged structures **416** in fair yields and moderate to good levels of enantiocontrol (Scheme 200).^271^ The reaction proceeded under chiral amine catalysis, through repetitive dearomatization/aromatization via a domino Michael/Michael/Mannich addition sequence.

**Scheme 201:**
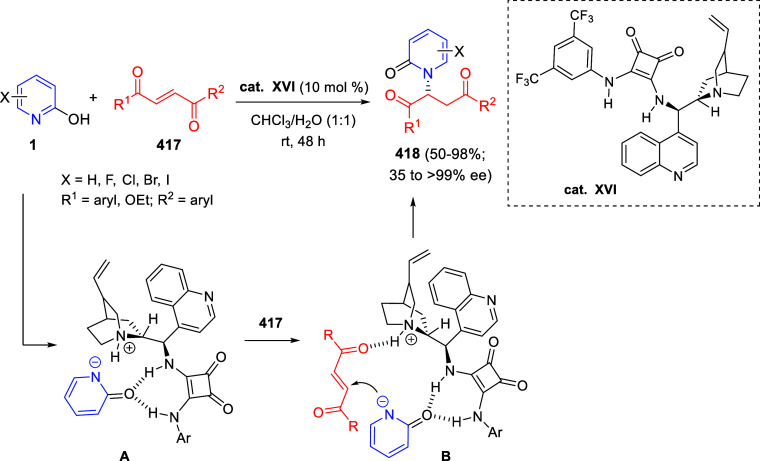
Enantioselective Michael Addition of 2-Hydroxypyridines to α,β-Unsaturated 1,4-Dicarbonyl Compounds

Squaramide-catalyst **XVI**, prepared from (+)-cinchonine, was employed by Hou and co-workers as bifunctional catalyst for the enantioselective aza-Michael addition of 2-hydroxypyridines **1** to α,β-unsaturated 1,4-dicarbonyl compounds **417** (Scheme 201).^275^ The reaction afforded *N*-substituted 2-pyridones **418** in generally good yields and enantioselectivities. The authors found that 3-halo- and 5-halo-2-hydroxypyridines were better Michael donors than their 4-halogenated or nonhalogenated analogues. 6-Chloro-2-hydroxypyridine did not react for steric reasons. Michael acceptors included aromatic γ-diketones and γ-ketoesters, but poor results were obtained with aromatic rings bearing electron-donating substituents.

DFT calculations were performed in order to propose a plausible reaction mechanism. Accordingly, 2-hydroxypyridines would form an ion-pair with the protonated tertiary amine of the catalyst, assisted by squaramide coordination in intermediate **A**. Afterward, the tertiary amine would activate the Michael acceptor while squaramide still coordinates the Michael donor to facilitate the enantioselective aza-Michael addition in intermediate **B** (Scheme 201).

**Scheme 202:**
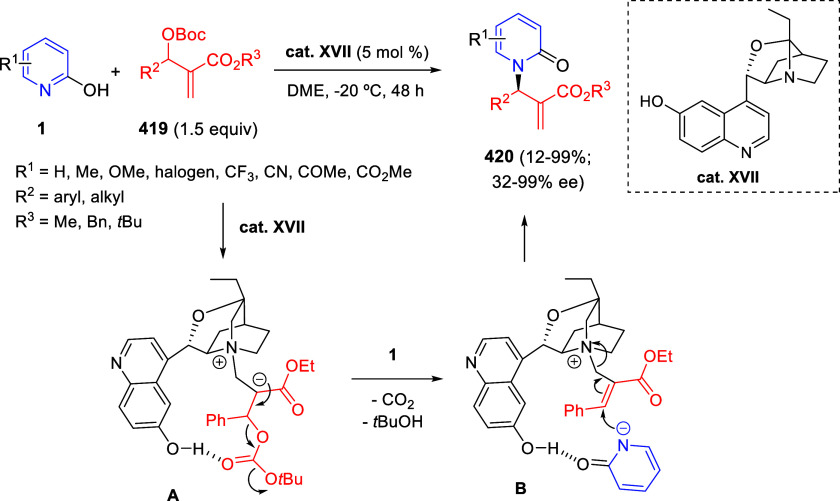
Bifunctional Lewis Base-Catalyzed Asymmetric *N*-Allylic Alkylation of 2-Hydroxypyridines

Very recently, Ye, Luo and co-workers reported a chiral Lewis base-catalyzed enantioselective *N*-allylic alkylation of 2-hydroxypyridines **1** with Morita–Baylis–Hillman (MBH) carbonates **419** (Scheme 202).^276^ The reaction was catalyzed by β-isocinchonine **XVII** and delivered the corresponding *N*-alkylated 2-pyridones **420** in moderate to excellent yields with good to excellent enantioselectivities, regardless the electronic properties of the pyridine substituents. Alkyl (R^2^) carbonates **419** gave poor results in terms of yield and enantioselectivity in comparison with aryl carbonates.

Experimental and computational studies revealed that the hydrogen bond interaction between the chiral Lewis base catalyst and 2-hydroxypyridines plays a crucial role in this reaction. The authors proposed that the tertiary amine of the catalyst would be added to the Michael acceptor of MBH carbonates to form intermediate **A**, while the quinoline hydroxide would be activating the carbonate by hydrogen bonding to facilitate the elimination of CO_2_ and *tert*-butoxide (Scheme 202). In this way, the base necessary to deprotonate the 2-hydroxypyridine would be generated *in situ* and a new Michael acceptor would be formed. The quinoline hydroxide of the catalyst would be essential to coordinate the deprotonated 2-hydroxypyridine substrate, assisting the enantioselective aza-Michael addition in intermediate **B** to generate *N*-allylic alkylated products after the catalyst removal.

**Scheme 203:**
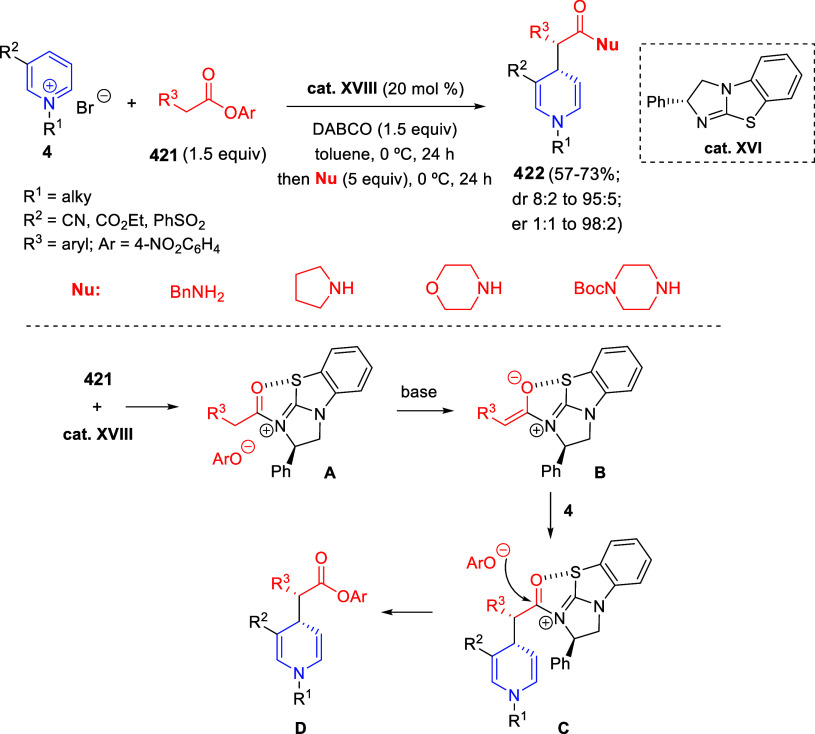
Catalytic Enantioselective Synthesis of 1,4-Dihydropyridines via the Addition of C(1)-Ammonium Enolates to Pyridinium Salts

In 2021, Smith and co-workers disclosed a regio- and stereoselective addition of C(1) ammonium enolates, generated *in situ* from aryl esters and an isothiourea catalyst, to pyridinium salts **4** bearing an electron withdrawing substituent at the 3-position (Scheme 203).^277^ They found that isothiourea catalyst **XVIII** reacted with aryl esters **421** to form *N*-acyl ammonium salts **A**, which, in the presence of DABCO as a base, delivered C(1)-ammonium enolate intermediates **B**. These enolates were added to the C4-position of *N*-alkylpyridinium salts **4** in a diastereo- and enantioselective manner, to render *N*-acyl ammonium salt intermediates **C** which, after aryloxide addition, formed aryl ester adducts **D** with concomitant regeneration of the catalyst. In order to improve the stability of aryl ester adducts **D**, they were derivatized to the corresponding isolable amides **422** by addition of amines as nucleophiles.

*N*-Alkylpyridinium salts **4** bearing an electron withdrawing substituent at the 3-position such as the cyano or 3-phenylsulfonyl groups gave excellent results, while the ethyl ester substituent delivered the product as a racemate. Similarly, aryl acetic *p*-nitrophenyl esters **421** bearing an electron-withdrawing 4-trifluoromethylphenyl substituent (R^3^) gave the corresponding 1,4-dihydropyridine product in good yield and excellent diastereoselectivity although as a racemic mixture, probably due to a base-promoted background reaction without the participation of the catalyst. Amine nucleophiles were shown to be generally applicable (Scheme 203).

### 6.3 Base-Mediated Dearomatizations

**Scheme 204:**
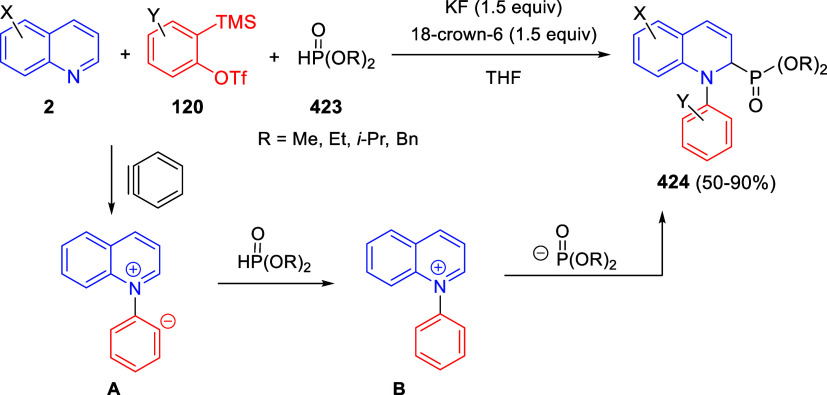
Aryne-Induced Dearomative Phosphonylation of Quinolines

In 2016, He and Dai reported an aryne-induced dearomative phosphonylation of quinolines **2** by means of a multicomponent reaction with aryne precursors **120** and dialkyl phosphonates **423** in the presence of KF and 18-crown-6 to furnish phosphonylated dihydroquinolines **424** (Scheme 204).^278^ Arynes are reactive intermediates, and they would be trapped by the quinoline nucleophiles to form zwitterionic species **A** that would be protonated. Then, nucleophilic addition of the phosphonate to the iminium ion **B** would render the final products **424**. Quinolines bearing both electron-donating and electron-withdrawing groups underwent the multicomponent reaction efficiently, as well as isoquinolines. Regarding the aryne precursor, electron-donating substituents were tested at different positions, affording the corresponding products in good yields. Finally, different dialkyl phosphites performed successfully, while diphenyl phosphite could not undergo the reaction.

**Scheme 205:**
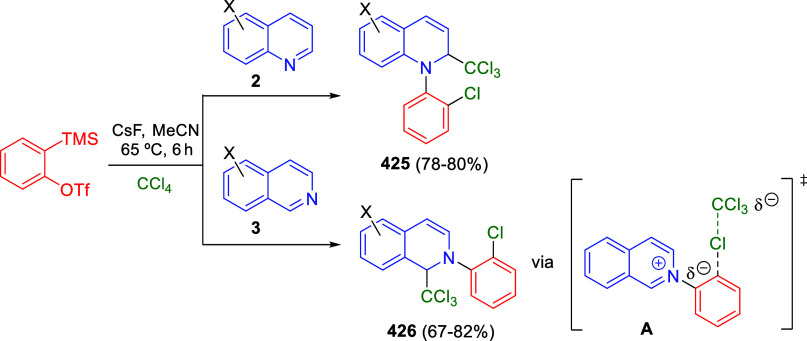
Formal Insertion of *N*-Heteroarenes and Arynes into the C–Cl Bond of Carbon Tetrachloride

Another three-component aryne-induced dearomatization reaction of quinolines and isoquinolines involved a formal insertion of the C=N bond into the C–Cl bond of carbon tetrachloride (Scheme 205).^279^ Thus, the reaction of quinolines **2** or isoquinolines **3** with 2-(trimethylsilyl)phenyl triflate as the benzyne source and CCl_4_ in the presence of CsF furnished chlorinated dihydroquinolines **425** and dihydroisoquinolines **426** in good yields, with incorporation of a chlorine atom at the benzyne precursor. Although the authors performed DFT calculations of the reaction with imines as the source of C=N bonds, the proposed mechanism would initiate by nucleophilic addition of the heteroarene to the benzyne, generating an aryl anion intermediate that would react through an S_N_2-type process with CCl_4_, generating an ion pair consisting of an iminium cation and a trichloromethyl anion (transition state **A**) that would evolve through nucleophilic addition of the trichloromethyl anion to the 1-position of the isoquinoline ring, leading to the final products (Scheme 205).

**Scheme 206:**
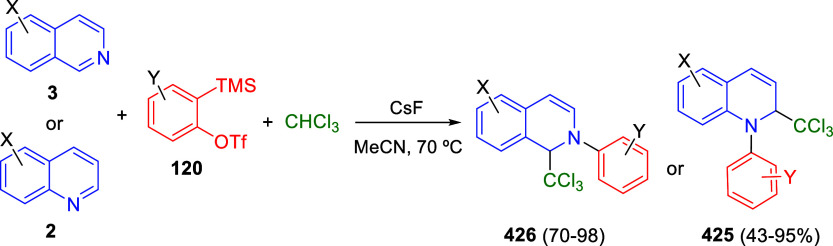
Aryne Triggered Dearomatization Reaction of Isoquinolines and Quinolines with Chloroform

Almost simultaneously, Tan and co-workers reported an analogous dearomatization reaction of isoquinolines and quinolines with chloroform through *in situ* electrophilic aryne activation and nucleophilic addition of a trichloromethyl anion (Scheme 206).^280^ Quinolines **2** and isoquinolines **3** reacted with benzyne precursors **120** and chloroform to render 1-trichloromethyl *N*-aryl dihydroisoquinolines **426** and 2-trichloromethyl *N*-aryl dihydroquinolines **425** in good yields. Aryne precursors incorporating various functional groups were well tolerated, although asymmetric benzynes provided mixtures of diastereoisomers. Regarding the starting isoquinolines, several groups at different positions were also well tolerated, including substrates containing bromide and iodide substituents, often incompatible with metal-mediated reactions. The dearomatization reaction was also efficient for quinoline derivatives at the C2-position exclusively, even in the case of a 2-methyl-substituted quinoline, which gave the corresponding product with a quaternary stereocenter in reasonable yield.

**Scheme 207:**
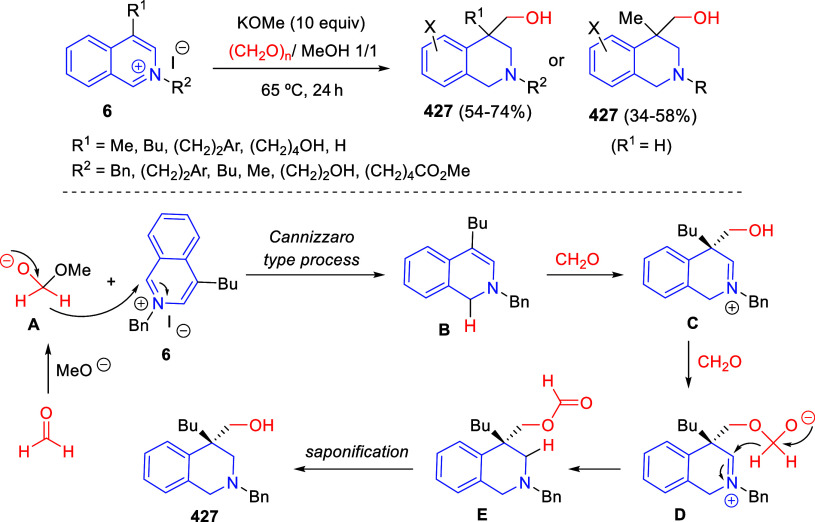
Metal-Free Reductive Hydroxymethylation of Isoquinolines

In 2019 Donohoe and co-workers described a metal-free reductive hydroxymethylation reaction of isoquinolinium salts **6** by reaction with potassium methoxide and formaldehyde in methanol, affording tetrahydroisoquinolines **427** bearing C4 quaternary stereocenters in good yields (Scheme 207).^281^ A variety of alkyl substituents were tolerated at the C4-position of the isoquinoline ring (R^1^); however, the process failed with aromatic groups. Similarly, several alkyl substituents were allowed at the heterocyclic nitrogen (R^2^). When the reaction was performed with C4-unsubstituted isoquinolinium salts (R^1^ = H), the isolated products showed a quaternary stereocenter bearing a methyl group at the C4-position (Scheme 207). This tandem methylation-hydroxymethylation process led to the formation of 2 new C–C bonds and it was evaluated with isoquinolines bearing electron-donating substituents, providing the final products in moderate yields.

Deuterium labeling studies and control experiments led the authors propose that the reaction would be initiated by the addition of methoxide to formaldehyde, rendering hemiacetal **A** that would deliver hydride to the C1-position of the isoquinolinium salt in a Cannizzaro-type reduction (Scheme 207). The newly formed enamine **B** would attack formaldehyde to form zwtterionic intermediate **C**, which in turn would react with another equivalent of formaldehyde to render hemiacetal **D**, which would transfer hydride in an intramolecular and diastereoselective fashion, through an Evans–Tishchenko process. Final *in situ* cleavage of formate **E** would render the final products **427**.

**Scheme 208:**
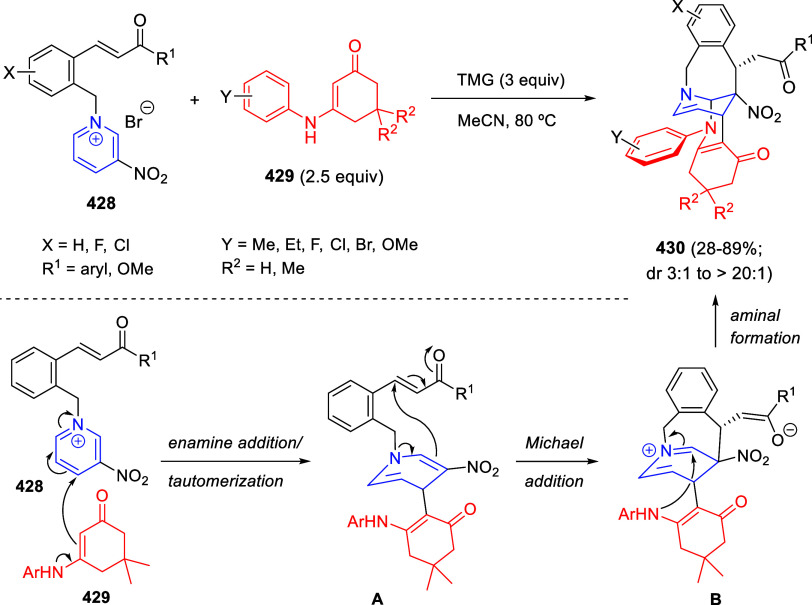
Dearomatization of Chalcone-Based Pyridinium Salts to Access Bibridged Benzoazepines

Chalcone-based pyridinium salts **428**, with multiple reactive sites, were employed by Wang and co-workers in a diastereoselective dearomatization reaction with enaminones **429** acting as bis-nucleophiles, in the presence of 1,1,3,3-tetramethylguanidine (TMG), to prepare bibridged benzoazepines **430** in moderated to good yields (Scheme 208).^282^ The reaction consisted of three sequential nucleophilic additions, namely a first intermolecular addition of the enaminone to the 4-position of the pyridinium ring followed by imine-enamine tautomerization, which would give the dearomatized intermediate **A**. Second, an intramolecular Michael addition of the nitroenamine to the chalcone would generate intermediate **B**, with an iminium moiety and, finally, the enaminone nitrogen would add to the iminium cation to form aminals **430** bearing two bridged rings and four contiguous stereocenters (Scheme 208). Different substituents on the phenyl ring (X) and the carbonyl functional group (R^1^) of chalcone-based pyridinium salts **428** could be well accommodated in this transformation, with complete regio- and diastereomeric control. The reaction was also tolerant to enaminones **429** with different substitution patterns on the phenyl and cyclohexenone rings.

**Scheme 209:**
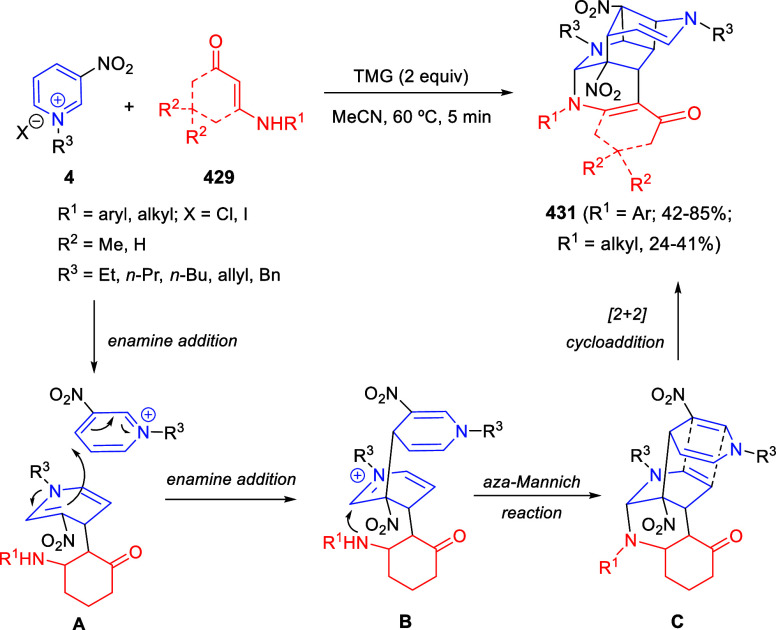
Base-Promoted Dearomative Multifunctionalization of Pyridinium Salts

Soon after, the same group reported a related one-pot multicomponent dearomatization of *N*-alkyl activated azaarenes, mediated by guanidines (Scheme 209).^283^ The authors found that 3-nitropyridinium salts **4** reacted with enaminones **429** in the presence of tetramethyl guanidine (TMG) as a base to render bibridged azaheterocycles **431** in good yields with excellent diasteroselectivities. The process comprised the participation of 2 equiv of nitropyridinium salt and 1 equiv of the enaminone and it allowed the simultaneous formation of five new chemical bonds and eight stereocenters. A plausible mechanism for this transformation would involve an initial addition of the enaminone to the 4-position of the pyridinium salt, generating intermediate **A**. Another nucleophilic attack to the 4-position of a second molecule of 3-nitropyridinium salt would result in the formation of iminium ion intermediate **B** (Scheme 209). Then, an intramolecular Mannich reaction over the pyridinium salt would generate tricyclic intermediate **C**, which would undergo a final intramolecular [2 + 2] cycloaddition to render final products **431**. *N*-Aryl enaminones **429** gave better yields of the final products than *N*-alkyl enaminones. Acyclic derivatives were also good partners for this protocol.

**Scheme 210:**
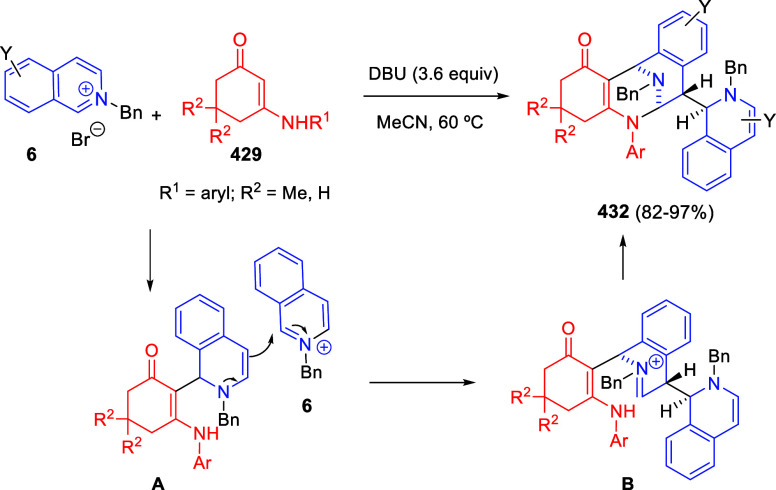
Base-Promoted Dearomative Trifunctionalization of Isoquinolinium Salts

This tandem protocol was also applied to isoquinolinium salts **6**, giving rise to polycycles **432** in good yields and excellent diastereocontrol, by means of a triple Mannich cascade sequence (Scheme 210).^283^ Different aryl enaminones **429** could participate in this transformation successfully.

**Scheme 211:**
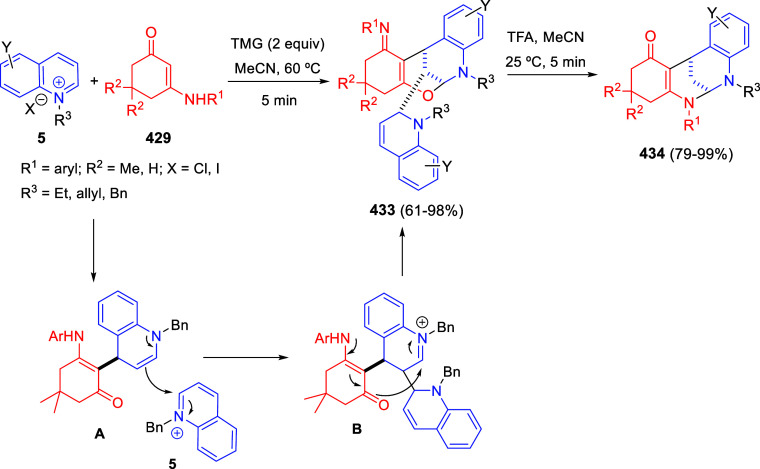
Base-Promoted Dearomative Tri- and Bifunctionalization of Quinolinium Salts

The analogous process starting from quinolinium salts **5** proceeded in a slightly different manner, so the C and O atoms of aryl enaminones **429** acted as nucleophilic reacting sites, giving rise to polycycles **433** in good yields with excellent diastereocontrol (Scheme 211).^283^ Then, treatment with TFA caused the *N*,*O*-ketal hydrolysis to yield bridged bicycles **434**. In this case, the initial 1,4-addition of the enaminone to the quinolinium salt would be followed by a Mannich-type reaction of enamine **A** with another equivalent of quinolinium salt **5**, rendering iminium salt **B**. Now, the oxygen atom would serve as an active nucleophilic site to attack the iminium ion via an intramolecular oxa-Mannich type reaction, furnishing trifunctionalized bridged cyclic products **433** (Scheme 211). Finally, in the presence of TFA, an acid-catalyzed sequential ring-opening/enamine-imine tautomerization/oxa-Mannich process would take place.

The same authors reported an analogous multicomponent dearomative multifunctionalization of pyridines, quinolines, and isoquinolines through an *in situ* activation strategy.^284^ In this case, the activation of the heteroarene was performed by means of an aryne precursor. In this manner, the authors accomplished the synthesis of bridged hydrogenated pyridines and (iso)quinolines in a highly regioselective and diastereoselective manner and also they could perform the dearomative trifunctionalization and bifunctionalization of quinolines.

**Scheme 212:**
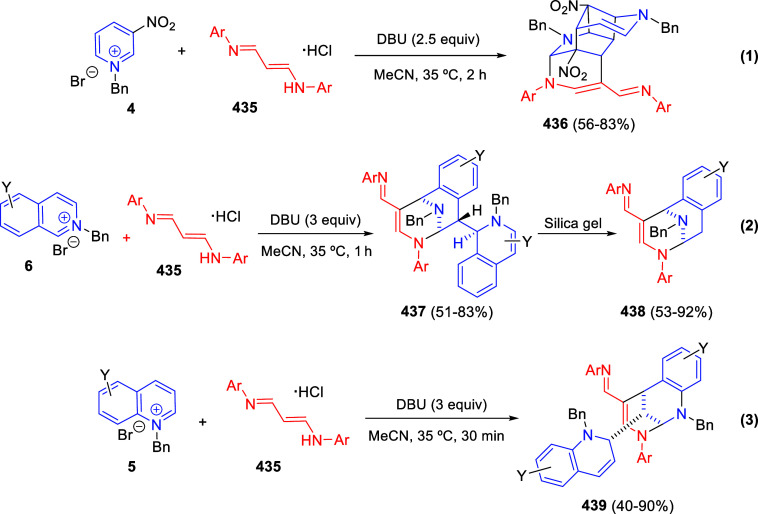
Diastereoselective Dearomative Multifunctionalization of *N*-Alkyl Activated Aza-arenes with 1,5-Diazapentadienium Salts

The group of Wang also reported an analogous strategy for the dearomative functionalization of pyridines, quinolines, and isoquinolines, employing in this case 1,5-diazapentadienium salts as 1,3-bis-nucleophiles (Scheme 212).^285^ Thus, 3-nitropyridinium bromide **4** reacted with diazadiene **435** in the presence of DBU to render products **436** in good yields (Scheme 212, eq 1). Isoquinolium salts **6** needed higher temperatures to afford trifunctionalized products **437**, which partially hydrolyzed in silica gel, furnishing compounds **438** in good yields (Scheme 212, eq 2). Finally, quinolinium salts **5** provided bridged *N*,*N*-ketals bearing partially and fully saturated quinoline skeletons **439** in good yields (Scheme 211, eq 3). In all cases, a variety of substituents at the aryl moieties of starting diazadienes were compatible with the process.

Moreover, the authors also realized the dearomative multifunctionalization of *N*-aryl aza-arenes through an *in situ* activation strategy, involving the use of 2-(trimethylsilyl)phenyl triflate as the benzyne precursor.^285^

**Scheme 213:**
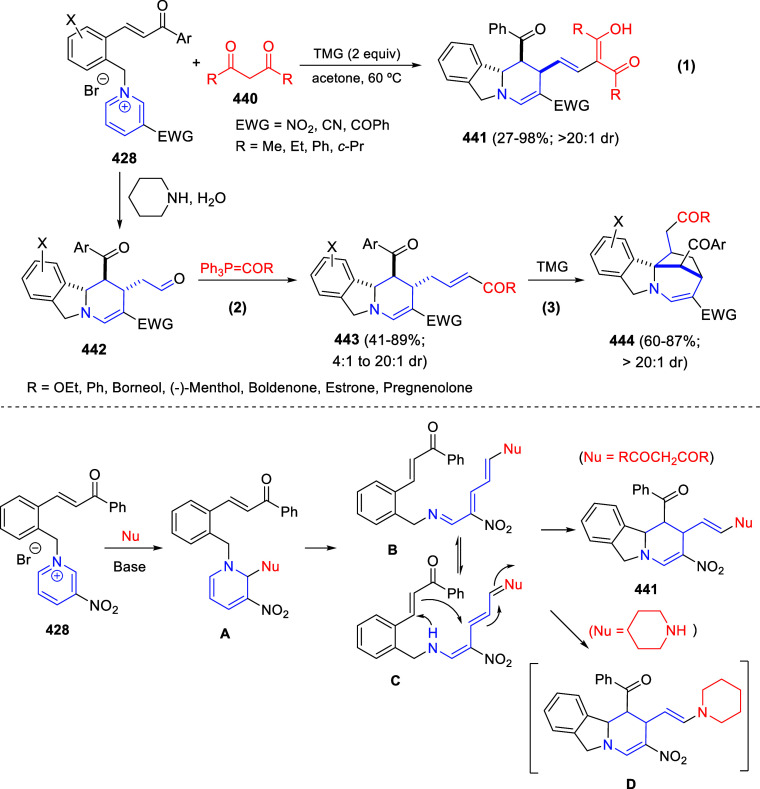
Skeletal Remodeling of Chalcone-Based Pyridinium Salts

In 2021, the groups of Wang, Zhao, and Zhang reported a skeletal remodeling strategy to transform chalcone-derived pyridinium salts into structurally complex polycyclic isoindolinones by means of a dearomative ring-opening/ring closing sequence (Scheme 213).^286^ When pyridinium salts **428** were treated with 1,3-diketones **440** in the presence of 1,1,3,3-tetramethyl guanidine (TMG) as a base, isoindoline-derived polycycles **441** were obtained in good yields as single diastereoisomers (Scheme 213, eq 1). Initially, the reaction was tested with pyridines bearing a nitro group at the C3-position, although other electron-withdrawing groups such as benzoyl or cyano groups were also compatible. Moreover, a wide range of pyridinium salts bearing substituents with different electronic properties at various positions were all tolerated in the process. Reagarding the nucleophilic counterpart, several alkyl and aryl diketones, including unsymmetrically substituted ones, were tested with high efficiency.

On the other hand, this skeletal remodeling strategy was also investigated with secondary amines as nucleophiles. Thus, when piperidine was employed, the ring opening/reconstruction process took place to render the corresponding enamines that were *in situ* hydrolyzed to the polycyclic aldehyde-containing isoindolinones **442** in good yields. These aldehydes were then trapped with several Wittig reagents, affording tricyclic derivatives **443** bearing a pendant conjugated ester or ketone moiety (Scheme 213, eq 2). The substitution effects of the aryl group neighboring the carbonyl group were evaluated, showing better yields with electron-donating groups than with electron-donating ones. Regarding the Wittig ylides, ester and acetophenone-derived ylides participated successfully in the cascade process, including ylides containing natural products and drug molecules. Finally, the addition of TMG led to bridged isoindoline polycycles **444** in a completely diastereoselective manner (Scheme 213, eq 3). Moreover, the Wittig reaction and the subsequent base-promoted cyclization could be performed in a tandem fashion.

Some control experiments and theoretical calculations were conducted in order to explain this ring opening/cyclization/Wittig sequence. The process would be initiated by the dearomative nucleophilic addition to the 2-position of the pyridinium salt to generate intermediate **A**, which would evolve with ring-opening to triene derivative **B**, in equilibrium with form **C** (Scheme 213). Then, an intramolecular cyclization reaction would render isoindoline-derived polycycles **441**. When piperidine was used as the nucleophile, after the (4 + 2) cycloaddition, intermediate **D** would be hydrolyzed to the corresponding aldehyde **442**. Upon reaction with the phosphorus ylide, compound **443** bearing an α,β-unsaturated ester group would undergo an intramolecular Michael addition to deliver the bridged polycycles **444**.

**Scheme 214:**
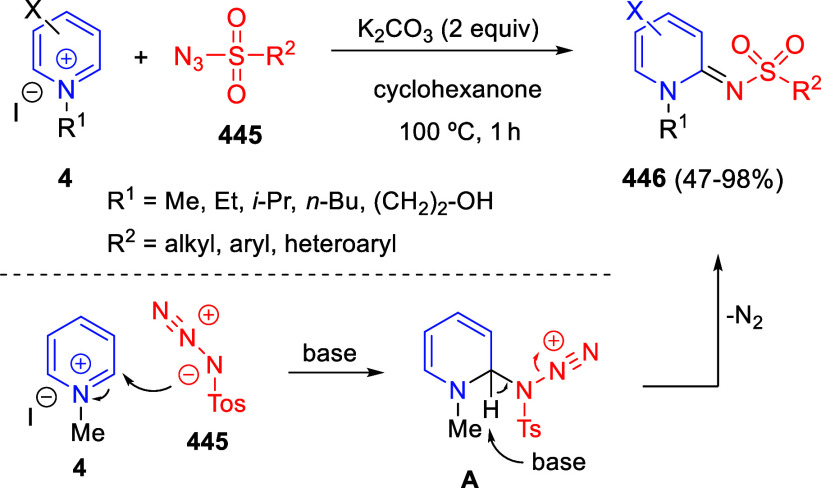
Direct C–H Sulfonylimination of Pyridinium Salts

Very recently, Fu and co-workers developed a highly efficient metal-free C–H sulfonylimination of pyridinium salts (Scheme 214).^287^ After optimization of the reaction conditions, treatment of pyridinium salts **4** with sulfonyl azides **445** in the presence of K_2_CO_3_ as a base in cyclohexanone at 100 °C furnished sulfonyl iminopyridines **446** in generally good yields. Different aryl and heteroaryl- 4-, 3-, and 2-substituted pyridinium salts, as well as pyridiniums bearing other functional groups such as ester, cyano, trifluoromethyl, methoxy or acetamide groups were compatible with the reaction. In addition, the quinolinium salt was also a suitable substrate; however, the isoquinolinium salt failed. Regarding the azide counterpart, several arylsulfonyl azides containing electron-donating groups at the *para*- or *meta*-positions gave the desired products in good to excellent yields, while substrates with electron-withdrawing groups resulted in lower to moderate yields.

The authors proposed a plausible mechanism based on control experiments, according to which the sulfonyl azide would add to the C2-position of the pyridine ring, forming intermediate **A**, from which a base-triggered elimination would occur, with concomitant release of N_2_, giving rise to final products (Scheme 214).

**Scheme 215:**
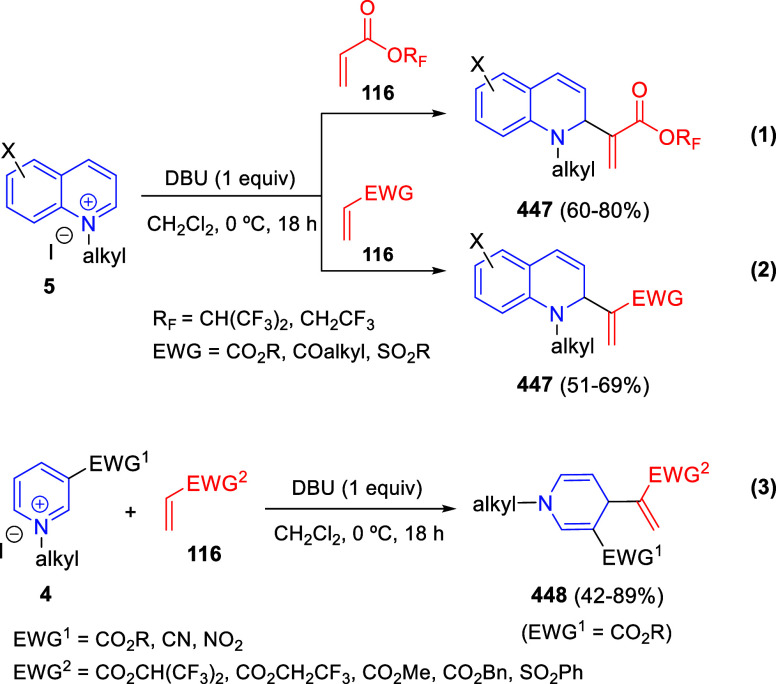
Regioselective Dearomatization of *N*-Alkylquinolinium and Pyridinium Salts under Morita–Baylis–Hillman Conditions

In 2022, Kalek and Pareek reported a base-promoted Morita–Baylis–Hillman reaction employing *N*-alkylquinolinium and pyridinium salts as electrophiles (Scheme 215).^288^ Quinolinium salts **5** reacted with fluorinated acrylates **116** in the presence of DBU to give dearomatized MBH-adducts **447** in a regioselective manner at the C2-position of the quinoline ring (Scheme 215, eq 1). The reaction was extended to other olefins bearing electron-withdrawing groups, providing again the C2-addition products (Scheme 214, eq 2). A broad range of α-(1,2-dihydroquinolin-2-yl)vinyl esters, ketones, and sulfones **447** were obtained in moderate to good yields.

The scope of this transformation was extended to *N*-alkylpyridinium salts **4**. In this case, the presence of an electron-withdrawing group at the C3-position was necessary to attain good reactivity and the dearomatization proceeded regioselectively at the C4-position when that electron-withdrawing group was an ester (Scheme 215, eq 3). On the other hand, with nitrile and nitro moieties, the nonselective formation of C4- and C6-addition products took place. Regarding the MBH reagent, alkyl and fluorinated acrylates and vinyl sulfones were good partners for the process.

**Scheme 216:**
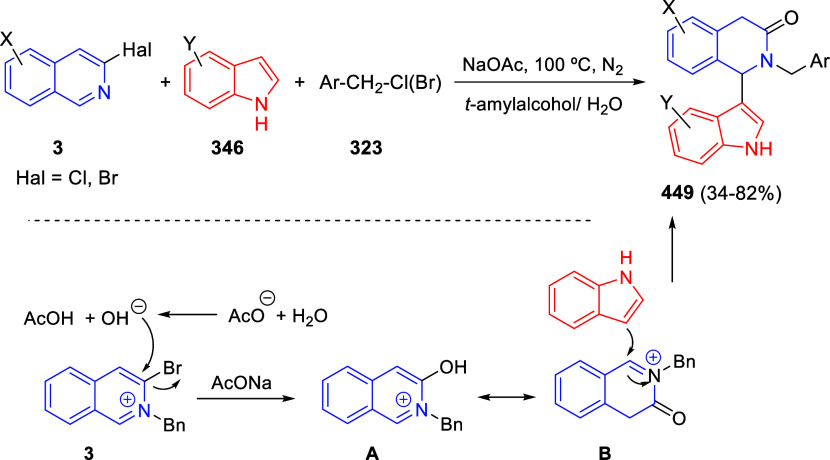
Nucleophilic Dearomatization of Isoquinolines via Three-Component Reaction of 3-Haloisoquinolines, Alkyl Halides, and Indoles

The three-component reaction of 3-haloisoquinolines **3**, alkyl halides **323**, and indoles **346** in the presence of AcONa as a base allowed the construction of disubstituted isoquinolinone derivatives **449** under metal-free conditions (Scheme 216).^289^ This transformation involved the trifunctionalization of the isoquinoline ring through a dearomatization strategy and it displayed high chemical selectivity and excellent functional group tolerance. Control experiments were performed in order to understand the mechanism of this transformation. According to the authors, the 3-bromoisoquinoline substrate would initially react with benzyl chloride to form the isoquinolinium salt. Simultaneously, the hydroxyl nucleophile would be generated under basic conditions and it would add to the isoquinolinium salt (Scheme 216). The resulting 3-hydroxyl quinolinium **A** would be in equilibrium with the iminium salt **B**, which would undergo Friedel–Crafts-type addition from the 3-position of the indole to furnish the final products.

**Scheme 217:**
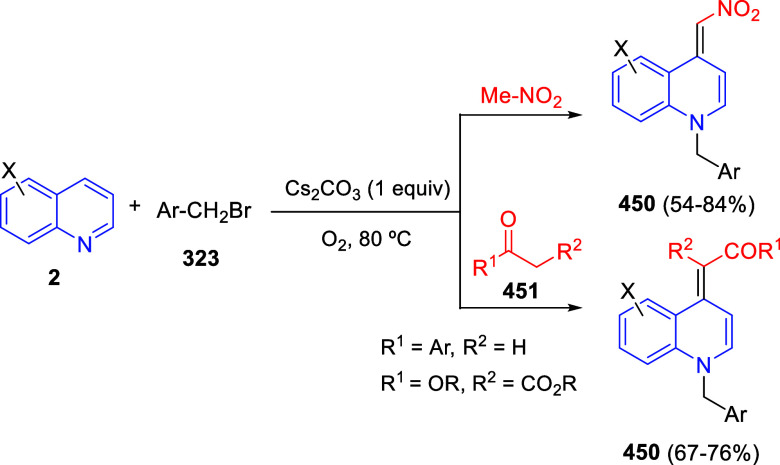
Selective 1,4-Difunctionalization of *In Situ*-Activated Quinolines with Nucleophiles

Chen and Zhu reported a three-component 1,4-difunctionalization reaction of quinoline and pyridine derivatives by means of their reaction with alkyl halides and active methylene/methyl compounds (Scheme 217).^290^ Thus, the solvent free reaction of quinolines **2**, benzyl bromides **323** and nitromethane under oxygen atmosphere in the presence of Cs_2_CO_3_ as a base afforded 1,4-dihydroquinolines **450** by means of a dearomative functionalization of the *in situ*-activated quinoline (Scheme 217, eq 1). Various quinolines bearing electron-withdrawing and/or electron-donating groups participated in this reaction, as well as pyridines bearing strongly electron-withdrawing groups (NO_2_ or CN) at the 3-position. Different benzyl halides and other alkyl halides with various functional groups also participated in the transformation. The process was further extended to other nucleophiles such as malonates or methyl aryl ketones **451**, giving the corresponding 1,4-dihydropyridines **450** in good yields (Scheme 217, eq 2). Regarding the reaction mechanism, quinoline would react with the benzyl bromide to form the quinolinium salt. Then, nucleophilic addition of the nucleophile to the 4-position would afford the functionalized quinoline, which would undergo oxidative dehydrogenation to give the stable conjugated diene product.

**Scheme 218:**
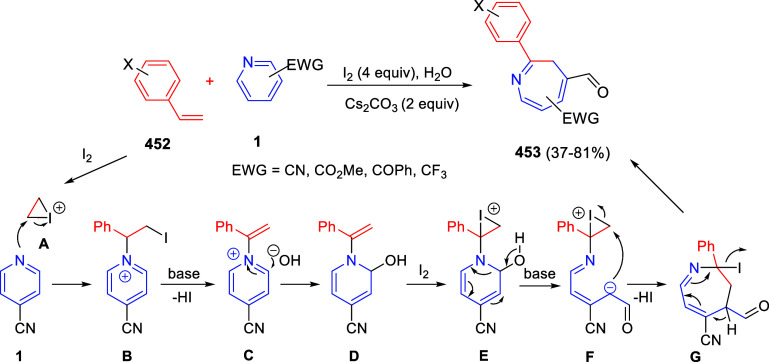
Iodine-Mediated Pyridine Ring Expansion for the Construction of Azepines

Huang and co-workers developed an iodine-mediated expansion of pyridine rings for the synthesis of azepine derivatives (Scheme 218).^291^ Specifically, treatment of styrenes **452** and pydirines **1** bearing an electron-withdrawing group with iodine and cesium carbonate furnished the desired azepines **453** in moderate to good yields. The reaction worked with pyridines bearing ester, cyano, aryl ketone of trifluoromethyl groups either at the C3- or C4-positions, although with CF_3_ the chemical yield was very low. Moreover, various electronically different substituents at the C3- and C4-positions of the styrene counterpart were compatible with the pyridine ring expansion.

Several experiments were carried out for the investigation of the reaction mechanism. In this context, styrene would react with iodine to form iodonium salt **A**, which would undergo ring-opening by attack of the pyridine nitrogen to render pyridinium salt **B** (Scheme 218). Then, the base-catalyzed dehydroiodination would lead to enamine **C**. Hydroxide addition to this pyridinium salt followed by electrophilic iodination of the resulting dienamine **D** would produce iodonium intermediate **E**. Further decomposition of this cyclic amino alcohol to enolate **F**, followed by intramolecular opening of the iodonium salt to form azepine **G** and dehydroiodination, would afford the final azepine derivatives **453**.

### 6.4 Other Types of Transition Metal-Free Dearomatizations

**Scheme 219:**
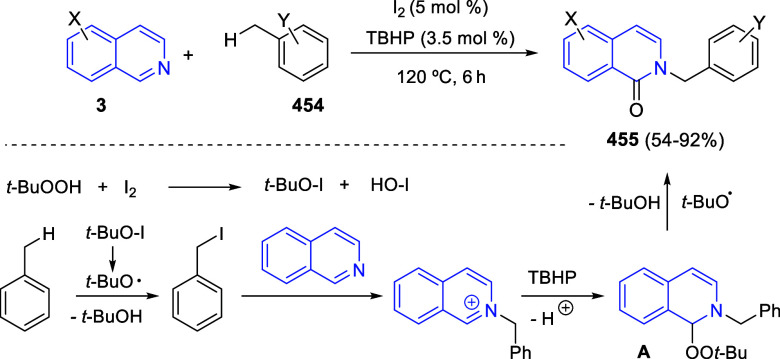
Iodine-Catalyzed Oxidative *N*-Alkylation/Amidation Cascade Reaction of Isoquinolines with Benzylic C–H Bonds

In 2016, Yang and co-workers developed a iodine-catalyzed oxidative functionalization of isoquinolines with benzylic C–H bonds by means of *N*-alkylation and amidation cascade process (Scheme 219).^292^ Thus, reaction of isoquinolines **3** with methylbenzenes **454** and catalytic amounts of iodine and *t*-butyl hydroperoxide (TBHP) afforded isoquinolinones **455** in good yields. Methylbenzenes bearing electron-donating or electron-withdrawing substituents were successfully transformed into the desired isoquinolinone products by reaction with differently substituted isoquinolines.

A plausible mechanism for this transformation would begin with the reaction of TBHP with molecular iodine, which would lead to the formation of *t*-BuOI and HOI. The benzylic iodination would proceed via homolytic attack with the *t*-BuOI radical, giving rise to benzyl iodide, which in turn would react with isoquinoline to form the isoquinolinium salt. Subsequent nucleophilic addition of TBHP would provide hemiaminal-type peroxide **A**, which would undergo O–O bond cleavage to afford the final isoquinolinone products (Scheme 219).

**Scheme 220:**
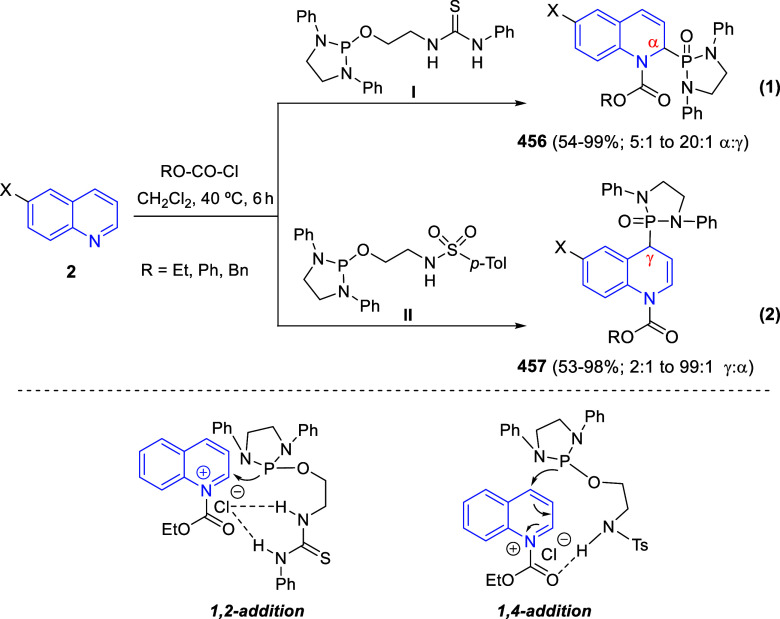
NHP-Promoted Regioselective Phosphonylation Reaction of Quinolines

Kang and co-workers described the regioselective phosphonylation of quinolines mediated by *N*-heterocyclic phosphines (NHPs) (Scheme 220).^293^ The reaction of quinolines, chloroformates, and NHP-thiourea **I** in dichloromethane at 40 °C afforded α-amino quinolinyl phosphonamides **456** by means of a Reissert-type reaction (Scheme 220, eq 1). Quinoline derivatives containing electron-donating and electron-withdrawing groups at the 6-position efficiently afforded the corresponding α-amino quinolinyl phosphonamides in moderate to good yields with high regioselectivity. On the other hand, when NHP-tosyl amide **II** was employed, an inversion of the regioselectivity occurred, affording γ-amino quinolinyl phosphonamides **457** via a 1,4-conjugate addition reaction (Scheme 220, eq 2). In general, quinolines with electron-donating groups exhibited higher regioselectivity than those with electron-withdrawing ones. Regarding the chloroformate counterpart, ethyl chloroformate provided the best regioselectivity in both cases, compared to phenyl- or benzyl chloroformates.

The high regioselectivity observed in the process could be ascribed to the H-bonding between the chloride anion and the thiourea motif through an ion-pairing process in the Reissert-type reaction, which would direct the addition to the C2-position of the quinoline when NHP-urea **I** was used. With other NHPs, this hydrogen-bonding is not possible, being more favorable the 1,4-addition pathway, activated by H-bonding between a carbonyl oxygen of the quinolinium and the tosylamide group (Scheme 220).

**Scheme 221:**
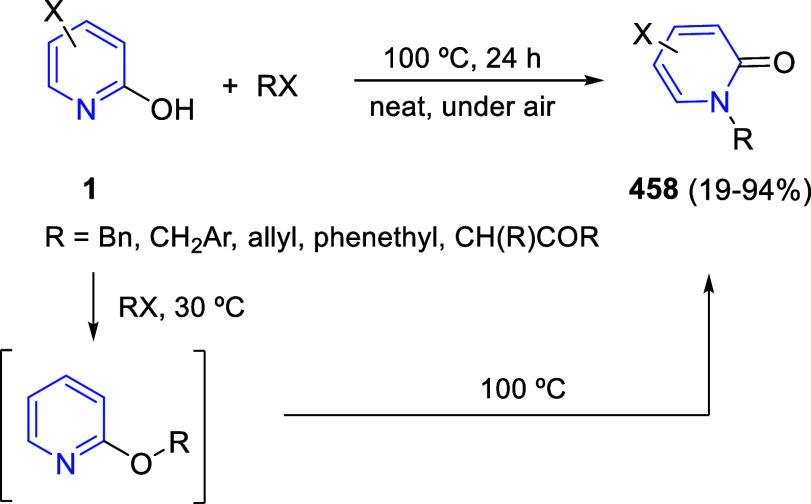
Selective *N*-Alkylation of 2-Hydroxypyridines with Organohalides under Catalyst and Base-Free Conditions

A selective *N*-alkylation of 2-hydroxypyridines was achieved by Xu and co-workers by means of the reaction with organohalides under solvent-, catalyst-, and base-free conditions (Scheme 221).^294^ Heating a mixture of 2-hydroxypyridines **1** and alkyl halides at 100 °C under air atmosphere gave rise to *N*-alkyl pyridones **458** in generally good yields with excellent *N*-selectivity. A wide variety of benzyl, primary, and secondary alkyl bromides were tolerated in the process, exhibiting good compatibility with other functional groups such as esters or ketones. Likewise, different substituents on the hydroxylpyridines were compatible with the process. When the reaction was performed at 30 °C, the *O*-alkylation product was formed, and this was converted into the *N*-alkyl pyridone at 100 °C, indicating that this was most likely the reaction pathway for the *N*-alkylation process (Scheme 221).

**Scheme 222:**
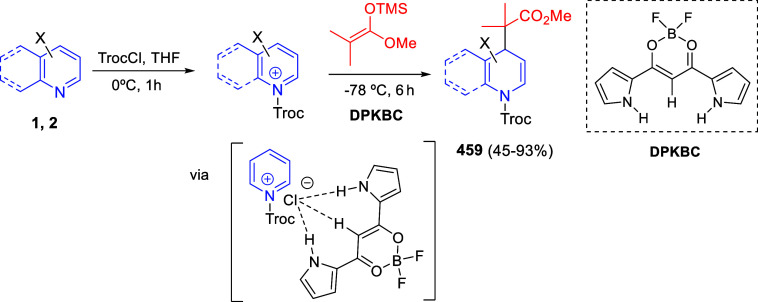
Pyrrole-Based Anion Binding Catalysis for the Mannich-Type Reaction of *N*-Acyl Heteroarenium Chlorides

Maeda and co-workers showed the ability of dipyrrolyldiketone boron complexes as hydrogen-bonding donor organocatalysts for the Mannich-type reaction of activated pyridines and quinolines with 1-methoxy-2-methyl-1-trimethylsiloxy-1-propene (Scheme 222).^295^ Initial treatment of pyridines **1** or quinolines **2** with 2,2,2-trichloroethyl chloroformate (TrocCl) afforded the corresponding *N*-acyl heteroarenium salts that in turn reacted with ketene silyl acetals in the presence of dipyrrolyldiketone boron complex (DPKBC), giving rise to the dearomatized 1,4-addition products **459** in good yields with high 1,4-selectivity. Substitution was allowed at the C6- and C3-positions of the quinoline ring and at the C2-position of the pyridine ring. ^1^H NMR experiments showed a hydrogen-bonding interaction between the pyrrole NH of the dipyrrolyldiketone boron catalyst and the chloride anion in the *N*-acyl heteroarenium salt, suggesting that the activation of the *N*-acyl heteroarenium chloride occurs through pyrrole-based anion binding catalysis (Scheme 222). Without the boron complex, the process took place in less than 10% yield. At the same time, this arrangement would block the 2-position of the heterocycle. After that, ketene silyl acetal would attack the heteroarenium electrophile to give the dearomatized products.

**Scheme 223:**
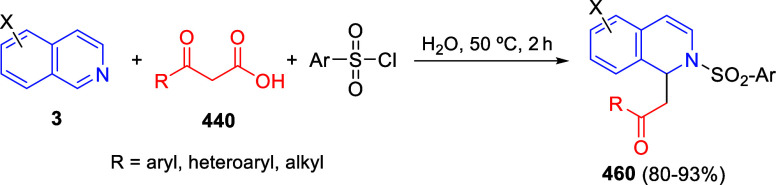
Catalyst-Free Decarboxylative Dearomatization of Isoquinolines with β-Keto Acids and Sulfonyl Chlorides in Water

In 2020, Hu and Jia described the three-component decarboxylative dearomatization of isoquinolines **3** with β-keto acids **440** and sulfonyl chlorides (Scheme 223).^296^ The combination of the three reagents in water at 50 °C provided dihydroisoquinolines **460** in excellent yields. Substitution at the C5- and C6-positions of the isoquinoline ring was allowed in the process, independently of the electronic nature of the substituents. Morevoer, alkyl, aryl, and heteroaryl keto acids gave excellent yields of the final products.

## 7 APPLICATIONS TO NATURAL PRODUCT SYNTHESIS

**Scheme 224:**
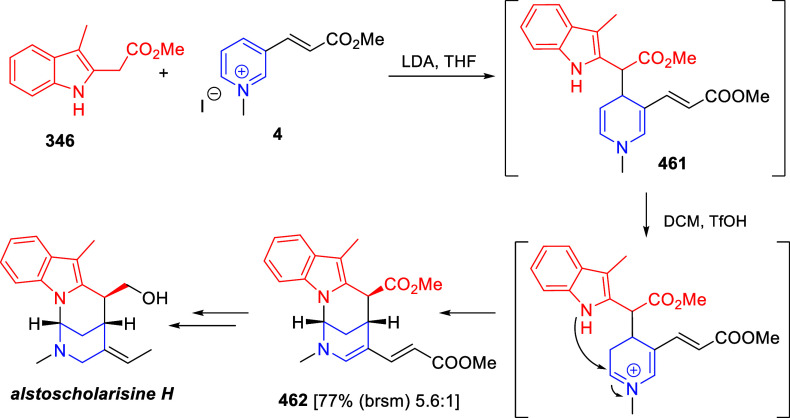
Dearomatization Strategy for the Synthesis of Alstoscholarisine H

Several authors have taken advantage of the *N*-heteroarene dearomatization strategy for the synthesis of natural products. For example, Xia and Lou employed a dearomative reaction of pyridines as a key step to the synthesis of alstoscholarisine H (Scheme 224).^297^ In this work, the enolate addition of indole ester **346** to pyridinium salt **4** took place with complete regioselectivity to give the 1,4-dihydropyridine derivative **461**. Upon treatment with triflic acid, the corresponding iminium ion evolved trough intramolecular attack of the indole nitrogen to render tetracycle **462**, containing the skeleton and the three stereocenters of the natural product.

**Scheme 225:**
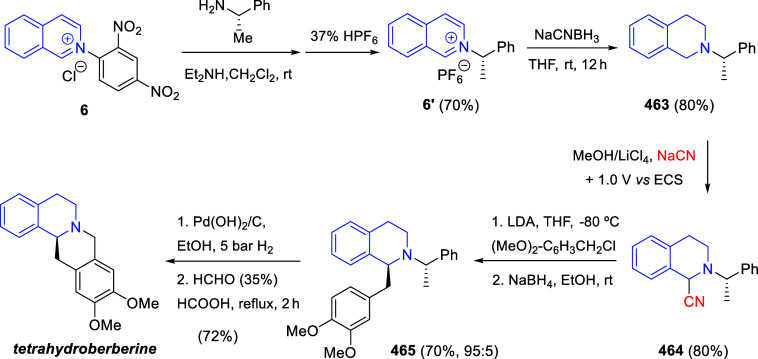
Dearomatization Strategy for the Synthesis of Tetrahydroberberine

In 2016, Hurvois and co-workers described the synthesis of tetrahydroberberine from Zincke salt **6** (Scheme 225).^298^ Its reaction with chiral phenylethylamine and subsequent treatment with HPF_6_ rendered the isoquinolinium hexafluorophosphate **6′**. The dearomatization of the isoquinoline ring was performed by reduction with NaCNBH_3_ to render tetrahydroquinoline **463**. The direct alkylation at the C1-position of the isoquinoline ring resulted difficult and the authors incorporated a cyano group by electrolysis instead. With compound **464** in hand, alkylation with the appropriate benzyl chloride followed by reductive decyanation with NaBH_4_ took place with excellent 1,3-steroinduction to render tetrahydroquinoline **465** in good yield. Finally, release of the chiral auxiliary was effected with Pearlman’s catalyst, and the cyclization to achieve the natural product was performed with formalin in acetic acid in good overall yield (Scheme 225).

**Scheme 226:**
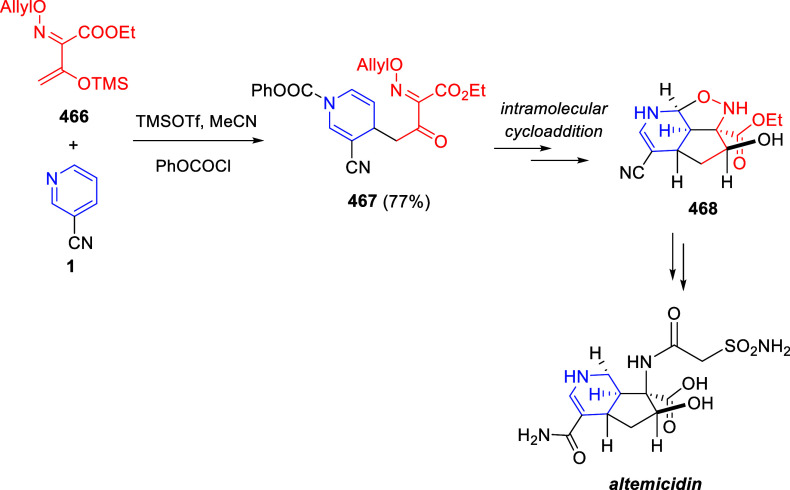
Dearomatization Strategy for the Synthesis of Altemicidin

More recently, Maimone and co-workers used a dearomative pyridinium addition followed by an intramolecular nitrone cycloaddition to create the skeleton of the natural product altemicidin (Scheme 226).^299^ Nitrile **1** was treated with silyl enol ether **466** in the presence of PhOCOCl as the activator of the pyridine ring and TMSOTf to promote the nucleophilic 1,4-addition that furnished dihydropyridine derivative **467** in 77% yield. Then, the *O*-allyl oxime moiety was transformed into the corresponding nitrone, which underwent an intramolecular 1,3-dipolar cycloaddition to render tricyclic derivative **468**, containing the skeleton of the desired natural product.

**Scheme 227:**
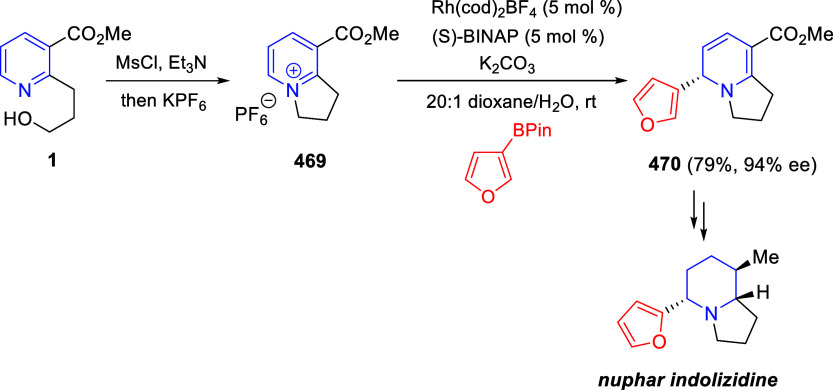
Dearomatization Strategy for the Synthesis of Nuphar Indolizidine

An enantioselective rhodium-catalyzed addition of boronic acids to pyridinium salts was employed by the group of Karimov as the key step in the total synthesis of nuphar indolizidine (Scheme 227).^240^ Starting from pyridine alcohol derivative **1**, mesylate formation and subsequent treatment with KPF_6_ provided the bicyclic pyridinium salt **469**. Then, the enantioselective addition of 3-furyl pinacol borane in the presence of rhodium catalyst Rh(cod)_2_BF_4_ and (*S*)-BINAP as the chiral ligand afforded 1,2-dihydropyridine derivative **470** in 79% yield and 94% ee. This compound contains the entire core of the natural product.

**Scheme 228:**
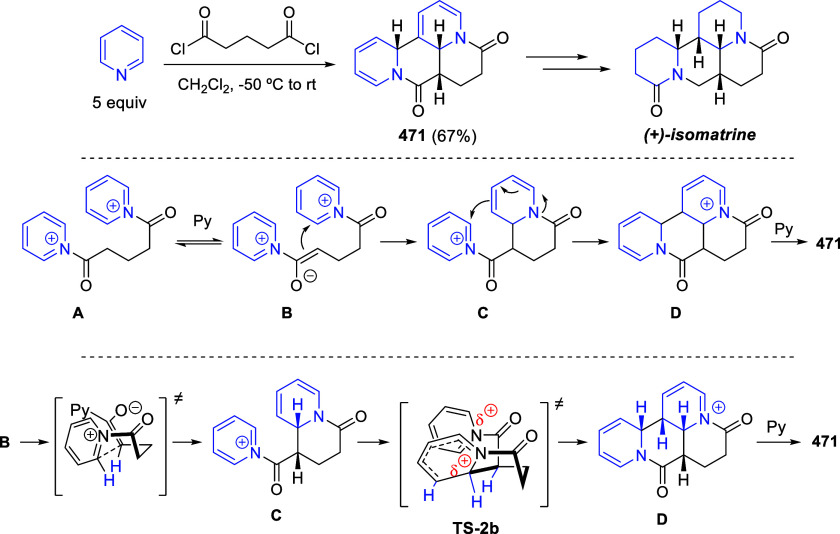
Dearomatization Strategy for the Synthesis of (+)-Isomatrine

Reisman and co-workers employed an annulative dearomatization of pyridine as the key step in the total synthesis of the tetracyclic alkaloid (+)-isomatrine (Scheme 228).^300^ In a bioinspired protocol, 5 equiv of pyridine reacted with glutaryl chloride to afford tetracyclic intermediate **471** that contains the whole skeleton of the natural product.

The authors performed DFT calculations to elucidate the reaction pathway. They found that the lowest energy TS for the first cyclization of intermediate **B** involved a syn-boat conformation (**TS1-***syn***-boat**) to form the syn product **C**. The lowest energy barrier for the **TS** for the second cyclization was **TS-2b** to form product **D**. Finally, deprotonation of intermediate **D** by pyridine followed the lowest energy pathway giving the *syn-syn* product **471** (Scheme 228).

Mechanistically, the reaction of gluratyl chloride with pyridine would form the bis-acylpyridinium intermediate **A** that, in the presence of another molecule of pyridine, would generate enolate **B**, which evolved by intramolecular addition over the internal pyridinium salt. Subsequent intramolecular addition of dienamine **C** to the pyridinium salt would render tetracycle **D**, which would isomerize with proton loss to the final product **471** (Scheme 228).

**Scheme 229:**
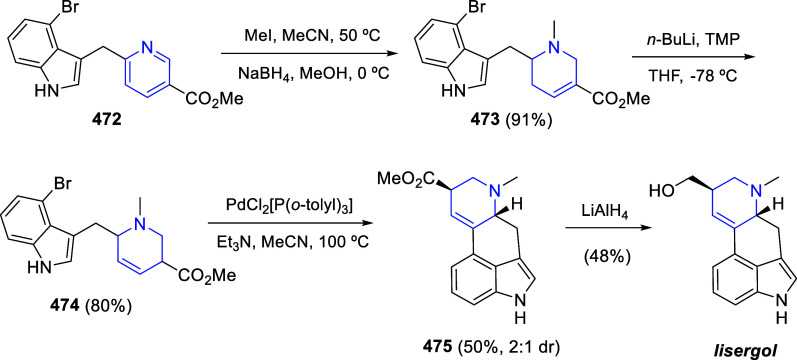
Dearomatization Strategy for the Synthesis of Lisergol

In 2022 the group of Wipf reported the synthesis of ergot alkaloids using a pyridine dearomatization and an intramolecular Heck reaction (Scheme 229).^301^ Indole derivative **472** was treated with MeI, and subsequent reduction of the pyridinium ring afforded tetrahydropyridine-derived indole **473** in 91% yield. Deprotonation followed by a kinetically controlled α-protonation of the ester dienolate rendered deconjugated ester **474**. This substrate underwent the intramolecular Heck reaction, which yielded a 2:1 mixture of diastereoisomeric tetracycles **475**. Finally, the reduction of the major isomer gave the natural product lysergol.

**Scheme 230:**
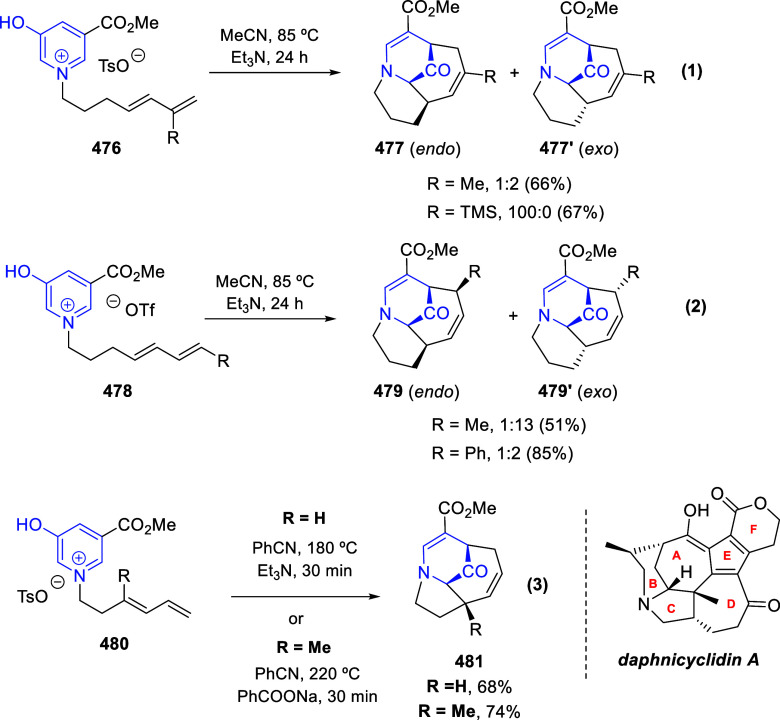
Dearomatization Strategy for the Synthesis of Daphnicyclidin A

As a final example, Harmata and co-workers recently developed the synthesis of the ABC ring system of daphnicyclidin A using an intramolecular [4 + 3] cycloaddition reaction of an oxidopyridinium ion (Scheme 230).^302^ Initially, the authors studied substrates with a three-carbon tether attached to the pyridinium ion. Thus, heating pyridinium substrates **476**, with dienes substituted at the C2-position, in the presence of Et_3_N as a base, triggered an intramolecular (4 + 3) cycloaddition to render a mixture of *endo***477** and *exo***477′** tricycles in good yields. The incorporation of the TMS group afforded exclusively the *endo* adduct (Scheme 230, eq 1). However, with substrates **478**, containing dienes substituted at the C1-position, the corresponding *exo* adducts **479′** were isolated as the major products (Scheme 230, eq 2). With these data in hand, the authors optimized the conditions to perform the cycloaddition on a substrate with a two-carbon tether. Accordingly, unsubstituted diene **480** (R = H) cyclized at 180 °C to render the tricyclic derivative **481** in 68% yield with complete *exo* selectivity. For the synthesis of the natural product daphnicyclidin A, the reaction was tested with the diene **480** (R = CH_3_) bearing a methyl group at the C4-position. After some optimization of the reaction conditions, the authors found that when the cycloaddition was performed at 220 °C in the presence of PhCOONa as a base, the *exo* adduct **481** was obtained in 74% yield as a single diastereoisomer (Scheme 230, eq 3). This compound contains the adequate stereochemistry of the tricyclic ABC system of the natural product.

The same authors employed an analogous strategy involving an intermolecular (4 + 3) cycloaddition of oxopyridinium ions with dienes, to obtain cocaine and scopolamine derivatives by means of a photochemically driven intramolecular (2 + 2) cycloaddition.^303^

## 8 CONCLUSIONS AND OUTLOOK

Bearing in mind the contributions of the last seven years included in this review, it is clear that the dearomatization reaction of pyridines, quinolines, and isoquinolines represents a hot research topic. A wide variety of methodologies have been devised in order to overcome the aromaticity barrier, at the same time installing functionality in a regio- and diastereoselective manner. Although impressive efforts have been directed to the synthesis of dihydro- and tetrahydro-derivatives, research activity in this area is beyond these strategies as multiple functionalizations of *N*-heteroarenes have been described, giving rise to complex architectures and generating molecular diversity with high levels of efficiency. It is quite obvious to expect that, in the next years, we will witness the development of new and innovative dearomatization methodologies that will enhance the utility of these transformations. Some of the challenges that lie ahead in the future of dearomative methodologies are commented below.

- Hydroboration and hydrosilylation protocols normally are directed to the synthesis of dihydro- or tetrahydroarenes. They reached an impressive level of efficacy in terms of regio-, diastereo-, and enantioselectivity, and several metal-catalyzed and metal-free methodologies have been devised to obtain these valuable intermediates. Despite the high development of this area, it is surprising that most examples are related to pyridines and quinolines, while the use of isoquinolines in those reactions clearly lagged behind. Although it is not easy to find an explanation for this fact, it seems that isoquinolines are less favorable substrates for hydroborations or hydrosilylations. Moreover, the combination of these reactions with other transformations in a tandem manner to perform multiple functionalizations of the heteroarenes is also very scarce to date. Therefore, a careful design of new starting materials increasing the complexity of the transformations combined with hydroborations and hydrosilylations will be desirable.
- The participation of pyridines, quinolines, and isoquinolines in cycloaddition reactions and dipolar cycloadditions of several orders is extraordinary, allowing for the generation of complex skeletons with simultaneous creation of multiple stereocenters. This is one of the best ways to achieve multiple functionalizations with the dearomative protocols. Most likely one of the questions that still arises in those transformations is that, in most cases, the reactions are performed in a racemic manner. Enantioselective examples in cycloaddition-type reactions are still insufficient, probably due to the fact that the background reaction in all those processes is very strong and the participation of a chiral catalyst is not obvious. Therefore, the development of new enantioselective protocols is a challenging task that still have to be improved.
- Intramolecular reactions offer a powerful way to construct complex polycyclic scaffolds with generally good control of the stereoselectivity, being also more favorable from an entropic point of view. Those features were employed in dearomative processes, being able to generate a wide variety of heterocycles under mild conditions. The design of new starting materials with the adequate functionality will be crucial to develop new methodologies that take advantage of the intramolecularity. Additionally, examples of asymmetric versions are still scarce and more work has to be done in this direction.
- On the topic of photocatalyzed dearomative reactions, a few examples of the application of this methodology for the dearomatization of heteroarenes are known. Despite the dazzling development of the photoredox catalysis in the past decade, the number of examples described in the bibliography related to dearomative protocols in comparison with other transformations is scarce, especially in the ones promoted by direct light absorption. In this context, the use of pyridines, quinolines, and isoquinolines for the formation of an electron donor–acceptor (EDA) complexes could be an interesting line of research to facilitate these reactions,^304^ as those complexes will be able to absorb visible light to initiate dearomatization events.
- Regarding Section 6, which covers mostly dearomatizations initiated by an intermolecular reaction, either promoted by a metal, a base, or an organocatalyst; in all cases, those transformations reached an extraordinary level of complexity, creating a wide variety of different heterocyclic scaffolds and exemplifying the robustness of this methodology as a synthetic tool. Although great advanced have been made, the preparation of highly substituted functionalized pyridines, quinolines, and isoquinolines still remains a challenge, which could be addressed using these transformations. We hope that the present review stimulates new discoveries in the field.

In general, dearomatization reactions of heteroarenes are already making notable impact on organic and medicinal chemistry. We are convinced that this trend will increase in the following years, offering innovating opportunities for the development of new synthetic methodologies.
